# Catalytic asymmetric hydroxylative dearomatization of 2-naphthols: synthesis of lacinilene derivatives†
†Electronic supplementary information (ESI) available. CCDC 1536822. For ESI and crystallographic data in CIF or other electronic format see DOI: 10.1039/c7sc02809a



**DOI:** 10.1039/c7sc02809a

**Published:** 2017-07-24

**Authors:** Yu Zhang, Yuting Liao, Xiaohua Liu, Xi Xu, Lili Lin, Xiaoming Feng

**Affiliations:** a Key Laboratory of Green Chemistry & Technology , Ministry of Education , College of Chemistry , Sichuan University , Chengdu 610064 , China . Email: liuxh@scu.edu.cn ; Email: xmfeng@scu.edu.cn ; Fax: +86 28 85418249 ; Tel: +86 28 85418249

## Abstract

An enantioselective hydroxylative dearomatization of 2-naphthols with oxaziridines has been accomplished using a *N*,*N*′-dioxide–scandium(iii) complex catalyst. Various substituted *ortho*-quinols could be obtained in high yields (up to 99%) and enantioselectivities (up to 95 : 5 er). This methodology could be applied in the synthesis of bioactive lacinilenes in a gram-scale reaction. Based on the experimental investigations and previous work, a possible catalytic model was proposed.

## Abstract 2

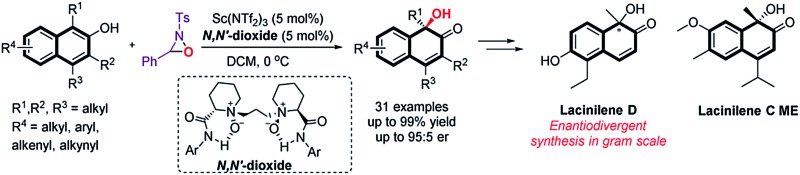
A highly enantioselective hydroxylative dearomatization of 2-naphthols with oxaziridines has been accomplished using an *N*,*N*′-dioxide–scandium(iii) complex catalyst. This methodology could be applied in the synthesis of bioactive lacinilenes in a gram-scale reaction.

## Introduction

**Fig. 1:**
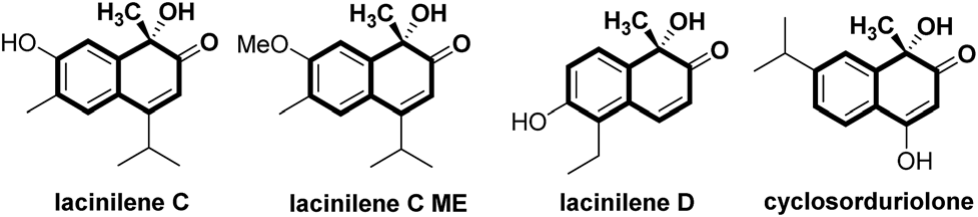
Representative active lacinilene derivatives bearing *ortho*-quinol structures.

Substituted *ortho*-quinols are essential structural motifs in a number of natural products and pharmaceuticals.^1^ For instance, chiral lacinilene derivatives (Fig. 1), a series of phytoalexines isolated from cotton plants, have been utilized for inhibiting the growth of cotton bacterial pathogens, such as *Xanthomonas campestris* or *malvacearum*.^2^ Studies have showed that the (*S*)-enantiomer of lacinilene C is more active than the (*R*)-enantiomer.^2*c*^ While these biological activities provide a justification for the development of approaches to the synthesis of enantiomerically enriched lacinilene derivatives, novel catalytic enantioselective methods remain limited.^2b,d^

**Scheme 1:**
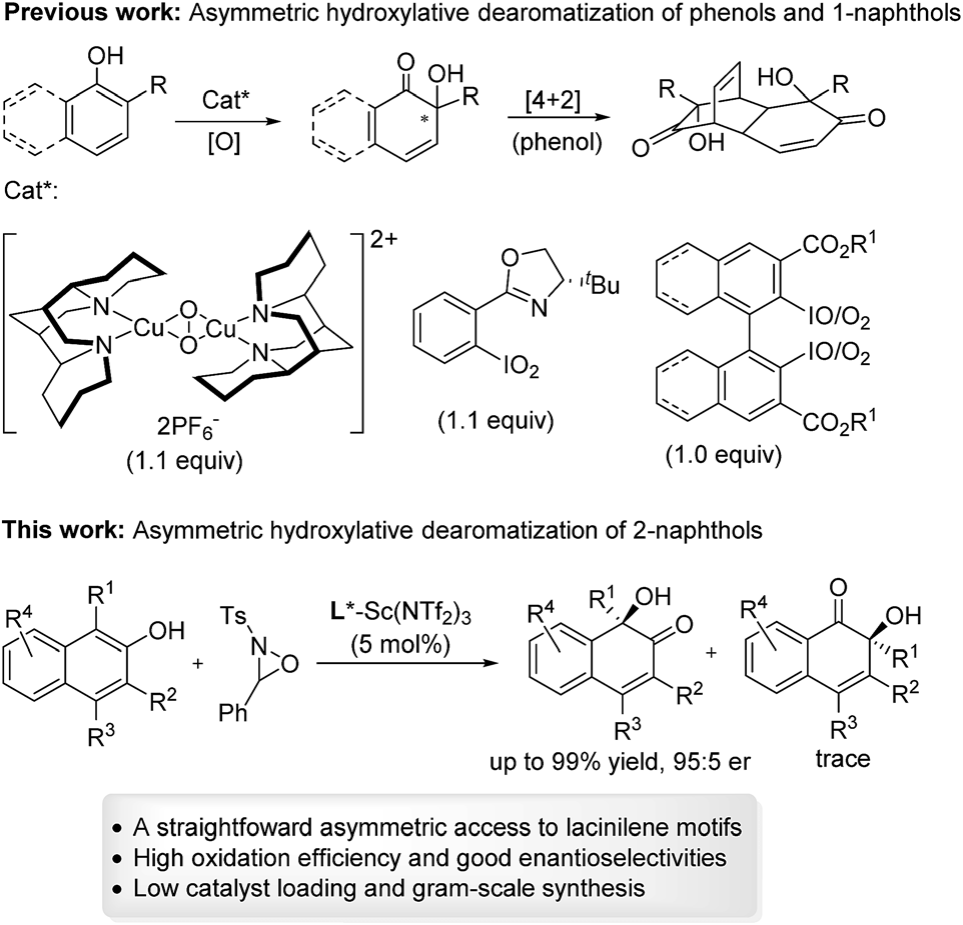
Catalytic asymmetric hydroxylative dearomatization of phenols and naphthols.

Optically active lacinilene derivatives in nature were proposed to be produced enzymically from the oxidation of dihydroxycadalenes, thus it is of practical interest to discover a catalytic asymmetric oxidative dearomatization route to the synthesis of these cadinanes.^3^ Compared with other successful dearomatization events of phenols or naphthols,^4,5^ controlling the chemo-, regio- and enantioselectivity of the asymmetric hydroxylative dearomatization is more difficult,^6^ as there might be serious side reactions in the presence of oxidants including overoxidation of alkene functions, competitive *para*-oxidation and homocoupling.^6c,e^ Additionally, the *ortho*-quinol product could undergo an unexpected α-ketol rearrangement, which enhances the difficulty of controlling the reactivity and selectivity.^6a,7^ In this respect, only a few reports related to asymmetric hydroxylative dearomatization of phenols or naphthols have been reported. Asymmetric oxidative dearomatization of phenolate mediated by copper–sparteine–dioxygen complexes followed a [4 + 2] dimerization cascade, giving bicyclo[2.2.2]octenones as the final products.^6*a*^ Several chiral hypervalent organoiodine compounds were developed for the asymmetric hydroxylative dearomatization of phenols and 1-naphthols.^6b–e^ Taking these examples into account, we want to engage in discovering new enantioselective strategies for the synthesis of *ortho*-quinol moieties with improved efficiency and selectivity. Here, we present an efficient asymmetric hydroxylative dearomatization of 2-naphthols catalyzed by a chiral *N*,*N*′-dioxide–scandium(iii) complex catalyst.^8^ The process could be applied to the synthesis of various 1-hydroxy-1-alkyl-naphthalen-2-one derivatives including lacinilene C methyl ether and lacinilene D, in high to excellent yields and good enantioselectivities under mild reaction conditions (Scheme 1).

## Results and discussion

We selected the hydroxylative dearomatization of 1-methylnaphthalen-2-ol **1a** as the model substrate using 3-phenyl-2-tosyl-1,2-oxaziridine **2a** as the oxidant which was proven to be chemoselective as a phase-transfer-catalyst under basic conditions (Table 1).^7*a*^ Initially, the catalytic asymmetric reaction was performed with 10 mol% of chiral *N*,*N*′-dioxide **l-PiPr_2_**–Sc(OTf)_3_ complex in DCM at 30 °C, and the desired product **3a** could be obtained dominantly with 80 : 20 er while the α-ketol rearrangement byproduct **4a** was isolated in around one-fourth of a 96% total yield (Table 1, entry 1). The evaluation of the structure of the *N*,*N*′-dioxides showed that **l-PiPr_2_** was the optimal ligand in terms of the enantioselectivity albeit ligand **l-PiMe_2_** and **l-PiPr_3_** improved the yield of the desired product **3a** (entries 2–5). Fortunately, changing the counterion of the scandium salt from ^–^OTf to ^–^NTf_2_ could suppress the α-ketol rearrangement, delivering the quinol **3a** in a 99% yield with 92 : 8 er (Table 1, entry 6). Further optimization of the reaction conditions, such as decreasing the temperature and the catalyst loading to 5 mol%, resulted in slightly improved enantioselectivity with maintained efficiency (entry 7). Lowering the catalyst loading to 1 mol% or the amount of the oxidant **2a** decreased either the yield or the selectivity a little (entries 8 and 9). We therefore chose the reaction conditions in Table 1, entry 7 for further studies.

**Table 1:** Optimization of the reaction conditions^*a*^

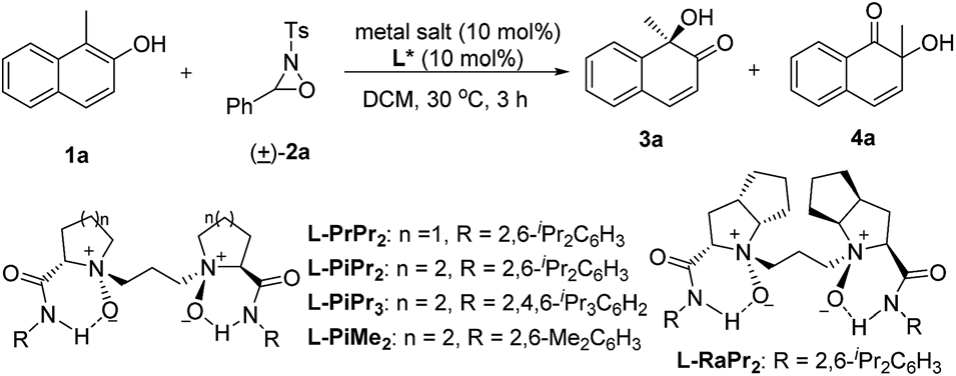					
Entry	Metal salt	l*	Yield^*b*^ (%)	Ratio (**3a**/**4a**)^*c*^	er (**3a**)^*c*^
1	Sc(OTf)_3_	**l-PiPr_2_**	96	73 : 27	80 : 20
2	Sc(OTf)_3_	**l-PrPr_2_**	99	79 : 21	63 : 37
3	Sc(OTf)_3_	**l-RaPr_2_**	99	75 : 25	53.5 : 46.5
4	Sc(OTf)_3_	**l-PiMe_2_**	90	>95 : 5	60 : 40
5	Sc(OTf)_3_	**l-PiPr_3_**	96	89 : 11	73 : 27
6	Sc(NTf_2_)_3_	**l-PiPr_2_**	99	>95 : 5	92 : 8
7^*d*^	Sc(NTf_2_)_3_	**l-PiPr_2_**	99	>95 : 5	95 : 5
8^*e*^	Sc(NTf_2_)_3_	**l-PiPr_2_**	86	>95 : 5	93.5 : 6.5
9^*d*^ ^,^ ^*f*^	Sc(NTf_2_)_3_	**l-PiPr_2_**	99	>95 : 5	94.5 : 5.5

^*a*^Unless otherwise noted, the reactions were performed with l*/Sc(iii) (1 : 1, 10 mol%), **1a** (0.10 mmol) and **2a** (2.0 equiv.) in DCM (1.0 mL) under N_2_ at 30 °C for 3 h.

^*b*^Isolated yield by silica gel chromatography.

^*c*^Determined by chiral HPLC analysis.

^*d*^5 mol% catalyst loading at 0 °C.

^*e*^1 mol% catalyst loading at 0 °C for 4 h.

^*f*^ **2a** (1.5 equiv.) was used.

We next explored the substrate scope of 2-naphthols (Table 2). The introduction of bromo or methoxyl groups at the C6-position of 2-naphthols had no obvious effect on the result. The 6-aryl substituted 2-naphthol derivatives **1d–1l** tethering various electron-donating and electron-withdrawing substituents could undergo the transformations smoothly, providing the products **3d–3l** in 95–99% yield and 93.5 : 6.5–95 : 5 er. It was noteworthy that 6-alkenyl and alkynyl substituted substrates **1m–1q** were compatible with the reaction conditions, and no aminohydroxylation of the unsaturated carbon–carbon bond occurred, giving the hydroxylative dearomatization products **3m–3q** in good to excellent yields and enantioselectivities.^9^ Additionally, 6-alkyl substituted 2-naphthols **3r–3w** bearing methyl, ethyl, and butyl groups were well tolerated, accomplishing the asymmetric hydroxylative reaction with the outcomes of 95–99% yield and 94 : 6–95 : 5 er. The installation of substituents to the 5- and 7-positions did not influence the reaction efficiency (**3x–3z**). The MOM-protected substrate **1z** could deliver the desired product **3z** with good results without any deprotection process occurring under the reaction conditions. However, the increase of steric hindrance at the *ortho*-position of 2-naphthol was harmful as a consequence (**3aa** and **3ab**).

**Table 2:** Substrate scope for 2-naphthols^*a*^

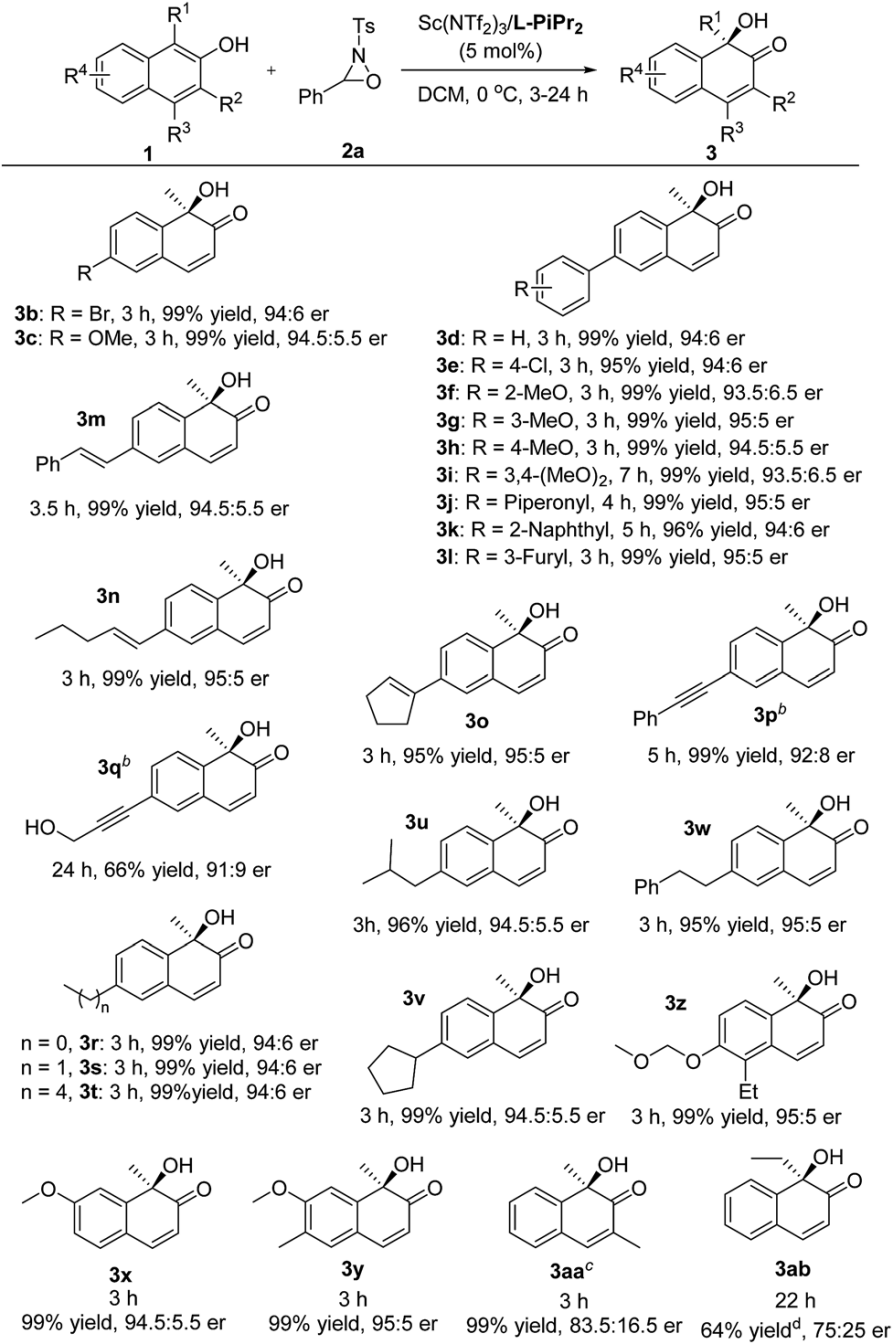

^*a*^Reaction conditions: the same as entry 7 in Table 1.

^*b*^10 mol% catalyst loading.

^*c*^ **l-PiEt_2_**–Sc(OTf)_3_ (1 : 1, 5 mol%).

^*d*^Total yield of **3ab** and **4ab**, **3ab**/**4ab** = 87 : 13.

**Scheme 2:**
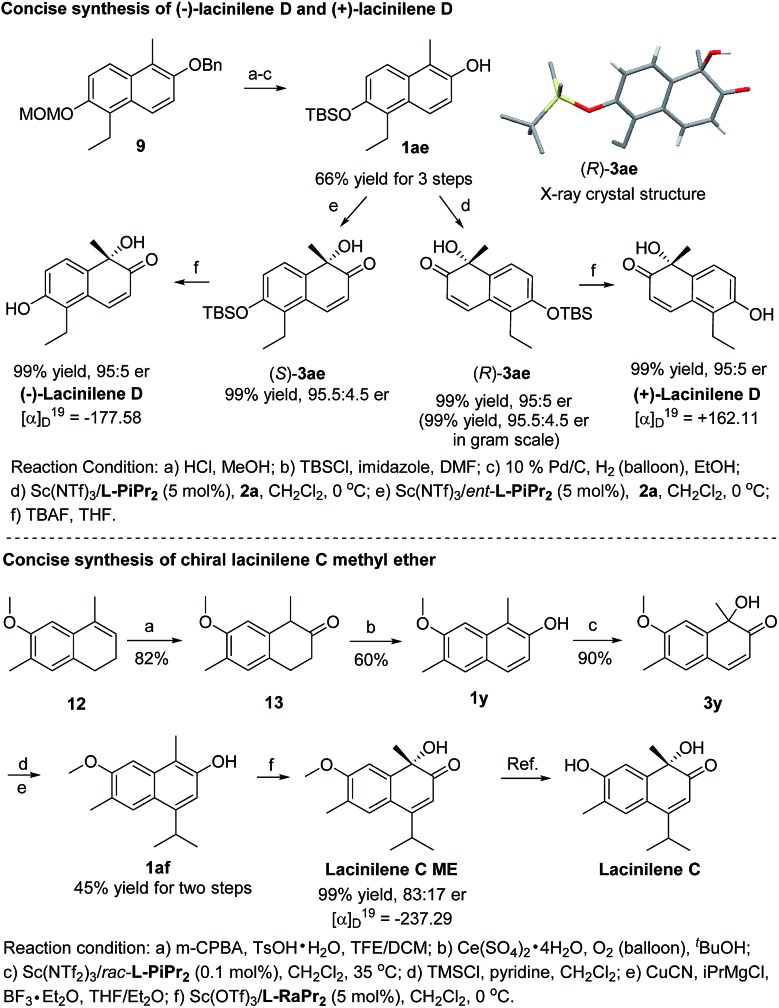
Concise synthesis of chiral lacinilene C methyl ether, (–)-lacinilene D and (+)-lacinilene D.

To show the synthetic utility of the current catalyst system, asymmetric synthesis of bioactive lacinilenes was carried out (Scheme 2). Initially, the direct deprotection of the product **3z** under acidic conditions formed the optically active lacinilene D, but an aromatization side product 1-ethyl-5-methylnaphthalene-2,6-diol was obtained.^2d,10^ It was anticipated that the TBS protecting group could be easily removed under neutral conditions, which might avoid the occurrence of the aromatization process. As expected, the TBS-substituted 2-naphthol **1ae** could be easily synthesized from **9** in 66% yield after 3 steps, which was further enantioselectively oxidized into the product **3ae** in quantitative yield and 95 : 5 er, even when it was performed at the gram scale. The absolute configuration of **3ae** from **l-PiPr_2_**–Sc(NTf_2_)_3_ complex catalysis was determined to be (*R*) by X-ray crystal diffraction analysis.^11^ For the benefit of the further differential biological activity study on each enantiomer of the chiral lacinilenes,^2*c*^ (*S*)-lacinilene D was synthesized using an *ent*-**l-PiPr_2_**–Sc(NTf_2_)_3_ complex with a comparable result of 99% yield and 95 : 5 er. Next, the synthesis of optically active lacinilene C methyl ether was explored. The synthetic route began from 1,2-dihydronaphthalene **12**, which could be easily accessed from 2-methoxytoluene through a four-step protocol.^2*d*^ Subsequent two-step oxidation could afford the 2-naphthol derivative **1y** in 49% yield, which underwent hydroxylative dearomatization catalyzed by 0.1 mol% of the Sc(NTf_2_)_3_/*rac*-**l-PiPr_2_** complex to produce racemic lacinilene **3y** in 90% yield.^2*d*^ After trimethylsilylation and copper catalyzed 1,4-addition/aromatization, 2-naphthol **1af** could be attained in 45% yield after two steps. By treatment with oxaziridine **2a** in the presence of Sc(OTf)_3_/**l-RaPr_2_**, chiral lacinilene C methyl ether could be obtained in quantitative yield and 83 : 17 er, which could further transform to lacinilene C according to the literature.^2*b*^

**Scheme 3:**
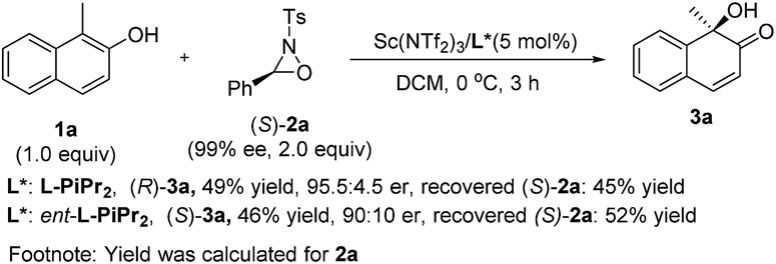
Control experiments.

To elucidate the stereochemical course of the oxidation process, some control experiments were conducted (Scheme 3). The optically pure oxaziridine (*S*)-**2a** reacted with 2-naphthol **1a** in the presence of the Sc(NTf_2_)_3_/**l-PiPr_2_** complex, affording the (*R*)-quinol **3a** in 49% yield and 95.5 : 4.5 er with the recovered oxaziridine (*S*)-**2a** in 45% yield.^12*d*^ Using *ent*-**l-PiPr_2_** as the ligand, (*S*)-quinol **3a** was obtained in 46% yield and 90 : 10 er with the recovered oxaziridine (*S*)-**2a** in 52% yield. This indicates that the chiral matched and mis-matched effect between chiral ligand and chiral oxaziridine was not obvious in this case compared to previous reports,^12^ and there might be negligible interaction between the chiral catalyst and oxaziridine.

**Fig. 2:**
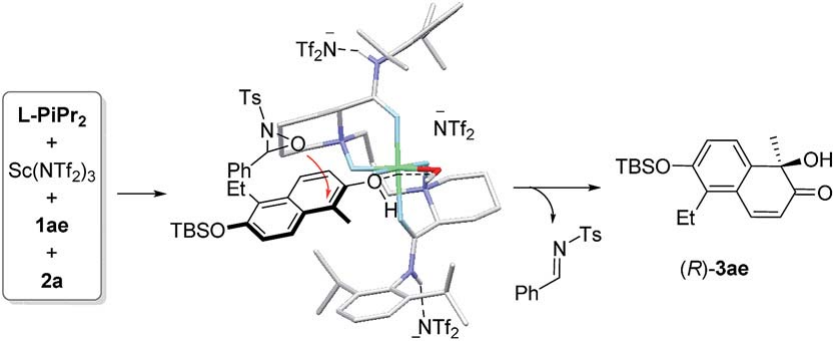
Proposed enantioselective catalytic model.

To probe into the interaction between the catalysts and 2-naphthol, ^1^H NMR analysis of the mixture of components was carried out (see ESI† for details). The chemical shift of 1-methyl 2-naphthol **1a** remained nearly unchanged after Sc(NTf_2_)_3_ was added. There was an obvious high-field shift for most signals of **1a** after mixing with the Sc(NTf_2_)_3_/**l-PiPr_2_** catalyst. This indicates that the chiral catalyst makes the 2-naphthol reactive for hydroxylative reactions. Based on these results and our previous study on the chiral *N*,*N*′-dioxide–metal complex catalysts,^8,13^ we suggested an enantioselective catalytic model as shown in Fig. 2. The ligand **l-PiPr_2_** binds to the scandium(iii) center *via* four oxygens to form a polycyclic octahedral metal complex catalyst. The 2-naphthol coordinates to the metal center at one of the vacant sites, with its *Re*-face shielded by one amide unit of the ligand. Therefore, **2a** preferably attacked the α-position of 2-naphthol from the *Si*-face to generate the corresponding *R*-configured product **3ae** and imine byproduct. If a substituent was introduced into the C3 or C4 positions of 2-naphthol, the steric hindrance discrimination between the two sides of the hydroxyl group decreases, thus it is difficult to control the face-selection. As a result, the enantioselectivity for the generation of product **3aa** and lacinilene C methyl ether is lower than that for the others.

## Conclusions

In summary, we have described a highly chemo- and enantioselective hydroxylative dearomatization of 2-naphthol derivatives with oxaziridine catalyzed by a chiral *N*,*N*′-dioxide–Sc(NTf_2_)_3_ complex catalyst. The desired substituted *ortho*-quinols with one quaternary carbon stereogenic center were afforded with high enantioselectivities and reactivity (up to 99% yield and 95 : 5 er). The α-ketol rearrangement byproducts were efficiently suppressed. This new procedure has been successfully applied to the catalytic asymmetric synthesis of the phytoalexines lacinilenes. The application of the *N*,*N*′-dioxide/metal catalyst system in the synthesis of other bioactive molecules will be explored.
