# Supplementary material for: Catalytic asymmetric hydroxylative dearomatization of 2-naphthols: synthesis of lacinilene derivatives
Source: Chem Sci. 2017 Jul 24;8(9):6645–9. doi: 10.1039/c7sc02809a (PMC5625263; doi:10.1039/c7sc02809a)

Electronic Supplementary Material (ESI) for Chemical Science.
This journal is © The Royal Society of Chemistry 2017

Supporting Information

CONTENTS:

1. General remarks……………………………………………………………….…2

2. General procedure for preparation 2-naphthols derivatives………………..….2

3. General procedure for the preparation 1y, 1z, 1ae and 1af……………….….10

4. General procedure for the synthesis of chiral *N,N'*-dioxides…………………17

5. General procedure for the preparation of the racemic products……………..17

6. Experimental procedure for the scale-up reaction……………………………18

7. ^{1}H NMR and HRMS experiments……………………………..……………..…18

8. Optimization of reaction conditions…………………………………………..…21

9. Full list of the substrate scope…………………………………………………...23

10. The analytical and spectral characterization data of products………………24

11. The X-ray data for 3ae……………………………………………………………54

12. References…………………………………………………………………….…...54

13. Copies of NMR spectra……………………………………… ……………...55

1. General remarks

^{1}H NMR spectra were recorded on commercial instruments (400 MHz). Chemical shifts were recorded in ppm relative to tetramethylsilane and with the solvent resonance as the internal standard ($CDCl_3$, δ = 7.262). Data were reported as follows: chemical shift, multiplicity (s = singlet, d = doublet, t = triplet, q = quartet, m = multiplet), coupling constants (Hz), integration. ^{13}C NMR data were collected on commercial instruments (100 MHz) with complete proton decoupling. Chemical shifts were reported in ppm from the tetramethylsilane with the solvent resonance as internal standard ($CDCl_3$, δ = 77.160). Enantiomeric excesses were determined by chiral HPLC analysis on Daicel Chiralcel IA, IC, IE, and Phenomenex Chiralcel Lux 5u Cellulose-1, Lux 5u Cellulose-3 at 23 °C with UV detector at 254 nm in comparison with the authentic racemates. Optical rotations were determined after flash column chromatography purification and reported as follows: $[\alpha]_D^T$ (c: g/100 mL, in CH_2Cl_2). HRMS were recorded on a commercial apparatus (ESI source). All the reactions were carried out under an atmosphere of nitrogen in over-dried apparatus. All the solvents were purified by usual methods before use. Chromatography: Qingdao Haiyang silica gel, HG/T2354-92, H CP.

2. General procedure for preparation 2-naphthols derivatives

The 2-naphthols derivatives **1a, 1c, 1r, 1s, 1x 1ab** were prepared by previously reported methods from 2-naphthols. [1]

Compound **1b** is prepared according to the literature.[2]

Compounds **1d-1q, 1t-1w** are prepared from compound **1b** according to the literature. [3]

1aa[3], **1ac**[3] and **1ad**[4] are known compounds.

1-methylnaphthalen-2-ol (1a)

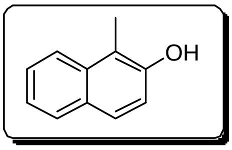

White solid; m.p. 78-80 °C, ^{1}H NMR (400 MHz, $CDCl_3$) δ = 7.94 (d, *J* = 9.2 Hz, 1H), 7.80 (d, *J* = 8.0 Hz, 1H), 7.64 (d, *J* = 8.4 Hz, 1H), 7.56 - 7.48 (m, 1H), 7.42 - 7.32 (m, 1H), 7.08 (d, *J* = 8.8 Hz,

1H), 5.00 (s, 1H), 2.56 (s, 3H); ^{13}C NMR (101 MHz, $CDCl_3$) δ = 150.9, 132.5, 130.5, 130.4, 129.6, 126.6, 125.2, 118.8, 117.1, 115.8, 10.62. HRMS (ESI-TOF) calcd for $C_{11}H_{10}ONa^+$ ([M+Na$^+$]) = 181.0624, Found 181.0631.

6-bromo-1-methylnaphthalen-2-ol (1b)

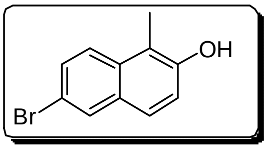

Purple solid; m.p. 94-96 °C, ^{1}H NMR (400 MHz, $CDCl_3$) δ = 7.91 (d, *J* = 2.0 Hz, 1H), 7.78 (d, *J* = 8.8 Hz, 1H), 7.60 - 7.49 (m, 2H), 7.07 (d, *J* = 8.8 Hz, 1H), 4.91 (s, 1H), 2.51 (s, 3H); ^{13}C NMR (101 MHz, $CDCl_3$) δ = 150.9, 132.5, 130.5, 130.4, 129.6, 126.6, 125.2, 118.78, 117.1, 115.8, 10.6. HRMS (ESI-TOF) calcd for $C_{11}H_9{}^{79}BrO\ Na^+$ ([M+Na$^+$]) = 258.9729, Found 258.9723. HRMS (ESI-TOF) calcd for $C_{11}H_9{}^{81}BrONa^+$ ([M+Na$^+$]) = 260.9709, Found 260.9716.

6-methoxy-1-methylnaphthalen-2-ol (1c)

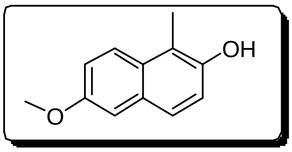

White solid; m.p. 116-119 °C, ^{1}H NMR (400 MHz, DMSO-d_6) δ = 9.23 (s, 1H), 7.77 (d, *J* = 8.8 Hz, 1H), 7.52 (d, *J* = 8.8 Hz, 1H), 7.24 - 7.18 (m, 1H), 7.16 - 7.07 (m, 2H), 3.82 (s, 3H), 2.39 (s, 3H); ^{13}C NMR (101 MHz, DMSO-d_6) δ = 154.6, 150.4, 129.0, 128.8, 125.4, 124.3, 118.4, 118.1, 114.9, 106.6, 55.0, 10.5. HRMS (ESI-TOF) calcd for $C_{12}H_{12}O_2Na^+$ ([M+Na$^+$]) = 211.0730, Found 221.0739.

1-methyl-6-phenylnaphthalen-2-ol (1d)

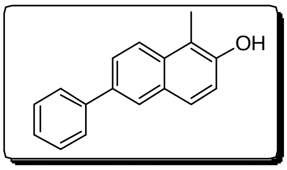

White solid; m.p. 149-152 °C, ^{1}H NMR (400 MHz, DMSO-d_6) δ = 9.59 (s, 1H), 8.09 (d, *J* = 1.2 Hz, 1H), 7.94 (d, *J* = 8.8 Hz, 1H), 7.84 - 7.75 (m, 3H), 7.72 (d, *J* = 8.8 Hz, 1H), 7.49 (t, *J* = 8.0 Hz, 2H), 7.37 (t, *J* = 7.6 Hz, 1H), 7.21 (d, *J* = 8.8 Hz, 1H), 2.45 (s, 3H); ^{13}C NMR (101 MHz, DMSO-d_6) δ = 152.4, 140.1, 133.7, 133.0, 128.9, 128.2, 127.2, 127.0, 126.5, 125.6, 124.9, 123.6, 118.4, 114.5, 10.4. HRMS (ESI-TOF) calcd for $C_{17}H_{14}ONa^+$ ([M+Na$^+$]) = 257.0937, Found 257.0937.

6-(4-chlorophenyl)-1-methylnaphthalen-2-ol (1e)

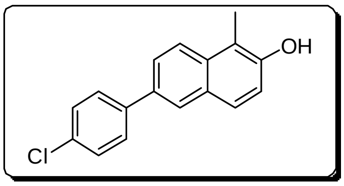

White solid; m.p. 164-167 °C, ^{1}H NMR (400 MHz, DMSO-d_6) δ = 9.64 (s, 1H), 8.10 (s, 1H), 7.94 (d, *J* = 8.8 Hz, 1H), 7.87 - 7.68 (m, 4H), 7.53 (d, *J* = 8.4 Hz, 2H), 7.21 (d, *J* = 8.8 Hz, 1H), 2.44 (s, 3H); ^{13}C NMR (101 MHz, DMSO-d_6) δ = 152.6, 138.9, 133.1, 132.3, 131.8, 128.8, 128.2, 128.2, 127.3, 125.7, 124.6, 123.7, 118.5, 114.6, 10.4. HRMS (ESI-TOF) calcd for $C_{17}H_{13}{}^{35}ClONa^+$ ([M+Na$^+$]) = 291.0547, Found 291.0543. HRMS (ESI-TOF) calcd for $C_{17}H_{13}{}^{37}ClONa^+$ ([M+ Na$^+$]) = 293.0518, Found 293.0518.

6-(2-methoxyphenyl)-1-methylnaphthalen-2-ol (1f)

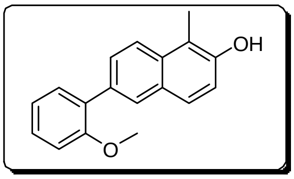

White solid; m.p. 92-94 °C, ^{1}H NMR (400 MHz, DMSO-d_6) δ = 9.53 (s, 1H), 7.94 - 7.78 (m, 2H), 7.65 (d, *J* = 8.8 Hz, 1H), 7.60 (dd, *J* = 8.8, 1.6 Hz, 1H), 7.44 - 7.30 (m, 2H), 7.15 (dd, *J* = 23.6, 8.8 Hz, 2H), 7.05 (t, *J* = 7.6 Hz, 1H), 3.77 (s, 3H), 2.45 (s, 3H); ^{13}C NMR (101 MHz, DMSO-d_6) δ = 156.3, 152.2, 132.6, 132.1, 130.5, 129.9, 128.6, 128.1, 127.8, 127.8, 127.0, 122.2, 120.8, 118.1, 114.4, 111.7, 55.4, 10.4. HRMS (ESI-TOF) calcd for $C_{18}H_{16}O_2Na^+$ ([M+Na$^+$]) = 287.1043, Found 287.1046.

6-(3-methoxyphenyl)-1-methylnaphthalen-2-ol (1g)

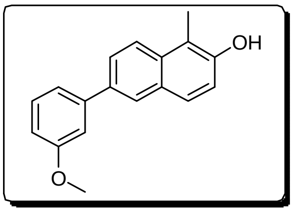

White solid; m.p. 114-118 °C, ^{1}H NMR (400 MHz, DMSO-d_6) δ = 9.59 (s, 1H), 8.10 (d, *J* = 2.0 Hz, 1H), 7.93 (d, *J* = 8.8 Hz, 1H), 7.78 (dd, *J* = 8.8, 2.0 Hz, 1H), 7.72 (d, *J* = 8.8 Hz, 1H), 7.43 - 7.28 (m, 3H), 7.21 (d, *J* = 8.8 Hz, 1H), 7.01 - 6.90 (m, 1H), 3.85 (s, 3H), 2.45 (s, 3H); ^{13}C NMR (101 MHz, DMSO-d_6) δ = 159.8, 152.4, 141.6, 133.6, 133.1, 129.9, 128.2, 127.2, 125.8, 125.0, 123.5, 118.9, 118.4, 114.5, 112.6, 112.0, 55.1, 10.4. HRMS (ESI-TOF) calcd for $C_{18}H_{16}O_2Na^+$ ([M+Na$^+$]) = 287.1043, Found 287.1045.

6-(4-methoxyphenyl)-1-methylnaphthalen-2-ol (1h)

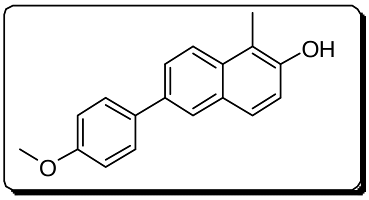

White solid; m.p. 180-186 °C, ^{1}H NMR (400 MHz, DMSO-d$_6$) δ = 9.54 (s, 1H), 8.01 (s, 1H), 7.90 (d, *J* = 8.8 Hz, 1H), 7.81 - 7.61 (m, 4H), 7.19 (d, *J* = 8.8 Hz, 1H), 7.04 (d, *J* = 8.4 Hz, 2H), 3.81 (s, 3H), 2.44 (s, 3H); ^{13}C NMR (101 MHz, DMSO-d$_6$) δ = 158.6, 152.1, 133.4, 132.6, 132.5, 128.3, 127.6, 127.0, 124.8, 123.5, 118.4, 114.5, 114.4, 55.1, 10.4. HRMS (ESI-TOF) calcd for $C_{18}H_{16}O_2Na^+$ ([M+Na$^+$]) = 287.1043, Found 287.1043.

6-(3,4-dimethoxyphenyl)-1-methylnaphthalen-2-ol (1i)

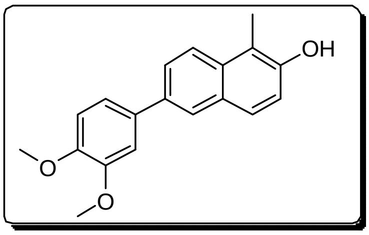

White solid; m.p. 184-186 °C, ^{1}H NMR (400 MHz, DMSO-d$_6$) δ = 9.53 (s, 1H), 8.04 (s, 1H), 7.90 (d, *J* = 8.8 Hz, 1H), 7.77 (d, *J* = 8.4 Hz, 1H), 7.69 (d, *J* = 8.8 Hz, 1H), 7.34 (s, 1H), 7.29 (d, *J* = 8.0 Hz, 1H), 7.19 (d, *J* = 8.4 Hz, 1H), 7.04 (d, *J* = 8.0 Hz, 1H), 3.88 (s, 3H), 3.80 (s, 3H), 2.44 (s, 3H); ^{13}C NMR (101 MHz, DMSO-d$_6$) δ = 152.2, 149.1, 148.2, 133.7, 132.9, 132.6, 128.3, 127.0, 125.0, 123.4, 118.7, 118.4, 114.5, 112.2, 110.3, 55.6, 10.4. HRMS (ESI-TOF) calcd for $C_{19}H_{18}O_3Na^+$ ([M+Na$^+$]) = 317.1148, Found 317.1149.

6-(benzo[d][1,3]dioxol-5-yl)-1-methylnaphthalen-2-ol (1j)

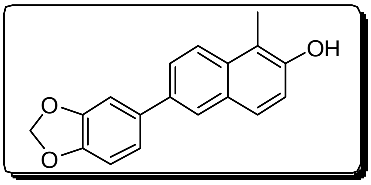

White solid; m.p. 127-129 °C, ^{1}H NMR (400 MHz, DMSO-d$_6$) δ = 9.53 (s, 1H), 8.01 (d, *J* = 1.6 Hz, 1H), 7.89 (d, *J* = 8.8 Hz, 1H), 7.72 (dd, *J* = 8.8, 2.0 Hz, 1H), 7.67 (d, *J* = 8.8 Hz, 1H), 7.36 (d, *J* = 1.6 Hz, 1H), 7.25 (dd, *J* = 8.4, 2.0 Hz, 1H), 7.18 (d, *J* = 8.8 Hz, 1H), 7.02 (d, *J* = 8.0 Hz, 1H), 6.08 (s, 2H), 2.43 (s, 3H); ^{13}C NMR (101 MHz, DMSO-d$_6$) δ = 152.2, 148.0, 146.5, 134.5, 133.5, 132.7, 128.2, 127.0, 125.2, 124.9, 123.4, 120.0, 118.4, 114.5, 108.7, 107.0, 101.0, 10.4. HRMS (ESI-TOF) calcd for $C_{18}H_{14}O_3Na^+$ ([M+Na$^+$]) = 301.0835, Found 301.0831.

5-methyl-[2,2'-binaphthalen]-6-ol (1k)

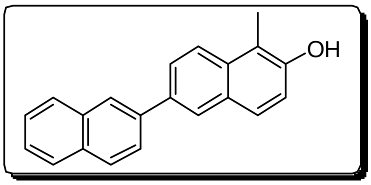

White solid; m.p. 170-174 °C, ^{1}H NMR (400 MHz, DMSO-d_6) δ = 9.63 (s, 1H), 8.33 (s, 1H), 8.26 (s, 1H), 8.07 - 7.88 (m, 6H), 7.77 (d, *J* = 8.4 Hz, 1H), 7.60 - 7.48 (m, 2H), 7.24 (dd, *J* = 8.8, 2.0 Hz, 1H), 2.47 (s, 3H); ^{13}C NMR (101 MHz, DMSO-d_6) δ = 152.6, 137.4, 133.4, 133.4, 133.1, 132.1, 128.4, 128.3, 128.1, 127.5, 127.3, 126.4, 126.0, 125.9, 125.1, 125.1, 124.8, 123.7, 118.5, 114.6, 10.5. HRMS (ESI-TOF) calcd for $C_{21}H_{16}ONa^+$ ([M+Na$^+$]) = 307.1093, Found 307.1104.

6-(furan-3-yl)-1-methylnaphthalen-2-ol (1l)

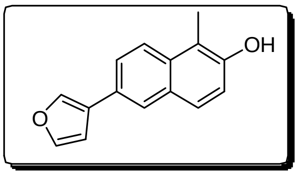

White solid; m.p. 163-165 °C, ^{1}H NMR (400 MHz, DMSO-d_6) δ = 9.53 (s, 1H), 8.24 (s, 1H), 7.99 (s, 1H), 7.85 (d, *J* = 8.8 Hz, 1H), 7.77 (d, *J* = 1.2 Hz, 1H), 7.71 (d, *J* = 8.8 Hz, 1H), 7.62 (d, *J* = 8.8 Hz, 1H), 7.19 (d, *J* = 8.8 Hz, 1H), 7.06 (s, 1H), 2.43 (s, 3H); ^{13}C NMR (101 MHz, DMSO-d_6) δ = 152.6, 144.7, 139.5, 133.2, 128.7, 127.1, 126.4, 126.2, 124.8, 124.6, 123.8, 118.9, 115.2, 109.2, 10.9. HRMS (ESI-TOF) calcd for $C_{15}H_{12}O_2K^+$ ([M+K$^+$]) = 263.0469, Found 263.0471.

(*E*)-1-methyl-6-styrylnaphthalen-2-ol (1m)

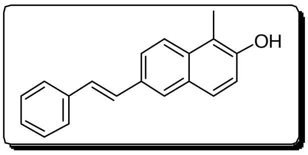

White solid; m.p. 146-150 °C, ^{1}H NMR (400 MHz, DMSO-d_6) δ = 9.58 (s, 1H), 7.92 - 7.77 (m, 3H), 7.70 - 7.59 (m, 3H), 7.43 - 7.23 (m, 5H), 7.18 (d, *J* = 8.8 Hz, 1H), 2.43 (s, 3H); ^{13}C NMR (101 MHz, DMSO-d_6) δ = 152.6, 137.3, 133.3, 131.0, 128.7, 128.6, 128.0, 127.4, 127.2, 127.0, 126.9, 126.3, 123.6, 123.3, 118.3, 115.0, 10.4. HRMS (ESI-TOF) calcd for $C_{19}H_{16}OK^+$ ([M+K$^+$]) = 299.0833, Found 299.0833.

(*E*)-1-methyl-6-(pent-1-en-1-yl)naphthalen-2-ol (1n)

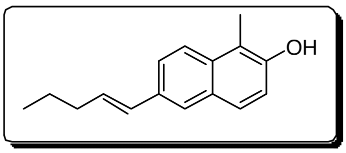

White solid; m.p. 106-110 °C, ^{1}H NMR (400 MHz, $CDCl_3$) δ = 7.84 (d, *J* = 8.8 Hz, 1H), 7.68 - 7.50 (m, 3H),

7.03 (d, J = 8.8 Hz, 1H), 6.52 (d, J = 15.6 Hz, 1H), 6.41 - 6.25 (m, 1H), 4.83 (s, 1H), 2.52 (s, 3H), 2.24 (q, J = 7.2 Hz, 3H), 1.62 - 1.48 (m, 2H), 1.06 - 0.91 (m, 3H); ^{13}C NMR (101 MHz, $CDCl_3$) δ = 150.4, 133.1, 132.9, 130.6, 129.9, 129.5, 127.4, 126.0, 124.2, 123.5, 117.8, 115.5, 35.4, 22.8, 13.9, 10.6. HRMS (ESI-TOF) calcd for $C_{16}H_{18}ONa^+$ ([M+Na$^+$]) = 227.1436, Found 227.1435.

6-(cyclopent-1-en-1-yl)-1-methylnaphthalen-2-ol (1o)

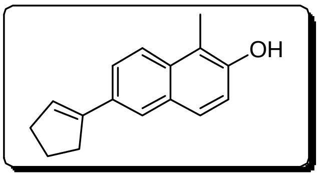

White solid; m.p. 158-161 °C, ^{1}H NMR (400 MHz, $CDCl_3$) δ = 7.84 (d, J = 8.8 Hz, 1H), 7.76 - 7.68 (m, 1H), 7.66 (s, 1H), 7.59 (d, J = 8.8 Hz, 1H), 7.04 (d, J = 8.4 Hz, 1H), 6.29 (s, 1H), 4.85 (s, 1H), 2.90 - 2.75 (m, 2H), 2.64 - 2.55 (m, 2H), 2.53 (s, 3H), 2.15 - 2.00 (m, 2H); ^{13}C NMR (101 MHz, $CDCl_3$) δ = 150.5, 142.4, 133.0, 131.7, 129.4, 127.6, 126.0, 124.9, 124.8, 123.2, 117.8, 115.5, 33.6, 33.3, 23.5, 10.6. HRMS (ESI-TOF) calcd for $C_{16}H_{16}ONa^+$ ([M+Na$^+$]) = 247.1093, Found 247.1098.

1-methyl-6-(phenylethynyl)naphthalen-2-ol (1p)

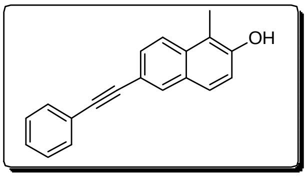

Brown solid; m.p. 110-112 °C, ^{1}H NMR (400 MHz, $CDCl_3$) δ = 7.99 (s, 1H), 7.89 (d, J = 8.8 Hz, 1H), 7.67 - 7.50 (m, 4H), 7.41 - 7.30 (m, 3H), 7.09 (d, J = 8.8 Hz, 1H), 5.05 (s, 1H), 2.53 (s, 3H); ^{13}C NMR (101 MHz, $CDCl_3$) δ = 151.5, 133.5, 132.2, 131.7, 129.0, 128.9, 128.5, 128.3, 127.5, 123.6, 123.5, 118.4, 117.8, 115.6, 90.0, 89.3, 10.6. HRMS (ESI-TOF) calcd for $C_{19}H_{14}OK^+$ ([M+K$^+$]) = 297.0682, Found 297.0681.

6-(3-hydroxyprop-1-yn-1-yl)-1-methylnaphthalen-2-ol (1q)

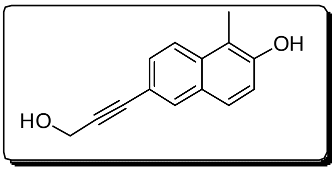

Light yellow solid; m.p. 130-132 °C, ^{1}H NMR (400 MHz, DMSO-d_6) δ = 9.70 (s, 1H), 7.89 (d, J = 1.6 Hz, 1H), 7.84 (d, J = 8.8 Hz, 1H), 7.63 (d, J = 8.8 Hz, 1H), 7.42 (dd, J = 8.4, 2.0 Hz, 1H), 7.20 (d, J = 9.2 Hz, 1H), 5.33 (t, J = 6.0 Hz, 1H), 4.34 (d, J = 6.0 Hz, 2H), 2.40 (s, 3H); ^{13}C NMR (101 MHz, DMSO-d_6) δ = 153.2, 133.1, 131.4,

128.1, 127.5, 126.7, 123.3, 118.7, 115.8, 114.9, 89.2, 84.2, 49.5, 10.3. HRMS (ESI-TOF) calcd for $C_{14}H_{12}O_2Na^+$ ([M+Na$^+$]) = 235.0730, Found 235.0740.

1,6-dimethylnaphthalen-2-ol (1r)

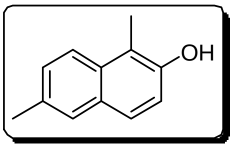

White solid; m.p. 53-56 °C, ^{1}H NMR (400 MHz, $CDCl_3$) δ = 7.82 (d, *J* = 8.8 Hz, 1H), 7.58 - 7.48 (m, 2H), 7.37 - 7.31 (m, 1H), 7.03 (d, *J* = 8.8 Hz, 1H), 4.84 (s, 1H), 2.52 (s, 3H), 2.48 (s, 3H); ^{13}C NMR (101 MHz, $CDCl_3$) δ = 145.0, 132.6, 132.1, 129.5, 128.7, 127.6, 126.8, 123.2, 117.7, 115.2, 21.4, 10.6. HRMS (ESI-TOF) calcd for $C_{12}H_{12}ONa^+$ ([M+Na$^+$]) = 195.0780, Found 195.0785.

6-ethyl-1-methylnaphthalen-2-ol (1s)

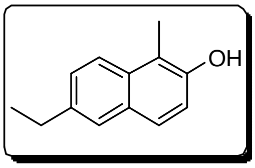

White solid; m.p. 68-70 °C, ^{1}H NMR (400 MHz, $CDCl_3$) δ = 7.81 (d, *J* = 8.8 Hz, 1H), 7.61 - 7.47 (m, 2H), 7.34 (d, *J* = 8.8 Hz, 1H), 6.98 (d, *J* = 8.8 Hz, 1H), 5.11 (s, 1H), 2.76 (q, *J* = 7.6 Hz, 2H), 2.48 (s, 3H), 1.29 (t, *J* = 7.6 Hz, 3H); ^{13}C NMR (101 MHz, $CDCl_3$) δ = 149.9, 139.0, 132.3, 129.5, 127.6, 126.9, 126.3, 123.3, 117.7, 115.4, 28.75, 15.7, 10.6. HRMS (ESI-TOF) calcd for $C_{13}H_{15}O^+$ ([M+H$^+$]) = 187.1117, Found 187.1117.

1-methyl-6-pentylnaphthalen-2-ol (1t)

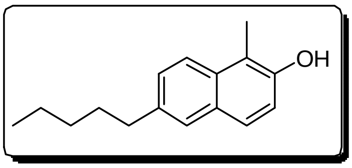

White solid; m.p. 75-78 °C, ^{1}H NMR (400 MHz, $CDCl_3$) δ = 7.85 (d, *J* = 8.8 Hz, 1H), 7.63 - 7.52 (m, 2H), 7.37 (d, *J* = 8.0 Hz, 1H), 7.04 (d, *J* = 8.8 Hz, 1H), 4.88 - 4.81 (m, 1H), 2.75 (t, *J* = 7.6 Hz, 2H), 2.54 (s, 3H), 1.76 - 1.65 (m, 2H), 1.46 - 1.29 (m, 4H), 0.96 - 0.86 (m, 3H); ^{13}C NMR (101 MHz, $CDCl_3$) δ = 150.0, 137.7, 132.3, 129.5, 128.0, 127.1, 126.9, 123.2, 117.6, 115.2, 35.8, 31.7, 31.3, 22.7, 14.2, 10.6. HRMS (ESI-TOF) calcd for $C_{16}H_{20}ONa^+$ ([M+Na$^+$]) = 251.1406, Found 251.1403.

6-isobutyl-1-methylnaphthalen-2-ol (1u)

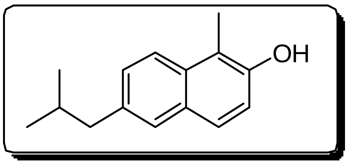

White solid; m.p. 102-104 °C, ^{1}H NMR (400 MHz, $CDCl_3$) δ = 7.84 (d, *J* = 8.8 Hz, 1H), 7.57 (d, *J* = 8.8 Hz, 1H), 7.52 (d, *J* = 0.8 Hz, 1H), 7.33 (dd, *J* = 8.8, 2.0 Hz, 1H), 7.04 (d, *J* = 8.8 Hz, 1H), 4.82 (s, 1H), 2.62 (d, *J* = 7.2 Hz, 2H), 2.53 (s, 3H), 2.03 - 1.90 (m, 1H), 0.95 (d, *J* = 6.9 Hz, 6H); ^{13}C NMR (101 MHz, $CDCl_3$) δ = 150.0, 136.5, 132.3, 129.4, 128.5, 128.0, 127.0, 123.0, 117.6, 115.2, 45.4, 30.4, 22.6, 10.6. HRMS (ESI-TOF) calcd for $C_{15}H_{18}ONa^+$ ([M+Na$^+$]) = 237.1250, Found 237.1254.

6-cyclopentyl-1-methylnaphthalen-2-ol (1v)

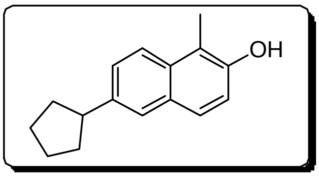

White solid; m.p. 80-83 °C, ^{1}H NMR (400 MHz, $CDCl_3$) δ = 7.86 (d, *J* = 8.8 Hz, 1H), 7.62 - 7.53 (m, 2H), 7.43 (dd, *J* = 8.8, 2.0 Hz, 1H), 7.04 (d, *J* = 8.8 Hz, 1H), 4.80 (d, *J* = 2.0 Hz, 1H), 3.20 - 3.07 (m, 1H), 2.53 (s, 3H), 2.23 - 2.08 (m, 2H), 1.91 - 1.63 (m, 6H); ^{13}C NMR (101 MHz, $CDCl_3$) δ = 150.0, 141.2, 132.4, 129.4, 127.0, 126.8, 125.6, 123.2, 117.7, 115.2, 45.8, 34.7, 25.7, 10.6. HRMS (ESI-TOF) calcd for $C_{16}H_{19}O^+$ ([M+H$^+$]) = 227.1430, Found 227.1444.

1-methyl-6-phenethylnaphthalen-2-ol (1w)

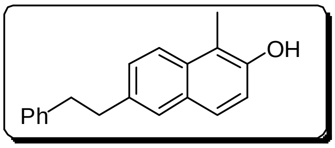

White solid; m.p. 88-92 °C, ^{1}H NMR (400 MHz, $CDCl_3$) δ = 7.85 (d, *J* = 8.8 Hz, 1H), 7.62 - 7.52 (m, 2H), 7.38 - 7.28 (m, 3H), 7.24 - 7.16 (m, 3H), 7.05 (d, *J* = 8.8 Hz, 1H), 4.91 (s, 1H), 3.11 - 2.97 (m, 4H), 2.54 (s, 3H); ^{13}C NMR (101 MHz, $CDCl_3$) δ = 150.2, 142.0, 136.5, 132.5, 129.4, 128.6, 128.5, 127.9, 127.2, 127.0, 126.0, 123.3, 117.7, 115.3, 38.0, 37.8, 10.6. HRMS (ESI-TOF) calcd for $C_{19}H_{18}ONa^+$ ([M+Na$^+$]) = 285.1250, Found 285.1244.

7-methoxy-1-methylnaphthalen-2-ol (1x)

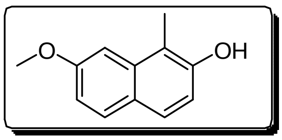

White solid; m.p. 106-108 °C, ^1H NMR (400 MHz, $CDCl_3$) δ = 7.68 (d, *J* = 8.8 Hz, 1H), 7.55 (d, *J* = 8.8 Hz, 1H), 7.17 (d, *J* = 2.4 Hz, 1H), 7.03 (dd, *J* = 8.8, 2.4 Hz, 1H), 6.92 (d, *J* = 8.8 Hz, 1H), 5.00 (s, 1H), 3.96 (s, 3H), 2.50 (s, 3H); ^{13}C NMR (101 MHz, $CDCl_3$) δ = 158.3, 151.2, 135.3, 130.1, 127.2, 124.7, 115.4, 115.2, 114.4, 102.4, 55.4, 10.8. HRMS (ESI-TOF) calcd for $C_{12}H_{12}O_2Na^+$ ([M+Na^+]) = 221.0730, Found 221.0738.

7-methoxy-1,6-dimethylnaphthalen-2-ol (1y)

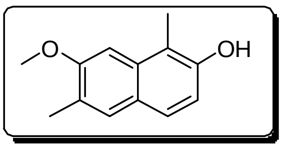

White solid; m.p. 169-170 °C, ^1H NMR (400 MHz, DMSO-d_6) δ = 9.29 (s, 1H), 7.49 (s, 1H), 7.43 (d, *J* = 8.4 Hz, 1H), 7.06 (s, 1H), 6.96 (d, *J* = 8.8 Hz, 1H), 3.91 (s, 3H), 2.38 (s, 3H), 2.25 (s, 3H); ^{13}C NMR (101 MHz, DMSO-d_6) δ = 156.5, 151.9, 133.7, 129.0, 125.5, 123.2, 123.0, 115.2, 113.5, 100.3, 55.0, 16.2, 10.6. HRMS (ESI-TOF) calcd for $C_{13}H_{14}O_2^+$ ([M+H^+]) = 203.1072, Found 203.1275.

1-ethylnaphthalen-2-ol (1ab)

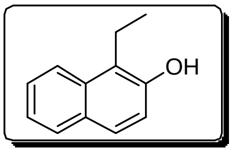

White solid; m.p. 92-95 °C, ^1H NMR (400 MHz, $CDCl_3$) δ = 7.96 (d, *J* = 8.4 Hz, 1H), 7.79 (d, *J* = 8.0 Hz, 1H), 7.64 (d, *J* = 8.8 Hz, 1H), 7.53 - 7.47 (m, 1H), 7.38 - 7.32 (m, 1H), 7.07 (d, *J* = 8.8 Hz, 1H), 4.92 (s, 1H), 3.08 (q, *J* = 7.6 Hz, 2H), 1.30 (t, *J* = 7.6 Hz, 3H); ^{13}C NMR (101 MHz, $CDCl_3$) δ = 150.1, 133.0, 129.6, 128.8, 127.7, 126.5, 123.2, 123.0, 121.8, 117.8, 18.4, 14.3. HRMS (ESI-TOF) calcd for $C_{12}H_{12}ONa^+$ ([M+Na^+]) = 195.0780, Found 195.0780.

3. General procedure for the preparation 1y, 1z, 1ae and 1af

3.1. General procedure for the preparation **1z**, and **1ae**

The compounds **5** were prepared by previously reported methods from 6-((tert-butyldimethylsilyl)oxy)naphthalen-2-ol.[1]

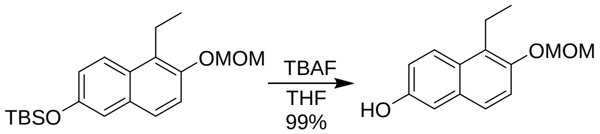

5 **6**

5-ethyl-6-(methoxymethoxy)naphthalen-2-ol (6): TBAF (12 mmol) was added to solution of **5** (10 mmol) in tetrahydrofuran (50.0 mL) at 0 °C and stirred for 10 min. After the reaction completion, the reaction was quenched by saturated NH_4Cl, the organic layer was extracted with ethyl acetate, washed with brine, dried over Na_2SO_4, filtered and then concentrated. The residue was purified by column chromatography (ethyl acetate/petroleum ether = 1/10, v/v) to afford the product **6**.

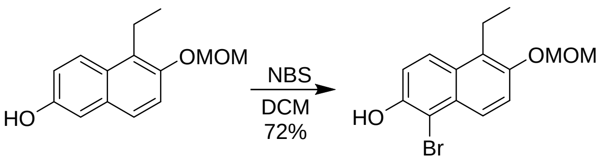

6 **7**

1-bromo-5-ethyl-6-(methoxymethoxy)naphthalen-2-ol (7): NBS (3.9 mmol) was added to solution of **6** (3.9 mmol) in CH_2Cl_2 (20.0 mL) and stirred for 5 min. After the reaction completion, the reaction was quenched by H_2O The organic layer was extracted with CH_2Cl_2, dried over Na_2SO_4, filtered and then concentrated. The residue was purified by column chromatography (ethyl acetate/petroleum ether = 1/20, v/v) to afford the product **7**.

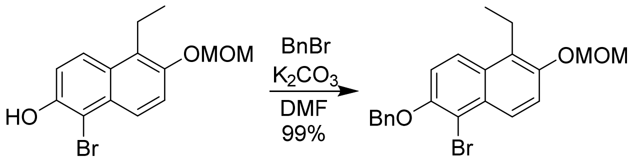

7 **8**

2-(benzyloxy)-1-bromo-5-ethyl-6-(methoxymethoxy)naphthalene (8): To a solution of **7** (8.0 mmol) in DMF (10 mL) were added K_2CO_3 (12 mmol), BnBr (12 mmol) at room temperature. The mixture was continuously stirred for 3 h. After reaction completion, the resulting solution was quenched by water, extracted with ethyl acetate, dried over Na_2SO_4, filtered and then concentrated. The residue was

purified by column chromatography (ethyl acetate/petroleum ether = 1/30, v/v) to afford the product **8**.

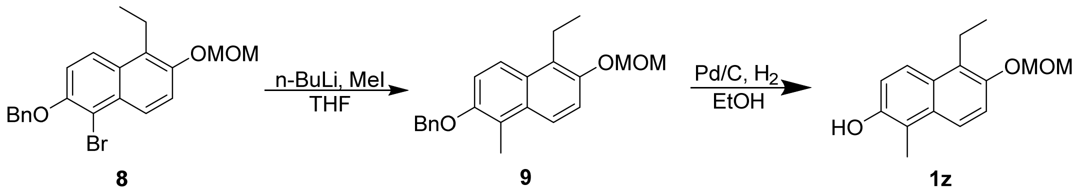

2-(benzyloxy)-5-ethyl-6-(methoxymethoxy)-1-methylnaphthalene (1z): To a solution of **8** (4.0 mmol) in THF (20.0 mL) under N_2 atmosphere was added n-BuLi (4.4 mmol), at -78 ^oC. The mixture was continuously stirred at the same temperature for 1 h, then MeI (10 mmol) was added. The mixture was warming up to room temperature and continuously stirred for 1 h. Then, the resulting solution was quenched by saturated NH_4Cl solution and extracted with ethyl acetate. The combined organic layer was washed with brine, dried over Na_2SO_4, filtered and then concentrated. *This material was directly used in the next stage without further purification.* 10 % Pd/C (100 mg) and **9** was added EtOH (6 mL) under H_2 atmosphere. The mixture was stirred at room temperature for 7 h, filtered and then concentrated. The residue was purified by column chromatography (ethyl acetate/petroleum ether = 1/10, v/v) to afford the **1z** (590 mg, 60% yield for two steps).

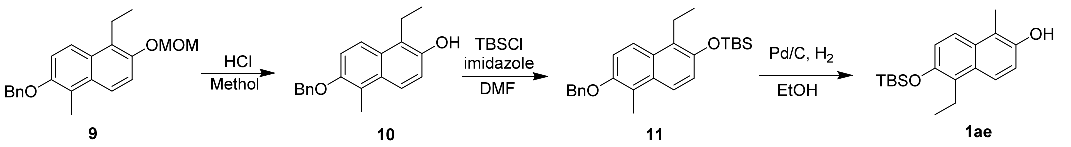

6-((tert-butyldimethylsilyl)oxy)-5-ethyl-1-methylnaphthalen-2-ol (1ae): To a solution of **9** (3 mmol) in CH_3OH (6 mL) was warmed to 50 ^oC and conc. HCl aq. (35%, 4 drops) was added. After stirring for 1 h, 10 mL of water was added and the aqueous layer was extracted with EtOAc. The combined organic layers were washed with brine, dried over Na_2SO_4, and concentrated under reduced pressure to give a crude product of **10**. *This material was directly used in the next stage without further purification.* To a solution of **10** in DMF (10 mL) was added imidazole (4.5 mmol)

and TBSCl (4.5 mmol). The mixture was warming up to 60 °C and continuously stirred for 1 h. Then, the resulting solution was extracted with ethyl acetate. The combined organic layer was washed with brine, dried over Na_2SO_4, filtered and then concentrated. *This material was directly used in the next stage without further purification.* 10 % Pd/C (100 mg) and **11** was added EtOH (6 mL) under H_2 atmosphere. The mixture was stirred at room temperature for 7 h, filtered and then concentrated. The residue was purified by column chromatography (ethyl acetate/petroleum ether = 1/20, v/v) to afford the **1ae** (626 mg, 66% yield for three steps).

5-ethyl-6-(methoxymethoxy)naphthalen-2-ol (6)

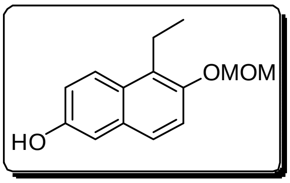

White solid; ^{1}H NMR (400 MHz, $CDCl_3$) δ = 7.89 (d, *J* = 8.8 Hz, 1H), 7.48 - 7.40 (m, 1H), 7.37 - 7.29 (m, 1H), 7.18 - 7.03 (m, 2H), 5.89 (s, 1H), 5.30 (s, 2H), 3.59 (s, 3H), 3.12 (q, *J* = 7.2 Hz, 2H), 1.28 (t, *J* = 7.6 Hz, 3H); ^{13}C NMR (101 MHz, $CDCl_3$) δ = 152.0, 150.1, 131.3, 128.0, 127.6, 125.8, 125.5, 118.2, 117.6, 110.3, 95.7, 56.3, 18.7, 14.9.

1-bromo-5-ethyl-6-(methoxymethoxy)naphthalen-2-ol (7)

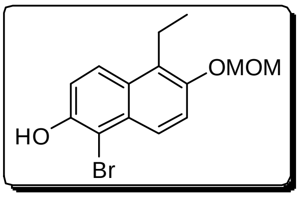

White solid; ^{1}H NMR (400 MHz, $CDCl_3$) δ = 7.88 (d, *J* = 9.2 Hz, 2H), 7.46 (d, *J* = 9.2 Hz, 1H), 7.25 (d, *J* = 8.8 Hz, 1H), 5.87 (s, 1H), 5.27 (s, 2H), 3.53 (s, 3H), 3.09 (q, *J* = 7.6 Hz, 2H), 1.23 (t, *J* = 7.6 Hz, 3H); ^{13}C NMR (101 MHz, $CDCl_3$) δ = 150.8, 149.1, 128.9, 128.7, 127.7, 124.8, 124.8, 118.2, 117.4, 106.9, 95.4, 56.3, 18.9, 14.9.

2-(benzyloxy)-1-bromo-5-ethyl-6-(methoxymethoxy)naphthalene (8)

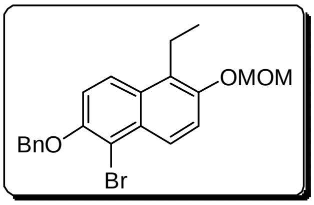

White solid; ^{1}H NMR (400 MHz, $CDCl_3$) δ = 8.13 (d, *J* = 9.6 Hz, 1H), 7.91 (d, *J* = 9.2 Hz, 1H), 7.56 - 7.50 (m, 2H), 7.48 (d, *J* = 9.6 Hz, 1H), 7.42 - 7.37 (m, 2H), 7.35 - 7.31

(m, 1H), 7.28 - 7.24 (m, 1H), 5.28 (s, 4H), 3.53 (s, 3H), 3.09 (q, J = 7.6 Hz, 2H), 1.23 (t, J = 7.6 Hz, 3H); ^{13}C NMR (101 MHz, $CDCl_3$) δ = 151.5, 151.0, 137.0, 129.7, 129.5, 128.7, 128.1, 127.4, 127.1, 125.9, 124.3, 118.3, 116.2, 111.0, 95.4, 72.1, 56.3, 18.8, 14.9.

5-ethyl-6-(methoxymethoxy)-1-methylnaphthalen-2-ol (1z)

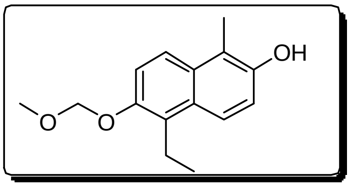

White solid; m.p. 110-112 °C, ^{1}H NMR (400 MHz, $CDCl_3$) δ = 7.78 (t, J = 8.8 Hz, 2H), 7.41 (d, J = 9.6 Hz, 1H), 7.08 (d, J = 9.2 Hz, 1H), 5.28 (s, 2H), 4.82 (s, 1H), 3.55 (s, 3H), 3.11 (q, J = 7.6 Hz, 2H), 2.52 (s, 3H), 1.26 (t, J = 7.6 Hz, 3H); ^{13}C NMR (101 MHz, $CDCl_3$) δ = 150.0, 149.1, 130.5, 128.4, 127.8, 122.8, 122.5, 118.1, 117.1, 116.0, 95.6, 56.2, 18.8, 15.0, 10.9. HRMS (ESI-TOF) calcd for $C_{15}H_{18}O_3Na^+$ ([M+Na$^+$]) = 269.1154, Found 269.1149.

6-((tert-butyldimethylsilyl)oxy)-5-ethyl-1-methylnaphthalen-2-ol (1ae)

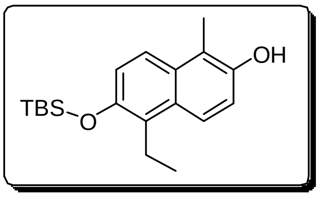

White solid; m.p. 70-74 °C, ^{1}H NMR (400 MHz, $CDCl_3$) δ = 7.78 (d, J = 8.8 Hz, 1H), 7.71 (d, J = 8.8 Hz, 1H), 7.14 (d, J = 9.2 Hz, 1H), 7.08 (d, J = 9.2 Hz, 1H), 4.96 - 4.85 (m, 1H), 3.09 (q, J = 7.2 Hz, 2H), 2.54 (s, 3H), 1.26 (t, J = 7.6 Hz, 3H), 1.10 (s, 9H), 0.30 (s, 6H); ^{13}C NMR (101 MHz, $CDCl_3$) δ = 148.7, 148.3, 129.9, 128.9, 128.1, 122.8, 122.1, 120.8, 117.8, 116.0, 26.0, 19.0, 18.5, 14.7, 10.9, -3.8. HRMS (ESI-TOF) calcd for $C_{19}H_{28}O_2Si$ Na^+ ([M+Na$^+$]) = 339.1756, Found 339.1747.

3.2. General procedure for the preparation **1y** and **1af**

6-methoxy-4,7-dimethyl-1,2-dihydronaphthalene (12) was prepared by previously reported. [5]

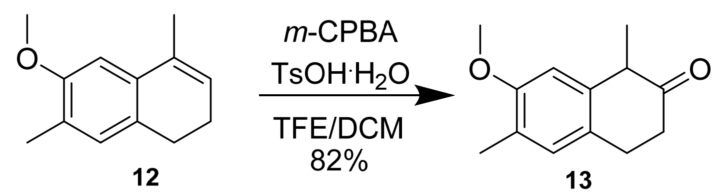

7-methoxy-1,6-dimethyl-3,4-dihydronaphthalen-2(1H)-one (13): To a stirred solution of **12** (42 mmol) in TFE/CH_2Cl_2 (1:4, 250 mL) at 0 °C was added *m*CPBA (52.5 mmol), and TsOH.H_2O (52.5 mmol). After 20 min, the reaction was quenched with $NaHCO_3$. Purification by column chromatography (ethyl acetate/petroleum ether = 1/10, v/v) to afford the **13** as colorless oil.

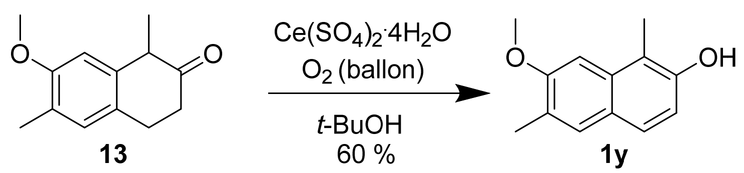

7-methoxy-1,6-dimethylnaphthalen-2-ol (1y): To a solution of **13** (20 mmol) and $Ce(SO_4)_2 \cdot 4H_2O$ (20 mmol) in *t*-BuOH (30 mL) under O_2 atmosphere was heated to 80 °C . After stirring for 10 h, the resulting solution was filtered and then concentrated. Purification by column chromatography (ethyl acetate/petroleum ether = 1/10, v/v) to afford the **1y** as white solid.

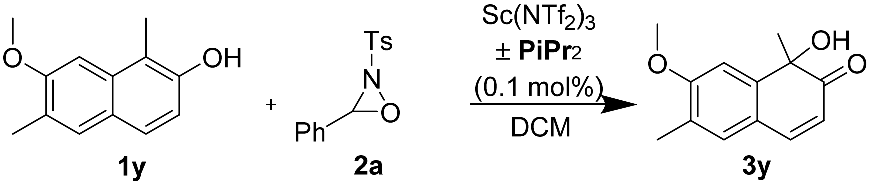

hydroxy-7-methoxy-1,6-dimethylnaphthalen-2(1H)-one (3y): To an oven-dried flask under nitrogen atmosphere was added $Sc(NTf_2)_3$ (0.1 mol%), *rac*-**L-PiPr₂** (0.1 mol%), compound **1y** (38.0 mmol) and CH_2Cl_2 (80.0 mL). After stirring at 35 °C for 30 min. Then oxaziridine **2a** (45.0 mmol) was added. The reaction mixture was stirred for 15 h and directly purified by flash column chromatography (DCM / ethyl acetate =

1:0 - 10:1) to afford the desired product **3y** (7.4 g, 90% yield).

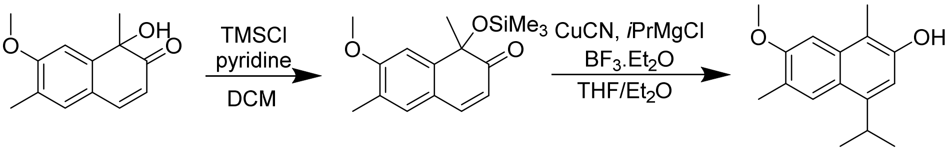

3y **14** **1af**

4-isopropyl-7-methoxy-1,6-dimethylnaphthalen-2-ol (1af): A solution of the **3y** (5.0 mmol) in dry dichloromethane (20 mL) and pyridine (5.5 mmol) was treated with TMSCl (6.3 mmol), and the mixture was stirred for 18 h at 20 °C. The resulting solution was extracted with DCM. The combined organic layer was washed with brine, dried over Na_2SO_4, filtered and then concentrated. *This material was directly used in the next stage without further purification.* A suspension of copper(I) cyanide (20 mmol) in THF (15 mL) and diethyl ether (15 mL) was treated under N_2 at -78 °C with a solution of the isopropyl magnesium chloride (40 mmol). Then, boron trifluoride etherate (10 mL) and the silyl ethers **14** (5.0 mmol) were added. The mixture was allowed to warm to 20 °C within 8 h and then hydrolyzed by addition of saturated aqueous ammonium chloride (200 mL). The organic phase was separated, the aqueous phase extracted twice with diethyl ether, dried ($MgSO_4$), and then concentrated. Purification by column chromatography (ethyl acetate/petroleum ether = 1/10, v/v) to afford the **1af** (549 mg, 45% yield for two steps) as white solid.

7-methoxy-1,6-dimethyl-3,4-dihydronaphthalen-2(1H)-one (13)

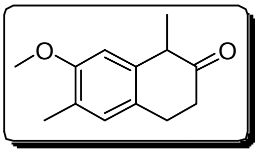

Colorless oil; ^{1}H NMR (400 MHz, $CDCl_3$) δ = 6.87 (s, 1H), 6.55 (s, 1H), 3.72 (s, 3H), 3.36 (q, J = 7.2 Hz, 1H), 2.91 - 2.82 (m, 2H), 2.53 - 2.33 (m, 2H), 2.10 (s, 3H), 1.36 (d, J = 7.2 Hz, 3H); ^{13}C NMR (101 MHz, $CDCl_3$) δ = 212.6, 156.8, 136.4, 129.8, 128.0, 124.8, 108.1, 55.4, 47.4, 37.6, 27.1, 15.7, 15.1.

4-isopropyl-7-methoxy-1,6-dimethylnaphthalen-2-ol (1af)

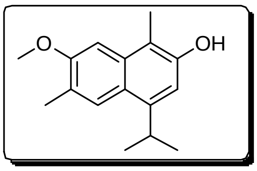

White solid; m.p. 162-164 °C, ^{1}H NMR (400 MHz, DMSO-d_6) δ = 7.77 (s, 1H), 7.09 (s, 1H), 6.83 (s, 1H), 4.82 (s, 1H), 3.95 (s, 3H), 3.70 - 3.58 (m, 1H), 2.46 (s, 3H), 2.37 (s, 3H), 1.34 (d, J = 6.8 Hz, 6H); ^{13}C NMR (101 MHz, DMSO-d_6) δ = 156.9, 150.1, 143.6, 134.5, 125.0, 124.7, 122.1, 111.8, 111.3, 101.3, 55.2, 28.3, 23.6, 17.0, 10.7. HRMS (ESI-TOF) calcd for $C_{16}H_{21}O_2^+$ ([M+H$^+$]) = 245.1536, Found 245.1538.

4. General procedure for the synthesis of chiral *N,N'*-dioxides

The *N,N'*-dioxide ligands were prepared by the similar procedure in the literatures. [6]

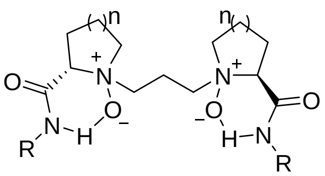

L-PrPr$_2$: R = 2,6-^{i}Pr$_2$C$_6$H$_3$, n = 1
L-PiPr$_2$: R = 2,6-^{i}Pr$_2$C$_6$H$_3$, n = 2
L-PiMe$_2$: R = 2,6-Me$_2$C$_6$H$_3$, n = 2
L-PiPr$_3$: R = 2,4,6-^{i}Pr$_3$C$_6$H$_2$, n = 2
L-PiPh: R = C$_6$H$_5$, n = 2
L-PiAd: R = 1-adamantane, n = 2

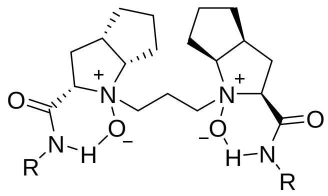

L-RaPr$_2$: R = 2,6-^{i}Pr$_2$C$_6$H$_3$

5. General procedure for the preparation of the racemic products

3b, **3d**, **3f**, **3r**, **3t**, **3ab** are Prepared by *Method A*, others are Prepared by *Method B.*

Method A

To a solution of Sc(OTf)$_3$ (10 mol%), 2-naphthols derivatives (0.1 mmol) in dry CH_2Cl_2 (1.0 mL), were added oxaziridine (0.2 mmol) and Et_3N (0.1 mmol). After stirring at room temperature for 3 h. The reaction mixture was then directly purified by flash column chromatography (DCM / ethyl acetate = 1:0 - 10:1) to afford the desired product.

Method B

To an oven-dried flask under nitrogen atmosphere was added Sc(NTf$_2$)$_3$ (5 mol%), *rac*-**L-PiPr$_2$** (5 mol%), 2-naphthols derivatives (0.1 mmol) and CH_2Cl_2 (1.0 mL). After stirring at 35 ℃ for 30 min, the reaction mixture was cool to 0 ℃, oxaziridine **2a** (0.2 mmol) was then added. The reaction mixture was stirred at 0 ℃ for 3 h and directly purified by flash column chromatography (DCM / ethyl acetate = 1:0 - 10:1)

to afford the desired product.

6. Experimental procedure for the scale-up reaction

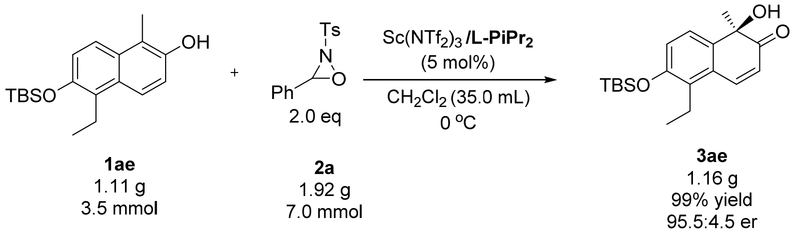

General Procedure: To an oven-dried flask under nitrogen atmosphere was added $Sc(NTf_2)_3$ (5 mol%), **L-PiPr₂** (5 mol%), compound **1ae** (1.11 g, 3.5 mmol) and CH_2Cl_2 (35.0 mL). After stirring at 35 ℃ for 30 min, the reaction mixture was cool to 0 ℃ and stirring for another 5 min. Then oxaziridine **2a** (1.92 g, 7.0 mmol) was added. The reaction mixture was stirred at 0 ℃ for 3 h and directly purified by flash column chromatography (DCM / ethyl acetate = 1:0 - 10:1) to afford the desired product (1.16 g, 99% yield, 95.5:4.5 er).

7. ^{1}H NMR and HRMS experiments

7.1 ^{1}H NMR spectra of **1a** in diverse conditions:

1. Red: **1a** in $CDCl_3$; Blue: $Sc(NTf_2)_3$/**1a** in $CDCl_3$

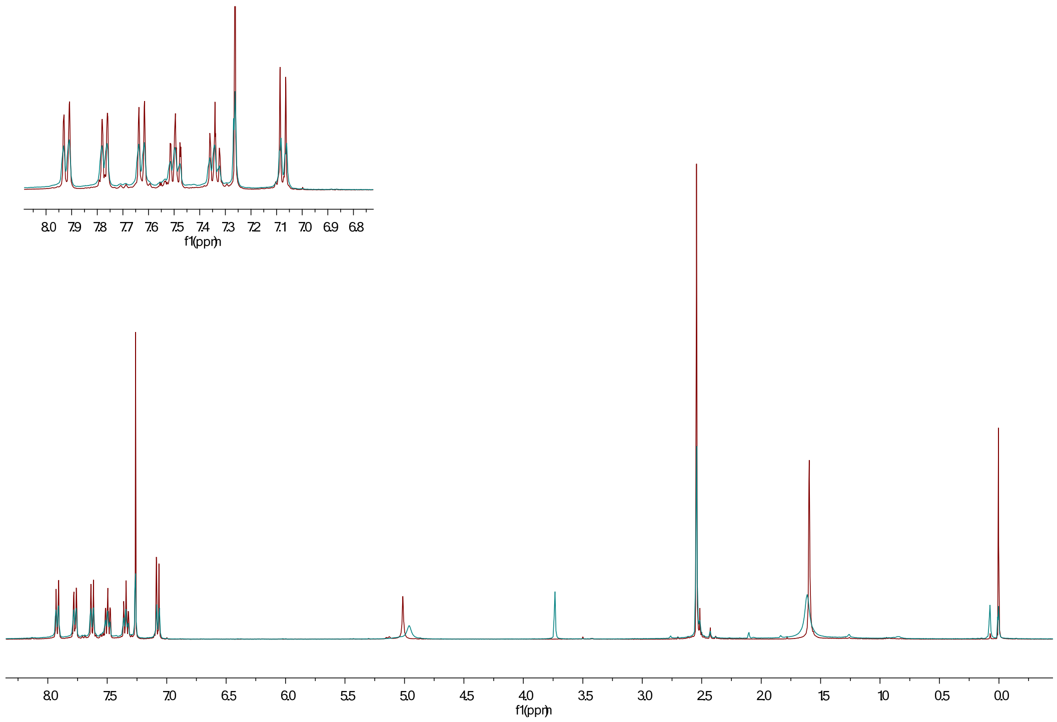

2. Red: **1a** in $CDCl_3$; Blue: **L-PiPr2**/$Sc(NTf_2)_3$/**1a** in $CDCl_3$

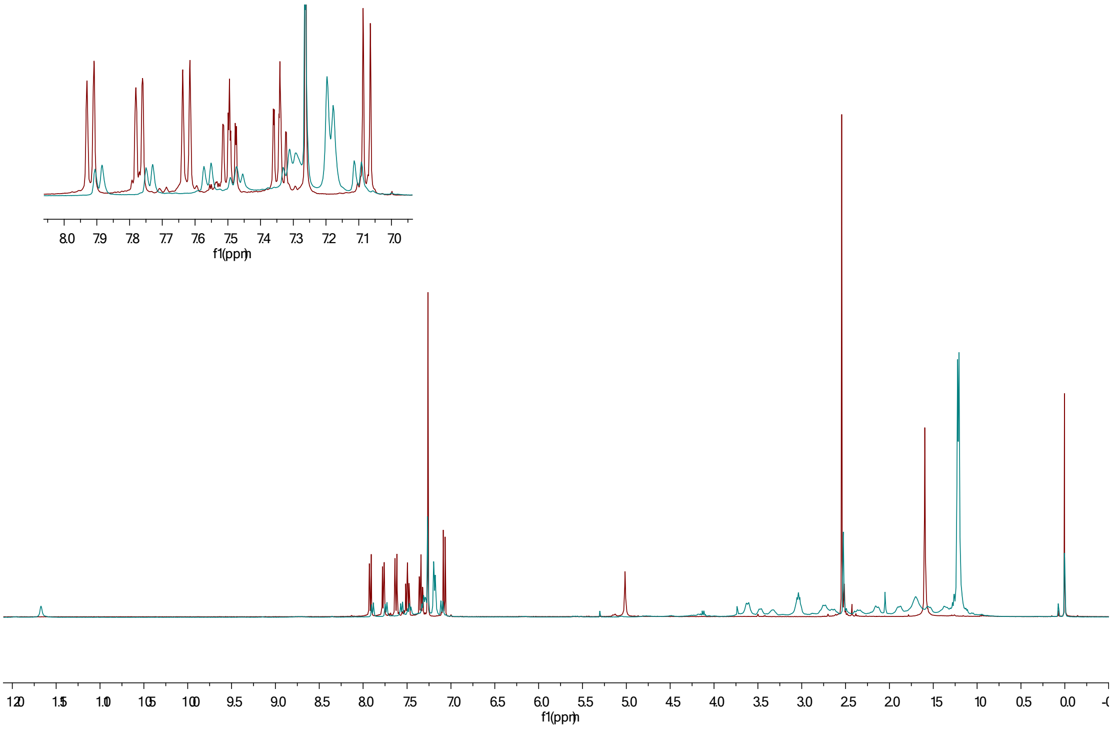

7.2 HRMS experiments

1. The mixture of $Sc(OTf)_3$ and **L-PiPr$_2$**.

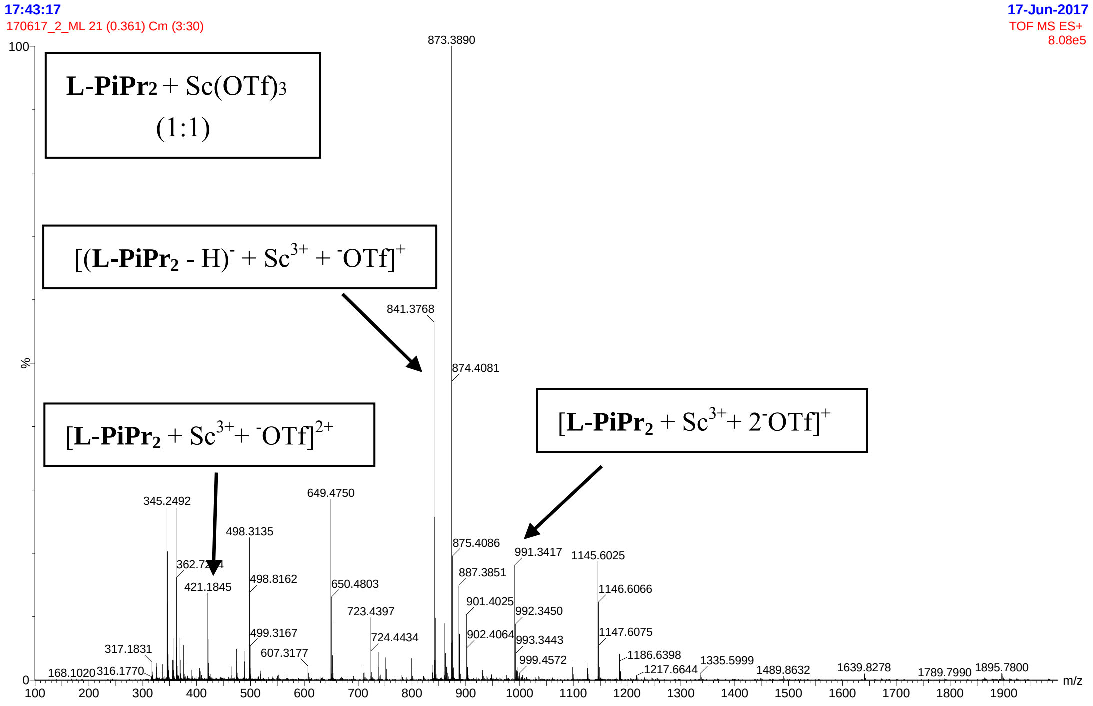

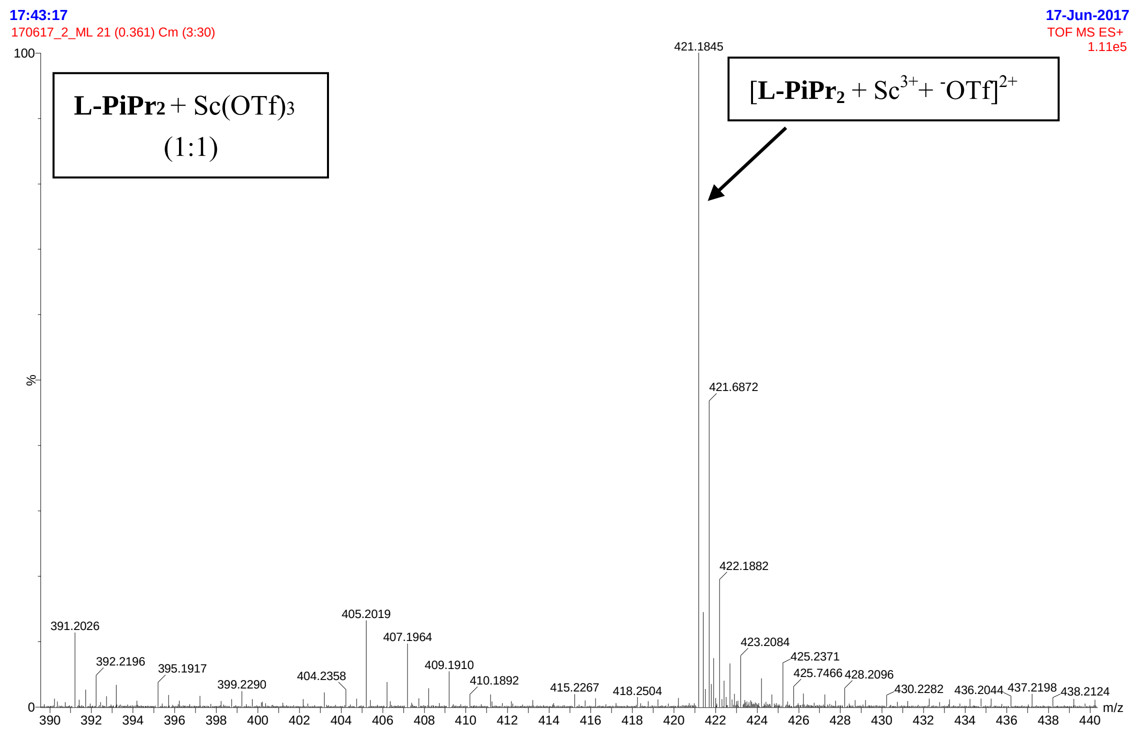

HRMS (ESI-TOF) calcd for [**L-PiPr$_2$** + Sc^{3+} + $^{-}$OTf]$^{2+}$ = 421.1841, Found 421.1845.

HRMS (ESI-TOF) calcd for [(**L-PiPr$_2$** - H)$^{-}$ + Sc^{3+} + $^{-}$OTf]$^{+}$ = 841.3610, Found 841.3768.

HRMS (ESI-TOF) calcd for [**L-PiPr$_2$** + Sc^{3+} + 2^{-}OTf]$^{+}$ = 991.3209, Found 991.3417.

2. The mixture of Sc(OTf)$_3$, **L-PiPr$_2$** and **1a**.

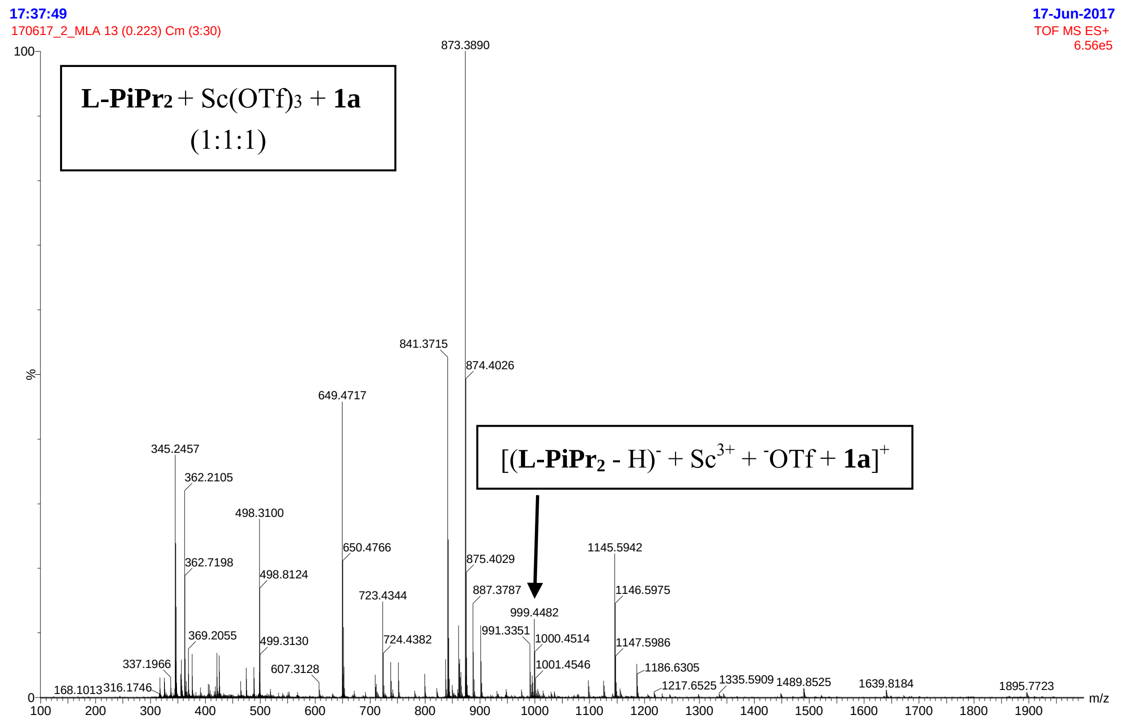

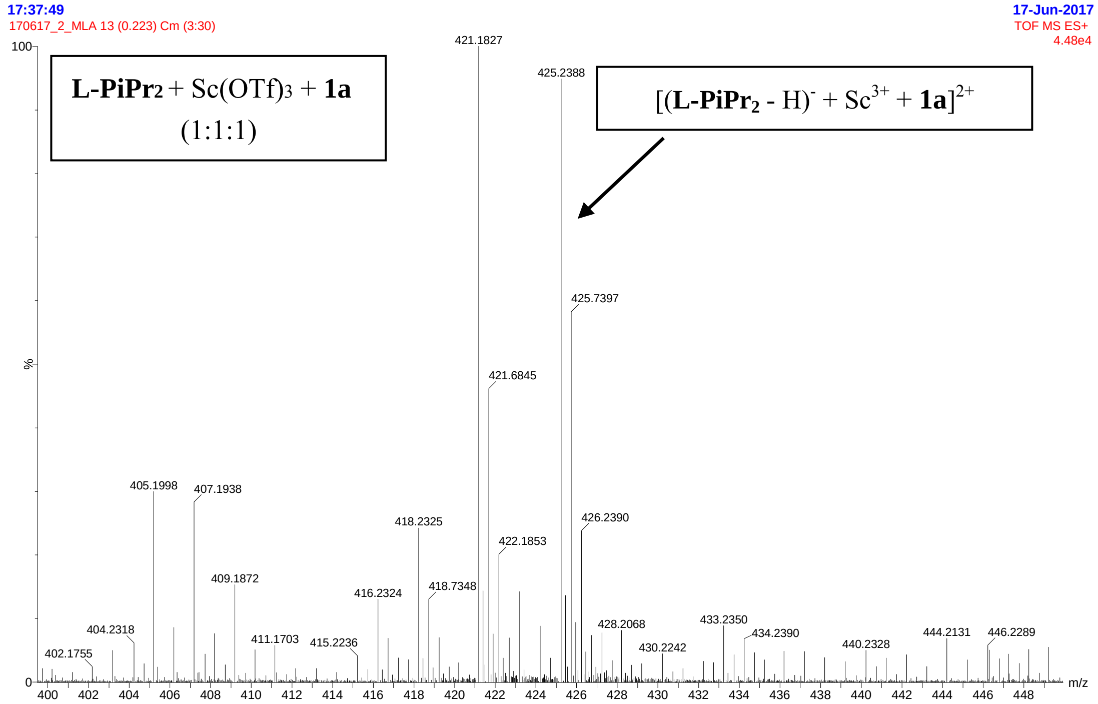

HRMS (ESI-TOF) calcd for [(**L-PiPr$_2$**-H)$^-$ + Sc^{3+} + **1a**]$^{2+}$ = 425.2408, Found 425.2388.

HRMS (ESI-TOF) calcd for [(**L-PiPr$_2$**-H)$^-$ + Sc^{3+} + $^-$OTf + **1a**]$^+$ = 999.4342, Found 999.4482.

8. Optimization of reaction conditions

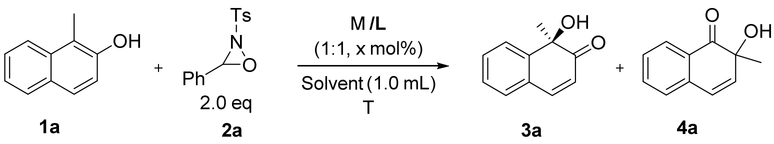

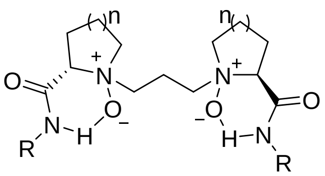

L-PrPr$_2$: R = 2,6-^{i}Pr$_2$C$_6$H$_3$, n = 1
L-PiPr$_2$: R = 2,6-^{i}Pr$_2$C$_6$H$_3$, n = 2
L-PiMe$_2$: R = 2,6-Me$_2$C$_6$H$_3$, n = 2
L-PiPr$_3$: R = 2,4,6-^{i}Pr$_3$C$_6$H$_2$, n = 2
L-PiPh: R = C$_6$H$_5$, n = 2
L-PiAd: R = 1-adamantane, n = 2

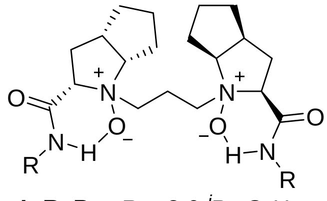

L-RaPr$_2$: R = 2,6-^{i}Pr$_2$C$_6$H$_3$

Entry[a]	**Ligand**	Metal salt	Oxidant	Solvent	x	T (°C)	Yield (%)[b]	ratio (**3a**:**4a**)[c]	Ee(**3a**/**4a**) (%)[c]
1	**L-PiPr$_2$**	Sc(OTf)$_3$	oxaziridine	DCM	10	30	96	73:27	60/64
2	**L-PrPr$_2$**	Sc(OTf)$_3$	oxaziridine	DCM	10	30	99	79:21	26/45
3	**L-RaPr$_2$**	Sc(OTf)$_3$	oxaziridine	DCM	10	30	99	75:25	7/11
4	**L-PiMe$_2$**	Sc(OTf)$_3$	oxaziridine	DCM	10	30	90	>95:5	20/n.d.

5	**L-PiPh**	$Sc(OTf)_3$	oxaziridine	DCM	10	30	92	>95:5	24/n.d.
6	**L-PiPr$_3$**	$Sc(OTf)_3$	oxaziridine	DCM	10	30	96	89:11	46/55
7	**L-PiAd**	$Sc(OTf)_3$	oxaziridine	DCM	10	30	99	87:13	20/58
8	**L-PiPr$_2$**	$Sc(acac)_3$	oxaziridine	DCM	10	30	99	>95:5	10/n.d.
9	**L-PiPr$_2$**	$Sc(O^iPr)_3$	oxaziridine	DCM	10	30	99	>95:5	18/n.d.
10	**L-PiPr$_2$**	$ScCl_3{\cdot}6H_2O$	oxaziridine	DCM	10	30	99	>95:5	10/n.d.
11	**L-PiPr$_2$**	$Sc(NTf_2)_3$	oxaziridine	DCM	10	30	99	>95:5	84/n.d.
12	**L-PiPr$_2$**	$Sc(NTf_2)_3$	*m*CPBA	DCM	10	30	60	>95:5	0/n.d.
13	**L-PiPr$_2$**	$Sc(NTf_2)_3$	H_2O_2	DCM	10	30	n.r.	--	--
14	**L-PiPr$_2$**	$Sc(NTf_2)_3$	^{t}BuOOH	DCM	10	30	n.r.	--	--
15	**L-PiPr$_2$**	$Sc(NTf_2)_3$	oxaziridine	DCM	10	0	99	>95:5	90
16^d	**L-PiPr$_2$**	$Sc(NTf_2)_3$	oxaziridine	DCM	10	-20	99	>95:5	89
17^e	**L-PiPr$_2$**	$Sc(NTf_2)_3$	oxaziridine	DCM	10	-40	58	89:11	85
18	**L-PiPr$_2$**	$Sc(NTf_2)_3$	oxaziridine	toluene	10	0	94	>95:5	86
19	**L-PiPr$_2$**	$Sc(NTf_2)_3$	oxaziridine	DCE	10	0	97	>95:5	88
20	**L-PiPr$_2$**	$Sc(NTf_2)_3$	oxaziridine	TCE	10	0	96	>95:5	74
21	**L-PiPr$_2$**	$Sc(NTf_2)_3$	oxaziridine	$CHCl_3$	10	0	92	>95:5	87
22^f	**L-PiPr$_2$**	$Sc(NTf_2)_3$	oxaziridine	DCM	10	0	99	>95:5	89
23	**L-PiPr$_2$**	$Sc(NTf_2)_3$	oxaziridine	DCM	5	0	99	>95:5	90
24	**L-PiPr$_2$**	$Sc(NTf_2)_3$	oxaziridine	$CHCl_3$	2.5	0	94	>95:5	88
25^d	**L-PiPr$_2$**	$Sc(NTf_2)_3$	oxaziridine	$CHCl_3$	1	0	86	>95:5	87

^{a}Unless otherwise noted, the reactions were performed with x mol% metal, x mol% ligand, **1a** (0.1 mmol) and **2** (0.2 mmol) in solvent (1.0 mL) under N_2 at 30 °C for 3 h. ^{b}Isolated tatol yield of **3a** and **4a** by silica gel chromatography. ^{c}Determined by chiral HPLC analysis (Chiralcel IE) after flash column chromatography purification. ^{d}The reaction time was 4 h. ^{e}The reaction time was 22 h. ^{f}**2a** (0.15 mmol) was used.

9. Full list of the substrate scope

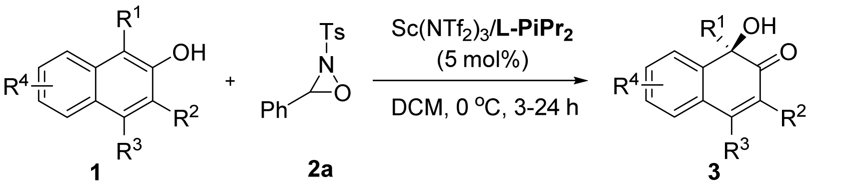

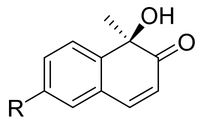

3b: R = Br, 3 h, 99% yield, 94:6 er
3c: R = OMe, 3 h, 99% yield, 94.5:5.5 er

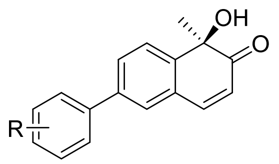

3d: R = H, 3 h, 99% yield, 94:6 er
3e: R = 4-Cl, 3 h, 95% yield, 94:6 er
3f: R = 2-MeO, 3 h, 99% yield, 93.5:6.5 er
3g: R = 3-MeO, 3 h, 99% yield, 95:5 er
3h: R = 4-MeO, 3 h, 99% yield, 94.5:5.5 er
3i: R = 3,4-MeO_2, 7 h, 99% yield, 93.5:6.5 er
3j: R = Piperonyl, 4 h, 99% yield, 95:5 er
3k: R = 2-Naphthyl, 5 h, 96% yield, 94:6 er
3l: R = 3-Furyl, 3 h, 99% yield, 95:5 er

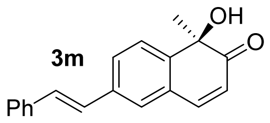

3.5 h, 99% yield, 94.5:5.5 er

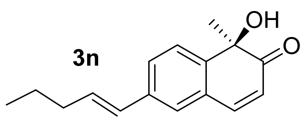

3 h, 99% yield, 95:5 er

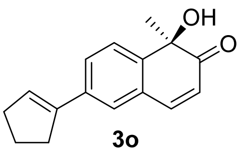

3o
3 h, 95% yield, 95:5 er

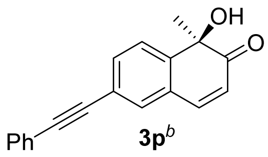

3p[b]
5 h, 99% yield, 92:8 er

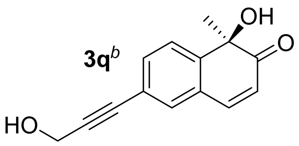

24 h, 66% yield, 91:9 er

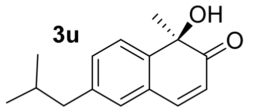

3h, 96% yield, 94.5:5.5 er

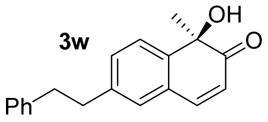

3 h, 95% yield, 95:5 er

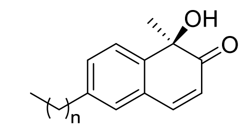

n = 0, **3r**: 3 h, 99% yield, 94:6 er
n = 1, **3s**: 3 h, 99% yield, 94:6 er
n = 4, **3t**: 3 h, 99%yield, 94:6 er

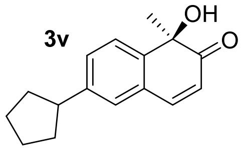

3 h, 99% yield, 94.5:5.5 er

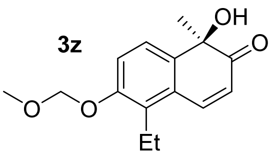

3 h, 99% yield, 95:5 er

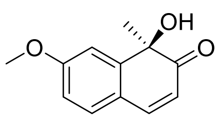

3x
3 h
99% yield, 94.5:5.5 er

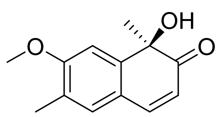

3y
3 h
99% yield, 95:5 er

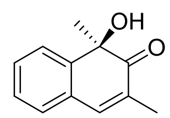

3aa[c]
3 h
99% yield, 83.5:16.5 er

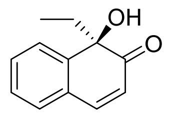

3ab
22 h
64% yield[d], 75:25 er

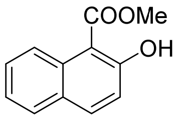

1ac
no reaction

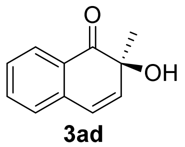

3ad
22h, 46% yield, 77:23 er

[a] Reaction conditions: The same as entry 7 in Table 1. [b] 10 mol% catalyst loading.
[c] **L-PiEt₂**-Sc(OTf)₃ (1:1, 5 mol%). [d] Total yield of **3ab** and **4ab**, **3ab**/**4ab** = 87:13.

10. The analytical and spectral characterization data of the products

(*R*)-1-hydroxy-1-methylnaphthalen-2(1H)-one (3a)

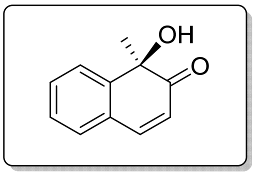

Colorless oil; 99% yield, 95:5 er; $[\alpha]^{25}_{D}$ = 265.96 (*c* = 0.28 in CH_2Cl_2).

HPLC DAICEL CHIRALCEL IE, n-hexane/2-propanol = 90/10, flow rate = 1.0 mL/min, λ = 254 nm, t_R (minor) = 15.90 min, t_R (major) = 16.42 min. ^{1}H NMR (400 MHz, $CDCl_3$) δ = 7.72 (d, *J* = 7.6 Hz, 1H), 7.47 - 7.41 (m, 2H), 7.35 - 7.28 (m, 2H), 6.20 (d, *J* = 10.0 Hz, 1H), 3.74 (s, 1H), 1.55 (s, 3H); ^{13}C NMR (101 MHz, $CDCl_3$) δ = 205.4, 146.1, 145.2, 130.8, 129.6, 128.4, 128.0, 125.7, 122.5, 77.3, 33.3. HRMS (ESI-TOF) calcd for $C_{11}H_{10}O_2Na^+$ ([M+Na$^+$]) = 197.0573, Found 197.0574.

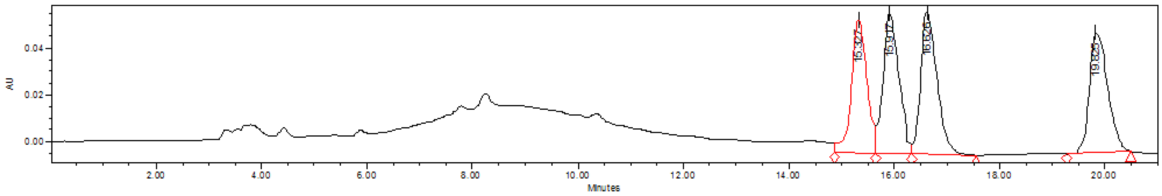

	Retention Time	Area	% Area
1	15.327	1171769	23.50
2	15.917	1270613	25.48
3	16.626	1316241	26.40
4	19.825	1227859	24.62

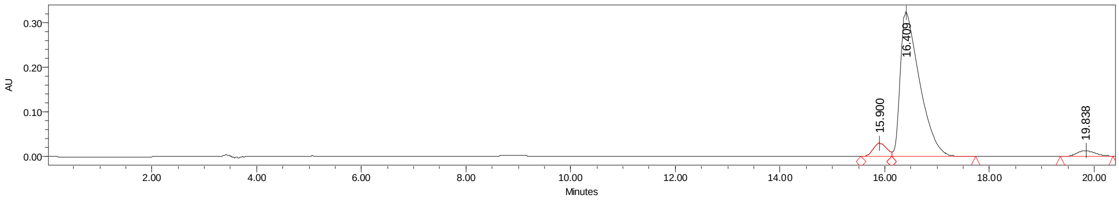

	Retention Time	Area	% Area
1	15.900	254413	4.77
2	16.419	4909175	92.03
3	19.838	170718	3.20

(*R*)-6-bromo-1-hydroxy-1-methylnaphthalen-2(1H)-one (3b)

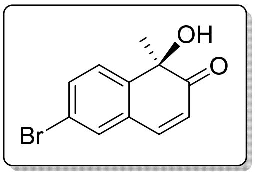

White solid; m.p. 54-56 °C; 99% yield, 94:6 er; $[\alpha]^{16}_{D}$ = 115.26 (*c* = 0.49 in CH_2Cl_2).

UPC[2] Phenomenex CHIRALCEL Lux 5u Cellulose-1, $scCO_2$/Methol = 97/3, flow rate = 1.0 mL/min, λ = 254 nm, t_R (major) = 11.0 min, t_R (minor) = 11.88 min. ^{1}H NMR (400 MHz, $CDCl_3$) δ = 7.61 - 7.57 (m, 1H), 7.57 - 7.52 (m, 1H), 7.44 (d, *J* = 2.0 Hz, 1H), 7.35 (d, *J* = 10.0 Hz, 1H), 6.23 (d, *J* = 9.8 Hz, 1H), 3.71 (s, 1H), 1.52 (s, 3H); ^{13}C NMR (101 MHz, $CDCl_3$) δ = 204.5, 144.4, 143.9, 133.4, 132.0, 130.3, 127.5, 123.8, 121.7, 77.1, 33.0. HRMS (ESI-TOF) calcd for $C_{11}H_9{}^{79}BrO_2Na^+$ ($[M+Na^+]$) = 274.9678, Found 274.9678. HRMS (ESI-TOF) calcd for $C_{11}H_9{}^{81}BrO_2Na^+$ ($[M+Na^+]$) = 276.9658, Found 276.9660.

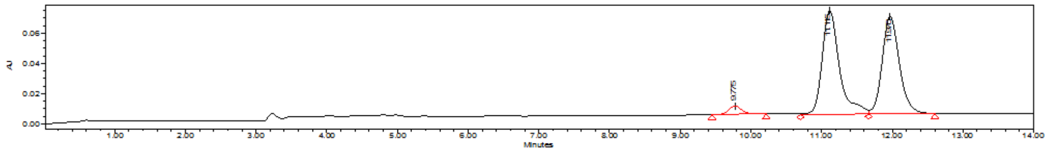

	Retention Time	Area	% Area
1	9.775	67163	2.89
2	11.115	1170495	50.32
3	11.970	1088509	46.79

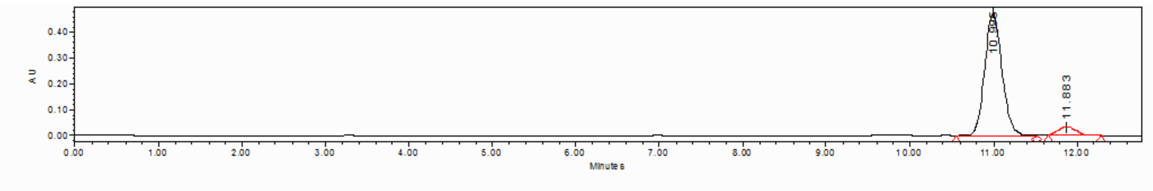

	Retention Time	Area	% Area
1	10.995	6817001	93.99
2	11.883	435620	6.01

(*R*)-1-hydroxy-6-methoxy-1-methylnaphthalen-2(1H)-one (3c)

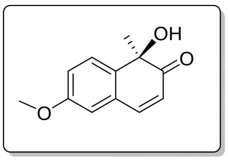

Colorless oil; m.p. 106-108 °C; 99% yield, 94.5:5.5 er; $[\alpha]^{25}_{D}$ = 216.97 (*c* = 0.33 in CH_2Cl_2).

HPLC DAICEL CHIRALCEL IC, n-hexane/2-propanol = 90/10, flow rate = 1.0 mL/min, λ = 254 nm, t_R (major) = 21.35 min, t_R (minor) = 26.86 min. ^{1}H NMR (400 MHz, $CDCl_3$) δ = 7.61 (d, *J* = 8.4 Hz, 1H), 7.37 (d, *J* = 9.6 Hz, 1H), 6.97 (dd, *J* = 8.4, 2.4 Hz, 1H), 6.82 (d, *J* = 2.4 Hz, 1H), 6.20 (d, *J* = 10.0 Hz, 1H), 3.83 (s, 3H), 3.66 (s, 1H), 1.52 (s, 3H); ^{13}C NMR (101 MHz, $CDCl_3$) δ = 205.5, 159.2, 145.9, 137.1, 129.5, 127.0, 123.1, 116.0, 114.8, 77.2, 55.6, 33.2. HRMS (ESI-TOF) calcd for $C_{12}H_{12}O_3$ ([M+Na$^+$]) = 227.0679, Found 227.0671.

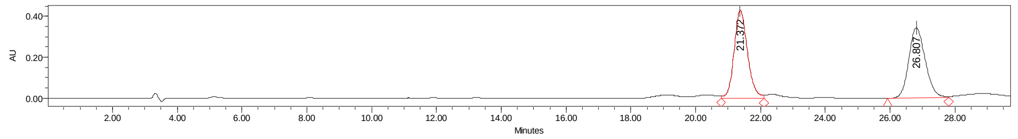

	Retention Time	Area	% Area
1	21.372	12393739	50.80
2	26.807	12001302	49.20

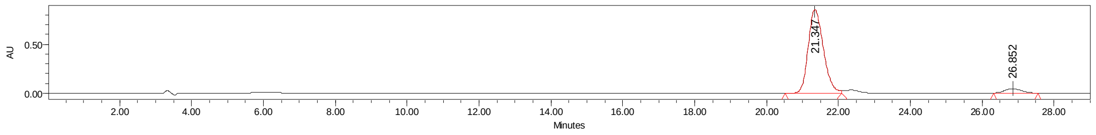

	Retention Time	Area	% Area
1	21.347	25145644	94.49
2	26.852	1466626	5.51

(*R*)-1-hydroxy-1-methyl-6-phenylnaphthalen-2(1H)-one (3d)

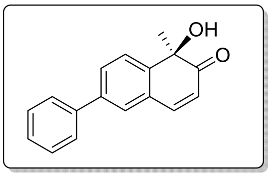

Colorless oil; 99% yield, 94:6 er; $[\alpha]^{16}_D$ = 82.69 (*c* = 0.34 in CH_2Cl_2).

HPLC DAICEL CHIRALCEL IA, n-hexane/2-propanol = 95/5, flow rate = 1.0 mL/min, λ = 254 nm, t_R (minor) = 17.00 min, t_R (major) = 20.50 min. ^{1}H NMR (400 MHz, $CDCl_3$) δ = 7.79 (d, *J* = 8.0 Hz, 1H), 7.67 (dd, *J* = 8.0, 2.0 Hz, 1H), 7.63 - 7.56 (m, 2H), 7.54 - 7.44 (m, 4H), 7.43 - 7.36 (m, 1H), 6.27 (d, *J* = 9.6 Hz, 1H), 3.76 (s, 1H), 1.60 (s, 3H); ^{13}C NMR (101 MHz, $CDCl_3$) δ = 205.2, 146.0, 143.9, 141.1, 140.0, 129.4, 129.0, 128.8, 128.2, 127.9, 127.1, 126.2, 122.9, 77.2, 33.2.

HRMS (ESI-TOF) calcd for $C_{17}H_{14}O_2Na^+$ ([M+Na+]) = 273.0886, Found 273.0890.

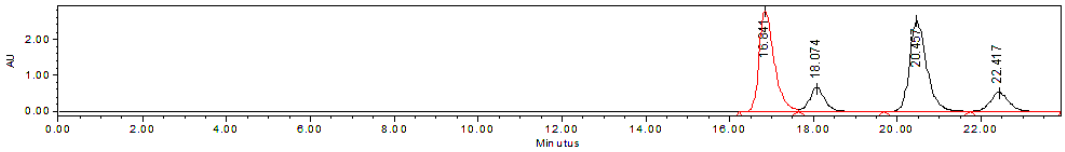

	Retention Time	Area	% Area
1	16.841	69088735	40.19
2	18.074	16437326	9.56
3	20.457	70564046	41.05
4	22.417	15815117	9.20

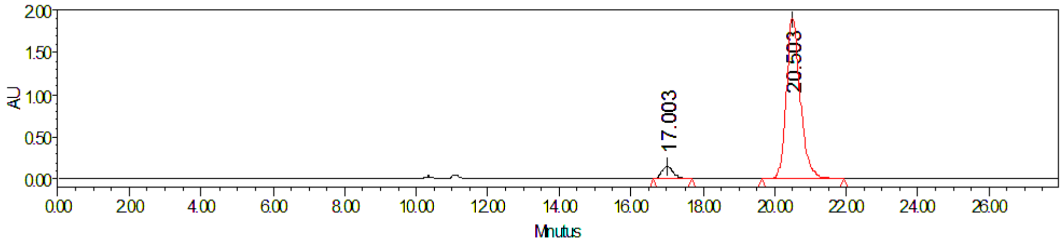

	Retention Time	Area	% Area
1	17.003	3235758	5.88
2	20.503	51822510	94.12

(*R*)-6-(4-chlorophenyl)-1-hydroxy-1-methylnaphthalen-2(1H)-one (3e)

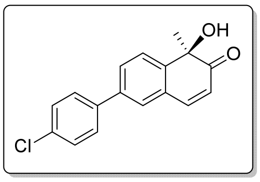

White solid; m.p. 60-61 °C; 95% yield, 94:6 er; $[\alpha]^{25}_{D}$ = 46.67 (*c* = 0.42 in CH_2Cl_2).

HPLC DAICEL CHIRALCEL IA, n-hexane/2-propanol = 90/10, flow rate = 1.0 mL/min, λ = 254 nm, t_R (major) = 20.75 min, t_R (minor) = 16.08 min. ^{1}H NMR (400 MHz, $CDCl_3$) δ = 7.83 - 7.74 (m, 1H), 7.65 - 7.60 (m, 1H), 7.55 - 7.40 (m, 6H), 6.35 - 6.22 (m, 1H), 3.79 - 3.72 (m, 2H), 164 - 1.54 (m, 3H); ^{13}C NMR (101 MHz, $CDCl_3$) δ = 205.1, 145.8, 144.2, 139.9, 138.4, 134.1, 129.2, 129.2, 129.0, 128.4, 128.0, 126.3, 123.1, 77.2, 33.2. HRMS (ESI-TOF) calcd for $C_{17}H_{13}{}^{35}ClO_2Na^+$ ([M+Na+]) = 307.0496, Found 307.0501. HRMS (ESI-TOF) calcd for $C_{17}H_{13}{}^{37}ClO_2Na^+$ ([M+Na+]) = 309.0467, Found 309.0481.

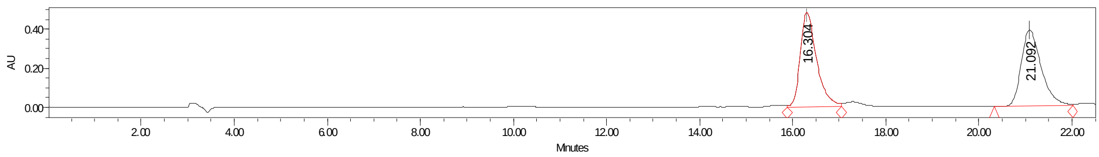

	Retention Time	Area	% Area
1	16.304	11815349	50.21
2	21.092	11716966	49.79

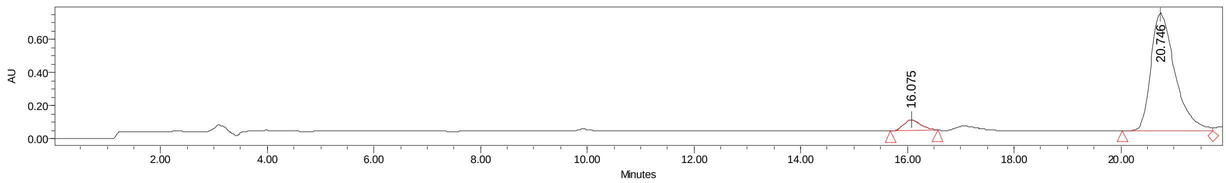

	Retention Time	Area	% Area
1	16.075	1420823	6.03
2	20.746	22148076	93.97

(*R*)-1-hydroxy-6-(2-methoxyphenyl)-1-methylnaphthalen-2(1H)-one (3f)

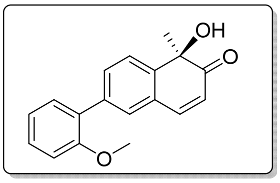

Colorless oil; 99% yield, 93.5:6.5 er; $[\alpha]^{25}_{D}$ = 77.08 (*c* = 0.48 in CH_2Cl_2).

HPLC DAICEL CHIRALCEL IC, n-hexane/2-propanol = 90/10, flow rate = 1.0 mL/min, λ = 254 nm, t_R (major) = 20.50 min, t_R (minor) = 24.99 min. ^{1}H NMR (400 MHz, $CDCl_3$) δ = 7.75 (d, *J* = 7.6 Hz, 1H), 7.61 (d, *J* = 7.6 Hz, 1H), 7.51 - 7.44 (m, 2H), 7.39 - 7.29 (m, 2H), 7.09 - 6.97 (m, 2H), 6.22 (d, *J* = 10.0 Hz, 1H), 3.83 (s, 3H), 3.74 (s, 1H), 1.60 (s, 3H); ^{13}C NMR (101 MHz, $CDCl_3$) δ = 205.5, 156.5, 146.4, 143.5, 138.4, 131.9, 130.8, 130.7, 129.4, 129.3, 128.2, 125.5, 122.5, 121.1, 111.4, 77.2, 55.7, 33.2. HRMS (ESI-TOF) calcd for $C_{18}H_{16}O_3Na^+$ ([M+Na$^+$]) = 303.0992, Found 303.0995.

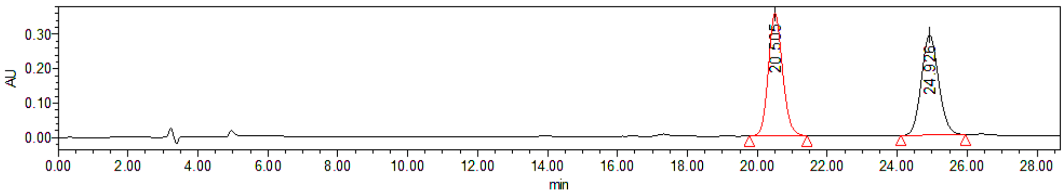

	Retention Time	Area	% Area
1	20.505	10062884	50.24
2	24.926	9968259	49.76

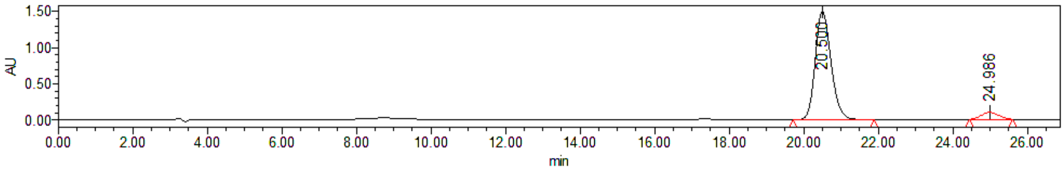

	Retention Time	Area	% Area
1	20.500	44063776	93.50
2	24.986	3062300	6.50

(*R*)-1-hydroxy-6-(3-methoxyphenyl)-1-methylnaphthalen-2(1H)-one (3g)

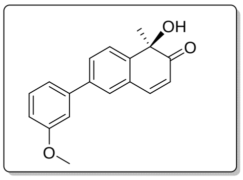

Colorless oil; 99% yield, 95:5 er; $[\alpha]^{25}_{D}$ = 66.67 (c = 0.49 in CH_2Cl_2).

HPLC DAICEL CHIRALCEL IC, n-hexane/2-propanol = 90/10, flow rate = 1.0 mL/min, λ = 254 nm, t_R (major) = 23.28 min, t_R (minor) = 26.30 min. ^{1}H NMR (400 MHz, $CDCl_3$) δ = 7.78 (d, J = 8.0 Hz, 1H), 7.68 - 7.63 (m, 1H), 7.53 - 7.46 (m, 2H), 7.41 - 7.35 (m, 1H), 7.21 - 7.15 (m, 1H), 7.14 - 7.08 (m, 1H), 6.93 (d, J = 8.4 Hz, 1H) 6.25 (d, J = 10.0 Hz, 1H), 3.88 (s, 3H), 3.76 (s, 1H), 1.59 (s, 3H); ^{13}C NMR (101 MHz, $CDCl_3$) δ = 205.2, 160.2, 146.0, 144.1, 141.5, 141.0, 130.1, 129.4, 128.8, 128.2, 126.2, 123.0, 119.6, 113.2, 113.0, 77.2, 55.5, 33.2. HRMS (ESI-TOF) calcd for $C_{18}H_{16}O_3Na^+$ ([M+Na$^+$]) = 303.0992, Found 303.0999.

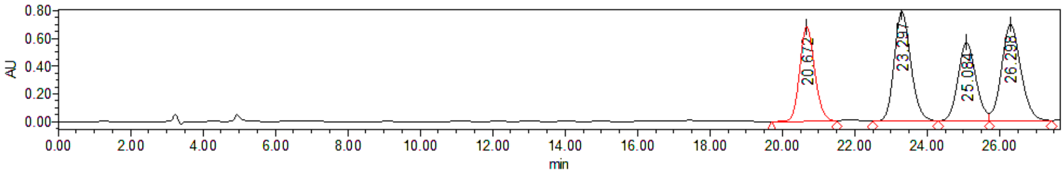

	Retention Time	Area	% Area
1	20.672	19684235	21.67
2	23.297	25374240	27.94
3	25.084	19703184	21.69
4	26.298	26065449	28.70

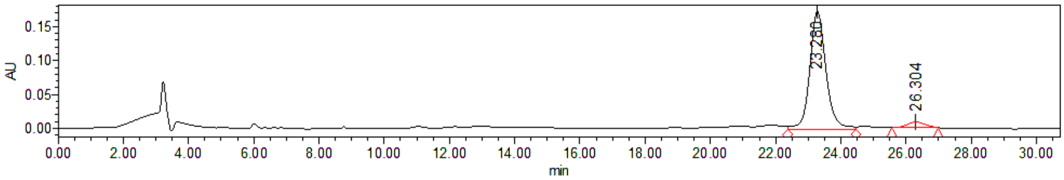

	Retention Time	Area	% Area
1	23.280	6046085	94.83
2	26.304	329482	5.17

***(R)*-1-hydroxy-6-(4-methoxyphenyl)-1-methylnaphthalen-2(1H)-one (3h)**

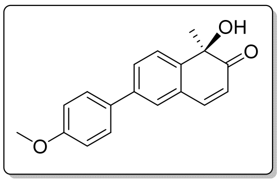

White solid; m.p. 106-108 °C; 99% yield, 94.5:5.5 er; $[\alpha]^{25}_{D}$ = 56.40 (c = 0.49 in CH_2Cl_2).

HPLC DAICEL CHIRALCEL IA, n-hexane/2-propanol = 90/10, flow rate = 1 mL/min, λ = 254 nm, t_R (minor) = 18.07 min, t_R (major) = 22.51 min. ^{1}H NMR (400 MHz, $CDCl_3$) δ = 7.76 (d, J = 8.0 Hz, 1H), 7.65 - 7.59 (m, 1H), 7.56 - 7.44 (m, 4H), 7.04 - 6.95 (m, 2H), 6.24 (d, J = 10.0 Hz, 1H), 3.86 (s, 3H), 3.76 (s, 1H), 1.59 (s, 3H); ^{13}C NMR (101 MHz, $CDCl_3$) δ = 205.3, 159.6, 146.1, 143.2, 140.7, 132.5, 128.9, 128.8, 128.2, 127.8, 126.2, 122.8, 114.5, 77.1, 55.5, 33.2. HRMS (ESI-TOF) calcd for $C_{18}H_{16}O_3Na^+$ ($[M+Na^+]$) = 303.0992, Found 303.0998.

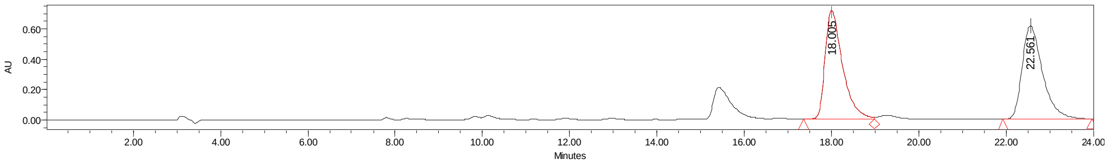

	Retention Time	Area	% Area
1	18.005	20033195	50.05
2	22.561	19992568	49.95

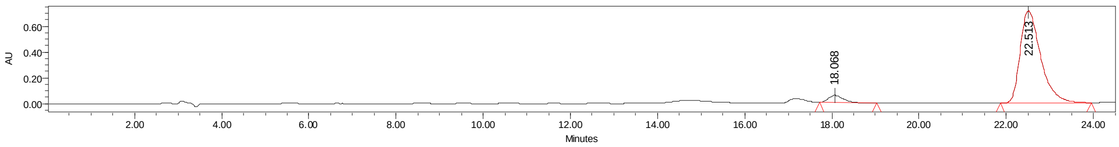

	Retention Time	Area	% Area
1	18.068	1375884	5.55
2	22.513	23435575	94.45

(*R*)-6-(3,4-dimethoxyphenyl)-1-hydroxy-1-methylnaphthalen-2(1H)-one (3i)

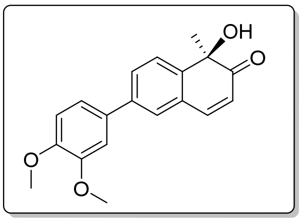

Yellow solid; m.p. 126-128 °C; 99% yield, 93.5:5.5 er; $[\alpha]^{25}_{D}$ = 48.32 (c = 0.24 in CH_2Cl_2).

HPLC DAICEL CHIRALCEL IA, n-hexane/2-propanol = 80/20, flow rate = 1.0 mL/min, λ = 254 nm, t_R (minor) = 14.22 min, t_R (major) = 17.81 min. ^{1}H NMR (400 MHz, $CDCl_3$) δ = 7.76 (d, J = 8.0 Hz, 1H), 7.66 - 7.60 (m, 1H), 7.53 - 7.44 (m, 2H), 7.17 - 7.12 (m, 1H), 7.11 - 7.05 (m, 1H) 6.96 (d, J = 8.4 Hz, 1H), 6.25 (d, J = 9.6 Hz, 2H), 3.96 (s, 3H), 3.93 (s, 3H), 3.72 (s, 1H), 1.59 (s, 3H); ^{13}C NMR (101 MHz, $CDCl_3$) δ = 205.3, 149.4, 149.2, 146.1, 143.4, 141.0, 133.0, 129.1, 128.8, 127.9, 126.2, 122.9, 119.5, 111.7, 110.4, 77.2, 56.1, 33.2. HRMS (ESI-TOF) calcd for $C_{19}H_{18}O_4$ ($[M+K^+]$) = 333.1097, Found 333.1089.

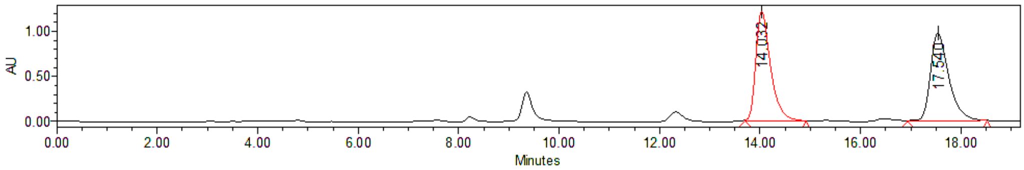

	Retention Time	Area	% Area
1	14.032	25022993	50.37
2	17.540	24659166	49.63

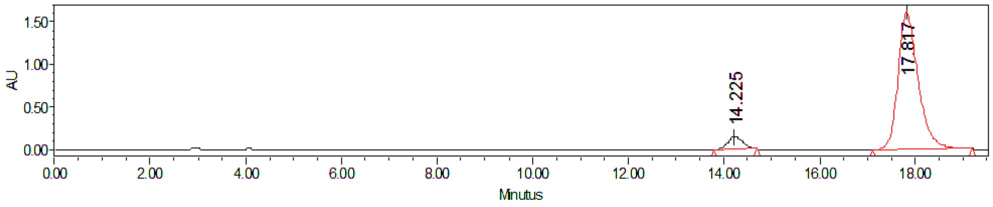

	Retention Time	Area	% Area
1	14.225	3182310	6.39
2	17.817	46586421	93.61

(*R*)-6-(benzo[d][1,3]dioxol-5-yl)-1-hydroxy-1-methylnaphthalen-2(1H)-one (3j)

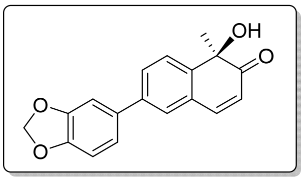

White solid; m.p. 79-83 °C 99% yield, 95:5 er; $[\alpha]^{25}_{D}$ = 48.05 (c = 0.31 in CH_2Cl_2).

HPLC DAICEL CHIRALCEL IA, n-hexane/2-propanol = 90/10, flow rate = 1.0 mL/min, λ = 254 nm, t_R (minor) = 22.94 min, t_R (major) = 25.30 min. ^{1}H NMR (400 MHz, $CDCl_3$) δ = 7.74 (d, J = 8.0 Hz, 1H), 7.58 (dd, J = 8.0, 2.0 Hz, 1H), 7.48 (d, J = 10.0 Hz, 1H), 7.42 (d, J = 2.0 Hz, 1H), 7.11 - 6.98 (m, 2H), 6.90 (d, J = 8.4 Hz, 1H), 6.24 (d, J = 9.6 Hz, 1H), 6.02 (s, 2H), 3.71 (s, 1H), 1.58 (s, 3H); ^{13}C NMR (101 MHz, $CDCl_3$) δ = 205.2, 148.4, 147.6, 146.0, 143.6, 140.8, 134.3, 129.1, 128.8, 127.9, 126.2, 123.0, 120.7, 108.8, 107.6, 101.4, 77.2, 33.2. HRMS (ESI-TOF) calcd for $C_{18}H_{14}O_4Na^+$ ([M+Na$^+$]) = 317.0784, Found 317.0782.

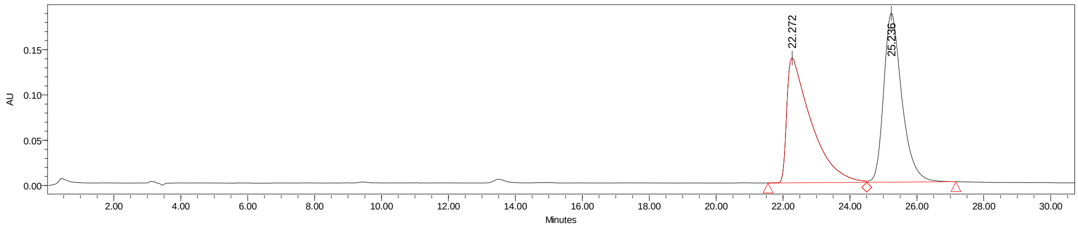

	Retention Time	Area	% Area
1	22.272	6984701	49.77
2	25.236	7050306	50.23

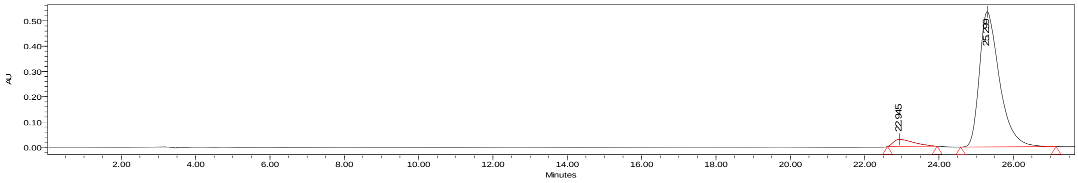

	Retention Time	Area	% Area
1	22.945	1074667	5.09
2	25.299	20022264	94.91

(*R*)-5-hydroxy-5-methyl-[2,2'-binaphthalen]-6(5H)-one (3k)

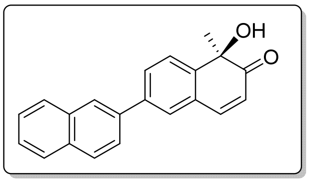

White solid; m.p. 84-87 °C 96% yield, 94:6 er; $[\alpha]^{25}_{D}$ = 23.85 (c = 0.52 in CH_2Cl_2).

HPLC DAICEL CHIRALCEL IA, n-hexane/2-propanol = 90/10, flow rate = 1.0 mL/min, λ = 254 nm, t_R (minor) = 16.37 min, t_R (major) = 19.31 min. ^{1}H NMR (400 MHz, $CDCl_3$) δ = 8.04 (s, 1H), 7.96 - 7.86 (m, 3H), 7.86 - 7.77 (m, 2H), 7.75 - 7.71 (m, 1H), 7.66 - 7.61 (m, 1H), 7.57 - 7.48 (m, 3H) 6.28 (d, *J* = 9.6 Hz, 1H), 3.75 (s, 1H), 1.62 (s, 3H); ^{13}C NMR (101 MHz, $CDCl_3$) δ = 205.2, 146.0, 144.0 141.0, 137.3, 133.7, 132.9, 129.6, 129.0, 128.8, 128.4, 128.3, 127.8, 126.6, 126.4, 126.3, 126.0, 125.3, 123.0, 77.2, 33.2. HRMS (ESI-TOF) calcd for $C_{21}H_{16}O_2Na^+$ ([M+Na$^+$]) = 323.1043, Found 323.1042.

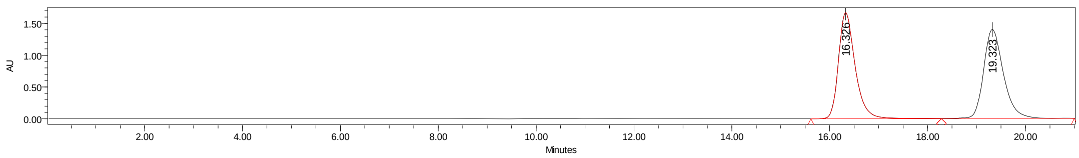

	Retention Time	Area	% Area
1	16.326	39385592	50.02
2	19.323	39357923	49.98

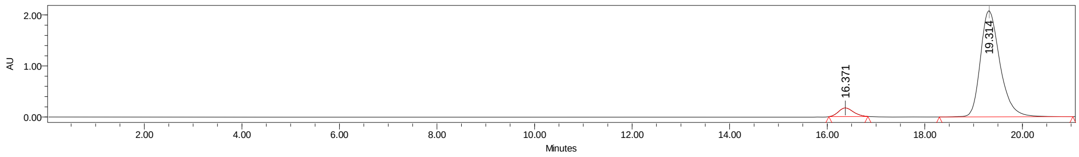

	Retention Time	Area	% Area
1	16.371	3592299	5.84
2	19.314	57955793	94.16

(*R*)-6-(furan-3-yl)-1-hydroxy-1-methylnaphthalen-2(1H)-one (3l)

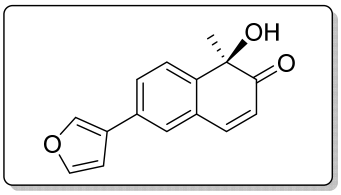

Yellow solid; m.p. 60-63 °C; 99% yield, 95:5 er; $[\alpha]^{25}_D$ = 105.71 (*c* = 0.42 in CH_2Cl_2).

HPLC DAICEL CHIRALCEL IA, n-hexane/2-propanol = 90/10, flow rate = 1.0 mL/min, λ = 254 nm, t_R (major) = 18.14 min, t_R (minor) = 15.67 min. ^{1}H NMR (400 MHz, $CDCl_3$) δ = 7.78 - 7.67 (m, 2H), 7.57 - 7.52 (m, 1H), 7.52 - 7.48 (m, 1H), 7.44 (d, *J* = 9.6 Hz, 1H) 7.40 (s, 1H),

6.74 - 6.65 (m, 1H), 6.29 - 6.17 (m, 1H), 3.73 (s, 1H), 1.56 (d, J = 1.2 Hz, 3H); ^{13}C NMR (101 MHz, $CDCl_3$) δ = 205.2, 145.8, 144.1, 143.6, 138.9, 132.4, 128.9, 128.0, 126.8, 126.2, 125.6, 123.0, 108.8, 77.2, 33.2. HRMS (ESI-TOF) calcd for $C_{15}H_{12}O_3Na^+$ ([M+Na$^+$]) = 263.0679, Found 263.0683.

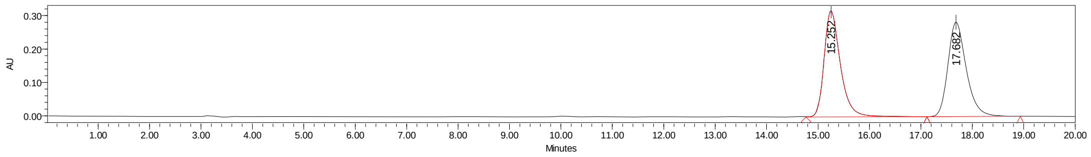

	Retention Time	Area	% Area
1	15.252	6964243	49.96
2	17.682	6975280	50.04

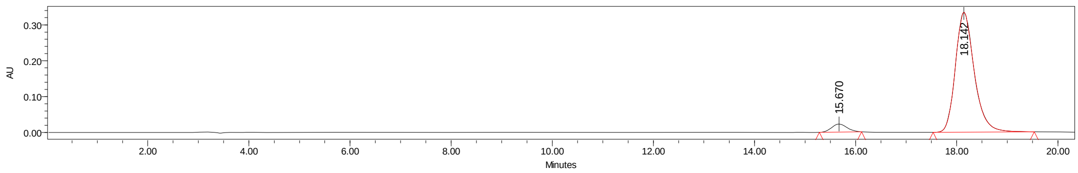

	Retention Time	Area	% Area
1	15.670	475066	5.20
2	18.142	8658167	94.80

(*R*,*E*)-1-hydroxy-1-methyl-6-styrylnaphthalen-2(1H)-one (3m)

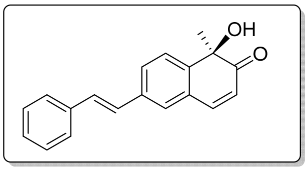

Yellow oil; 99% yield, 94.5:5.5 er; $[\alpha]^{25}_D$ = 22.20 (c = 0.54 in CH_2Cl_2).

HPLC DAICEL CHIRALCEL IC, n-hexane/2-propanol = 90/10, flow rate = 1 mL/min, λ = 254 nm, t_R (major) = 21.19 min, t_R (minor) = 24.05 min. ^{1}H NMR (400 MHz, $CDCl_3$) δ = 7.70 (d, J = 8.0 Hz, 1H), 7.60 - 7.55 (m, 1H), 7.54 - 7.48 (m, 2H), 7.47 - 7.33 (m, 4H), 7.32- 7.27 (m, 1H), 7.18 - 7.05 (m, 2H), 6.23 (d, J = 10.0 Hz, 1H), 3.73 (s, 1H), 1.56 (s, 3H); ^{13}C NMR (101 MHz, $CDCl_3$) δ = 205.2, 146.0, 144.1, 137.3, 137.0, 129.8, 128.9, 128.8, 128.6, 128.1, 127.5, 127.4, 126.7, 126.1, 123.0, 77.2, 33.2. HRMS (ESI-TOF) calcd for $C_{19}H_{16}O_2Na^+$ ([M+Na$^+$]) = 299.1043, Found 299.1042.

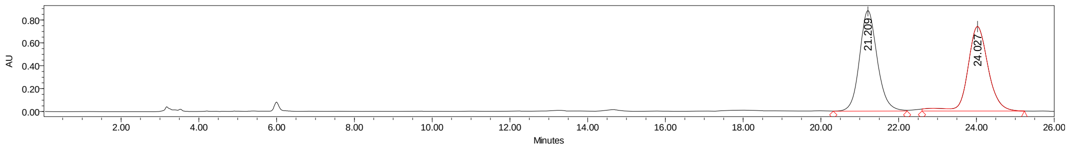

	Retention Time	Area	% Area
1	21.209	27722402	50.88
2	24.027	26758844	49.12

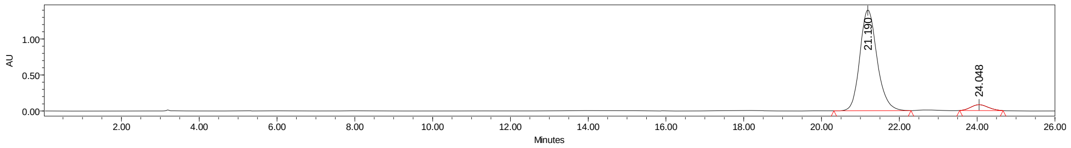

	Retention Time	Area	% Area
1	21.190	42856182	94.49
2	24.048	2498166	5.51

(*R*,*E*)-1-hydroxy-1-methyl-6-(pent-1-en-1-yl)naphthalen-2(1H)-one (3n)

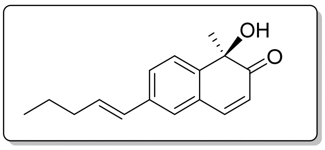

Colorless oil; 99% yield, 95:5 er; $[\alpha]^{25}_{D}$ = 124.19 (*c* = 0.43 in CH_2Cl_2).

HPLC DAICEL CHIRALCEL IE, n-hexane/2-propanol = 90/10, flow rate = 1.0 mL/min, λ = 254 nm, t_R (major) = 14.40 min, t_R (minor) = 15.27 min. ^{1}H NMR (400 MHz, $CDCl_3$) δ = 7.63 (d, *J* = 8.0 Hz, 1H), 7.46 - 7.34 (m, 2H), 7.28 - 7.23 (m, 1H), 6.42 - 6.33 (m, 1H), 6.33 - 6.22 (m, 1H), 6.20 (d, *J* = 10.0 Hz, 1H), 3.70 (s, 1H), 2.35 - 2.11 (m, 2H), 1.63 - 1.42 (m, 5H), 0.96 (t, *J* = 7.6 Hz, 3H); ^{13}C NMR (101 MHz, $CDCl_3$) δ = 205.3, 146.2, 143.3, 137.8, 132.3, 128.8, 128.5, 128.1, 127.0, 125.8, 122.7, 77.1, 35.2, 33.2, 22.6, 13.9. HRMS (ESI-TOF) calcd for $C_{16}H_{18}O_2Na^+$ ($[M+Na^+]$) = 265.1199, Found 265.1197.

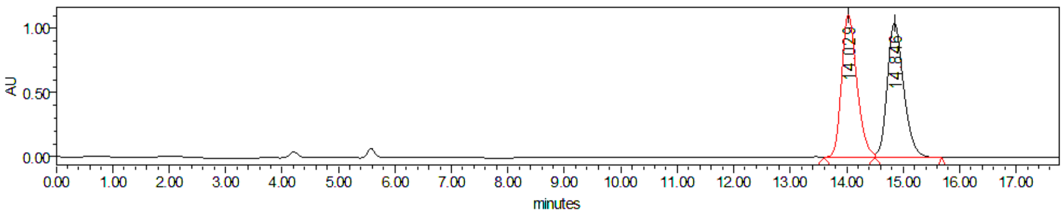

	Retention Time	Area	% Area
1	14.029	21080415	49.91
2	14.846	21157140	50.09

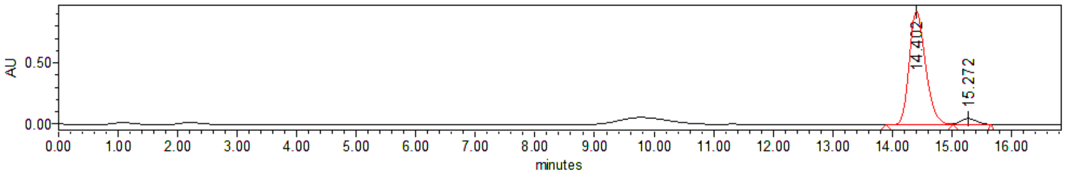

	Retention Time	Area	% Area
1	14.402	17594228	95.14
2	15.272	898529	4.86

(*R*)-6-(cyclopent-1-en-1-yl)-1-hydroxy-1-methylnaphthalen-2(1H)-one (3o)

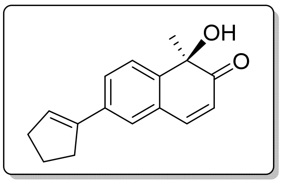

Yellow solid; m.p. 48-52 °C; 95% yield, 95:5 er; $[\alpha]^{25}_{D}$ = 114.11 (c = 0.33 in CH_2Cl_2).

HPLC DAICEL CHIRALCEL IC, n-hexane/2-propanol = 80/20, flow rate = 1.0 mL/min, λ = 254 nm, t_R (major) = 11.63 min, t_R (minor) = 14.96 min. ^{1}H NMR (400 MHz, $CDCl_3$) δ = 7.64 (d, J = 8.0 Hz, 1H), 7.52 - 7.48 (m, 1H), 7.43 (d, J = 9.6 Hz, 1H), 7.34 (d, J = 1.6 Hz, 1H), 6.25 - 6.22 (m, 1H), 6.20 (d, J = 10.0 Hz, 1H), 3.69 (s, 1H), 2.75 - 2.67 (m, 2H), 2.58 - 2.51 (m, 2H), 2.09 - 1.99 (m, 2H), 1.54 (s, 3H); ^{13}C NMR (101 MHz, $CDCl_3$) δ = 205.3, 146.3, 143.3, 141.4, 136.7, 128.3, 127.8, 127.4, 126.7, 125.6, 122.7, 77.2, 33.5, 33.3, 33.2, 23.4. HRMS (ESI-TOF) calcd for $C_{16}H_{16}O_2Na^+$ ([M+Na$^+$]) = 263.1043, Found 263.1039.

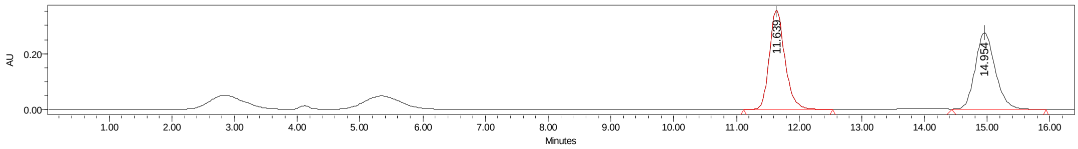

	Retention Time	Area	% Area
1	11.639	6085407	50.11
2	14.954	6059655	49.89

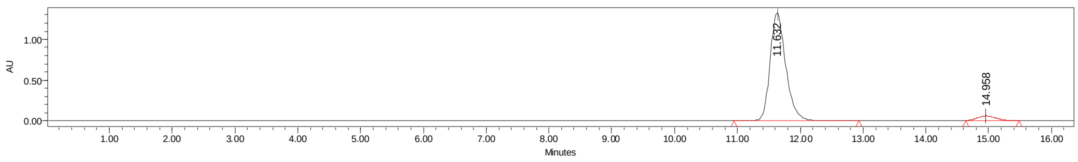

	Retention Time	Area	% Area
1	11.632	23003935	94.85
2	14.958	1249869	5.15

(*R*)-1-hydroxy-1-methyl-6-(phenylethynyl)naphthalen-2(1H)-one (3p)

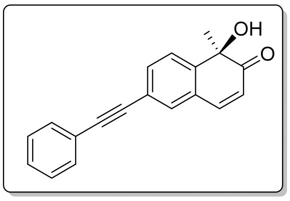

Brown oil; 99% yield, 92:8 er; $[\alpha]^{16}_{D}$ = 23.84 (c = 0.34 in CH_2Cl_2).

HPLC DAICEL CHIRALCEL IC, n-hexane/2-propanol = 95/5, flow rate = 1.0 mL/min, λ = 254 nm, t_R (major) = 19.32 min, t_R (minor) = 20.50 min. ^{1}H NMR (400 MHz, $CDCl_3$) δ = 7.71 (d, J = 8.0 Hz, 1H), 7.63 - 7.53 (m, 3H), 7.50 - 7.45 (m, 1H), 7.45 - 7.31 (m, 4H), 6.25 (d, J = 10.0 Hz, 1H), 3.73 (s, 1H), 1.56 (s, 3H); ^{13}C NMR (101 MHz, $CDCl_3$) δ = 204.8, 145.3, 144.8, 133.6, 132.4, 131.8, 128.7, 128.6, 128.6, 125.8, 123.3, 123.2, 122.9, 90.4, 88.3, 77.2, 33.2. HRMS (ESI-TOF) calcd for $C_{19}H_{14}O_2Na^+$ ([M+Na$^+$]) = 297.0886, Found 297.0887.

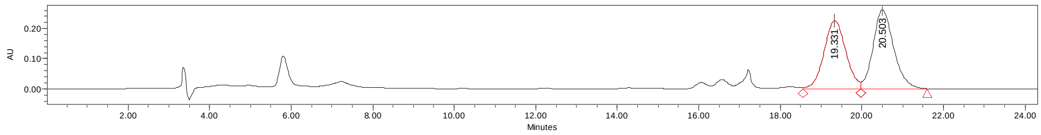

	Retention Time	Area	% Area
1	19.331	8350795	47.68
2	20.503	9162875	52.32

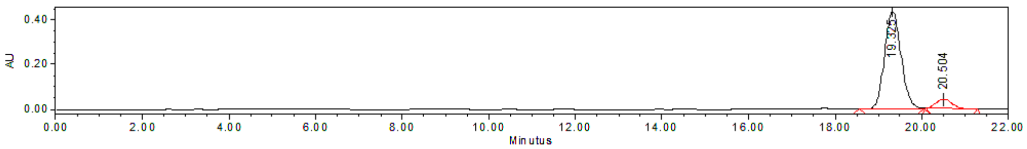

	Retention Time	Area	% Area
1	19.325	11536519	91.90
2	20.504	1016463	8.10

(*R*)-1-hydroxy-6-(3-hydroxyprop-1-yn-1-yl)-1-methylnaphthalen-2(1H)-one (3q)

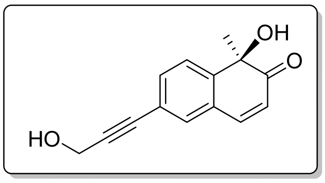

White solid; m.p. 62-64 °C; 66% yield, 91:9 er; $[\alpha]^{16}_{D}$ = 90.94 (c = 0.27 in CH_2Cl_2).

HPLC DAICEL CHIRALCEL IA, n-hexane/2-propanol = 95/5, flow rate = 1.0 mL/min, λ = 254 nm, t_R (major) = 28.80 min, t_R (minor) = 33.92 min. ^{1}H NMR (400 MHz, $CDCl_3$) δ = 7.66 (d, J = 8.0 Hz, 1H), 7.48 (d, J = 8.0 Hz, 1H), 7.40 - 7.33 (m, 2H), 6.23 (d, J = 10.0 Hz, 1H), 4.50 (s, 2H), 3.73 (s, 1H), 1.53 (s, 3H); ^{13}C NMR (101 MHz, $CDCl_3$) δ = 204.7, 145.1, 133.7, 132.4, 128.6, 125.8, 123.3, 122.5, 88.3, 84.6, 77.2, 51.7, 33.1. HRMS (ESI-TOF) calcd for $C_{14}H_{12}O_3Na^+$ ([M+Na$^+$]) = 251.0679, Found 251.0679.

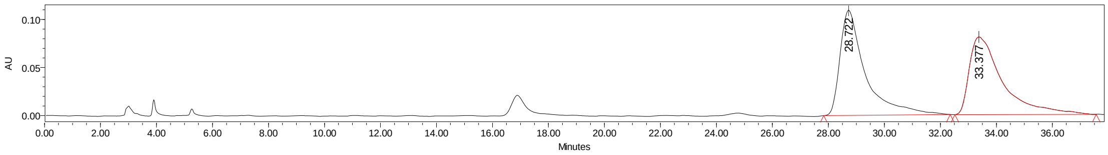

	Retention Time	Area	% Area
1	28.722	6847239	50.76
2	33.377	6643174	49.24

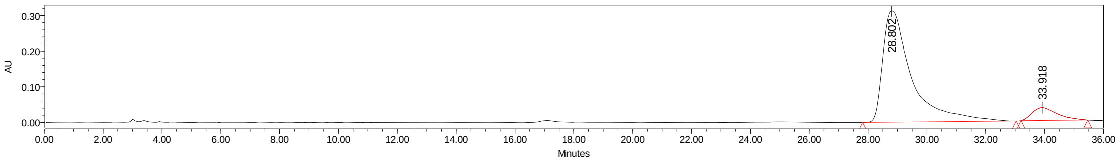

	Retention Time	Area	% Area
1	28.802	21249718	90.89
2	33.918	2130104	9.11

(*R*)-1-hydroxy-1,6-dimethylnaphthalen-2(1H)-one (3r)

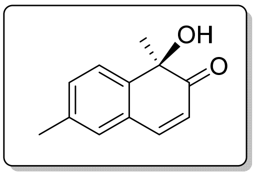

White solid; m.p. 54-56 °C; 99% yield, 94:6 er; $[\alpha]^{25}_{D}$ = 245.14 (c = 0.21 in CH_2Cl_2).

SFC Phenomenex CHIRALCEL Lux 5u Cellulose-1, CO_2/Methol = 97/3, flow rate = 1.0 mL/min, λ = 254 nm, t_R (minor) = 10.92 min, t_R (major) = 11.81 min. ^{1}H NMR (400 MHz, $CDCl_3$) δ = 7.59 (d, J = 7.6 Hz 1H), 7.39 (d, J = 10.0 Hz 1H), 7.28 - 7.23 (m, 1H), 7.11 (s, 1H), 6.18 (d, J = 10.0 Hz, 1H), 3.68 (s, 1H), 2.37 (s, 3H), 1.53 (s, 3H). ^{13}C NMR (101 MHz, $CDCl_3$) δ = 205.6, 146.3, 142.2, 137.7, 131.5, 130.2, 128.3, 125.6, 122.5, 77.1, 33.3, 21.1. HRMS (ESI-TOF) calcd for $C_{12}H_{12}O_2Na^+$ ([M+Na$^+$]) = 211.0730, Found 211.0730.

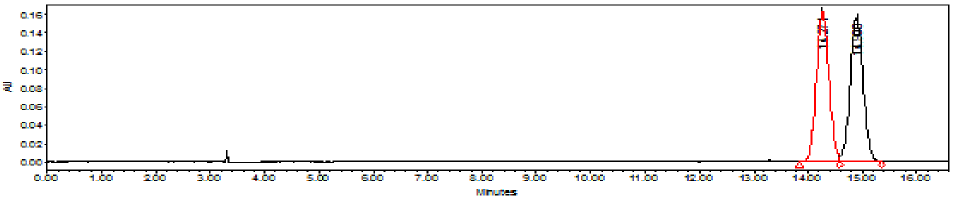

	Retention Time	Area	% Area
1	14.271	2601128	49.92
2	14.900	2609500	50.08

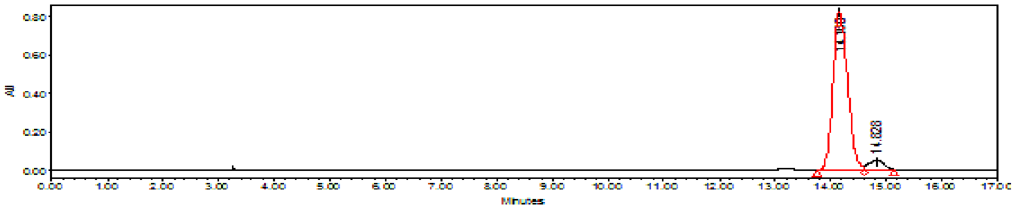

	Retention Time	Area	% Area
1	14.160	15018997	94.07
2	14.828	947460	5.93

(*R*)-6-ethyl-1-hydroxy-1-methylnaphthalen-2(1H)-one (3s)

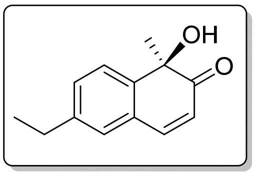

Colorless oil; 99% yield, 94:6 er; $[\alpha]^{25}_{D}$ = 226.64 (c = 0.27 in CH_2Cl_2).

HPLC DAICEL CHIRALCEL IC, n-hexane/2-propanol = 90/10, flow rate = 1.0 mL/min, λ = 254 nm, t_R (major) = 14.76 min, t_R (minor) = 18.02 min. ^{1}H NMR (400 MHz, $CDCl_3$) δ = 7.62 (d, J = 7.6 Hz, 1H), 7.41 (d, J = 10.0 Hz, 1H), 7.31 - 7.26 (m, 1H), 7.13 (s, 1H), 6.18 (d, J = 10.0 Hz, 1H), 3.69 (s, 1H), 2.67 (q, J = 7.6 Hz, 2H), 1.54 (s, 3H), 1.25 (t, J = 7.6 Hz, 3H); ^{13}C NMR (101 MHz, $CDCl_3$) δ = 205.6, 146.4, 144.1, 142.5, 130.4, 129.0, 128.4, 125.7, 122.4, 77.2, 33.3, 28.5, 15.6. HRMS (ESI-TOF) calcd for $C_{13}H_{14}O_2Na^+$ ([M+Na$^+$]) = 225.0886, Found 225.0891.

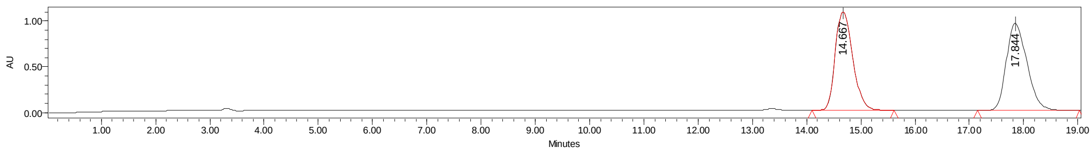

	Retention Time	Area	% Area
1	14.667	24147643	48.98
2	17.844	25150812	51.02

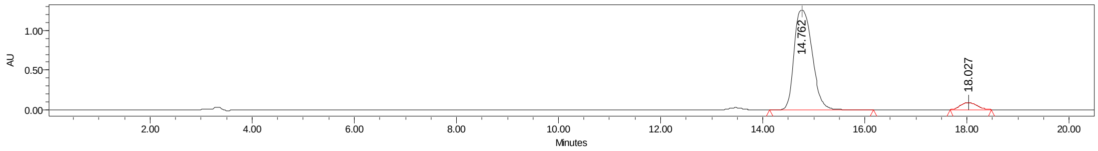

	Retention Time	Area	% Area
1	14.762	30630052	94.04
2	18.027	1942747	5.96

(*R*)-1-hydroxy-1-methyl-6-pentylnaphthalen-2(1H)-one (3t)

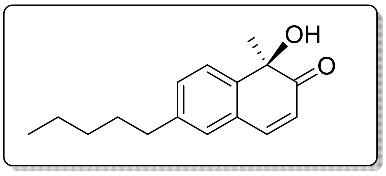

Colorless oil; 99% yield, 94:6 er; $[\alpha]^{25}_D$ = 168.94 (c = 0.45 in CH_2Cl_2).

HPLC DAICEL CHIRALCEL IC, n-hexane/2-propanol = 80/20, flow rate = 1.0 mL/min, λ = 254 nm, t_R (major) = 8.82 min, t_R (minor) = 10.44 min. ^{1}H NMR (400 MHz, $CDCl_3$) δ = 7.64 - 7.56 (m, 1H), 7.44 - 7.38 (m, 1H), 7.28 - 7.24 (m, 1H), 7.13 - 7.08 (m, 1H), 6.18 (m, d, J = 10.0 Hz, 2H), 3.68 (s, 1H), 2.61 (t, J = 7.6 Hz, 2H), 1.68 - 1.56 (m, 2H), 1.55 - 1.52 (m, 3H),

1.39 - 1.26 (m, 4H), 0.92 - 0.85 (m, 3H); ^{13}C NMR (101 MHz, $CDCl_3$) δ = 205.6, 146.4, 142.8, 142.4, 130.9, 129.6, 128.3, 125.6, 122.4, 77.1, 35.5, 33.2, 31.5, 31.1, 22.6, 14.1. HRMS (ESI-TOF) calcd for $C_{16}H_{20}O_2Na^+$ ([M+Na$^+$]) = 267.1356, Found 267.1354.

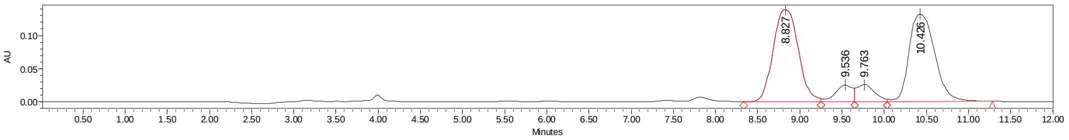

	Retention Time	Area	% Area
1	8.827	2871356	44.86
2	9.536	353367	5.52
3	9.763	377053	5.89
4	10.426	2799469	43.73

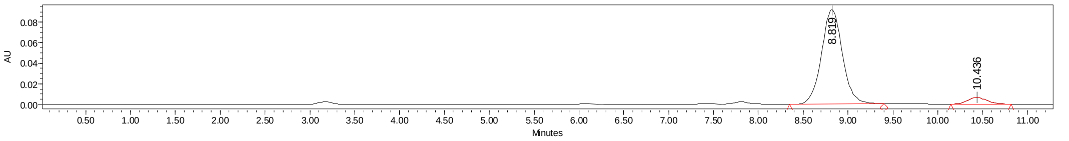

	Retention Time	Area	% Area
1	8.819	1492708	93.82
2	10.436	98368	6.18

(*R*)-1-hydroxy-6-isobutyl-1-methylnaphthalen-2(1H)-one (3u)

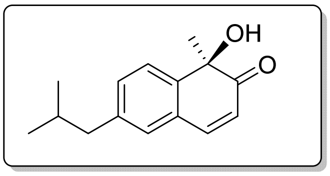

Colorless oil; 96% yield, 94.5:5.5 er; $[\alpha]^{25}_D$ = 180.57 (*c* = 0.39 in CH_2Cl_2).

HPLC DAICEL CHIRALCEL IB, n-hexane/2-propanol = 80/20, flow rate = 1.0 mL/min, λ = 254 nm, t_R (major) = 8.66 min, t_R (minor) = 9.73 min. ^1H NMR (400 MHz, $CDCl_3$) δ = 7.61 (d, *J* = 8.0 Hz, 1H), 7.40 (d, *J* = 9.6 Hz, 1H), 7.25 - 7.18 (m, 1H), 7.12 - 7.04 (m, 1H), 6.18 (d, *J* = 10.0 Hz, 1H), 3.68 (s, 1H), 2.48 (d, *J* = 7.2 Hz, 2H), 1.93 - 1.81 (m, 1H), 1.54 (s, 3H), 0.91 (d, *J* = 6.8 Hz, 6H); ^{13}C NMR (101 MHz, $CDCl_3$) δ = 205.6, 146.5, 142.5, 141.6, 131.6, 130.2, 128.2, 125.5, 122.4, 77.2, 45.0, 33.3, 30.3, 22.4. HRMS (ESI-TOF) calcd for $C_{15}H_{18}O_2Na^+$ ([M+Na$^+$]) = 253.1199, Found 253.1198.

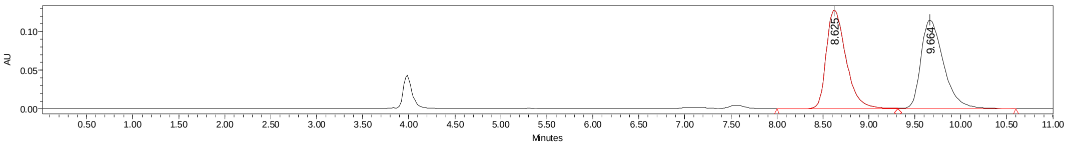

	Retention Time	Area	% Area
1	8.625	1899547	49.35
2	9.664	1949880	50.65

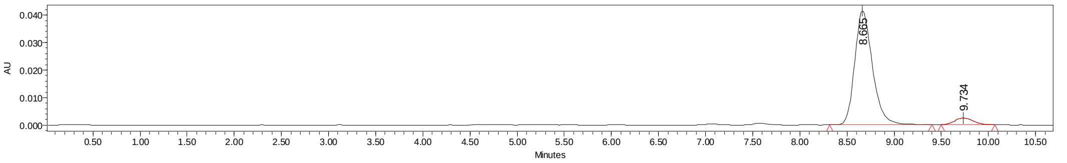

	Retention Time	Area	% Area
1	8.665	544106	94.32
2	9.734	32762	5.68

(*R*)-6-cyclopentyl-1-hydroxy-1-methylnaphthalen-2(1H)-one (3v)

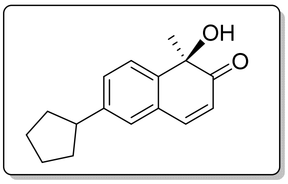

Colorless oil; 96% yield, 94.5:5.5 er; $[\alpha]^{25}_{D}$ = 146.82 (*c* = 0.44 in CH_2Cl_2).

HPLC DAICEL CHIRALCEL IC, n-hexane/2-propanol = 80/20, flow rate = 1.0 mL/min, λ = 210 nm, t_R (major) = 10.32 min, t_R (minor) = 12.56 min. ^{1}H NMR (400 MHz, $CDCl_3$) δ = 7.62 (d, *J* = 8.0 Hz, 1H), 7.41 (d, *J* = 10.0 Hz, 1H), 7.34 - 7.30 (m, 1H), 7.18 - 7.13 (m, 1H), 6.18 (d, *J* = 10.0 Hz, 1H), 3.67 (s, 1H), 3.06 - 2.95 (m, 1H), 2.14 - 2.03 (m, 2H), 1.88 - 1.77 (m, 2H), 1.76 - 1.65 (m, 2H), 1.64 - 1.52 (m, 5H); ^{13}C NMR (101 MHz, $CDCl_3$) δ = 205.6, 146.5, 146.5, 142.5, 129.6, 128.3, 128.3, 125.6, 122.4, 77.1, 45.5, 34.6, 33.2, 25.6. HRMS (ESI-TOF) calcd for $C_{16}H_{18}O_2Na^+$ ([M+Na$^+$]) = 265.1199, Found 265.1205.

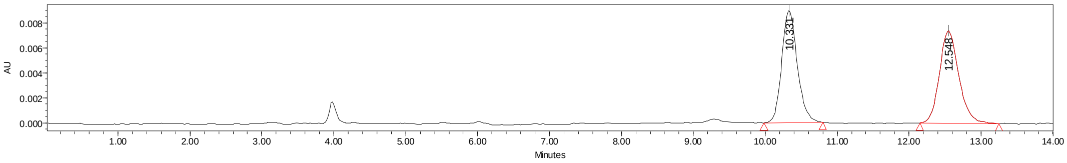

	Retention Time	Area	% Area
1	10.331	134646	49.42
2	12.548	137832	50.58

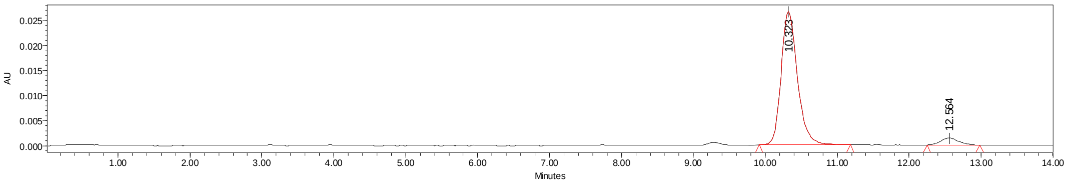

	Retention Time	Area	% Area
1	10.323	417283	94.41
2	12.564	24718	5.59

(*R*)-1-hydroxy-1-methyl-6-phenethylnaphthalen-2(1H)-one (3w)

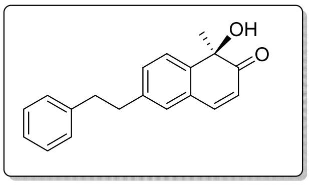

White solid; m.p. 56-58 °C; 95% yield, 95:5 er; $[\alpha]^{25}_{D}$ = 125.34 (c = 0.44 in CH_2Cl_2).

UPC[2] Phenomenex CHIRALCEL Lux 5u Cellulose-1, $scCO_2$/Methol = 90/10, flow rate = 1.0 mL/min, λ = 254 nm, t_R (major) = 22.37 min, t_R (minor) = 23.45 min. ^{1}H NMR (400 MHz, $CDCl_3$) δ = 7.62 (d, J = 6.0 Hz, 1H), 7.38 (d, J = 10.0 Hz, 1H), 7.32 - 7.24 (m, 3H), 7.23 - 7.15 (m, 3H), 7.08 (s, 1H), 6.18 (d, J = 10.0 Hz, 1H), 3.69 (s, 1H), 2.93 (s, 4H), 1.54 (s, 3H); ^{13}C NMR (101 MHz, $CDCl_3$) δ = 205.5, 146.3, 142.8, 141.6, 141.4, 131.0, 129.6, 128.6, 128.6, 128.4, 126.3, 125.7, 122.5, 77.1, 37.8, 37.5, 33.3. HRMS (ESI-TOF) calcd for $C_{19}H_{18}O_2Na^+$ ([M+Na$^+$]) = 301.1199, Found 301.1205.

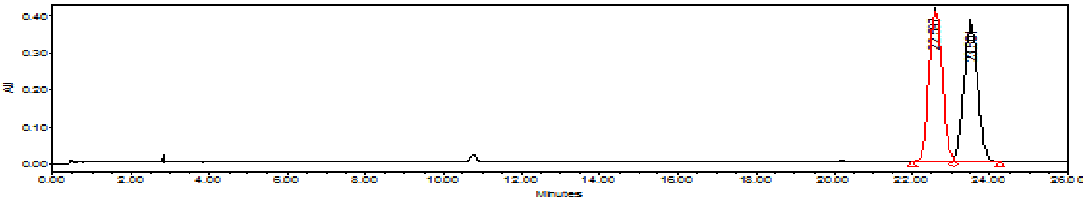

	Retention Time	Area	% Area
1	22.597	9332521	50.23
2	23.501	9247022	49.77

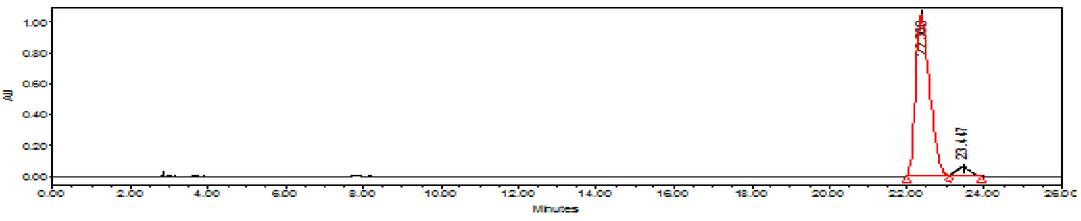

	Retention Time	Area	% Area
1	22.368	26257261	94.78
2	23.447	1446971	5.22

(*R*)-1-hydroxy-7-methoxy-1-methylnaphthalen-2(1H)-one (3x)

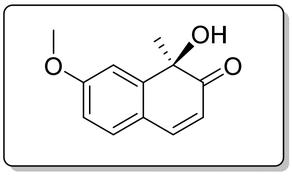

White solid; m.p. 42-43 °C; 99% yield, 94.5:5.5 er; $[\alpha]^{25}_{D}$ = 319.50 (c = 0.40 in CH_2Cl_2).

HPLC DAICEL CHIRALCEL IC, n-hexane/2-propanol = 90/10, flow rate = 1.0 mL/min, λ = 254 nm, t_R (major) = 20.65 min t_R (minor) = 25.31 min. ^{1}H NMR (400 MHz, $CDCl_3$) δ = 7.39 (d, J = 10.0 Hz, 1H), 7.28 - 7.25 (m, 1H), 7.23 (d, J = 8.4 Hz, 1H), 6.82 (dd, J = 8.8 Hz, 2.4 Hz, 1H), 6.07 (d, J = 9.6 Hz, 1H), 3.88 (s, 3H), 3.84 - 3.79 (m, 1H), 1.54 (s, 3H); ^{13}C NMR (101 MHz, $CDCl_3$) δ = 205.4, 162.0, 147.8, 146.1, 131.3, 121.6, 119.7, 113.6, 111.2, 77.4, 55.7, 33.7. HRMS (ESI-TOF) calcd for $C_{12}H_{12}O_3Na^+$ ([M+Na$^+$]) = 227.0679, Found 227.0678.

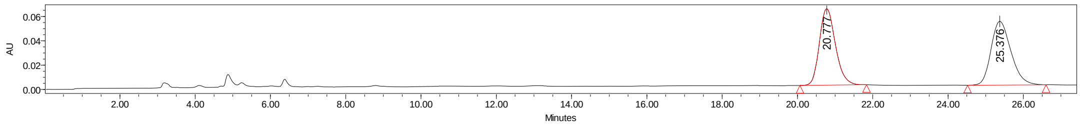

	Retention Time	Area	% Area
1	20.777	1856593	49.14
2	25.376	1921465	50.86

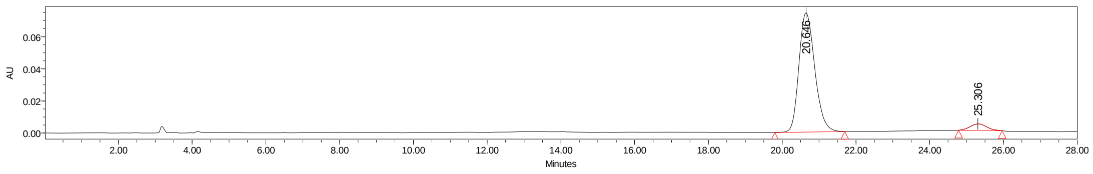

	Retention Time	Area	% Area
1	20.646	2219533	94.46
2	25.306	130079	5.54

(*R*)-1-hydroxy-7-methoxy-1,6-dimethylnaphthalen-2(1H)-one (3y)

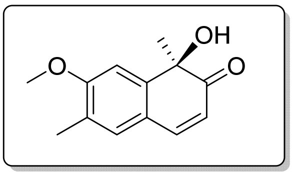

Yellow oil; 99% yield, 95:5 er; $[\alpha]^{16}_{D}$ = 348.98 (*c* = 0.40 in CH_2Cl_2).

HPLC DAICEL CHIRALCEL IA, n-hexane/2-propanol = 90/10, flow rate = 1.0 mL/min, λ = 254 nm, t_R (major) = 7.44 min, t_R (minor) = 8.16 min. ^{1}H NMR (400 MHz, $CDCl_3$) δ = 7.35 (d, *J* = 10.0 Hz, 1H), 7.18 (s, 1H), 7.06 (s, 1H), 6.05 (d, *J* = 9.6 Hz, 1H), 3.92 (s, 3H), 3.83 - 3.73 (m, 1H), 2.20 (s, 3H), 1.54 (s, 3H); ^{13}C NMR (101 MHz, $CDCl_3$) δ = 205.8, 160.2, 146.4, 145.4, 132.0, 126.0, 120.9, 119.5, 107.4, 77.4, 55.8, 55.8, 33.8, 15.9. HRMS (ESI-TOF) calcd for $C_{13}H_{14}O_3Na^+$ ($[M+Na^+]$) = 241.0835, Found 241.0838.

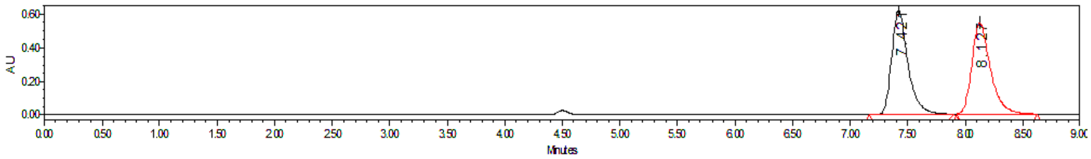

	Retention Time	Area	% Area
1	7.421	6202438	50.52
2	8.127	6075532	49.48

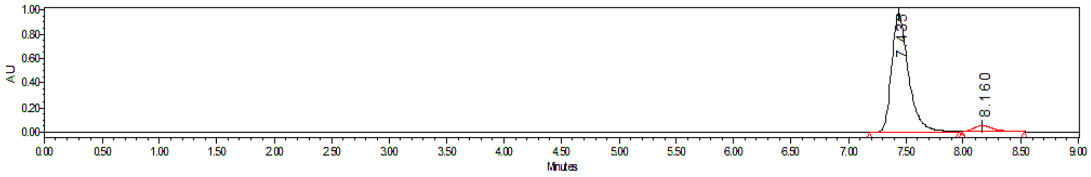

	Retention Time	Area	% Area
1	7.435	9925051	94.75
2	8.160	550175	5.25

(*R*)-5-ethyl-1-hydroxy-6-(methoxymethoxy)-1-methylnaphthalen-2(1H)-one (3z)

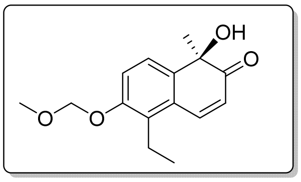

Yellow solid; m.p. 82-84 °C; 99% yield, 95:5 er; $[\alpha]^{16}_{D}$ = 126.92 (c = 0.66 in CH_2Cl_2).

HPLC DAICEL CHIRALCEL IA, n-hexane/2-propanol = 90/10, flow rate = 1.0 mL/min, λ = 254 nm, t_R (major) = 7.43 min, t_R (minor) = 8.89 min. ^{1}H NMR (400 MHz, $CDCl_3$) δ = 7.75 (d, J = 10.4 Hz, 1H), 7.49 (d, J = 8.4 Hz, 1H), 7.16 (d, J = 8.4 Hz, 1H), 6.22 (d, J = 10.0 Hz, 4H), 5.27 - 5.17 (m, 2H), 3.66 (s, 1H), 3.49 (s, 3H), 2.94 - 2.78 (m, 2H), 1.53 (s, 3H), 1.16 (t, J = 7.6 Hz, 3H); ^{13}C NMR (101 MHz, $CDCl_3$) δ = 205.5, 154.4, 142.0, 132.8, 124.0, 122.6, 116.0, 94.6, 77.1, 56.2, 33.5, 18.7, 15.6. HRMS (ESI-TOF) calcd for $C_{15}H_{18}O_4Na^+$ ($[M+Na^+]$) = 285.1097, Found 285.1107.

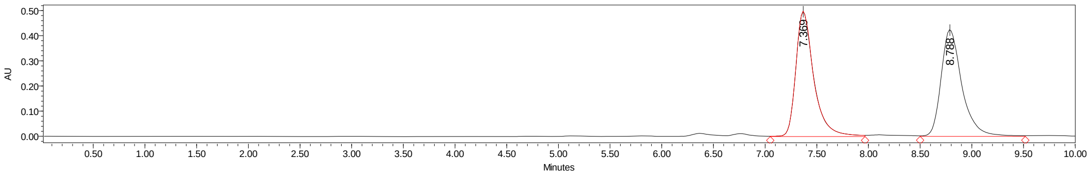

	Retention Time	Area	% Area
1	7.369	6012927	50.27
2	8.788	5948623	49.73

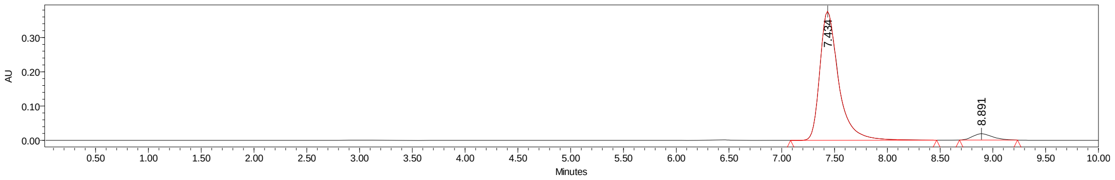

	Retention Time	Area	% Area
1	7.434	4632099	95.03
2	8.891	242466	4.97

(*R*)-1-hydroxy-1,3-dimethylnaphthalen-2(1H)-one (3aa)

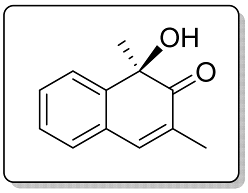

Colorless oil; 99% yield, 83.5:16.5 er; $[\alpha]^{19}_{D}$ = 289.62 (c = 0.21 in CH_2Cl_2).

UPC[2] Phenomenex CHIRALCEL Lux 5u Cellulose-3, $scCO_2$/Methol = 95/5, flow rate = 1.0 mL/min, λ = 254 nm, t_R (major) = 6.36 min, t_R (minor) = 7.03 min. ^{1}H NMR (400 MHz, $CDCl_3$) δ = 7.66 (d, *J* = 7.6 Hz, 1H), 7.40 - 7.34 (m, 1H), 7.32 - 7.26 (m, 1H), 7.24 - 7.17 (m, 2H), 3.73 (s, 1H), 2.04 (d, *J* = 1.6 Hz, 3H), 1.52 (s, 3H); ^{13}C NMR (101 MHz, $CDCl_3$) δ = 206.0, 144.2, 142.2, 130.5, 129.6, 128.7, 128.6, 127.9, 125.4, 77.1, 33.4, 15.5. HRMS (ESI-TOF) calcd for $C_{12}H_{12}O_2Na^+$ ([M+Na$^+$]) = 211.0730, Found 211.0733.

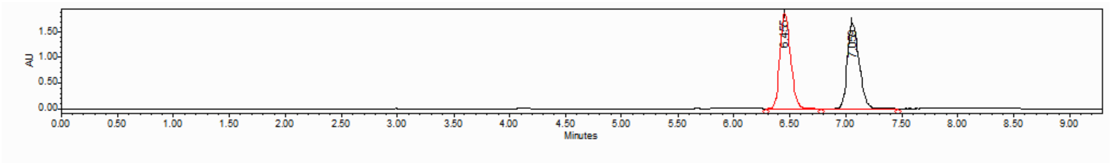

	Retention Time	Area	% Area
1	6.455	12197643	50.14
2	7.059	12130866	49.86

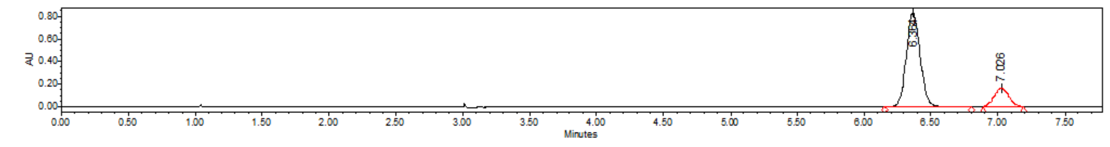

	Retention Time	Area	% Area
1	6.364	5965937	83.60
2	7.026	1170699	16.40

(*R*)-1-ethyl-1-hydroxynaphthalen-2(1H)-one (3ab)

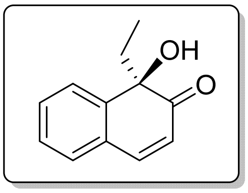

Colorless oil; 64% for total yield of **3ab** and **4ab**, **3ab**/**4ab** = 85:15 by ^{1}H NMR. 75:25 er/78:22 er.

HPLC DAICEL CHIRALCEL IE, n-hexane/2-propanol = 90/10, flow rate = 1.0 mL/min, λ = 254 nm, t_R (minor) = 12.81 min, t_R (major) = 14.30 min. ^{1}H NMR (400 MHz, $CDCl_3$) δ = 7.65 (d, *J* = 7.6 Hz, 1H), 7.46 - 7.37 (m, 2H), 7.34 - 7.24 (m, 2H), 6.17 (d, *J* = 9.6 Hz, 1H), 3.74 (s, 1H), 1.92 - 1.70 (m, 2H), 0.82 (t, *J* = 7.6 Hz, 3H); ^{13}C NMR (101 MHz, $CDCl_3$) δ = 205.6, 146.0,

144.5 130.3, 129.4, 129.1 127.9, 126.2, 122.9, 80.3, 38.7, 8.2. HRMS (ESI-TOF) calcd for $C_{12}H_{12}O_2Na^+$ ($[M+Na^+]$) = 211.0730, Found 211.0735.

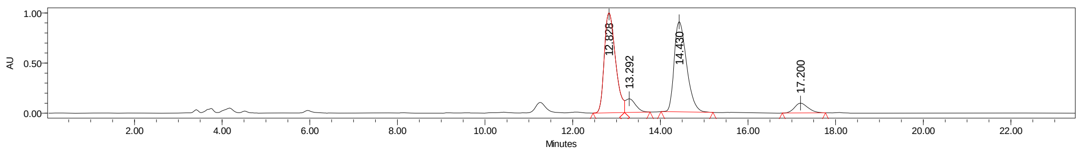

	Retention Time	Area	% Area
1	12.828	17644988	44.52
2	13.292	2195792	5.54
3	14.430	17700654	44.66
4	17.200	2092157	5.28

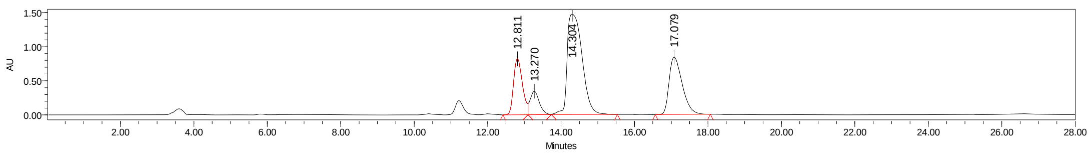

	Retention Time	Area	% Area
1	12.811	13769071	16.75
2	13.270	5985305	7.28
3	14.304	41585000	50.58
4	17.079	20869517	25.39

(*S*)-2-hydroxy-2-methylnaphthalen-1(2H)-one (3ad)

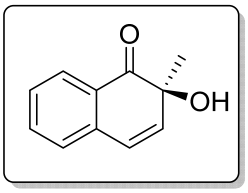

Colorless oil; 46% yield, 77:23 er. $[\alpha]^{19}_D$ = 142.27 (c = 0.19 in CH_2Cl_2).

HPLC DAICEL CHIRALCEL IE, n-hexane/2-propanol = 90/10, flow rate = 1.0 mL/min, λ = 254 nm, t_R (major) = 15.53 min, t_R (minor) = 20.17 min. ^{1}H NMR (400 MHz, $CDCl_3$) δ = 7.98 (d, J = 8.0 Hz, 1H), 7.59 (t, J = 7.6 Hz, 1H), 7.36 (t, J = 7.6 Hz, 1H), 7.22 (d, J = 7.6 Hz, 1H), 6.51 - 6.44 (m, 1H), 6.37 - 6.29 (m, 1H), 3.40 (s, 1H), 1.45 (s, 3H); ^{13}C NMR (101 MHz, $CDCl_3$) δ = 204.4, 138.0, 137.1, 135.3, 128.3, 128.3, 127.6, 127.4, 124.4, 75.5, 28.7. HRMS

(ESI-TOF) calcd for $C_{11}H_{10}O_2Na^+$ ($[M+Na^+]$) = 197.0573, Found 197.0574.

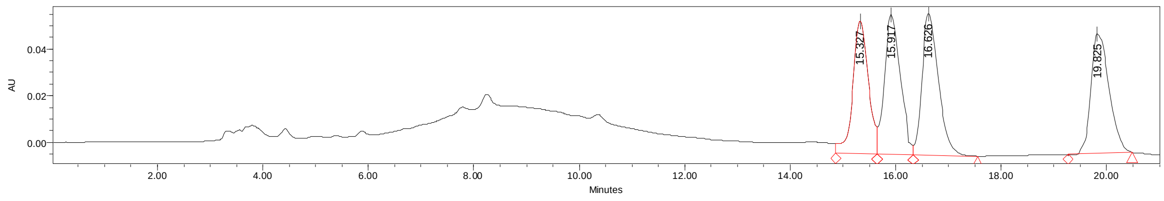

	Retention Time	Area	% Area
1	15.327	1171769	23.50
2	15.917	1270613	25.48
3	16.626	1316241	26.40
4	19.825	1227859	24.62

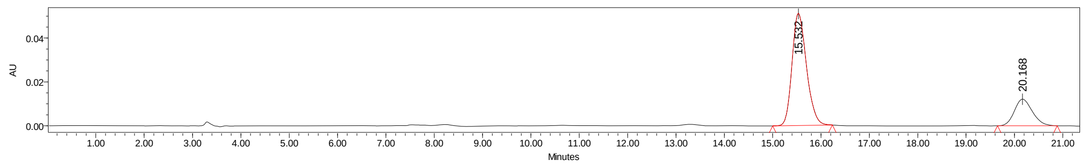

	Retention Time	Area	% Area
1	15.532	1058324	77.12
2	20.168	313993	22.88

(*R*)-6-((tert-butyldimethylsilyl)oxy)-5-ethyl-1-hydroxy-1-methylnaphthalen-2(1H)-one (3ae)

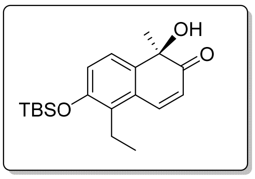

White solid; m.p. 70-72 °C; 99% yield, 95:5 er; $[\alpha]^{19}_{D}$ = 86.56 (*c* = 0.45 in CH_2Cl_2).

HPLC DAICEL CHIRALCEL IA, n-hexane/2-propanol = 90/10, flow rate = 1.0 mL/min, λ = 254 nm, t_R (major) = 4.46 min, t_R (minor) = 5.03 min. ^{1}H NMR (400 MHz, $CDCl_3$) δ = 7.73 (d, *J* = 10.0 Hz, 1H), 7.41 (d, *J* = 8.4 Hz, 1H), 6.86 (d, *J* = 8.4 Hz, 1H), 6.20 (d, *J* = 10.4 Hz, 1H), 3.65 (s, 1H), 2.89 - 2.75 (m, 2H), 1.53 (s, 3H), 1.13 (t, *J* = 7.6 Hz, 3H), 1.03 (s, 9H), 0.27 (s, 3H), 0.24 (s, 3H); ^{13}C NMR (101 MHz, $CDCl_3$) δ = 205.6, 153.0, 142.4, 138.3, 134.3, 127.2, 123.8, 122.4, 120.2, 77.1, 33.5, 25.8, 19.0, 18.4, 15.6, -3.9, -4.2. HRMS (ESI-TOF) calcd for $C_{19}H_{28}O_3Si$

Na$^+$ ([M+Na$^+$]) = 355.1705, Found 355.1707.

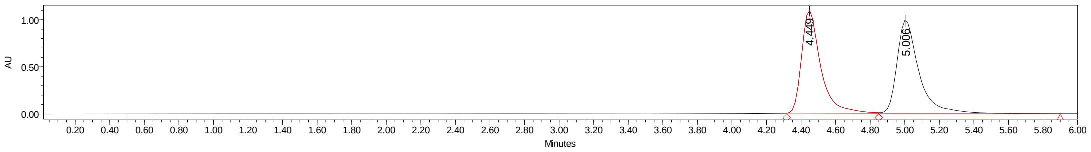

	Retention Time	Area	% Area
1	4.449	8561111	49.28
2	5.006	8812535	50.72

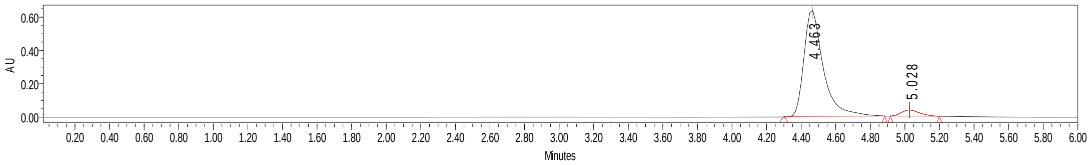

	Retention Time	Area	% Area
1	4.463	4940562	94.91
2	5.028	264748	5.09

(*R*)-5-ethyl-1,6-dihydroxy-1-methylnaphthalen-2(1H)-one (lacinilene D)

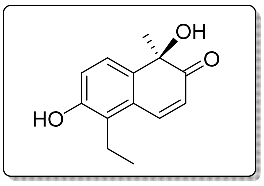

Yellow solid; m.p. 134-136 °C; 99% yield, 95:5 er; $[\alpha]^{19}_D$ = 162.11 (c = 0.45 in CH_2Cl_2).

UPC^2 Phenomenex CHIRALCEL Lux 5u Cellulose-1, $scCO_2$/Methol = 80/20, flow rate = 1.0 mL/min, λ = 254 nm, t_R (major) = 7.27 min, t_R (minor) = 7.90 min. ^{1}H NMR (400 MHz, $CDCl_3$) δ = 7.75 (d, J = 10.4 Hz, 1H), 7.41 (d, J = 8.4 Hz, 1H), 6.84 (d, J = 8.4 Hz, 1H), 6.23 (d, J = 10.4 Hz, 1H), 5.33 (s, 1H), 3.67 (s, 1H), 2.91 - 2.71 (m, 2H), 1.53 (s, 3H), 1.18 (t, J = 7.6 Hz, 3H); ^{13}C NMR (101 MHz, $CDCl_3$) δ = 205.8, 153.0, 142.2, 138.1, 129.9, 127.1, 124.0, 122.6, 117.4, 77.2, 33.5, 18.6, 15.4. HRMS (ESI-TOF) calcd for $C_{13}H_{14}O_3Na^+$ ([M+Na$^+$]) = 241.0841, Found 241.0841.

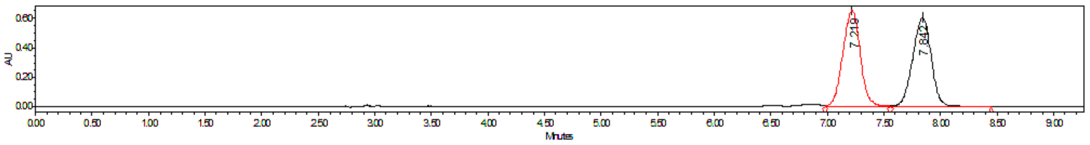

	Retention Time	Area	% Area
1	7.219	6889209	50.37
2	7.842	6787522	49.63

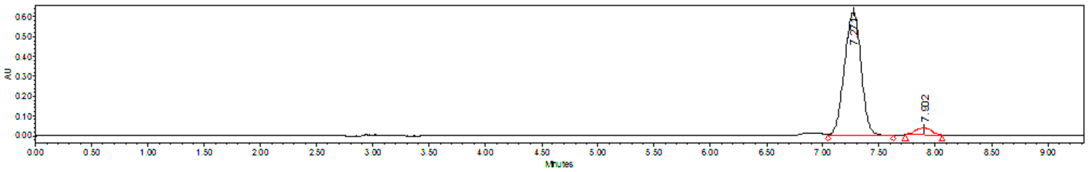

	Retention Time	Area	% Area
1	7.271	6421072	94.92
2	7.902	343676	5.08

(*S*)-6-((tert-butyldimethylsilyl)oxy)-5-ethyl-1-hydroxy-1-methylnaphthalen-2(1H)-one (3ae)

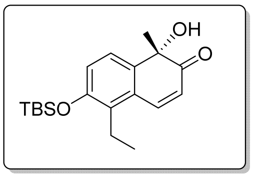

White solid; m.p. 68-70 °C; 99% yield, 95.5:4.5 er; $[\alpha]^{19}_{D}$ = -91.25 (c = 0.56 in CH_2Cl_2).

HPLC DAICEL CHIRALCEL IA, n-hexane/2-propanol = 90/10, flow rate = 1.0 mL/min, λ = 254 nm, t_R (minor) = 4.59 min, t_R (major) = 5.22 min. ^{1}H NMR (400 MHz, $CDCl_3$) δ = 7.73 (d, J = 10.0 Hz, 1H), 7.41 (d, J = 8.4 Hz, 1H), 6.86 (d, J = 8.4 Hz, 1H), 6.20 (d, J = 10.4 Hz, 1H), 3.65 (s, 1H), 2.89 - 2.75 (m, 2H), 1.53 (s, 3H), 1.13 (t, J = 7.6 Hz, 3H), 1.03 (s, 9H), 0.27 (s, 3H), 0.24 (s, 3H); ^{13}C NMR (101 MHz, $CDCl_3$) δ = 205.6, 153.0, 142.4, 138.3, 134.3, 127.2, 123.8, 122.4, 120.2, 77.1, 33.5, 25.8, 19.0, 18.4, 15.6, -3.9, -4.2. HRMS (ESI-TOF) calcd for $C_{19}H_{28}O_3SiNa^+$ ($[M+Na^+]$) = 355.1705, Found 355.1707.

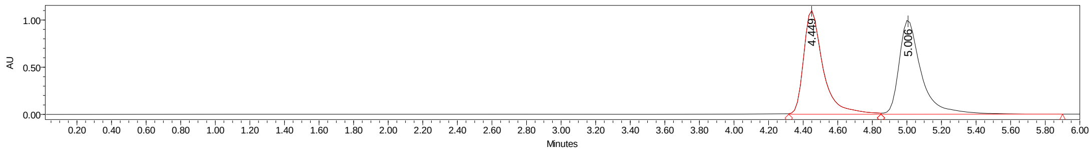

	Retention Time	Area	% Area
1	4.449	8561111	49.28
2	5.006	8812535	50.72

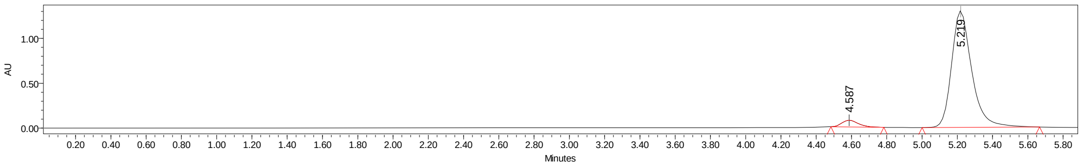

	Retention Time	Area	% Area
1	4.587	487245	4.52
2	5.219	10302329	95.48

(*S*)-5-ethyl-1,6-dihydroxy-1-methylnaphthalen-2(1H)-one (lacinilene D)

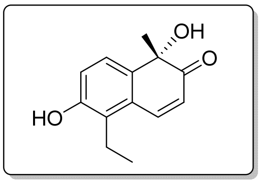

Yellow solid; m.p. 134-136 °C; 99% yield, 95:5 er; $[\alpha]^{19}_{D}$ = -177.58 (c = 0.33 in CH_2Cl_2).

UPC[2] Phenomenex CHIRALCEL Lux 5u Cellulose-1, $scCO_2$/Methol = 80/20, flow rate = 1.0 mL/min, λ = 254 nm, t_R (minor) = 7.20 min, t_R (major) = 7.79 min. ^{1}H NMR (400 MHz, $CDCl_3$) δ = 7.75 (d, J = 10.4 Hz, 1H), 7.41 (d, J = 8.4 Hz, 1H), 6.84 (d, J = 8.4 Hz, 1H), 6.23 (d, J = 10.4 Hz, 1H), 5.33 (s, 1H), 3.67 (s, 1H), 2.91 - 2.71 (m, 2H), 1.53 (s, 3H), 1.18 (t, J = 7.6 Hz, 3H); ^{13}C NMR (101 MHz, $CDCl_3$) δ = 205.8, 153.0, 142.2, 138.1, 129.9, 127.1, 124.0, 122.6, 117.4, 77.2, 33.5, 18.6, 15.4. HRMS (ESI-TOF) calcd for $C_{13}H_{14}O_3Na^+$ ([M+Na$^+$]) = 241.0841, Found 241.0841.

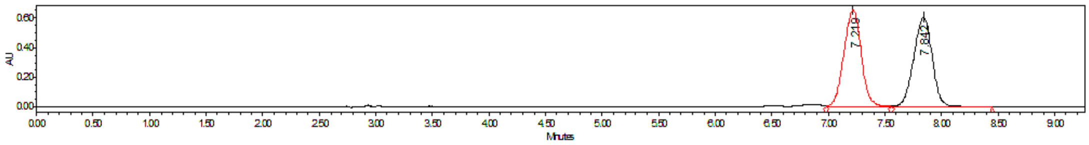

	Retention Time	Area	% Area
1	7.219	6889209	50.37
2	7.842	6787522	49.63

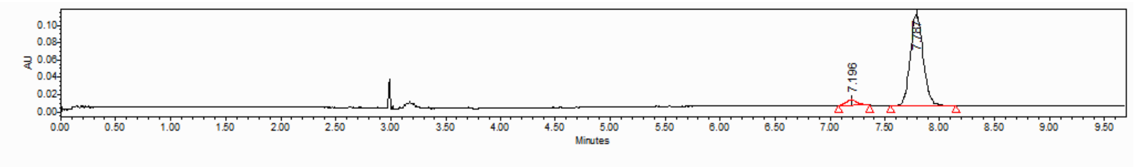

	Retention Time	Area	% Area
1	7.196	48556	5.27
2	7.787	872067	94.73

(*S*)-1-hydroxy-4-isopropyl-7-methoxy-1,6-dimethylnaphthalen-2(1H)-one (lacinilene C methyl ether)

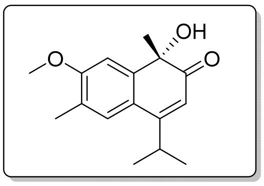

White solid; m.p. 98-100 °C; 99% yield, 83:17 er; $[\alpha]^{19}_{D}$ = -237.29 (c = 0.24 in CH_2Cl_2).

HPLC DAICEL CHIRALCEL IA, n-hexane/2-propanol = 90/10, flow rate = 1.0 mL/min, λ = 254 nm, t_R (minor) = 5.75 min, t_R (major) = 6.36 min. ^{1}H NMR (400 MHz, $CDCl_3$) δ = 7.35 (s, 1H), 7.21 (s, 1H), 6.02 (s, 1H), 3.92 (s, 3H), 3.87 (s, 1H), 3.28 - 3.15 (m, 1H), 2.24 (s, 3H), 1.53 (s, 3H), 1.27 (t, J = 6.4 Hz, 6H); ^{13}C NMR (101 MHz, $CDCl_3$) δ = 205.7, 164.4, 159.6, 145.7, 127.8, 125.5, 121.0, 114.9, 107.4, 77.0, 55.8, 34.1, 29.3, 22.4, 22.1, 16.4. HRMS (ESI-TOF) calcd for $C_{16}H_{20}O_3Na^+$ ([M+Na$^+$]) = 283.1305, Found 283.1315.

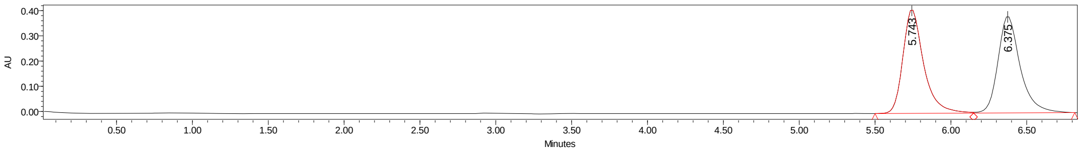

	Retention Time	Area	% Area
1	5.743	3907821	50.08
2	6.375	3894746	49.92

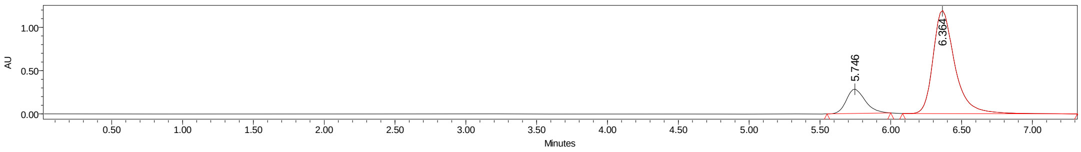

	Retention Time	Area	% Area
1	5.746	2596264	16.99
2	6.364	12682637	83.01

11. The X-ray data for 3ae

The **3ae** was recrystallized from Pet.

CCDC-1536822 (**3ae**) contain the supplementary crystallographic data for this paper. These data can be obtained free of charge from the Cambridge Crystallographic Data Centre via www.ccdc.cam.ac.uk./ data_request/cif.

12. References

[1]. T. Oguma, T. Katsuki, *J. Am. Chem. Soc.* **2012**, *134*, 20017-20020.

[2]. T. Kaku, N. Matsunaga, A. Ojida, T. Tanaka, T. Hara, M. Yamaoka, M. Kusaka, A. Tasaka, *Bioorganic & Medicinal Chemistry*. **2011**, *19*, 1751-1770.

[3]. Q. Yin, S.-G. Wang, X.-W. Liang, D.-W. Gao, J. Zheng S.-L. You, *Chem. Sci.* **2015**, *6*, 4179-4183.

[3]. C. Bosset, R. Coffinier, P. A. Peixoto, M. El Assal, K. Miqueu, J.-M. Sotiropoulos, L. Pouységu, S. Quideau, *Angew. Chem.* **2014**, *126*, 10018-10022; *Angew. Chem., Int. Ed.* **2014**, *53*, 9860-9864.

[5]. K. Krohn, G. Zimmermann, *J. Org. Chem.* **1998,** *63,* 4140-4142.

[6]. a) X. H. Liu, L. L. Lin, X. M. Feng, *Acc. Chem. Res.* **2011**, *44*, 574-587; b) X. H. Liu, L. L. Lin, X. M. Feng, *Org. Chem. Front.* **2014**, *1*, 298-302.

13. Copies of NMR spectra

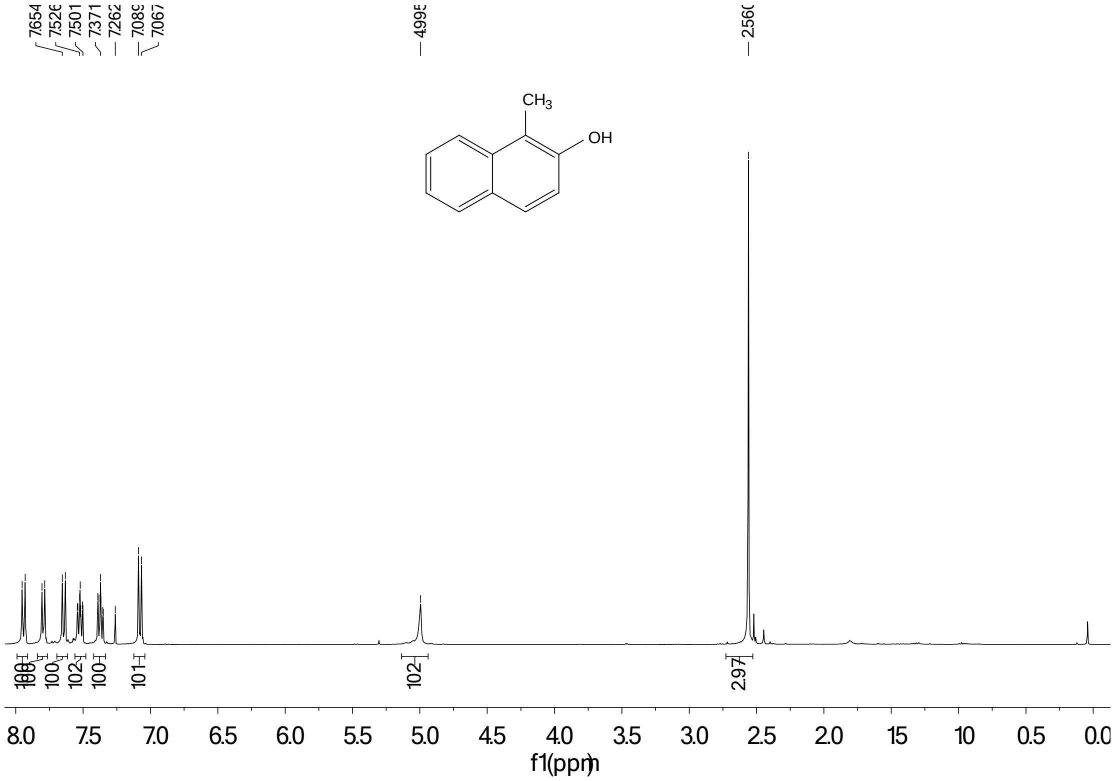

12.

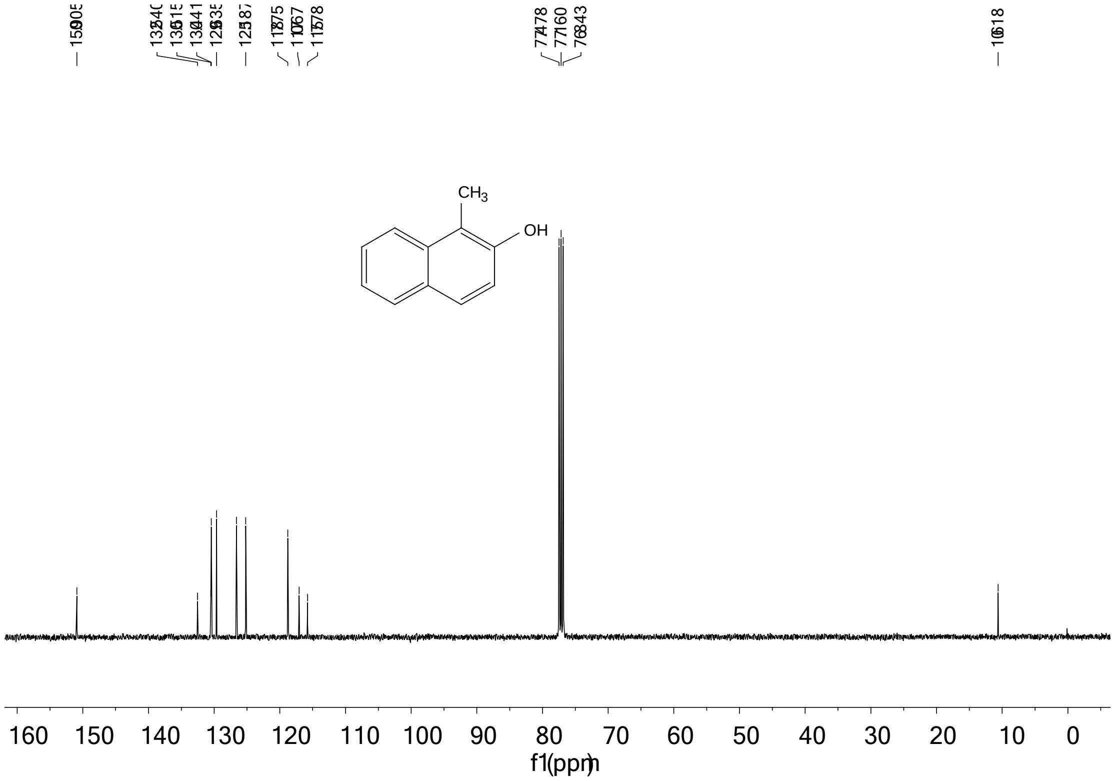

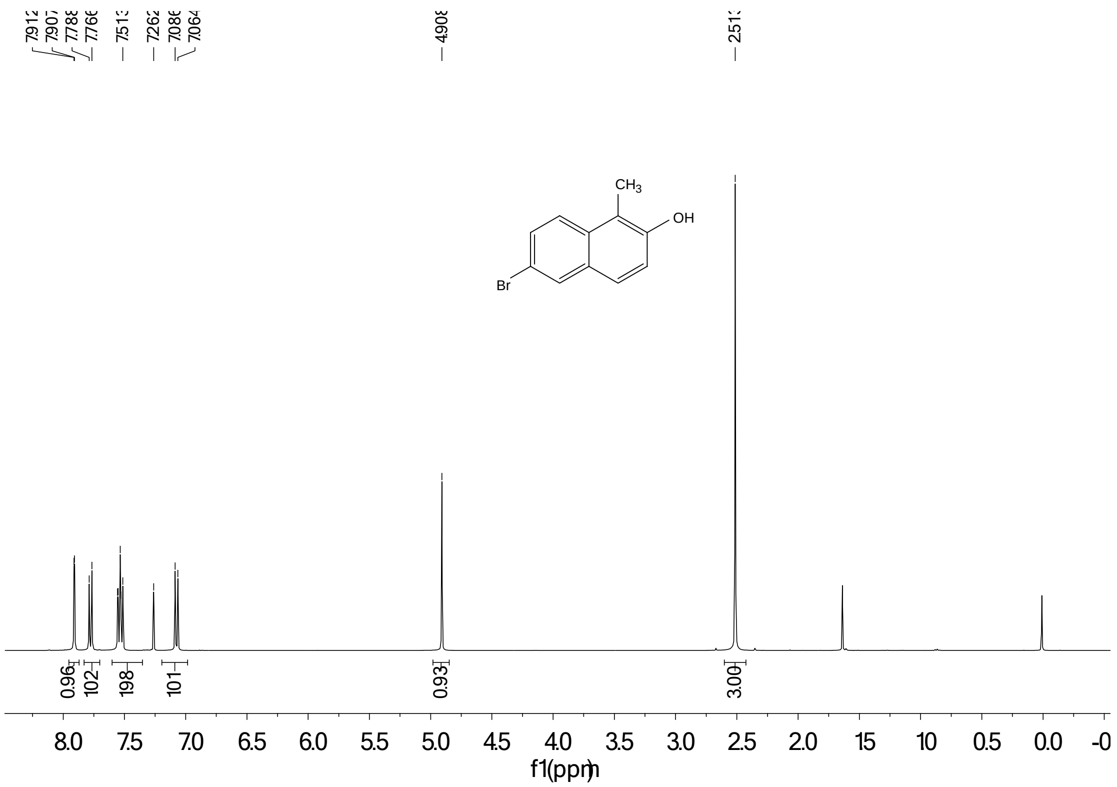
CH3
OH
Br
f1 (ppm)

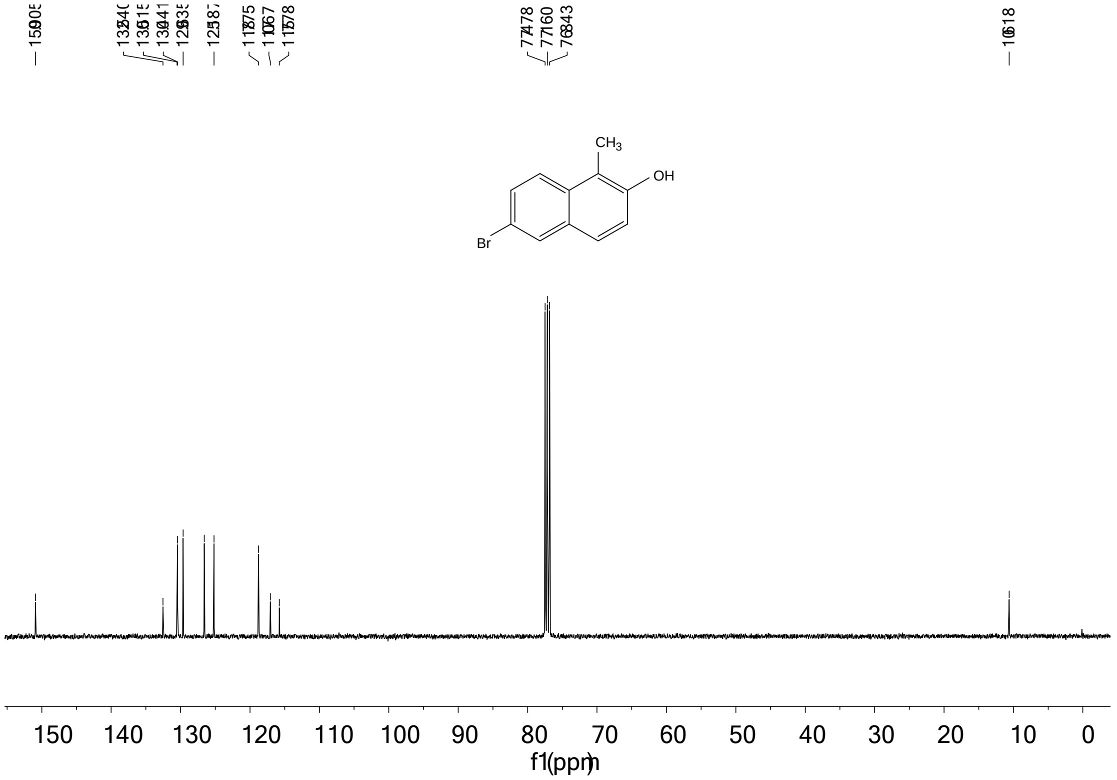
CH3
OH
Br
f1 (ppm)

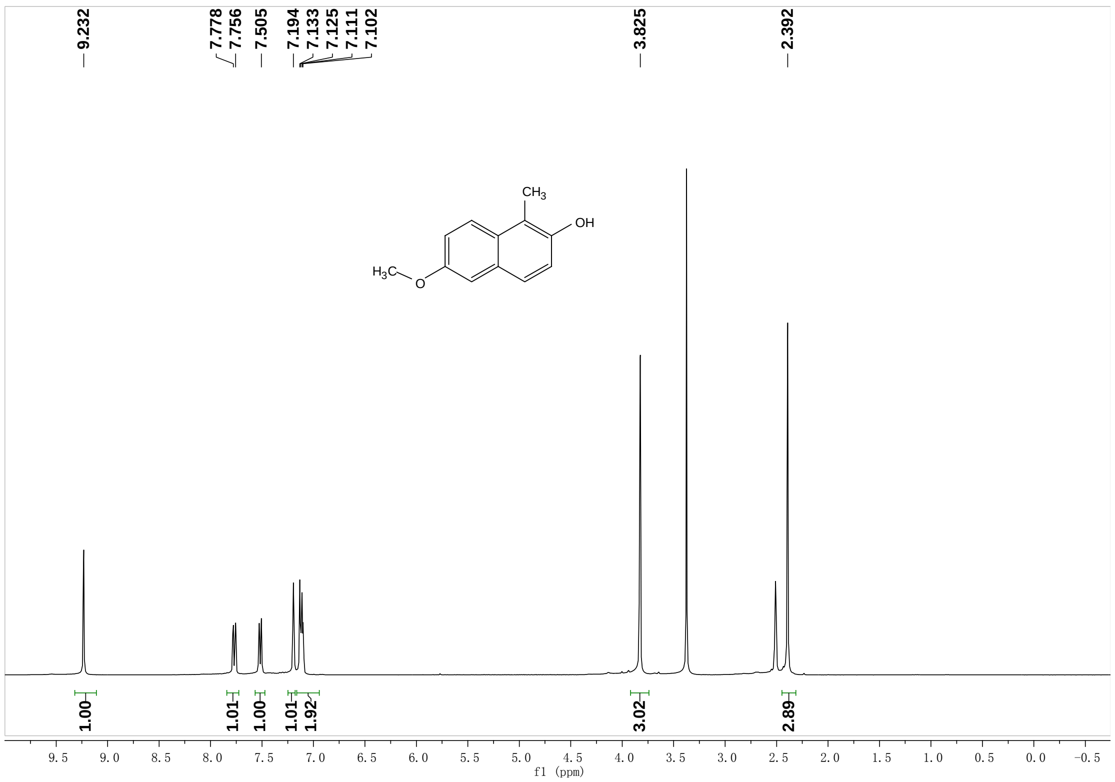

9.232
7.778
7.756
7.505
7.194
7.133
7.125
7.111
7.102
3.825
2.392
CH3
OH
H3C
O
1.00
1.01
1.00
1.01
1.92
3.02
2.89
f1 (ppm)

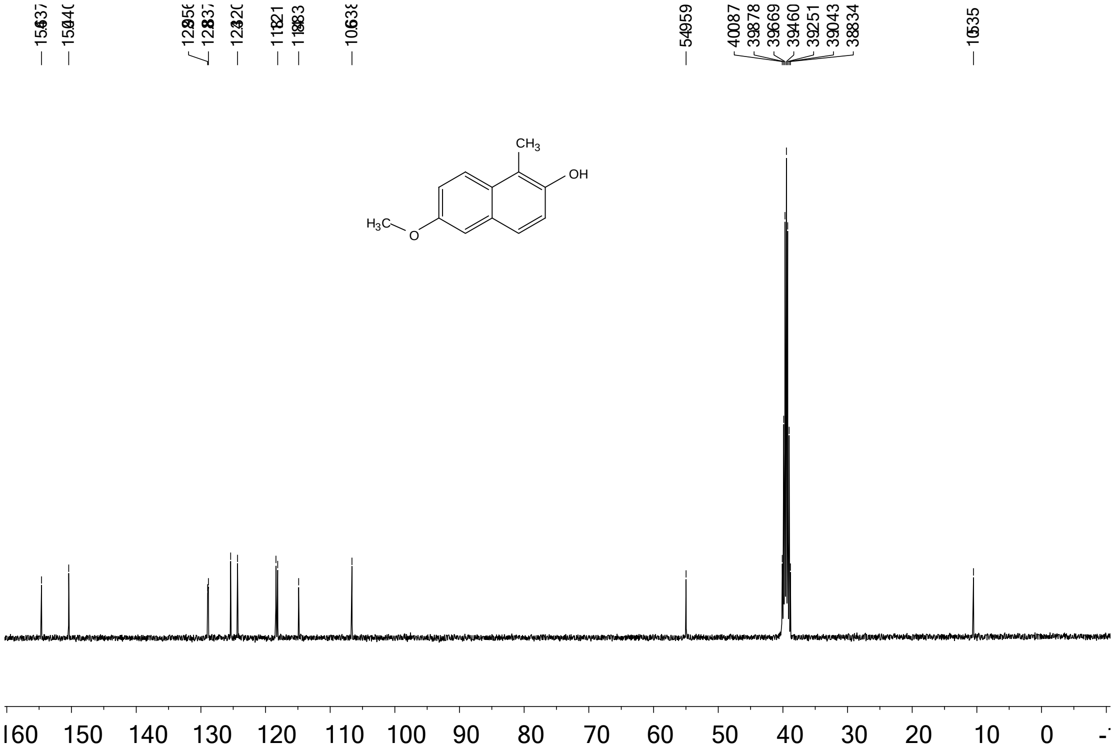

CH3
OH
H3C
O
f1 (ppm)

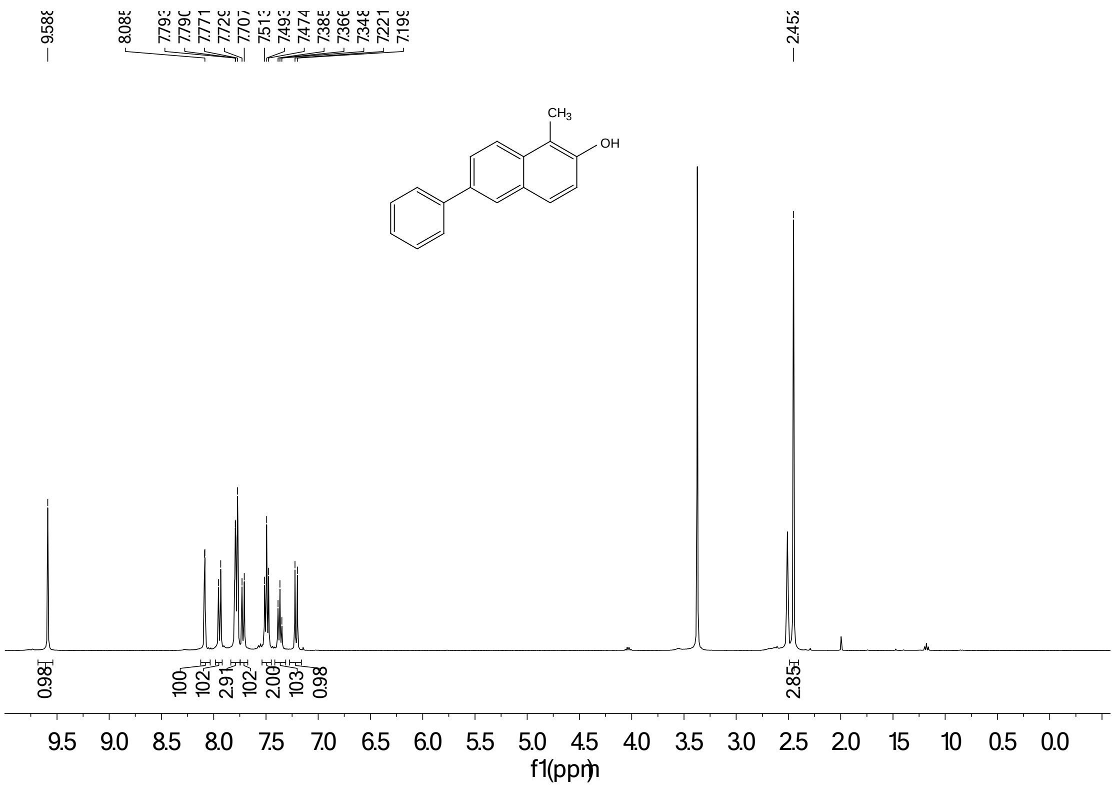
CH3
OH
f1 (ppm)

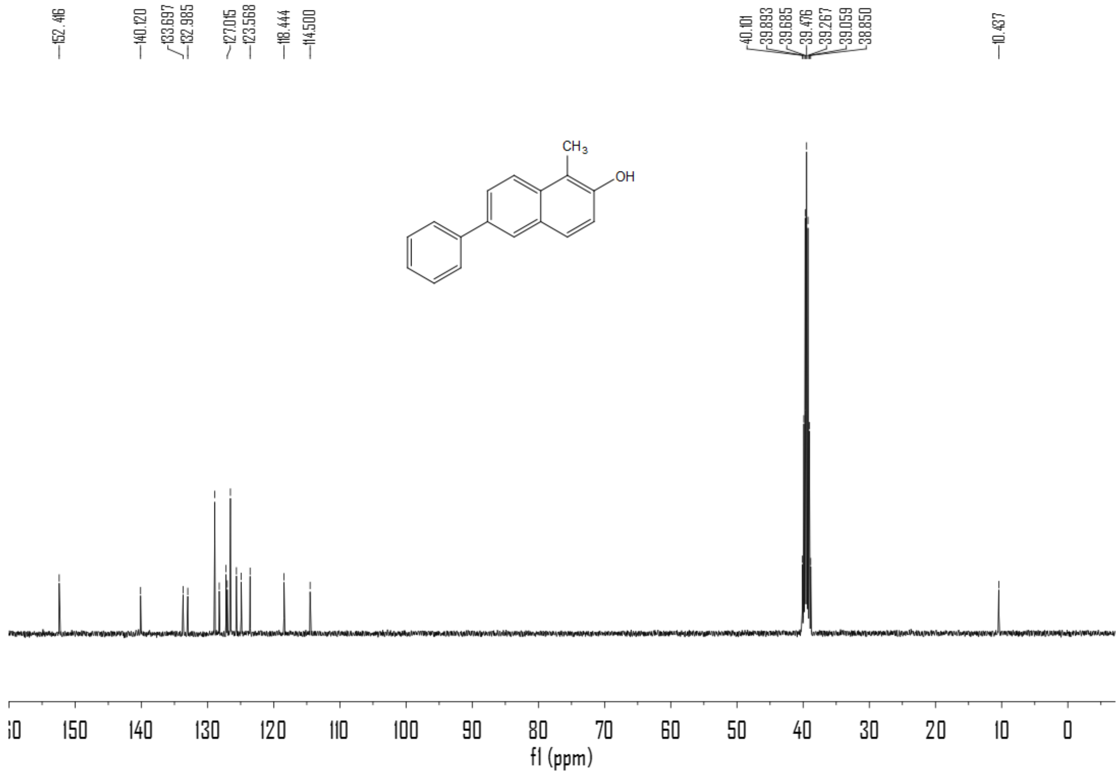
CH3
OH
f1 (ppm)

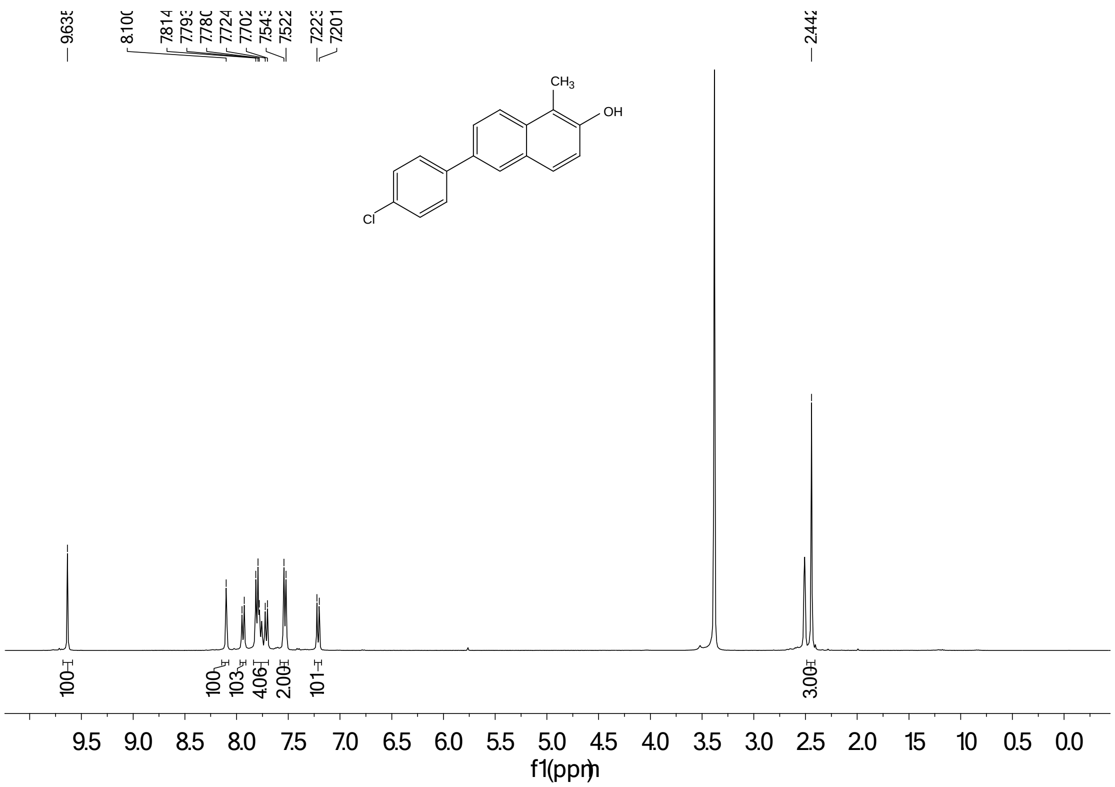
9.635
8.100
7.814
7.793
7.780
7.724
7.702
7.543
7.522
7.223
7.201
2.442
CH3
OH
Cl
1.00
1.03
4.06
2.00
1.01
3.00
9.5 9.0 8.5 8.0 7.5 7.0 6.5 6.0 5.5 5.0 4.5 4.0 3.5 3.0 2.5 2.0 1.5 1.0 0.5 0.0
f1 (ppm)

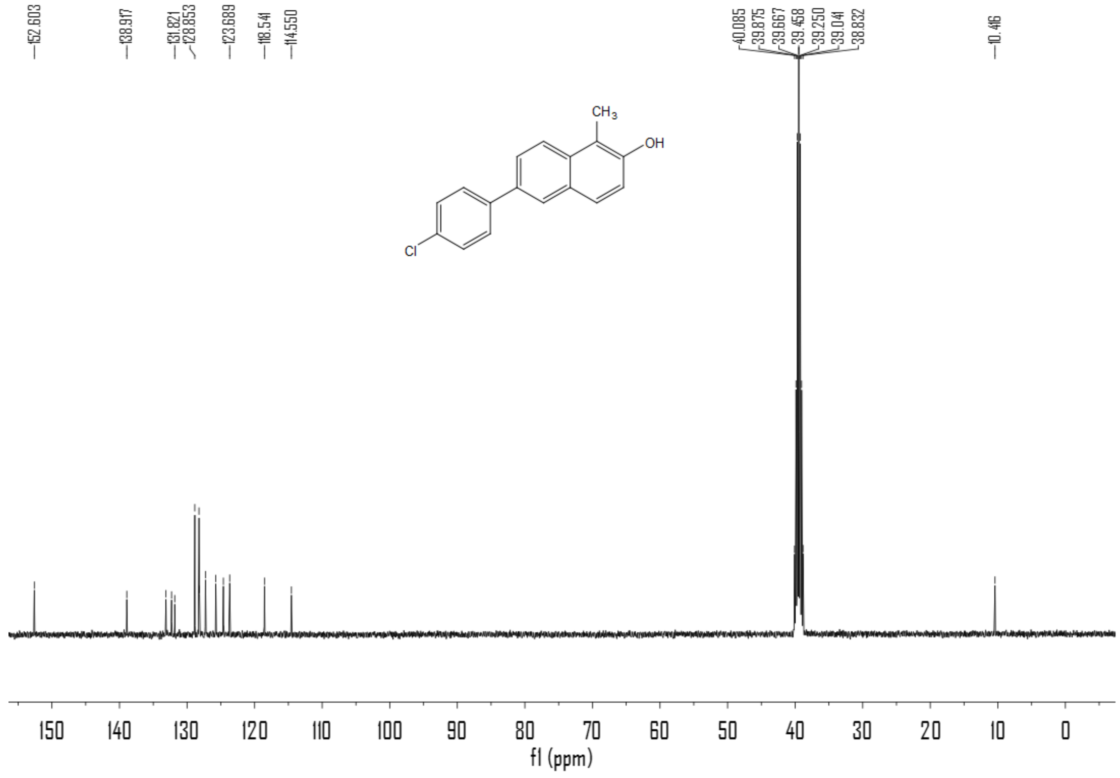
152.603
138.917
131.821
128.853
123.689
118.541
114.550
40.085
39.875
39.667
39.458
39.250
39.041
38.832
10.416
CH3
OH
Cl
150 140 130 120 110 100 90 80 70 60 50 40 30 20 10 0
f1 (ppm)

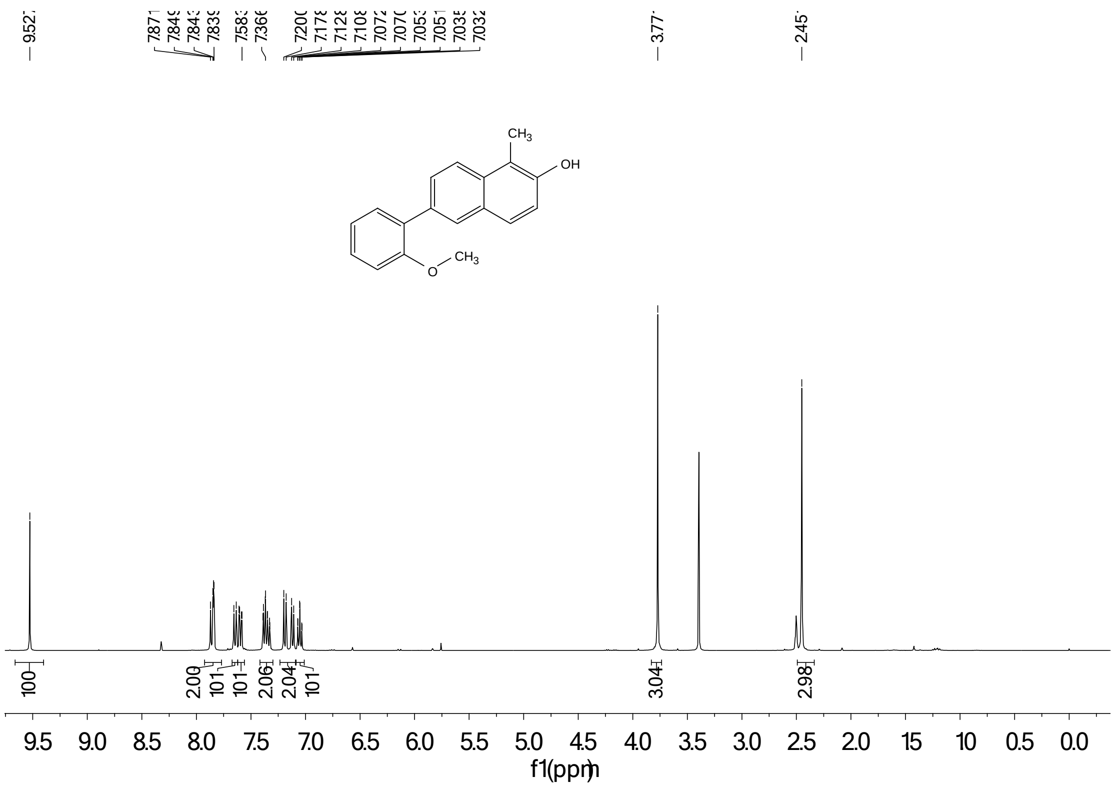

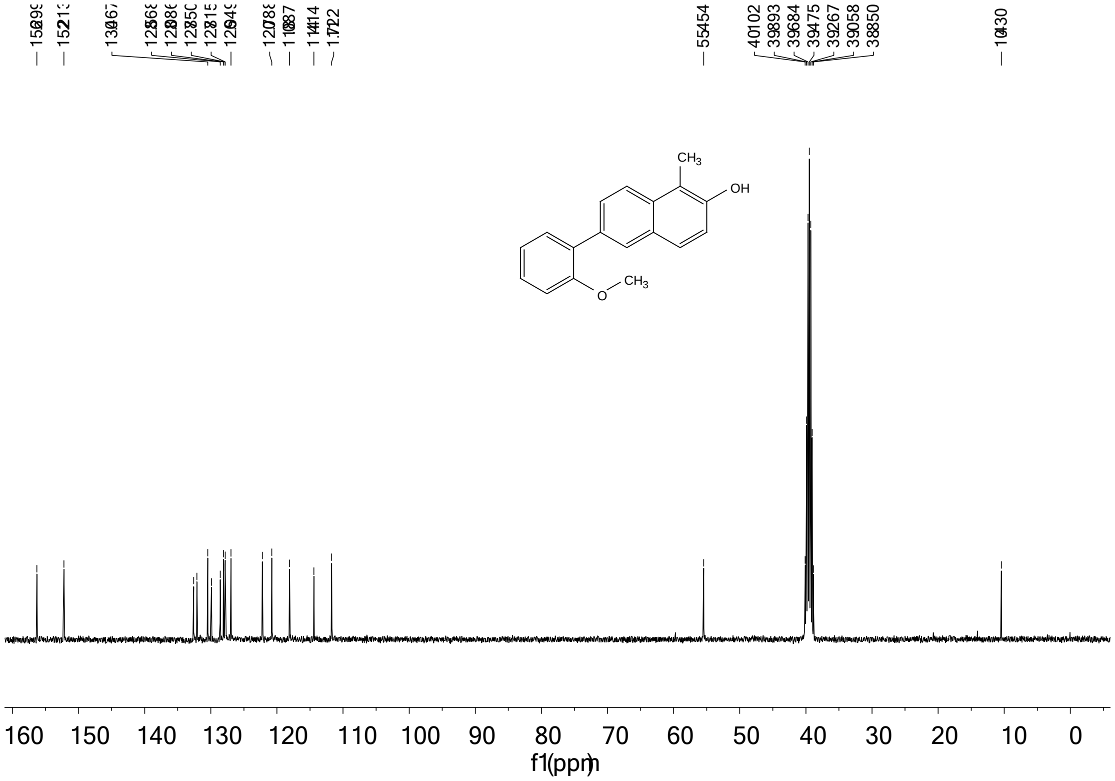

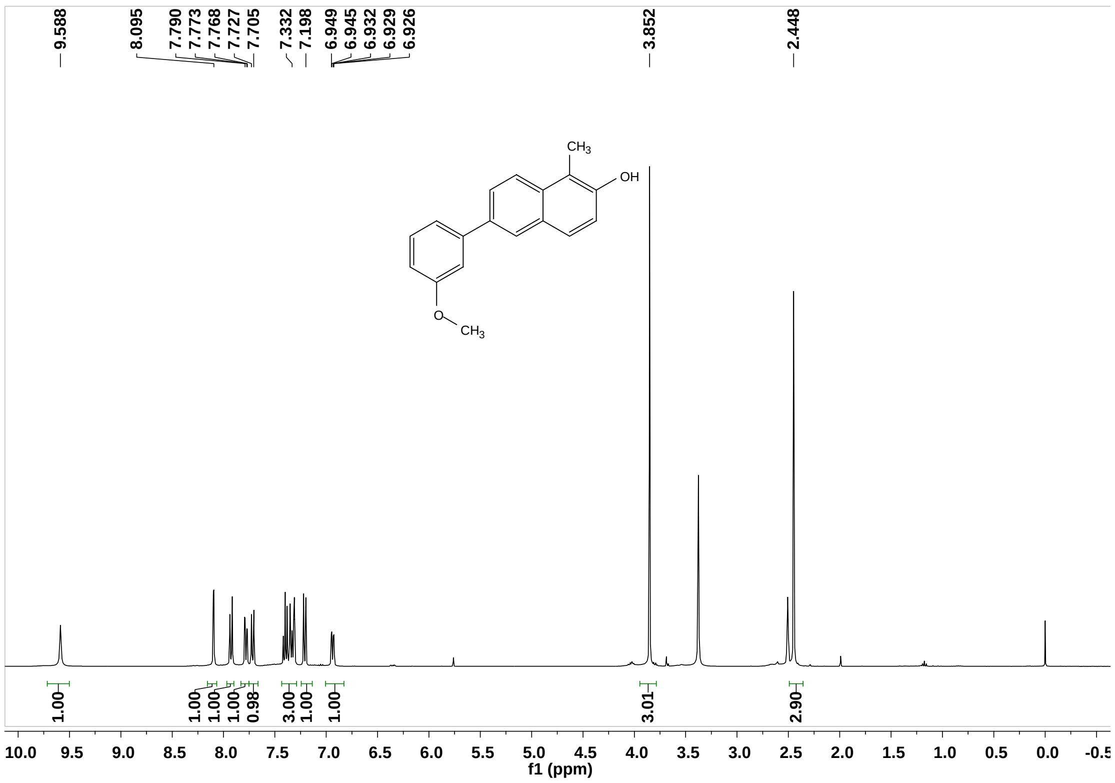

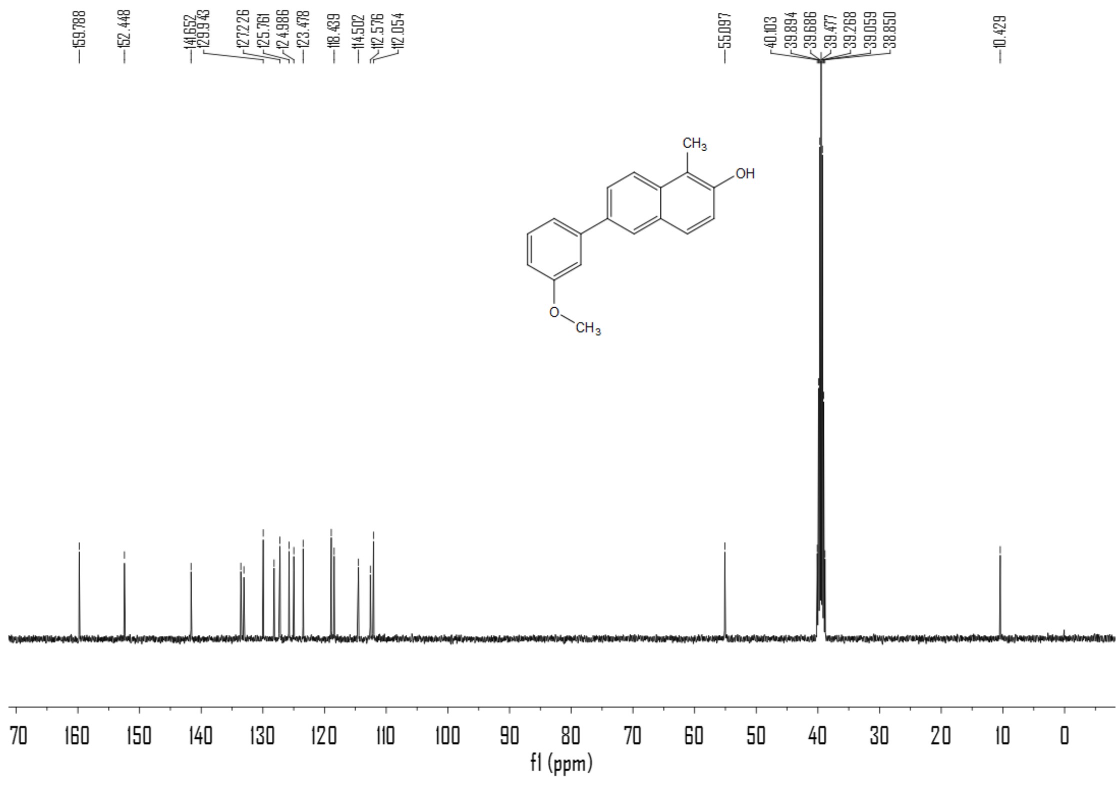

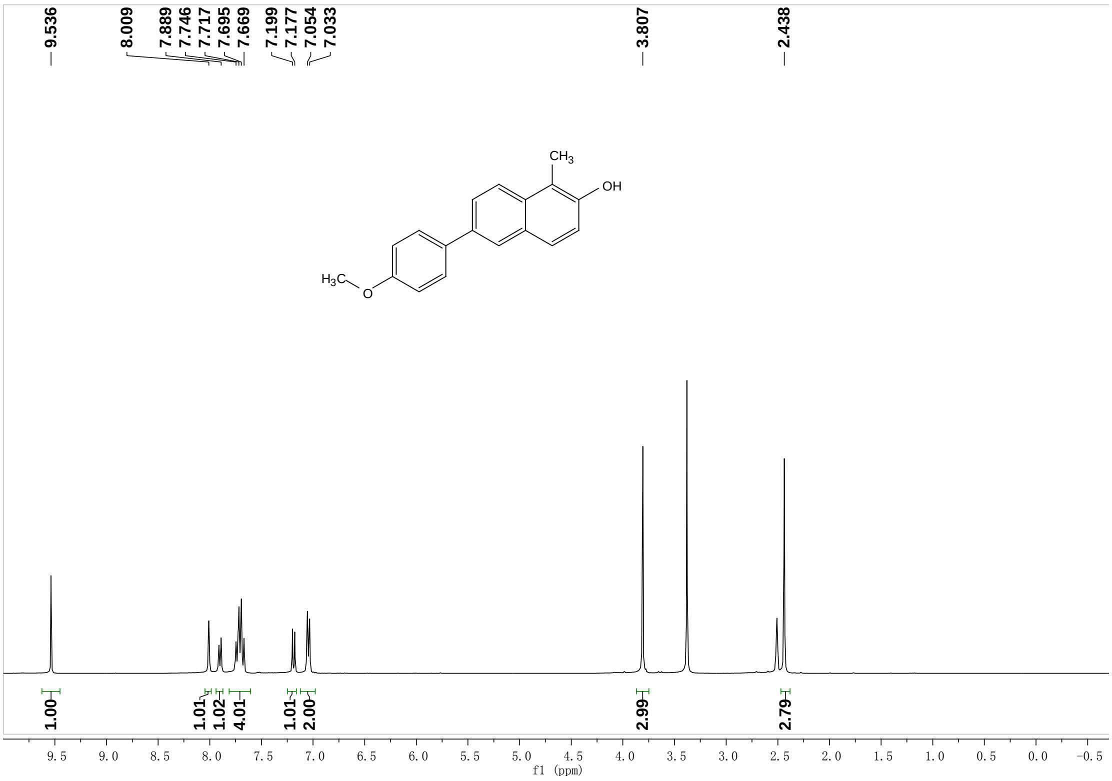
9.536
8.009
7.889
7.746
7.717
7.695
7.669
7.199
7.177
7.054
7.033
3.807
2.438
CH3
OH
H3C
O
1.00
1.01
1.02
4.01
1.01
2.00
2.99
2.79
f1 (ppm)

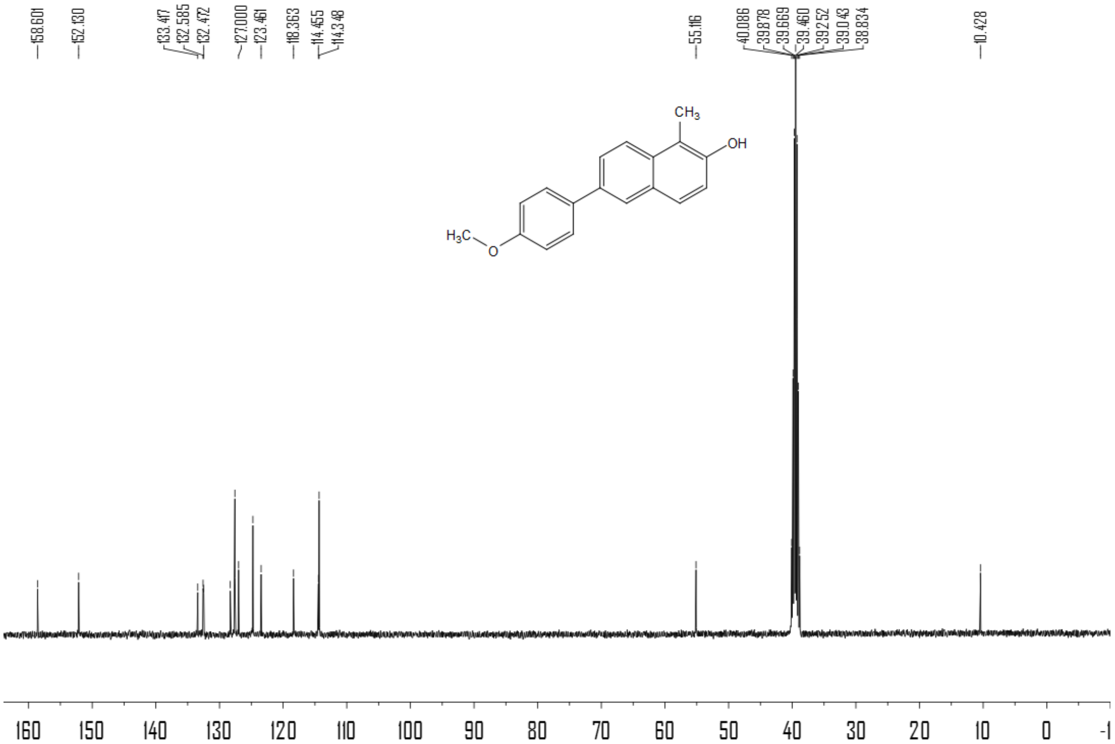
158.601
152.130
133.417
132.585
132.472
127.000
123.461
118.363
114.455
114.348
55.116
40.086
39.878
39.669
39.460
39.252
39.043
38.834
10.428
CH3
OH
H3C
O
f1 (ppm)

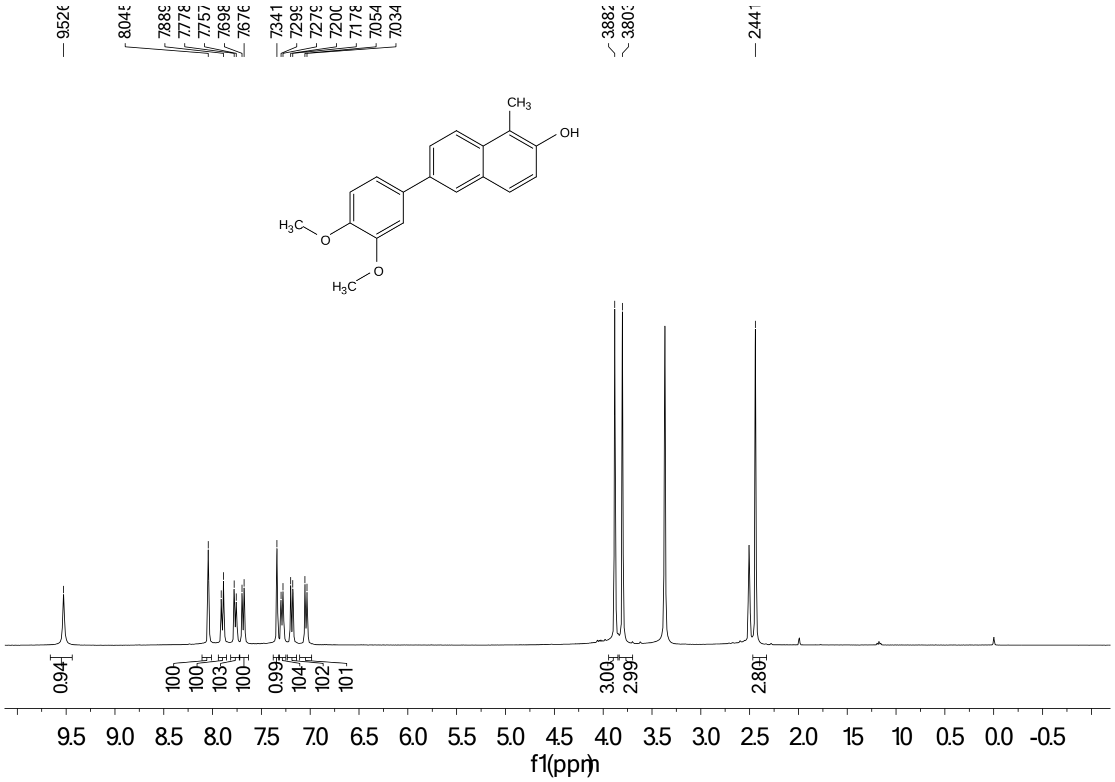
CH3
OH
H3C
O
f1 (ppm)

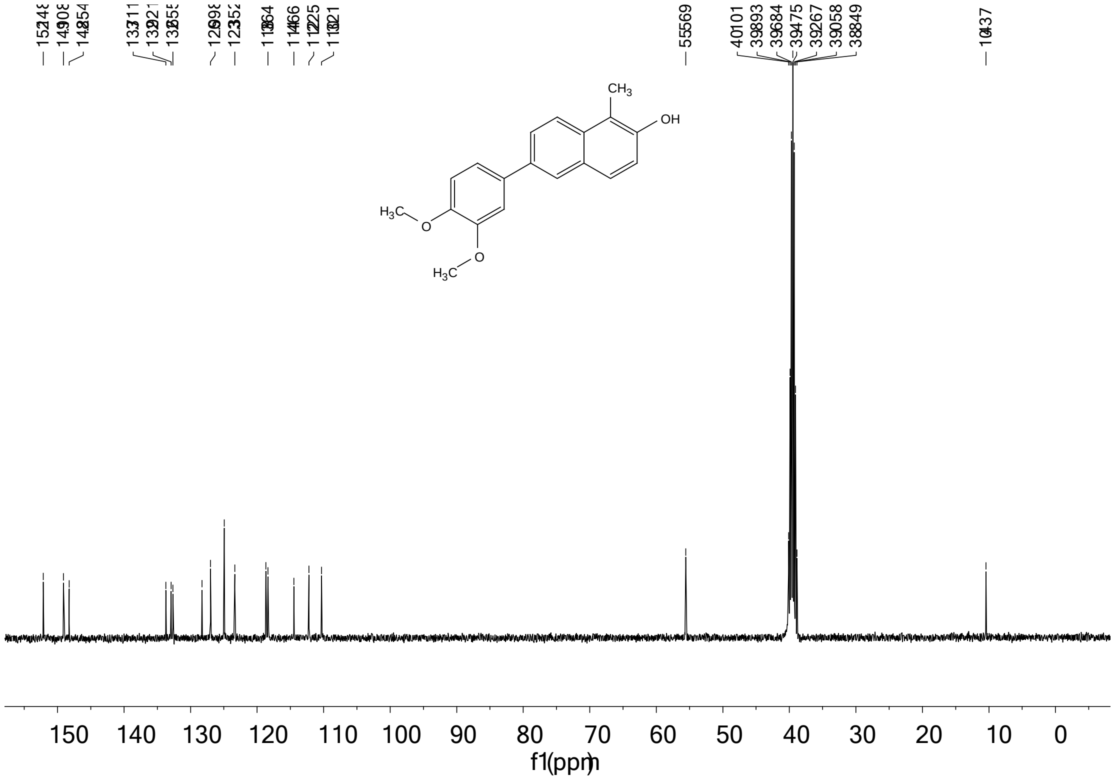
CH3
OH
H3C
O
f1 (ppm)

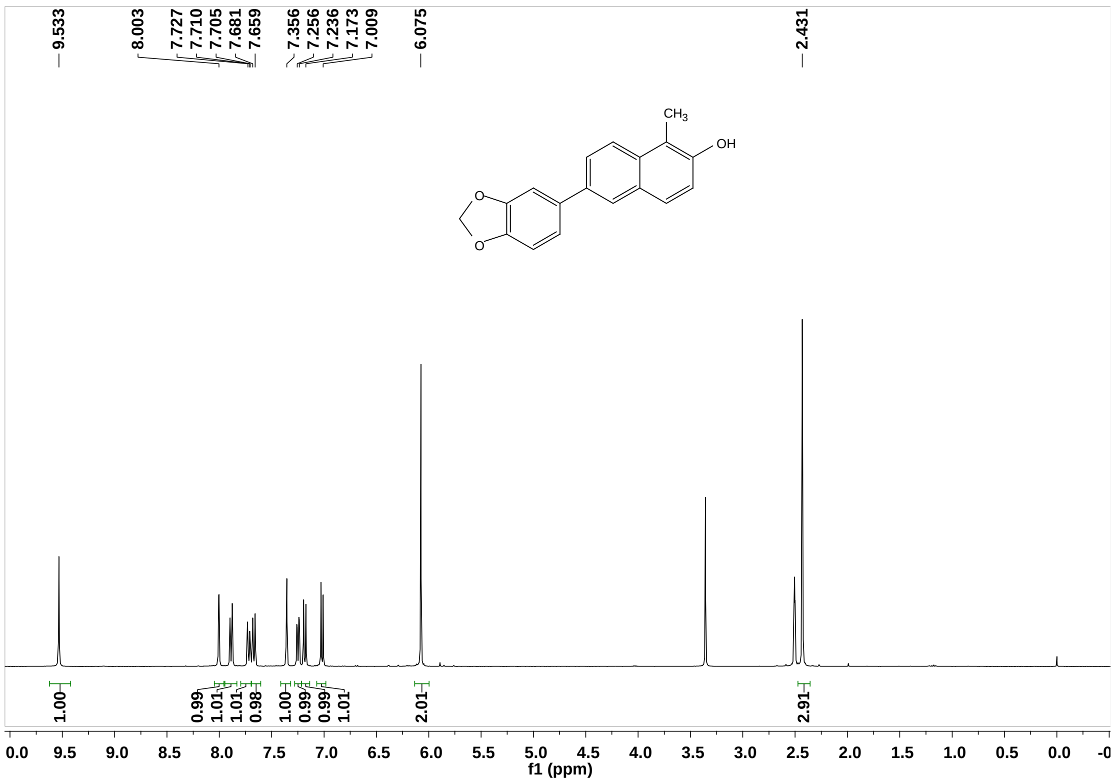

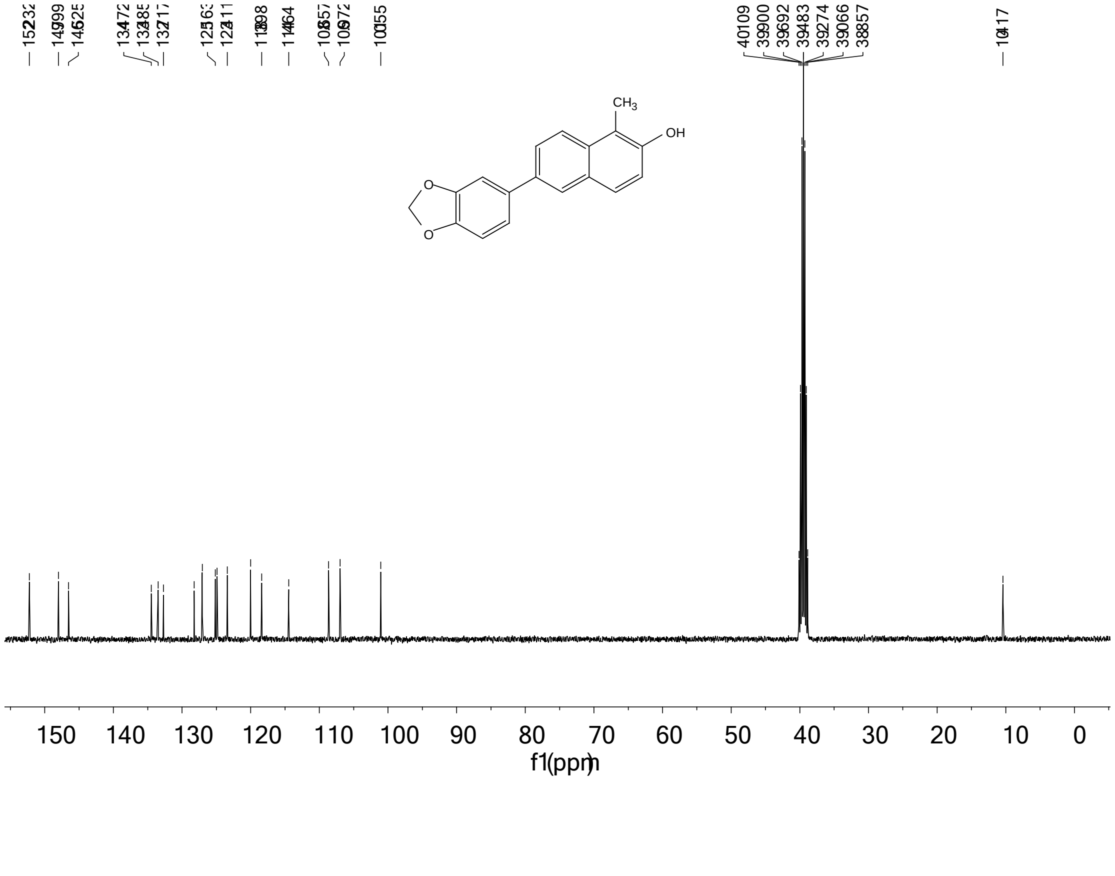

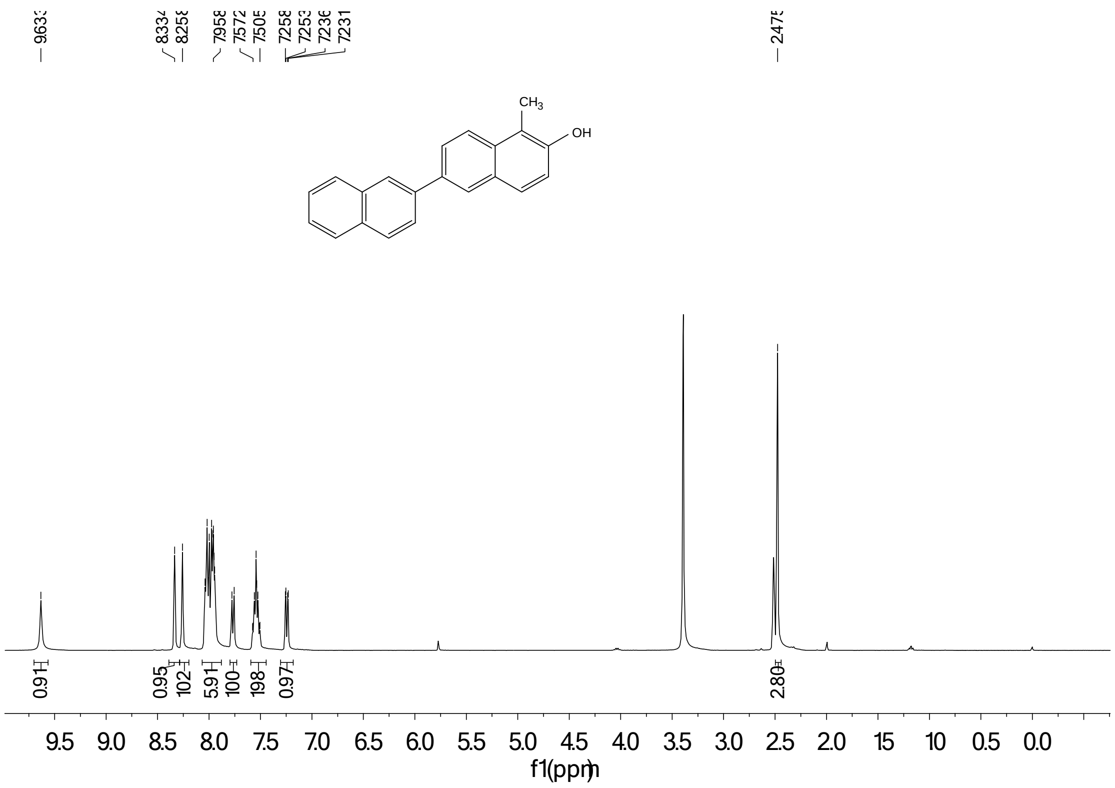

CH3
OH
f1 (ppm)

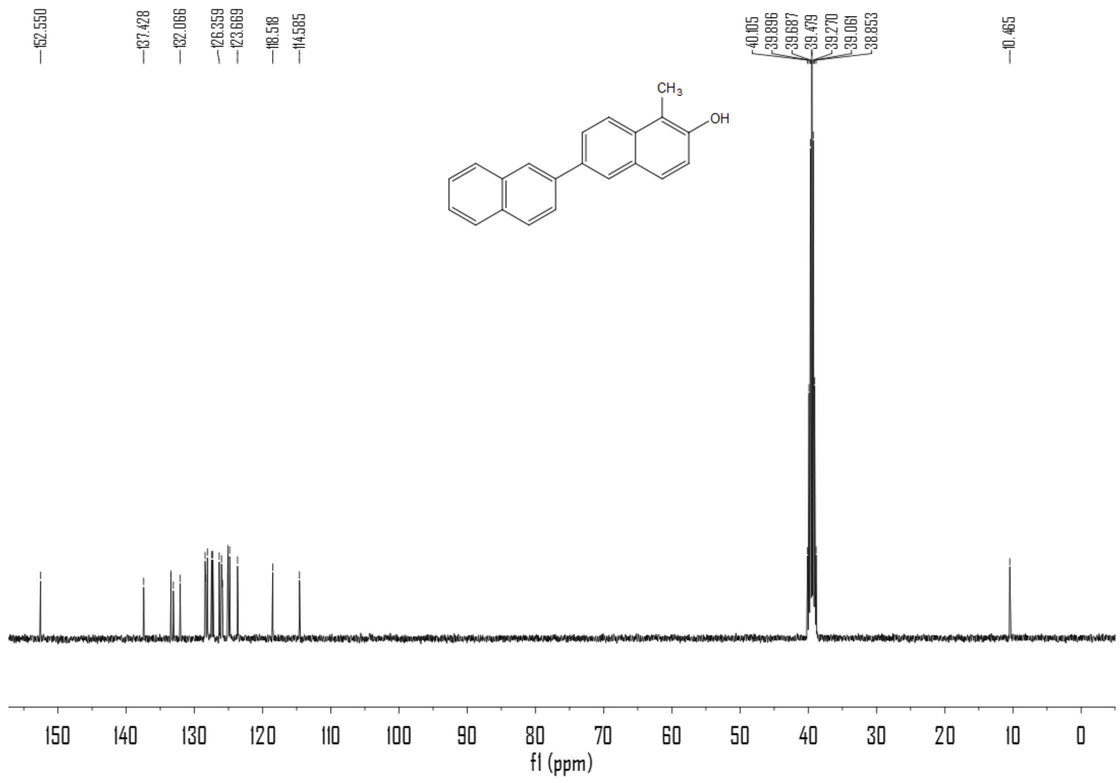

CH3
OH
f1 (ppm)

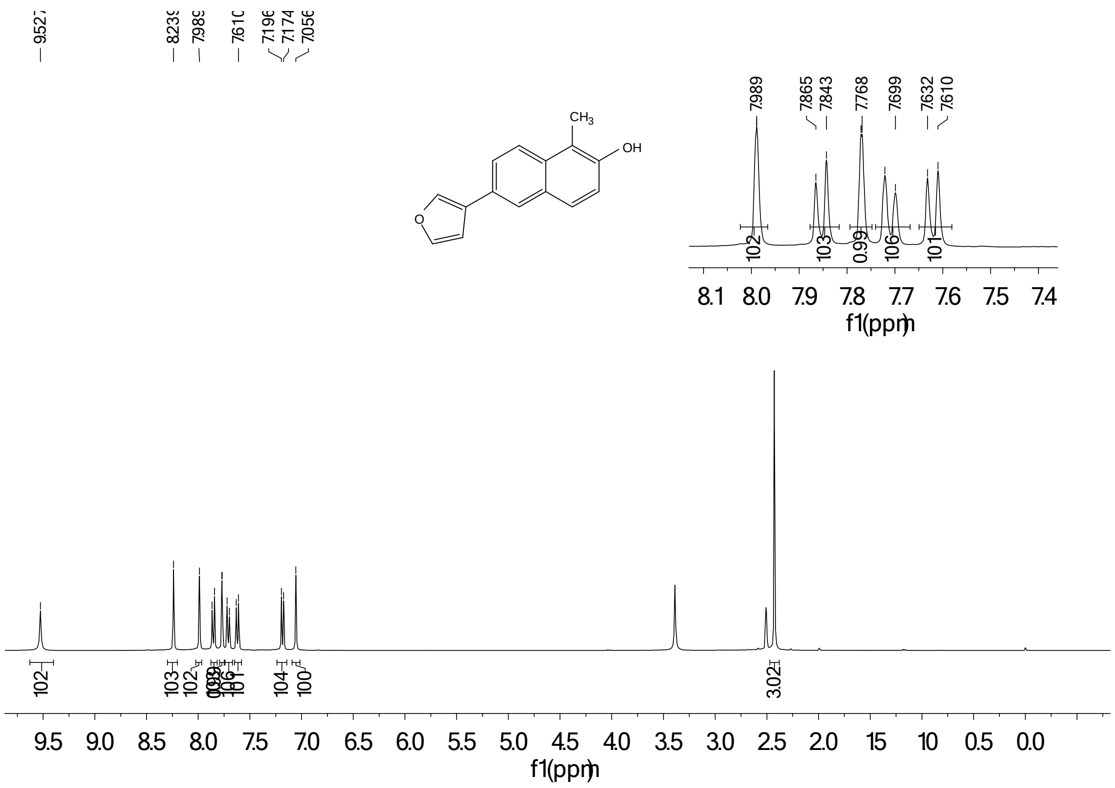
9.527
8.239
7.989
7.610
7.196
7.174
7.056
CH3
OH
O
7.989
7.865
7.843
7.768
7.699
7.632
7.610
1.02
1.03
0.99
1.06
1.01
8.1 8.0 7.9 7.8 7.7 7.6 7.5 7.4
f1 (ppm)
1.02
1.03
1.02
1.04
1.00
3.02
9.5 9.0 8.5 8.0 7.5 7.0 6.5 6.0 5.5 5.0 4.5 4.0 3.5 3.0 2.5 2.0 1.5 1.0 0.5 0.0
f1 (ppm)

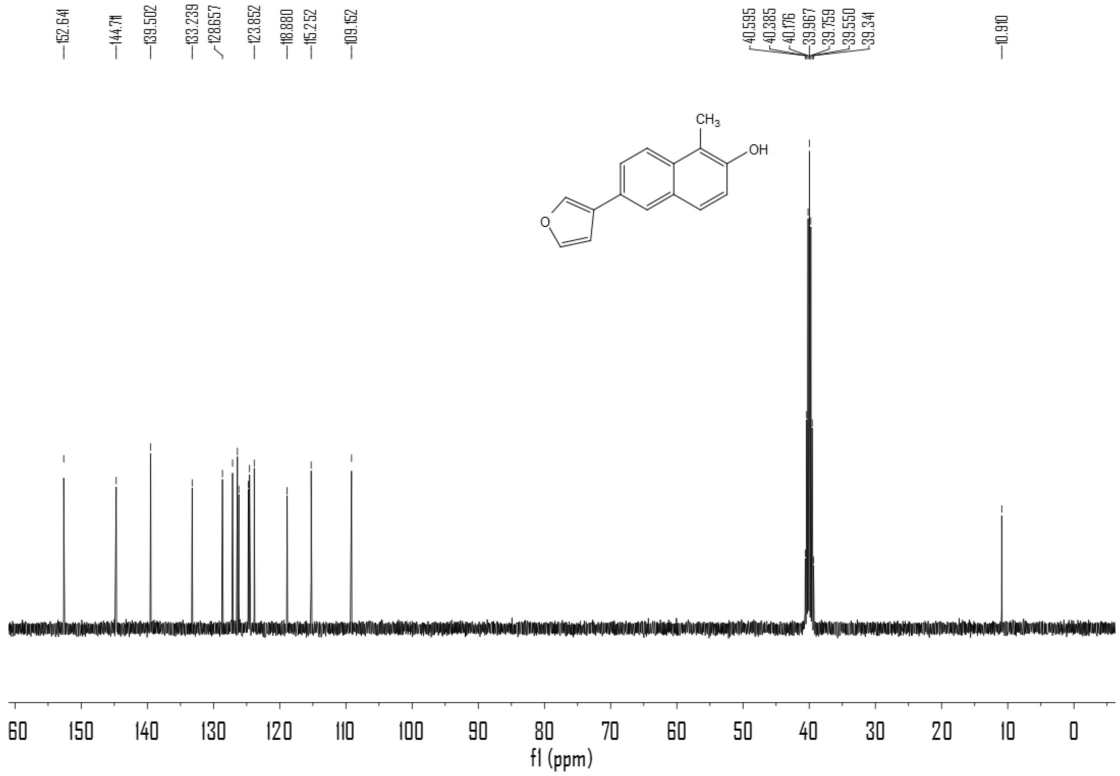
152.641
144.711
139.502
133.239
128.657
123.852
118.880
115.252
109.152
40.595
40.385
40.176
39.967
39.759
39.550
39.341
10.910
CH3
OH
O
150 140 130 120 110 100 90 80 70 60 50 40 30 20 10 0
f1 (ppm)

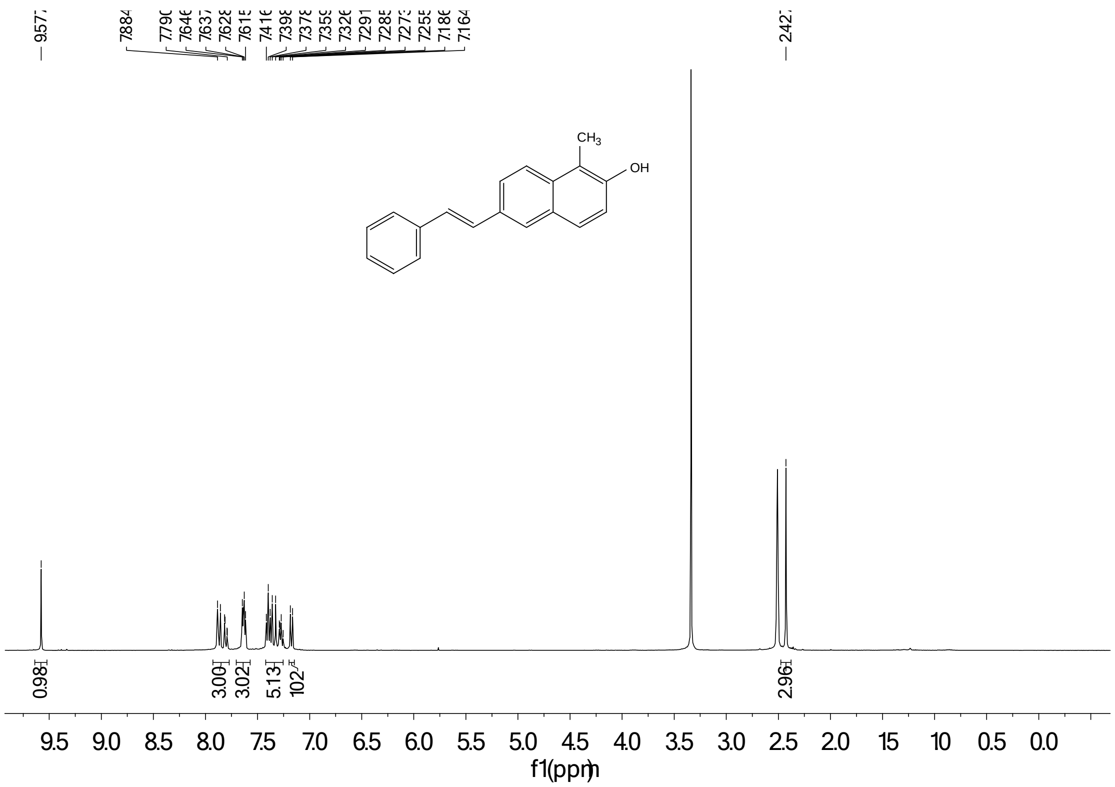

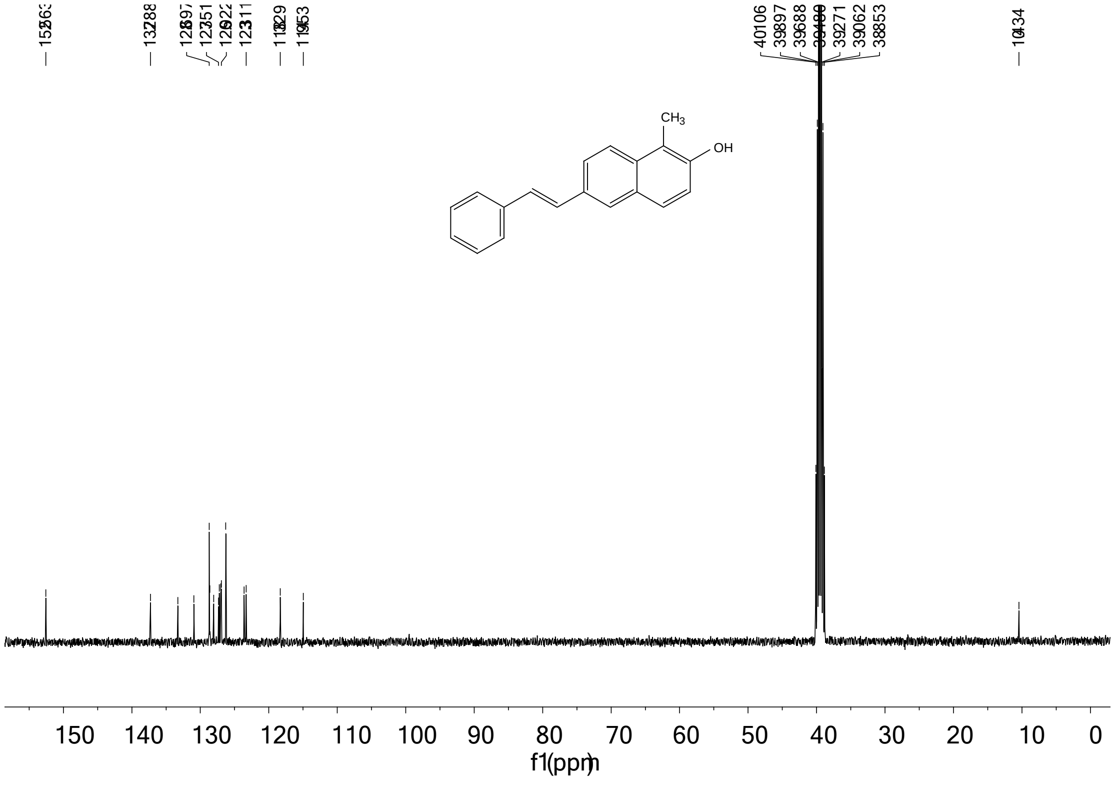

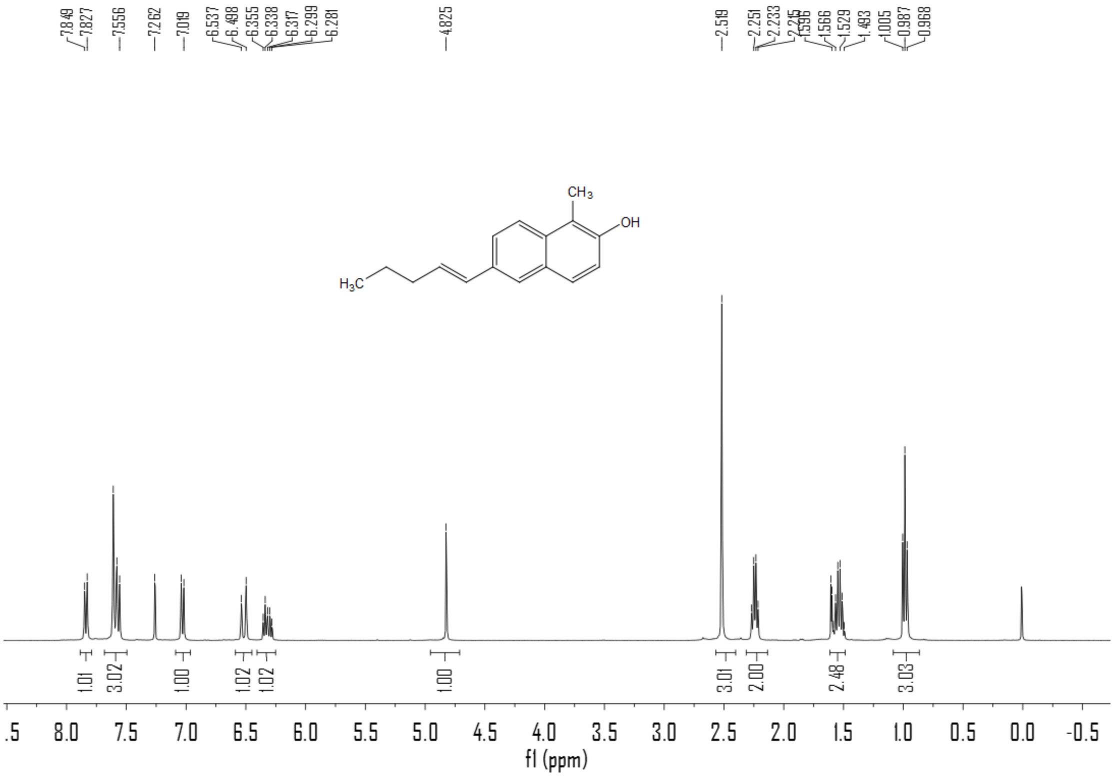

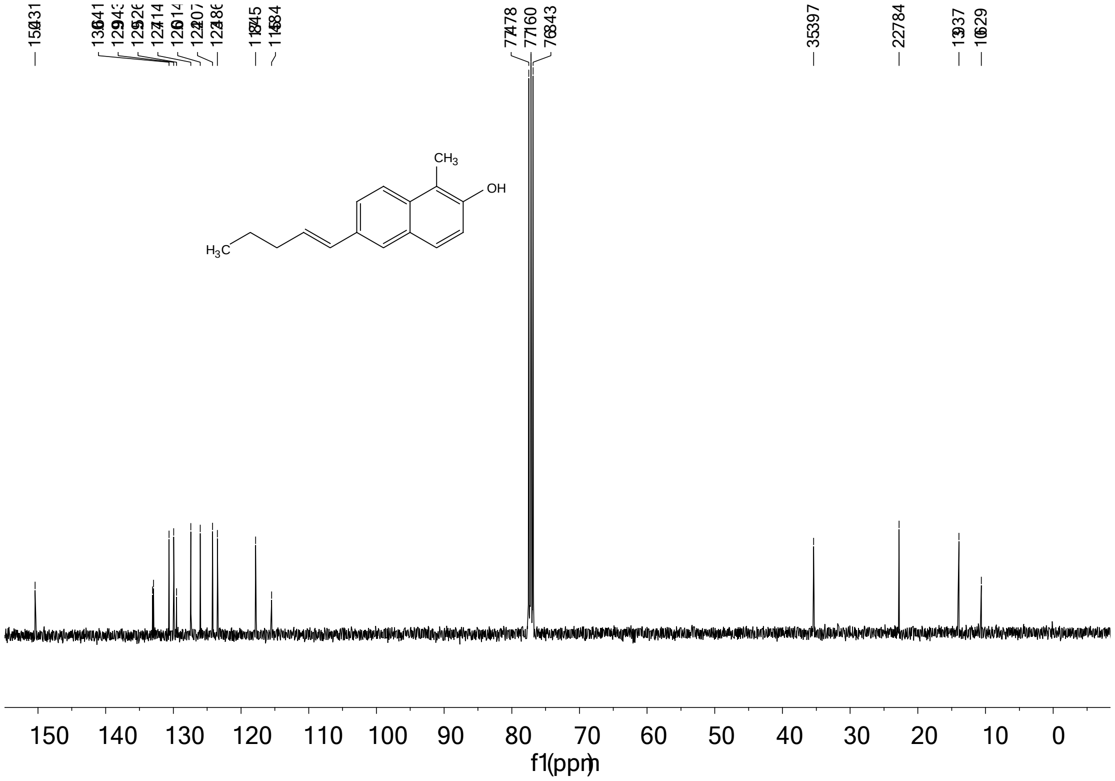

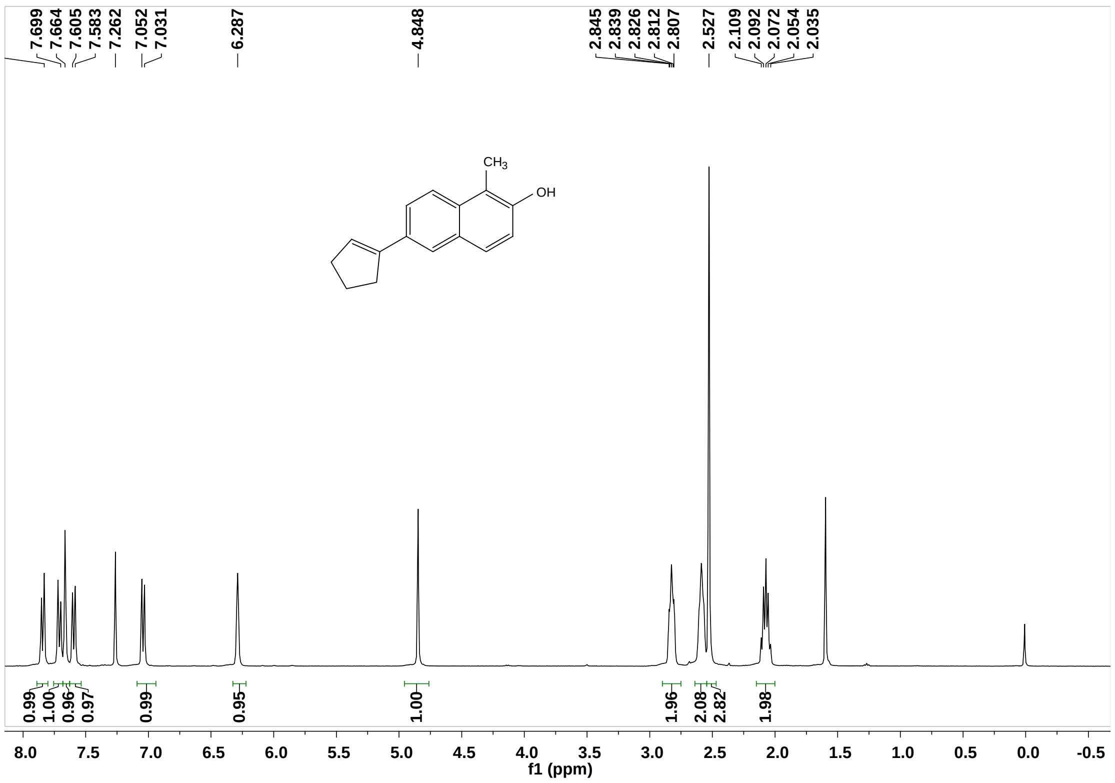

CH3
OH
f1 (ppm)

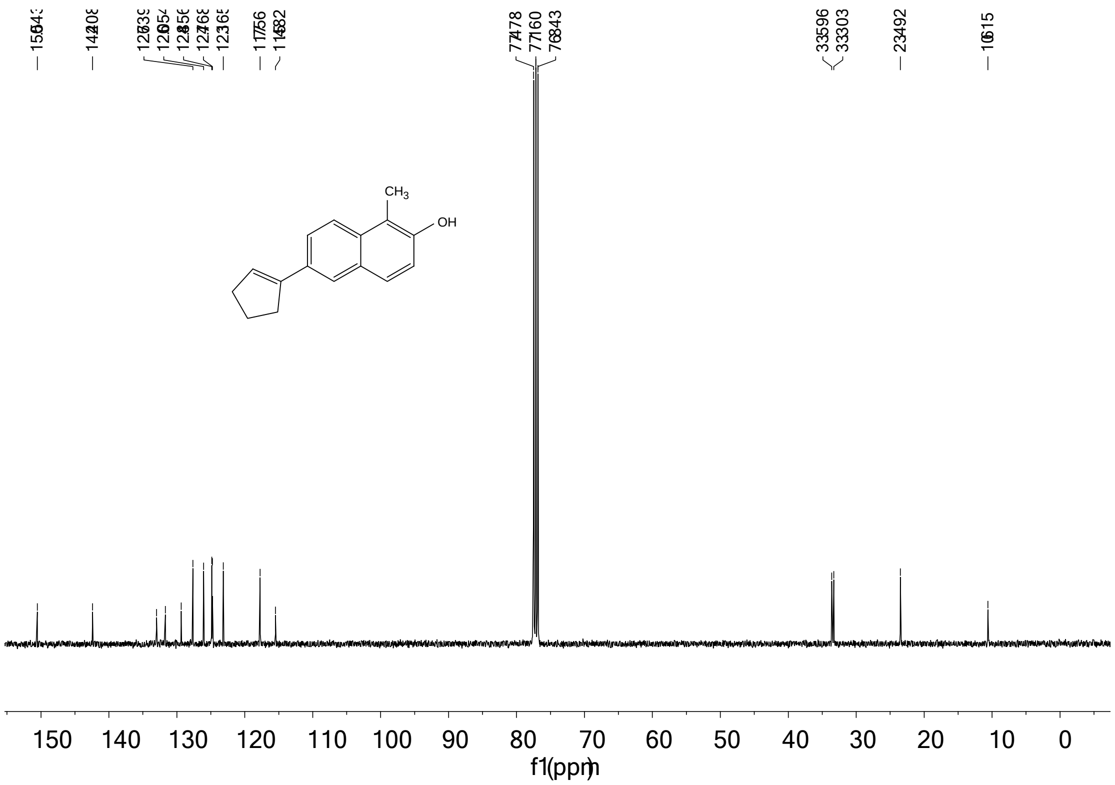

CH3
OH
f1 (ppm)

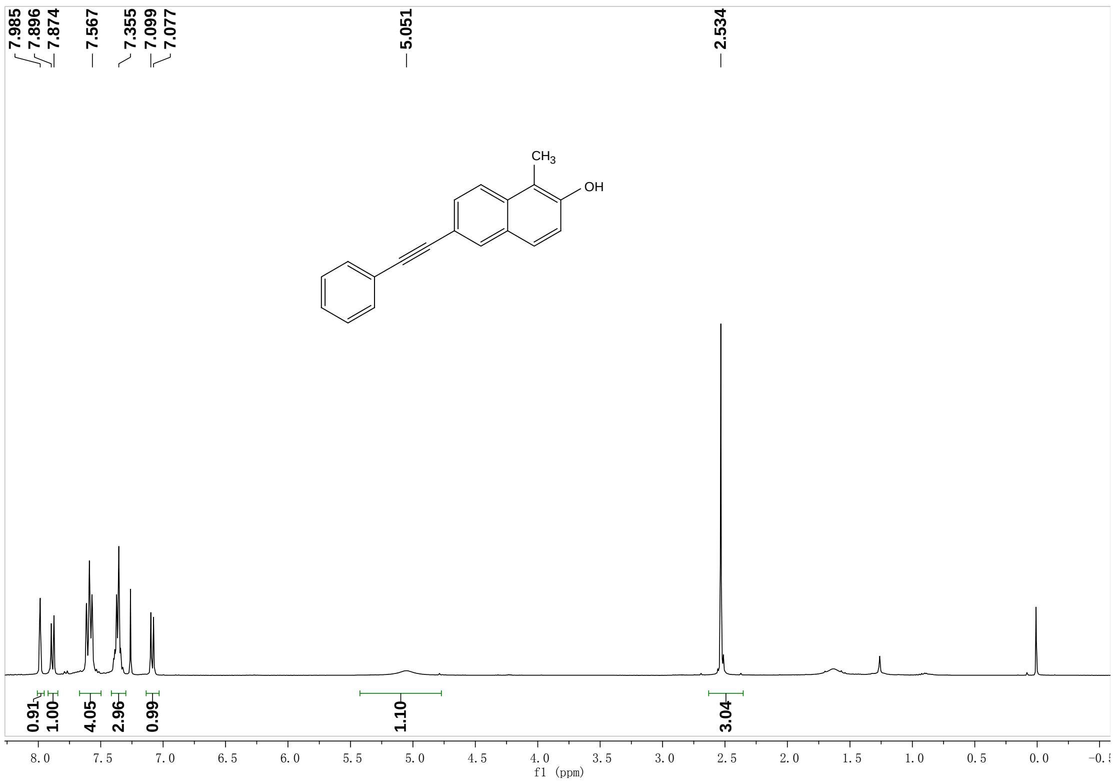
7.985
7.896
7.874
7.567
7.355
7.099
7.077
5.051
2.534
CH3
OH
0.91
1.00
4.05
2.96
0.99
1.10
3.04
f1 (ppm)

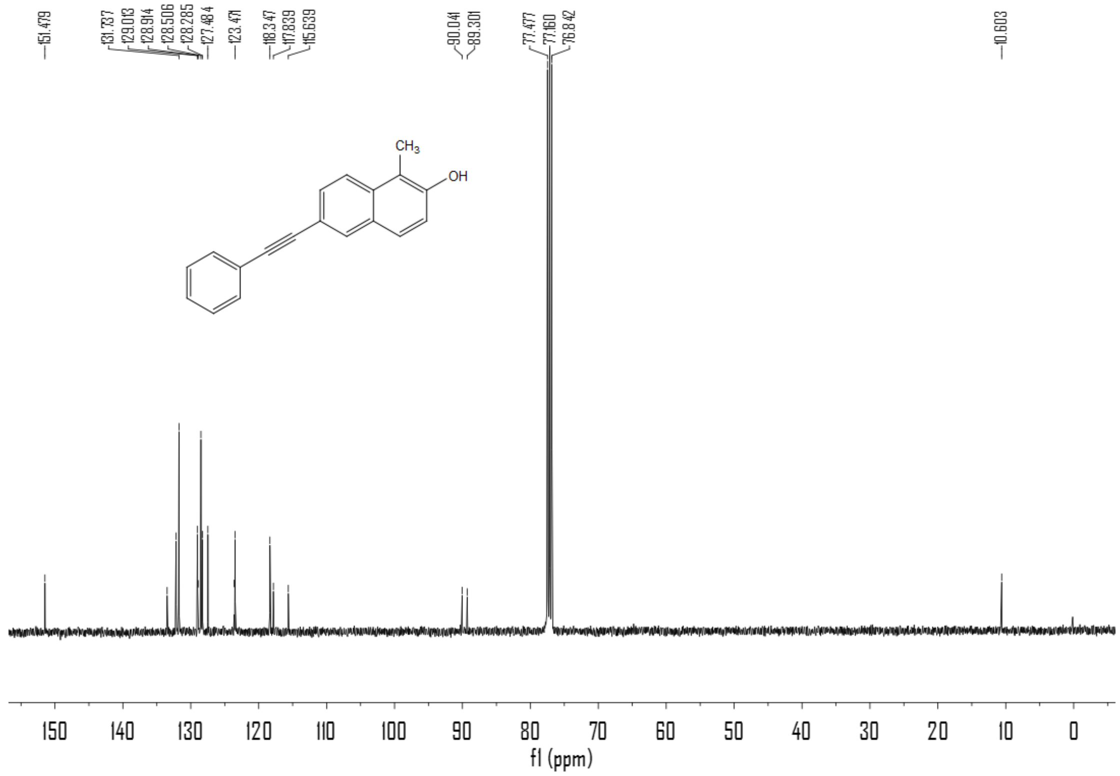
151.479
131.737
129.013
128.914
128.506
128.285
127.484
123.471
118.347
117.839
115.639
90.041
89.301
77.477
77.160
76.842
10.603
CH3
OH
f1 (ppm)

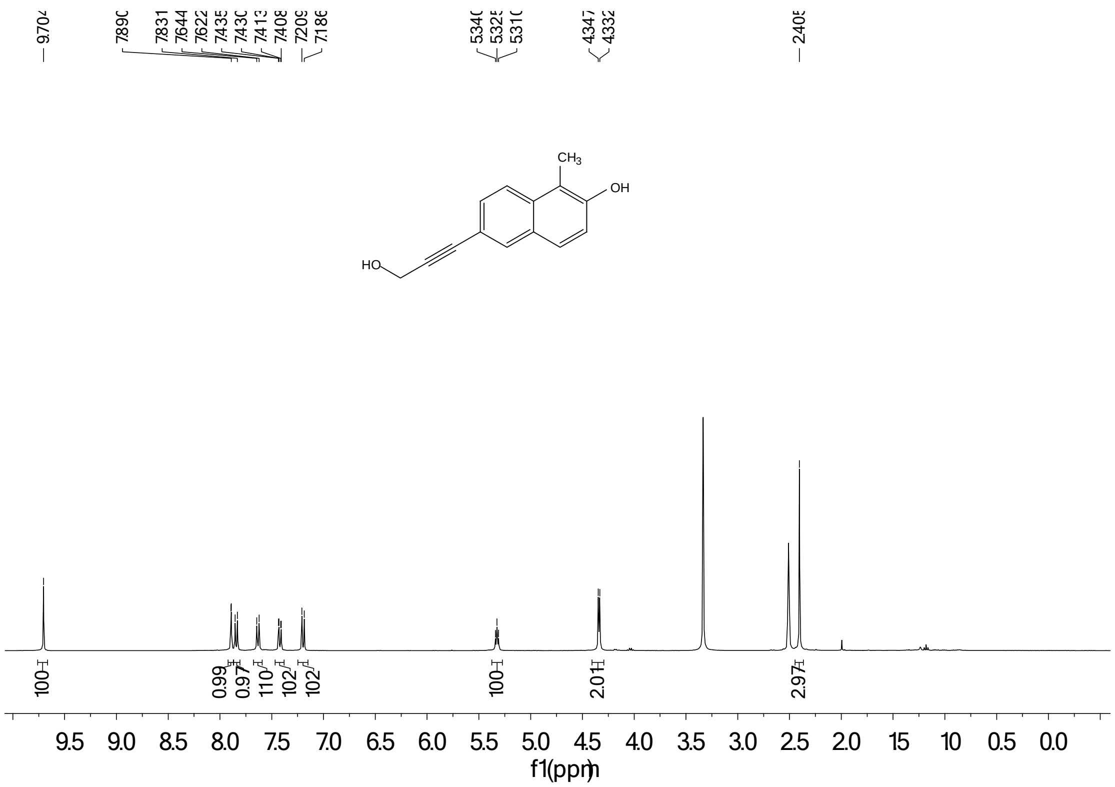

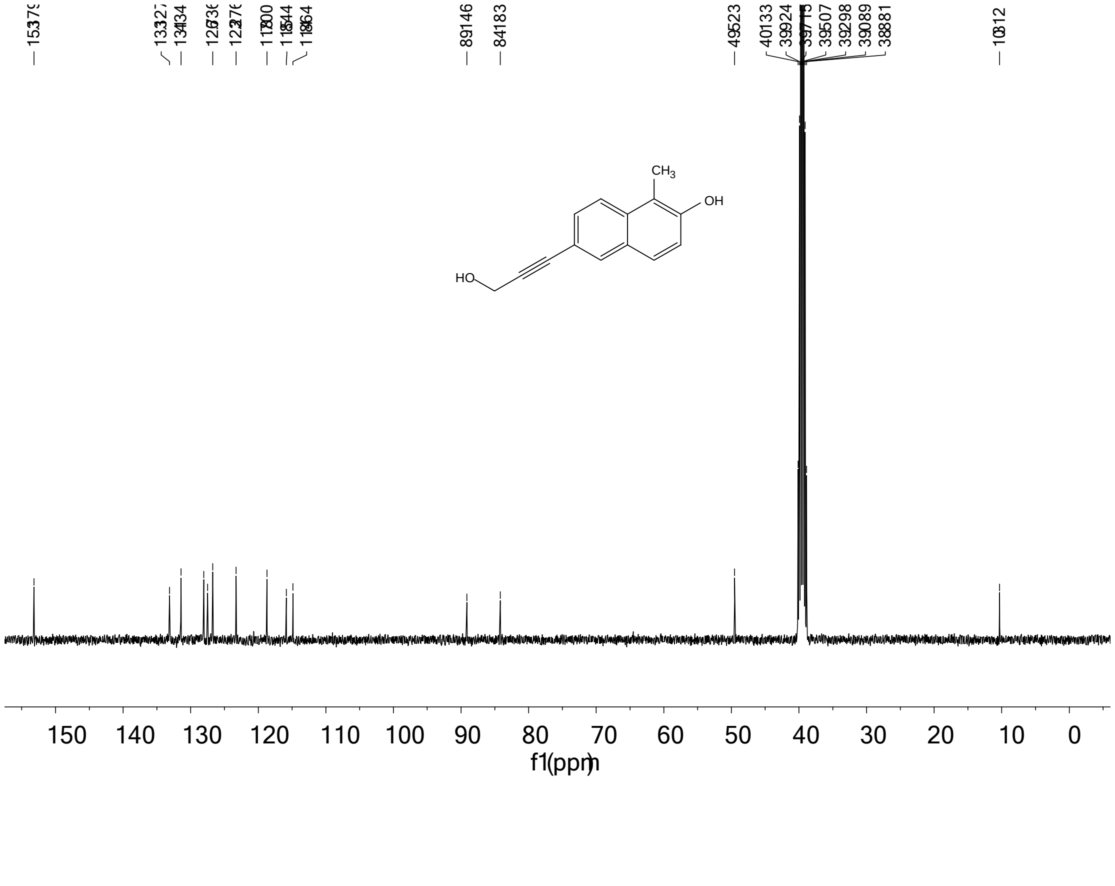

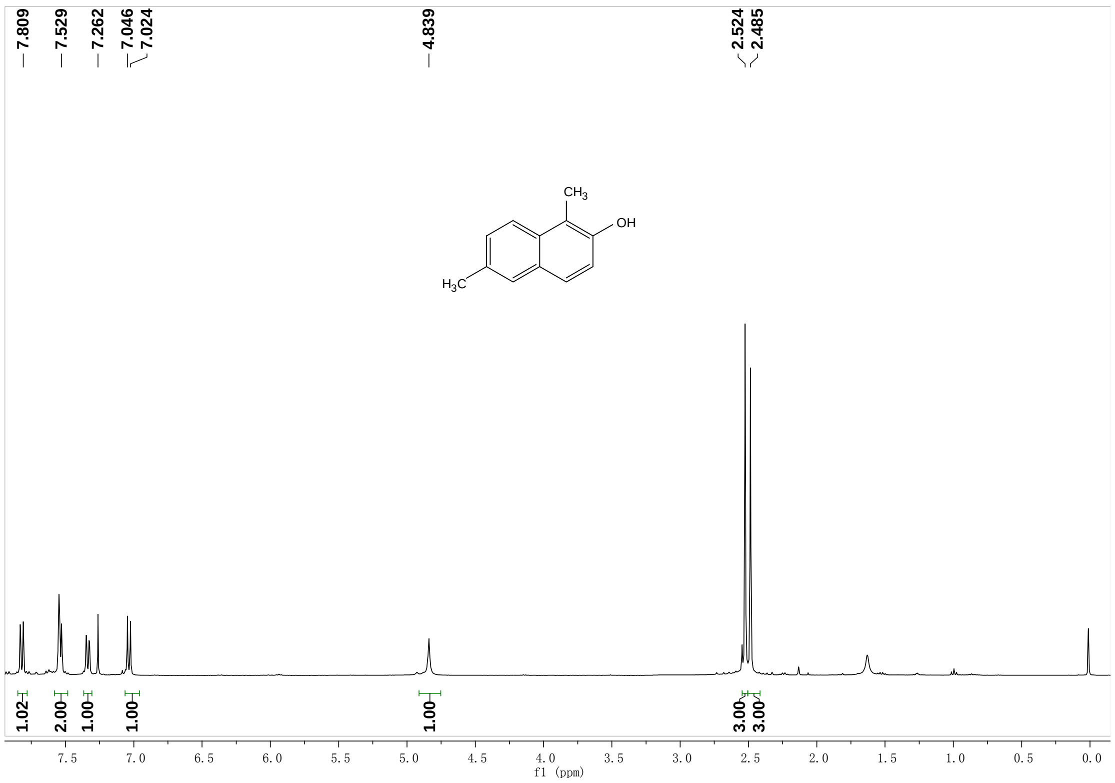

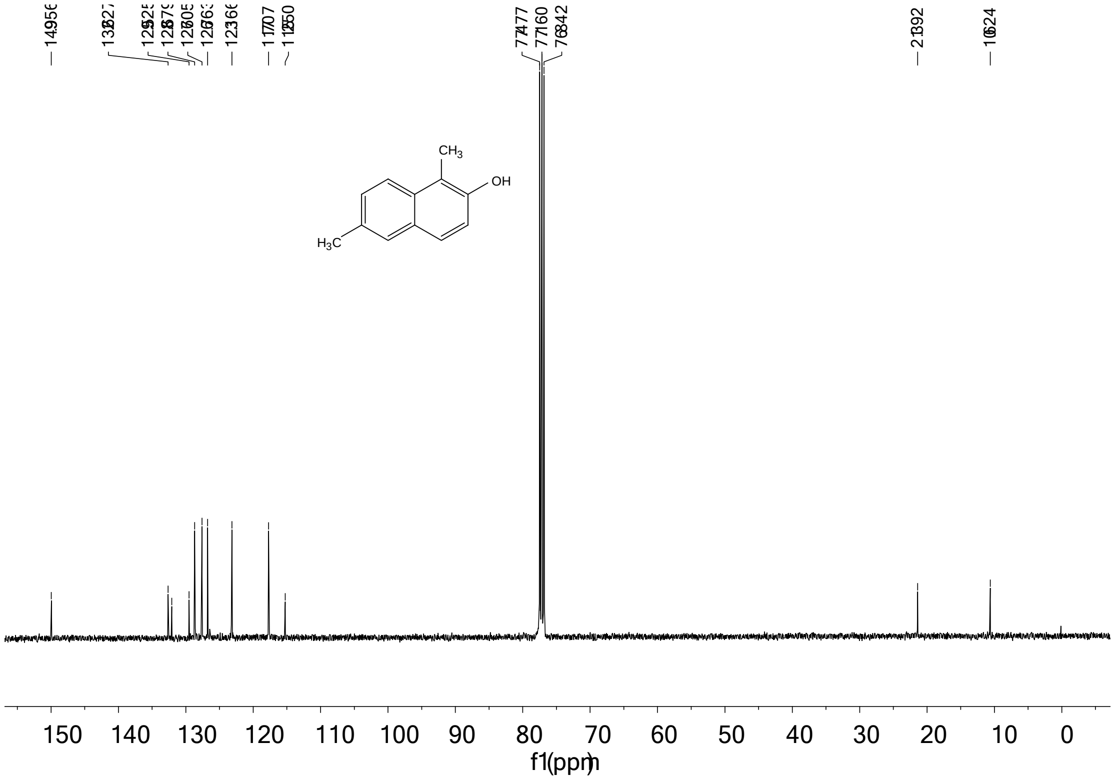

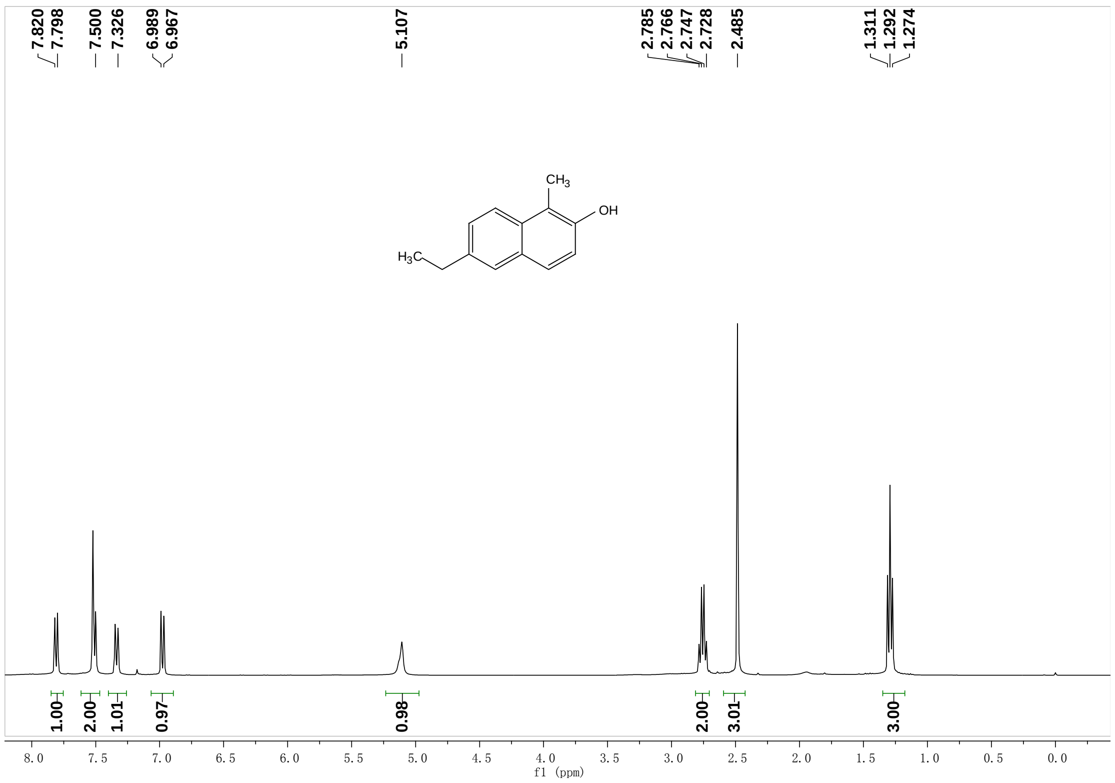

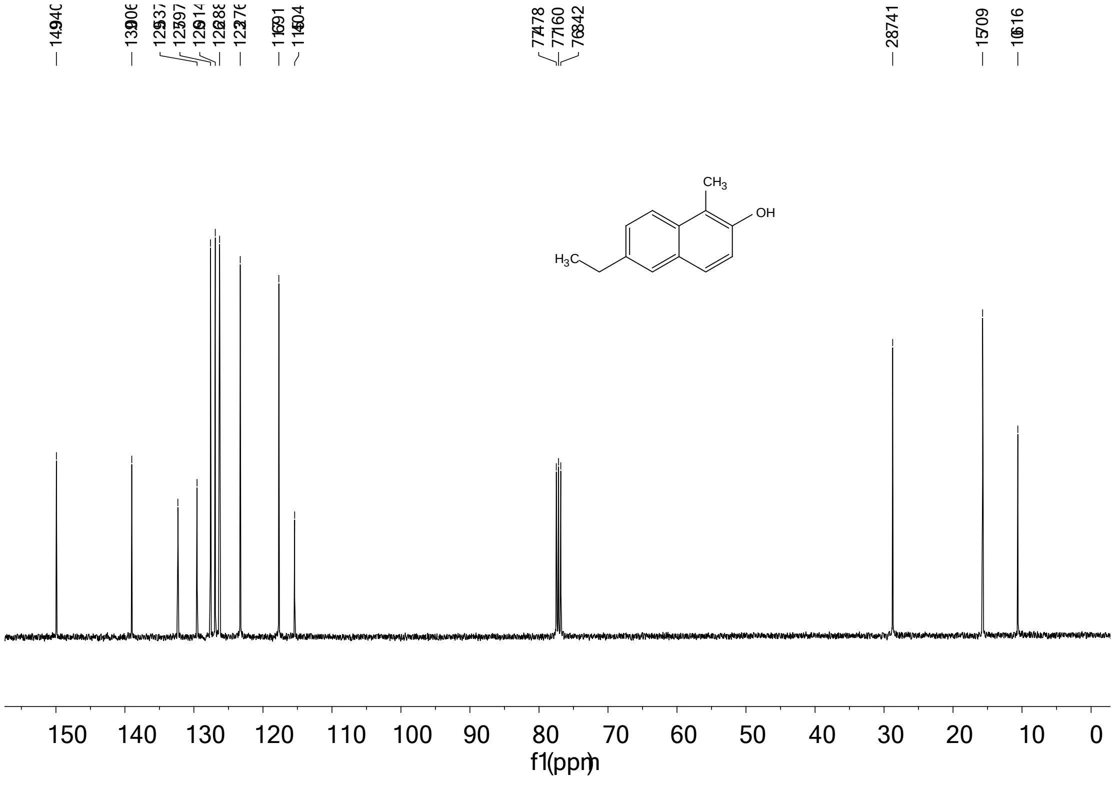

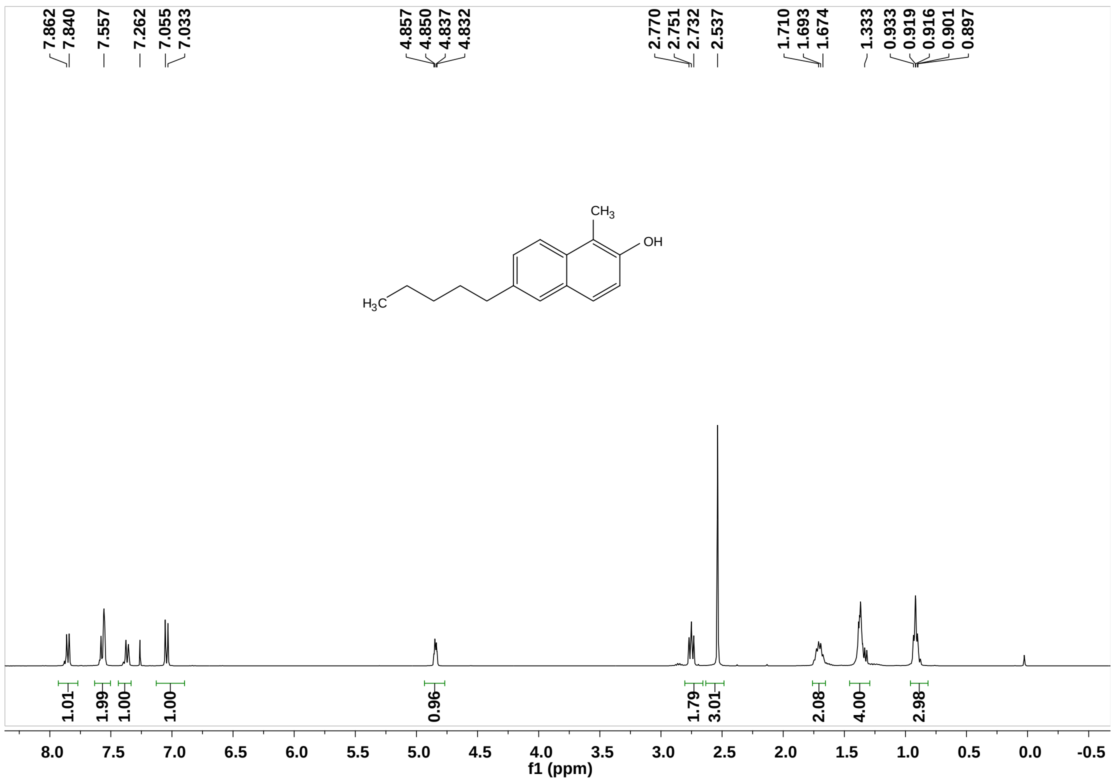

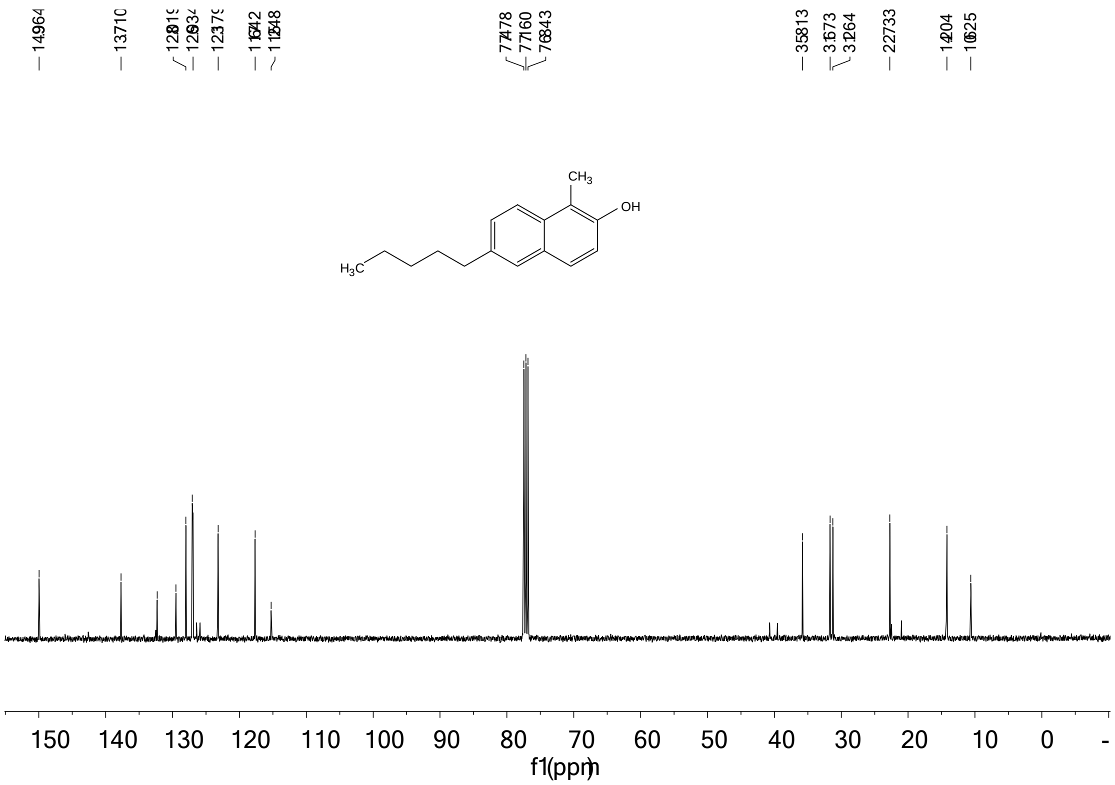

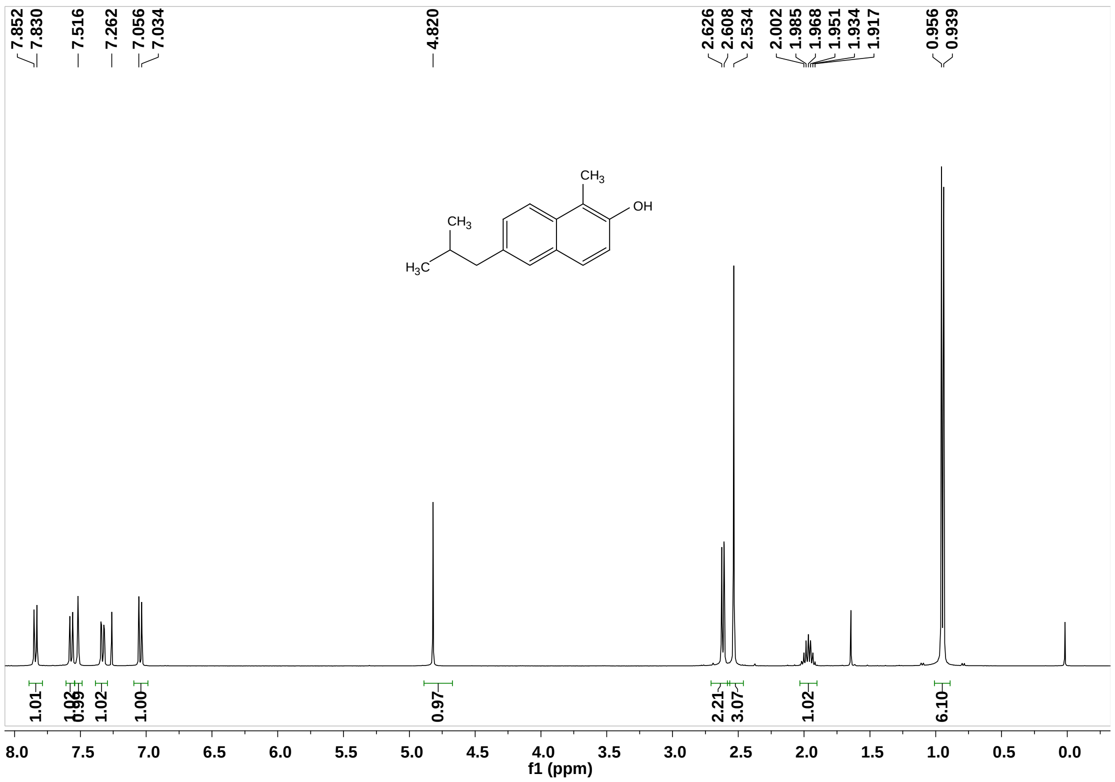

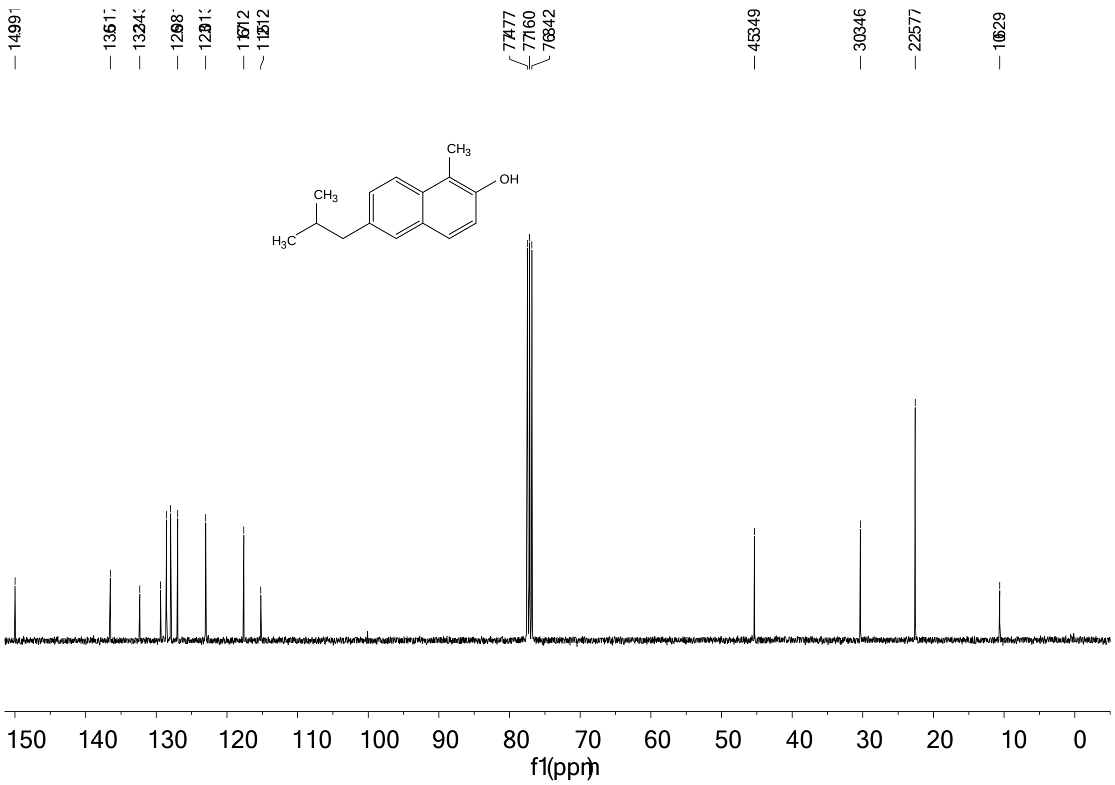

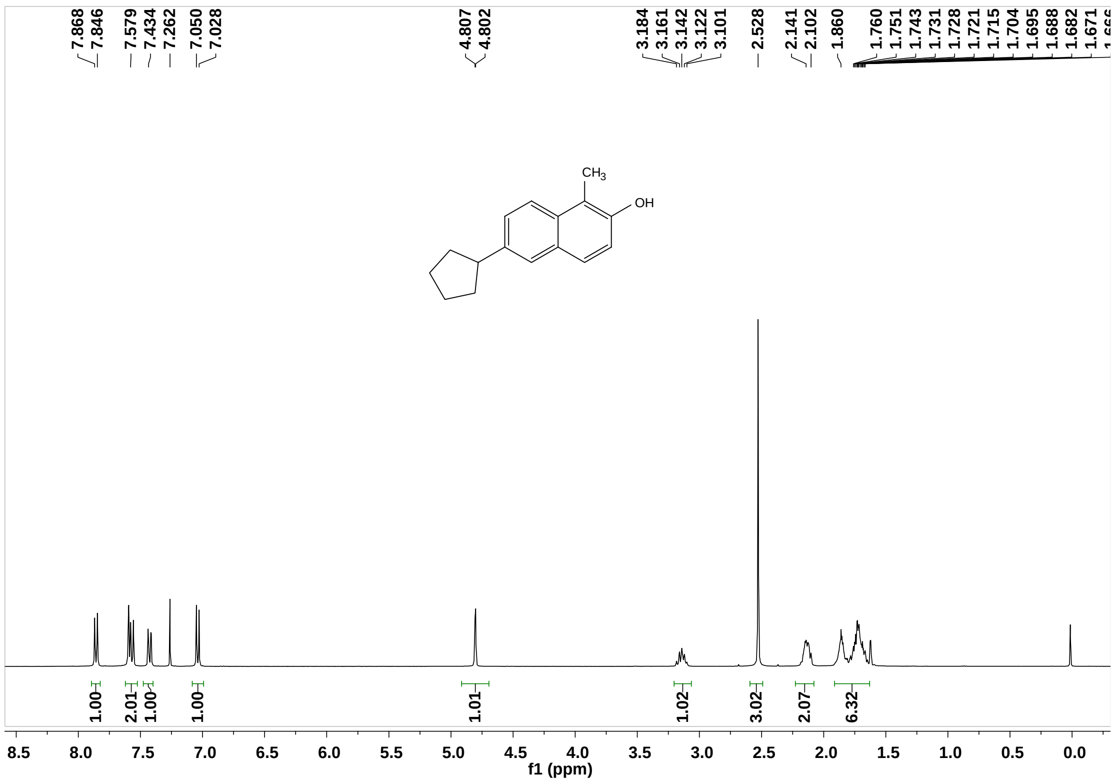
CH3
OH
f1 (ppm)

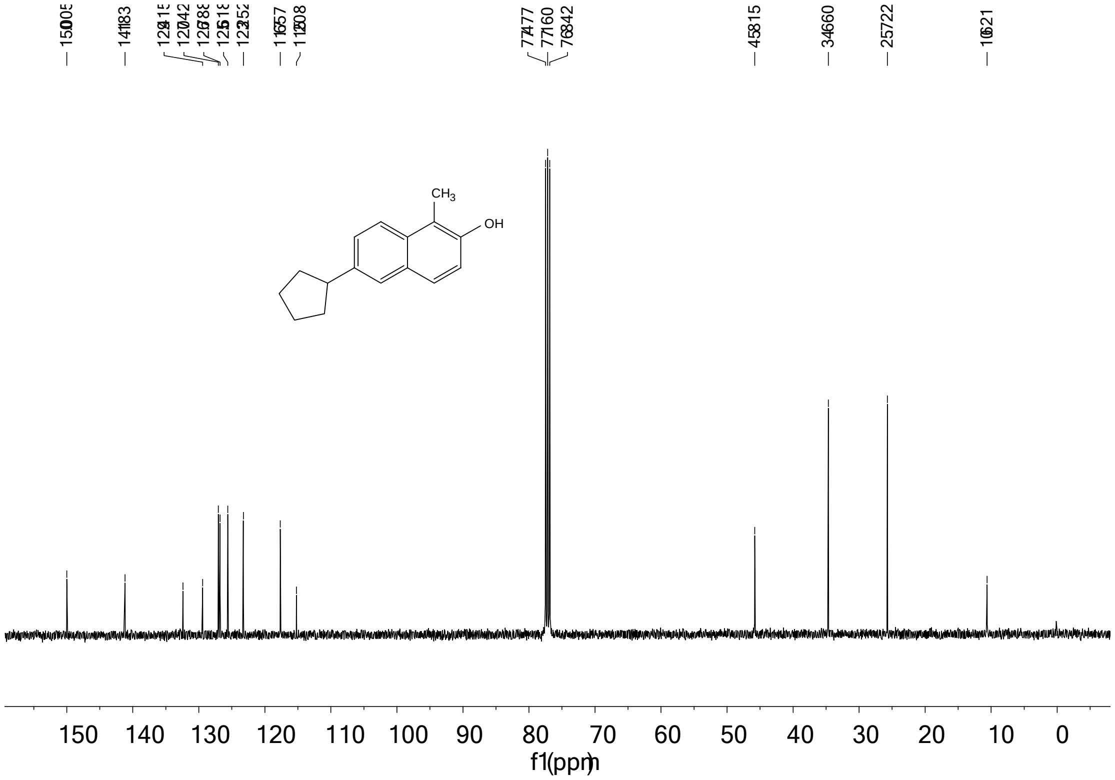
CH3
OH
f1 (ppm)

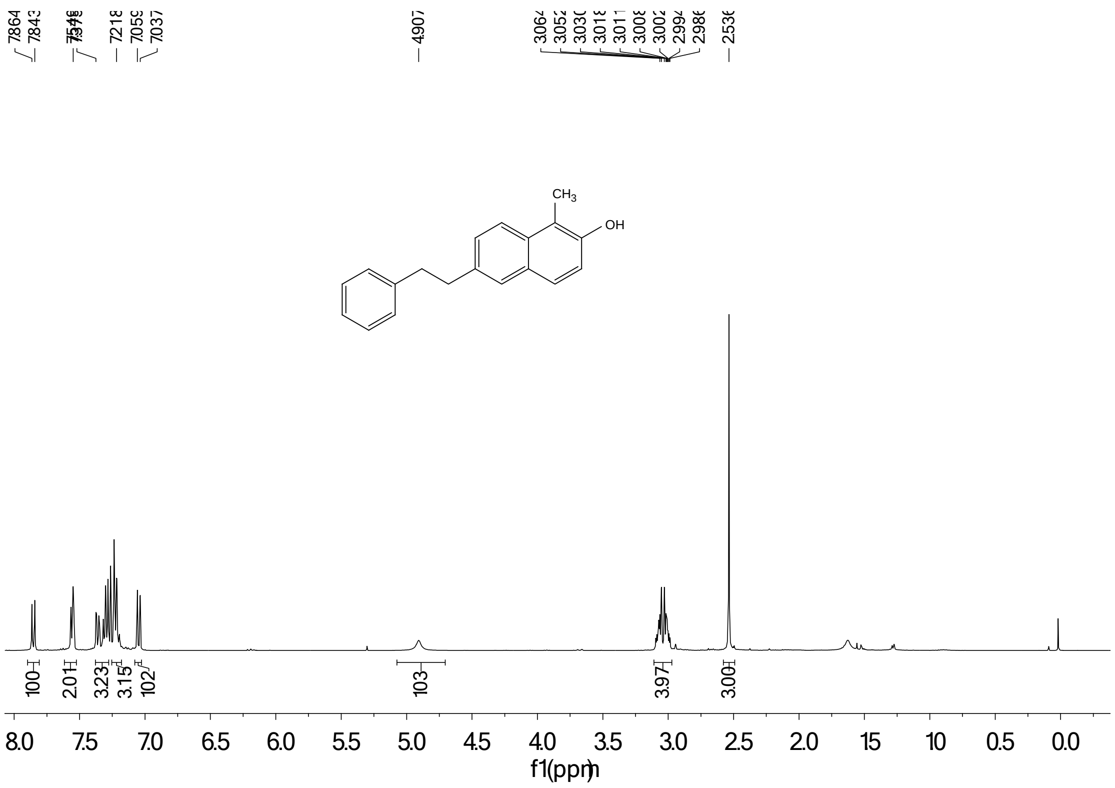
CH3
OH
f1 (ppm)

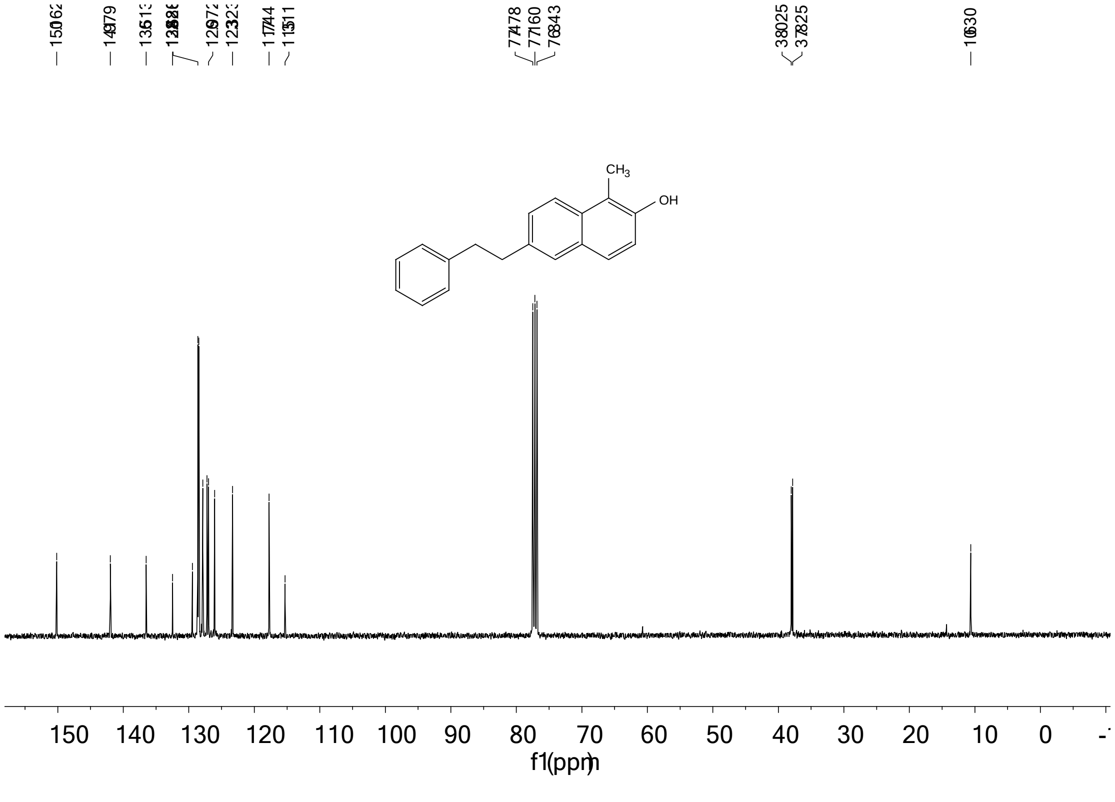
CH3
OH
f1 (ppm)

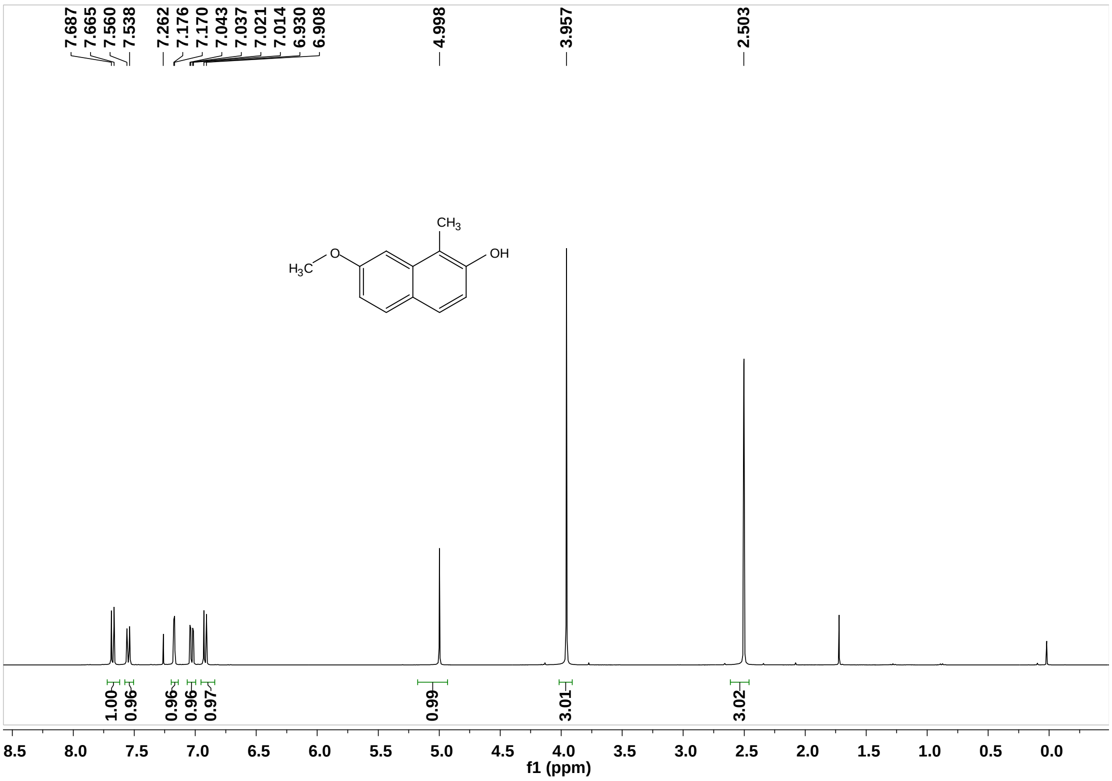
7.687
7.665
7.560
7.538
7.262
7.176
7.170
7.043
7.037
7.021
7.014
6.930
6.908
4.998
3.957
2.503
CH3
OH
H3C
O
1.00
0.96
0.96
0.96
0.97
0.99
3.01
3.02
f1 (ppm)

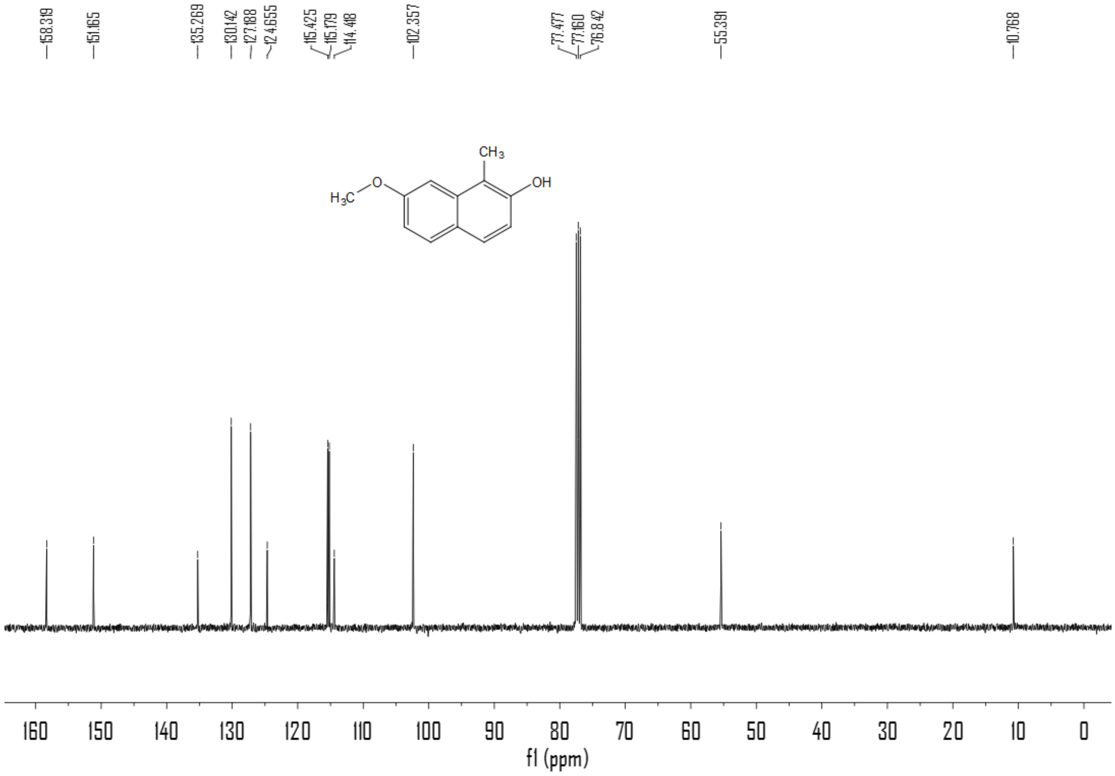
158.319
151.165
135.269
130.142
127.188
124.655
115.425
115.179
114.418
102.357
77.477
77.160
76.842
55.391
10.768
CH3
OH
H3C
O
f1 (ppm)

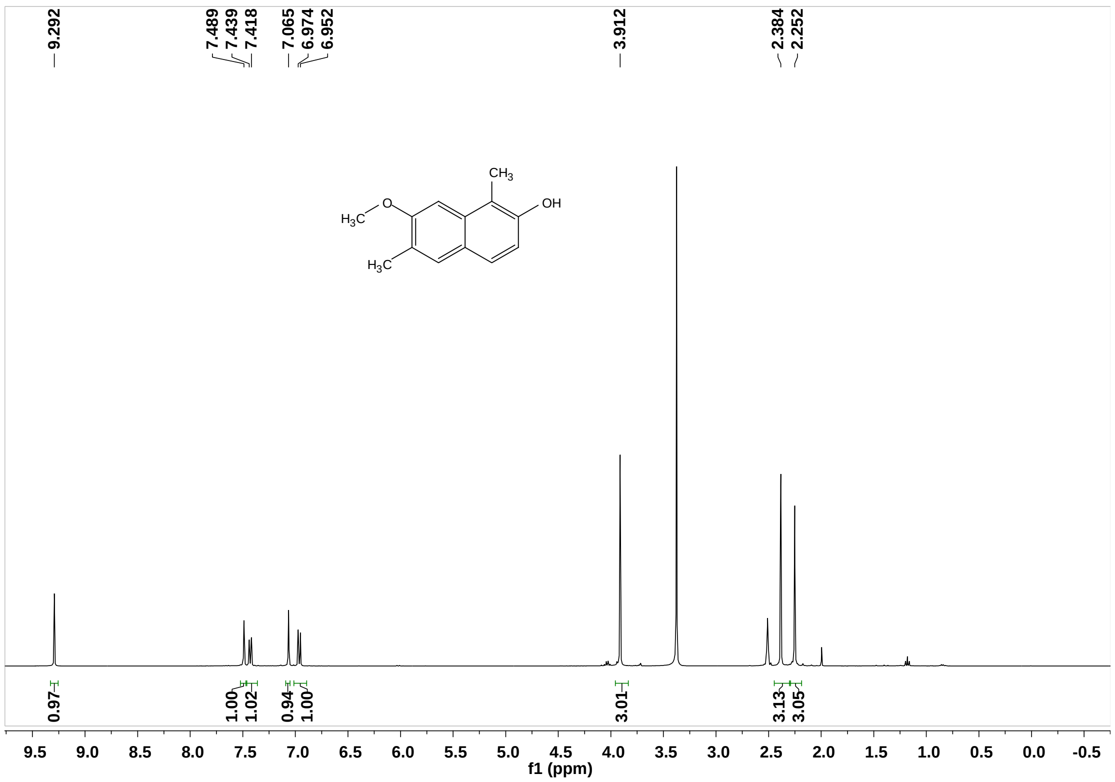

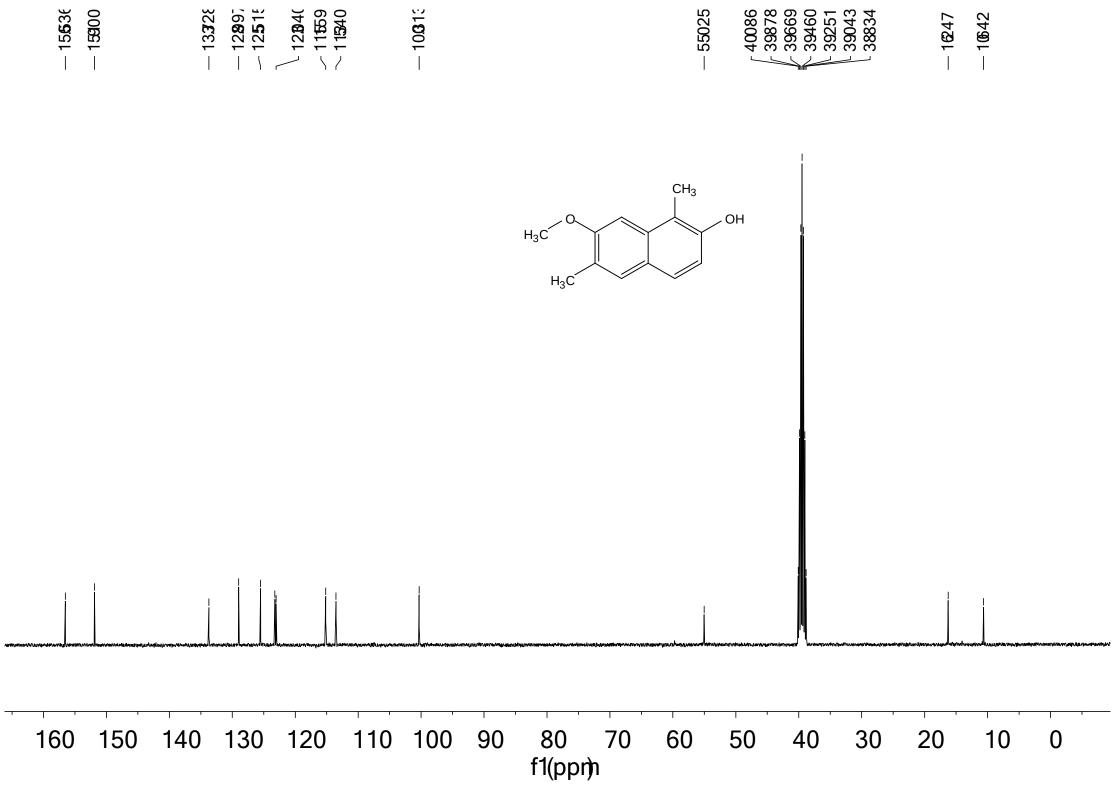

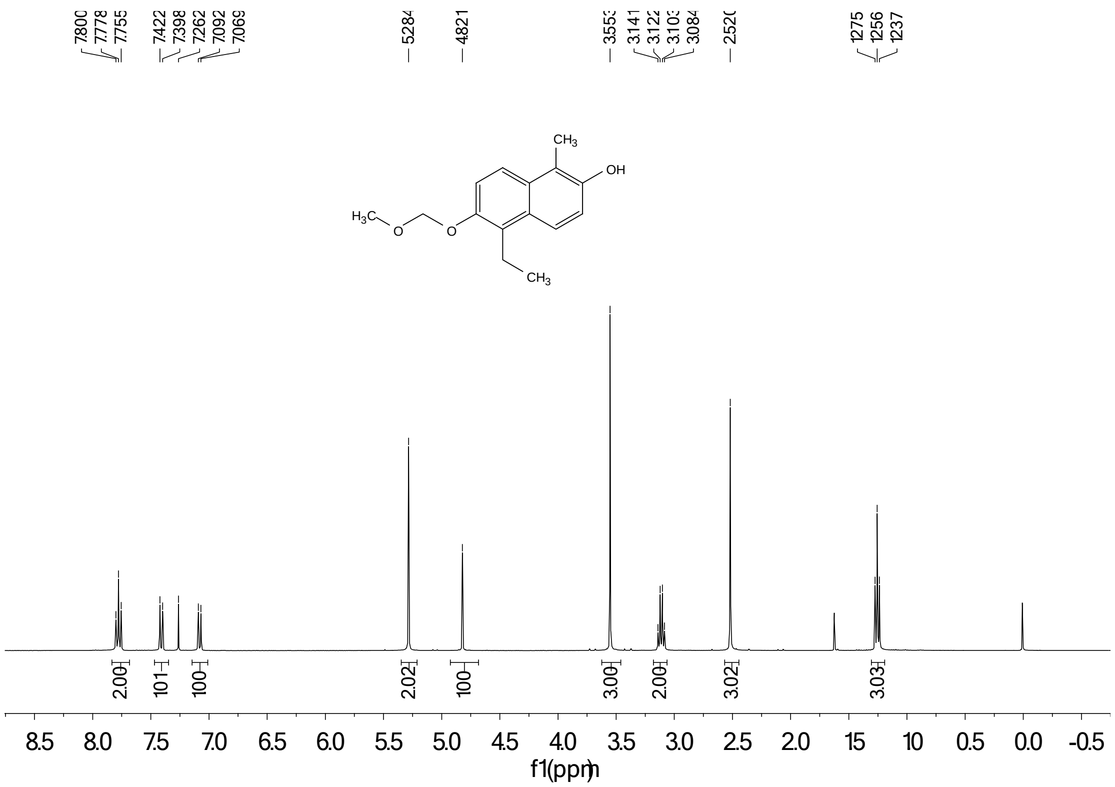

f1 (ppm)

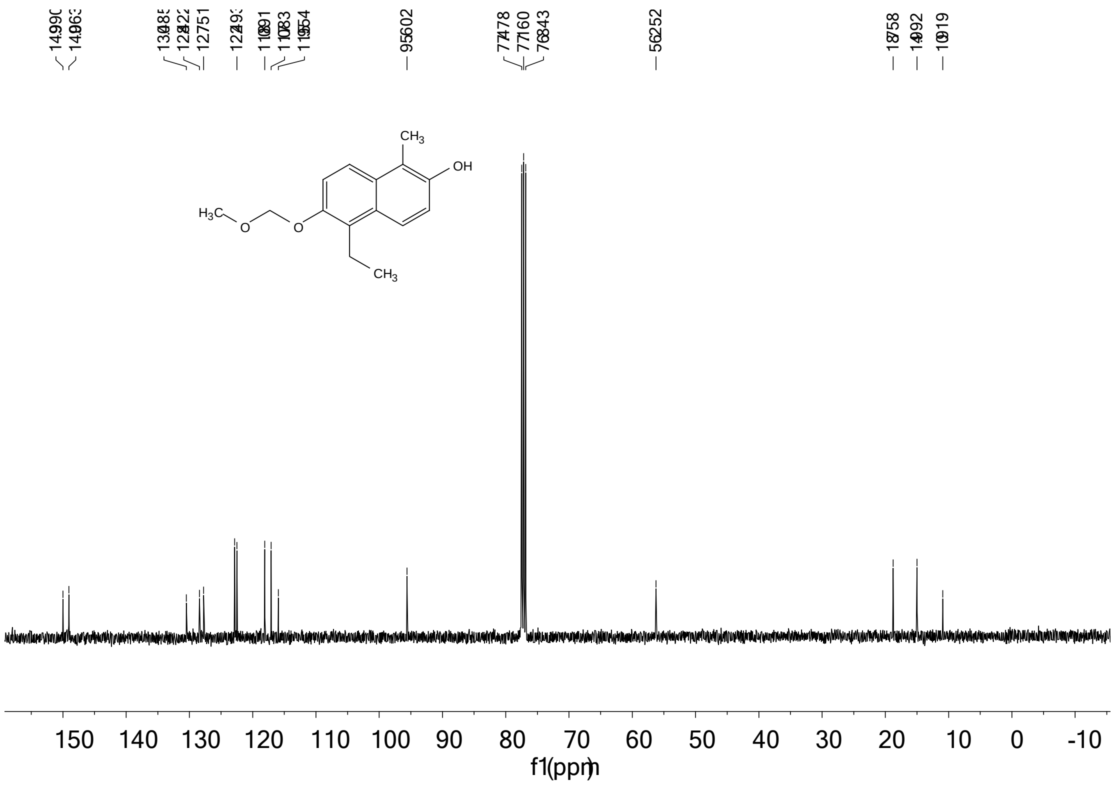

f1 (ppm)

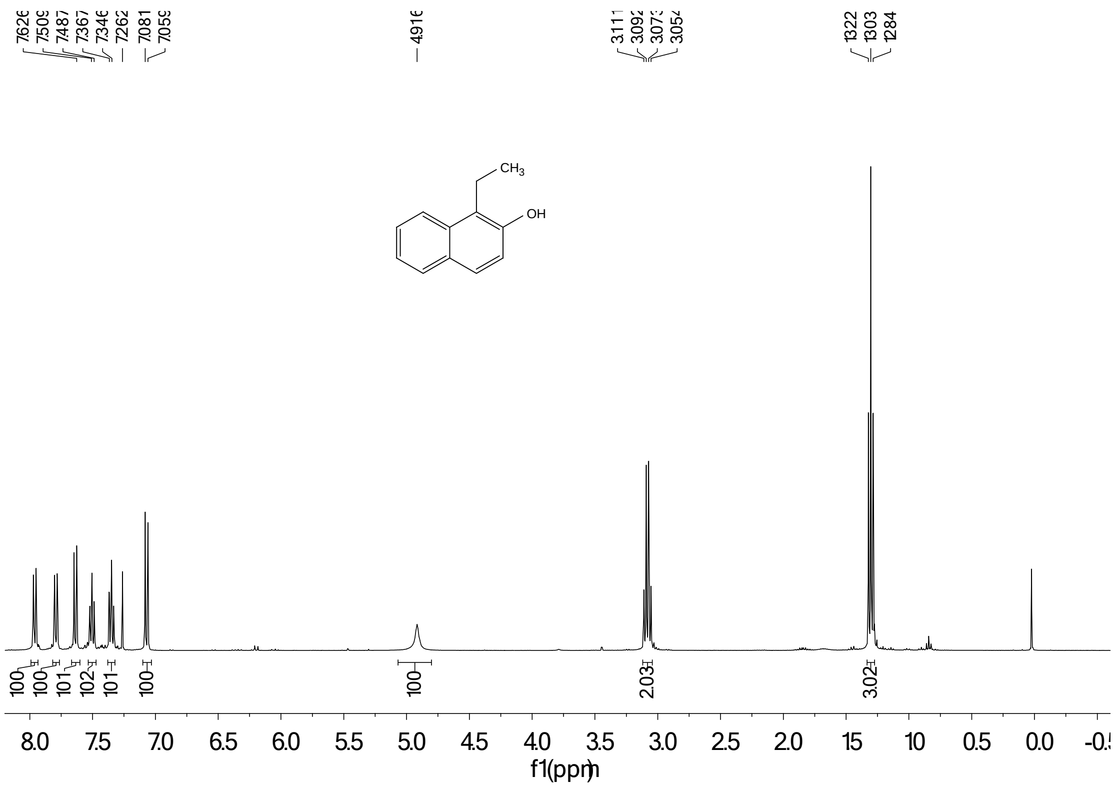

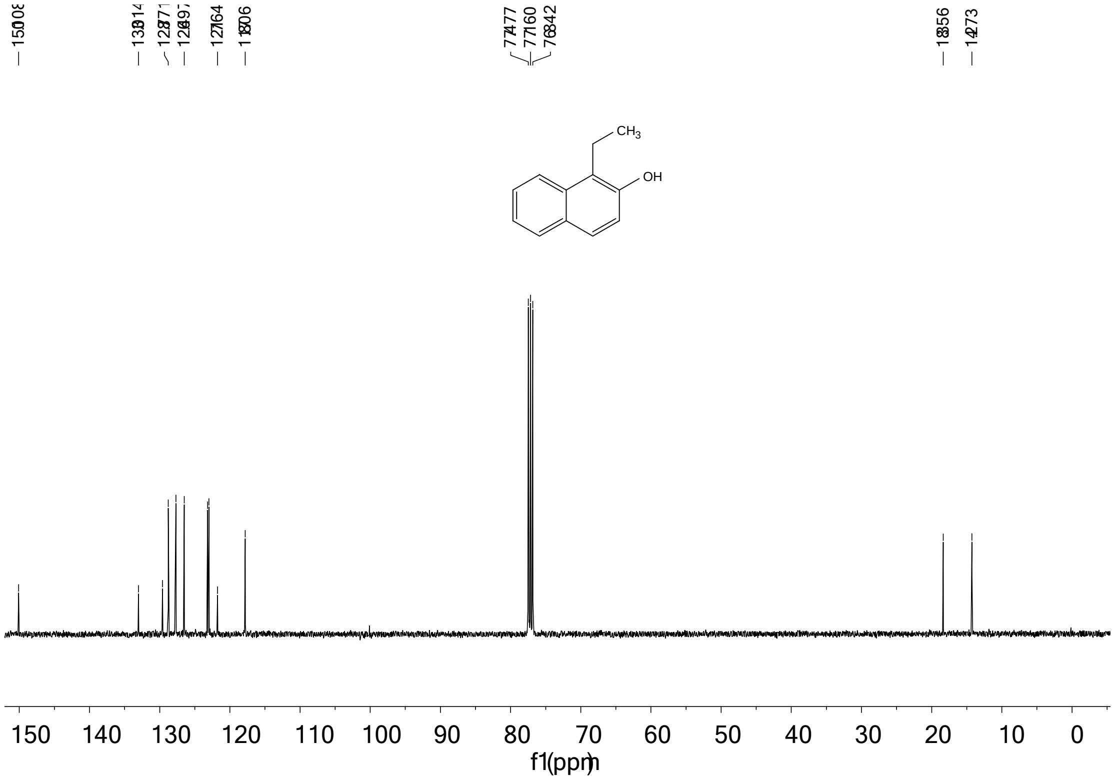

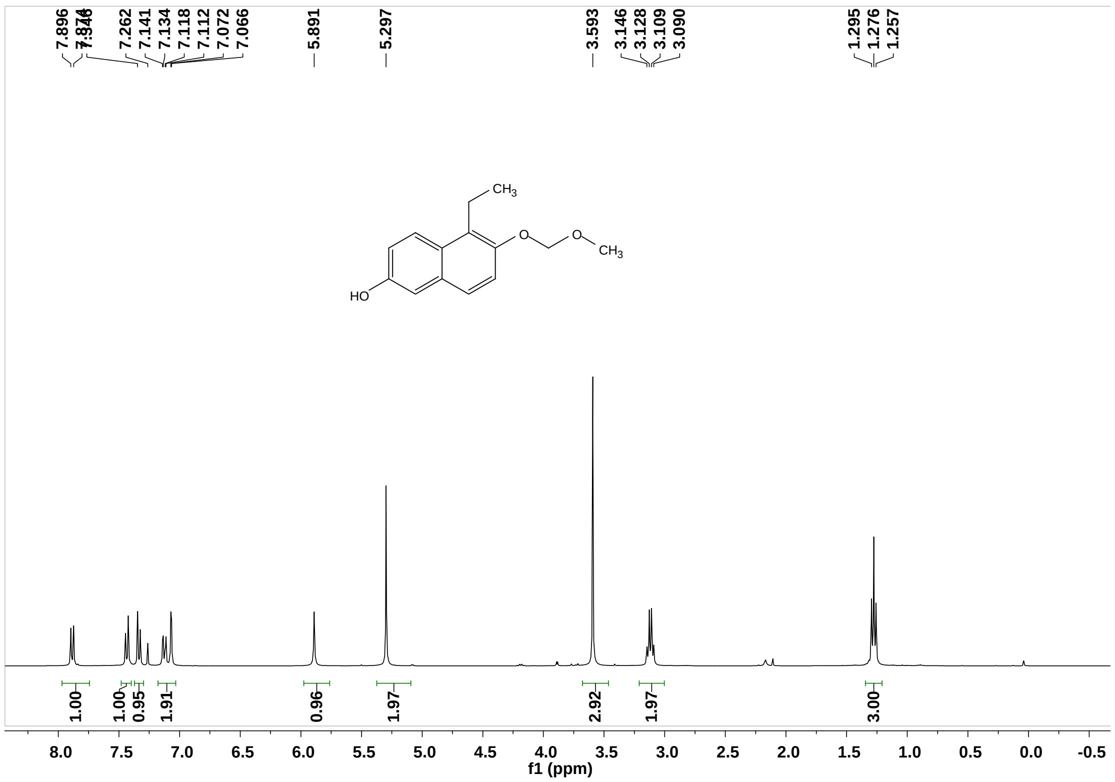

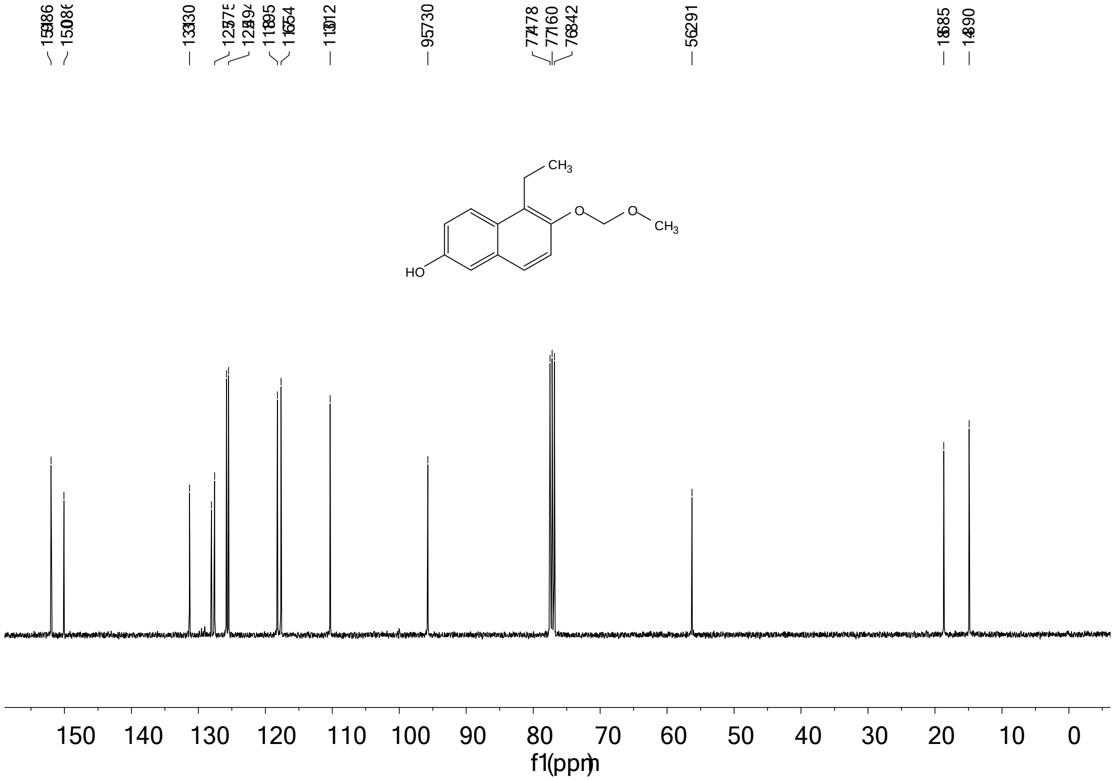

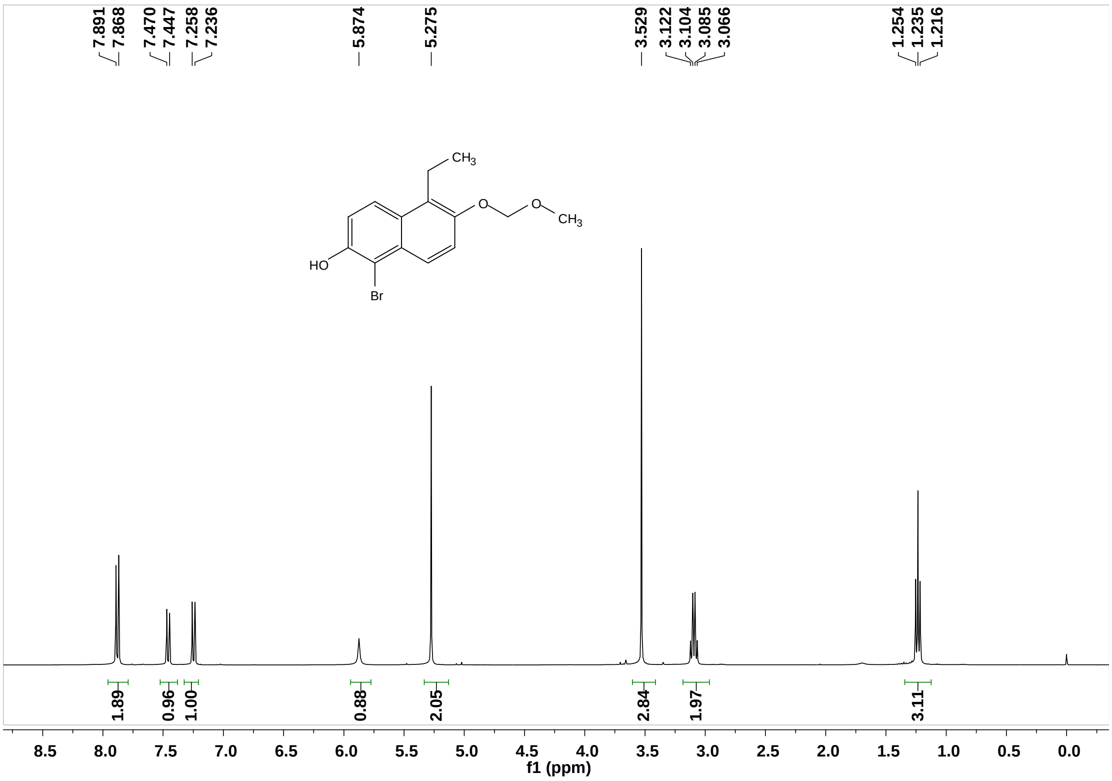
7.891
7.868
7.470
7.447
7.258
7.236
5.874
5.275
3.529
3.122
3.104
3.085
3.066
1.254
1.235
1.216
CH3
O
O
CH3
HO
Br
1.89
0.96
1.00
0.88
2.05
2.84
1.97
3.11
8.5
8.0
7.5
7.0
6.5
6.0
5.5
5.0
4.5
4.0
3.5
3.0
2.5
2.0
1.5
1.0
0.5
0.0
f1 (ppm)

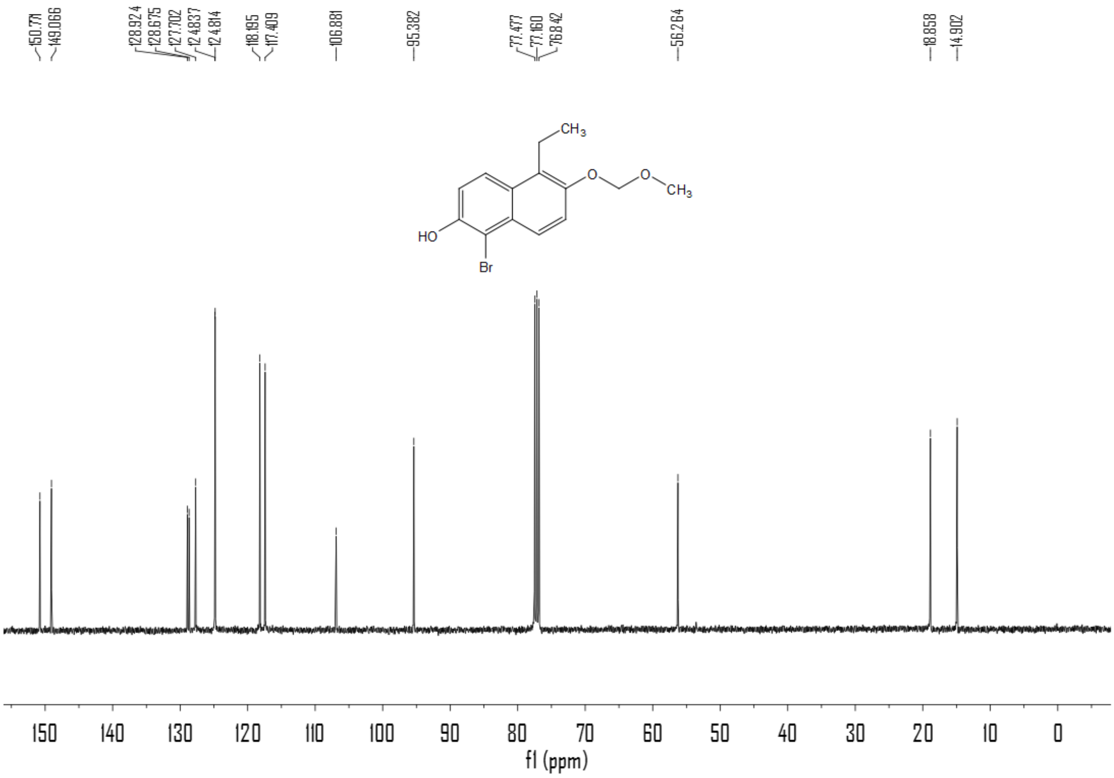
150.771
149.066
128.924
128.675
127.702
124.837
124.814
118.195
117.409
106.881
95.382
77.477
77.160
76.842
56.264
18.858
14.902
CH3
O
O
CH3
HO
Br
150
140
130
120
110
100
90
80
70
60
50
40
30
20
10
0
f1 (ppm)

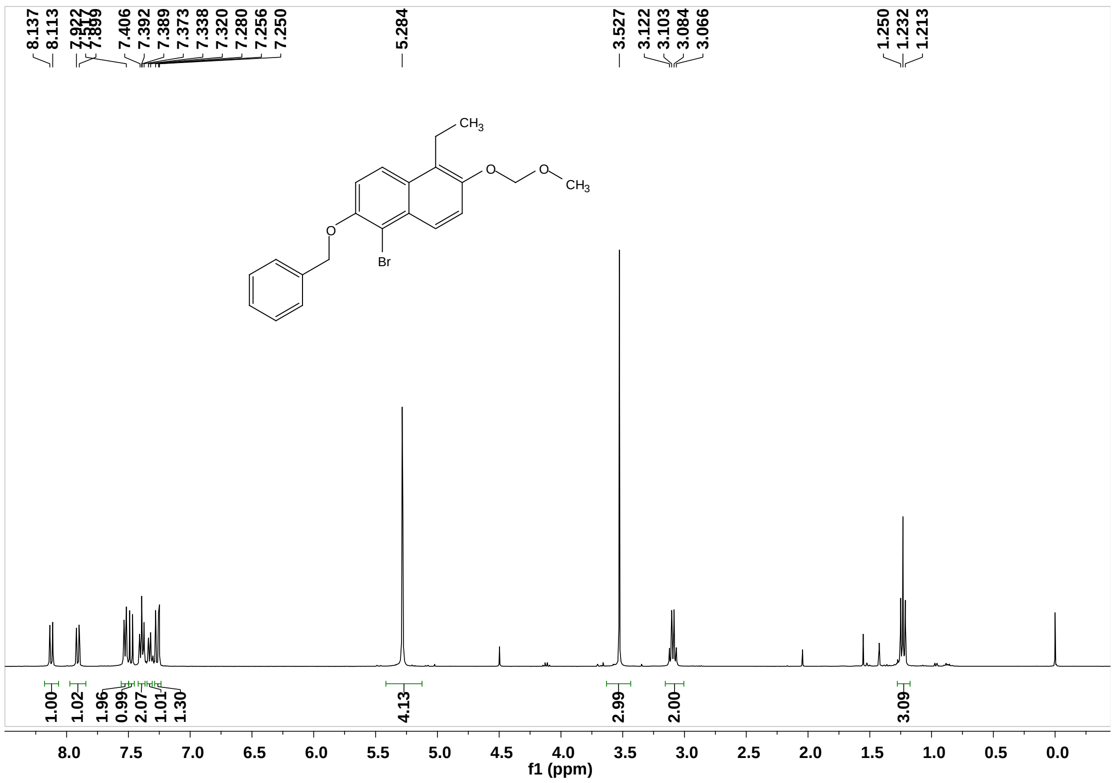

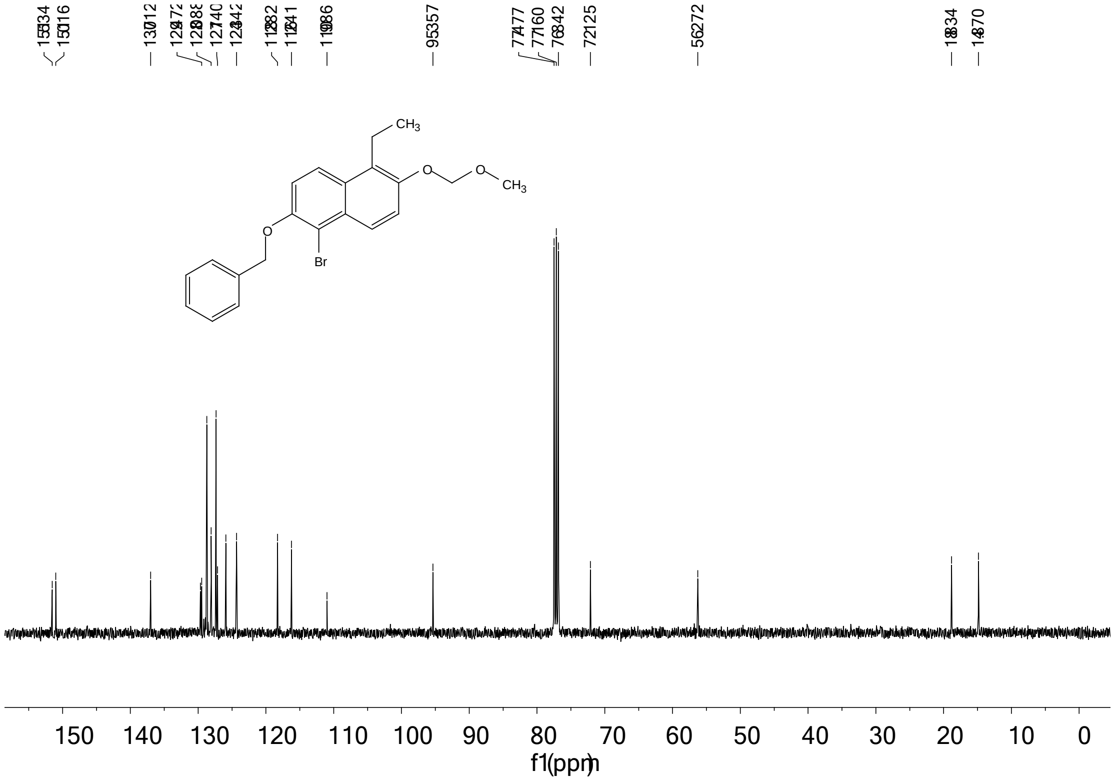

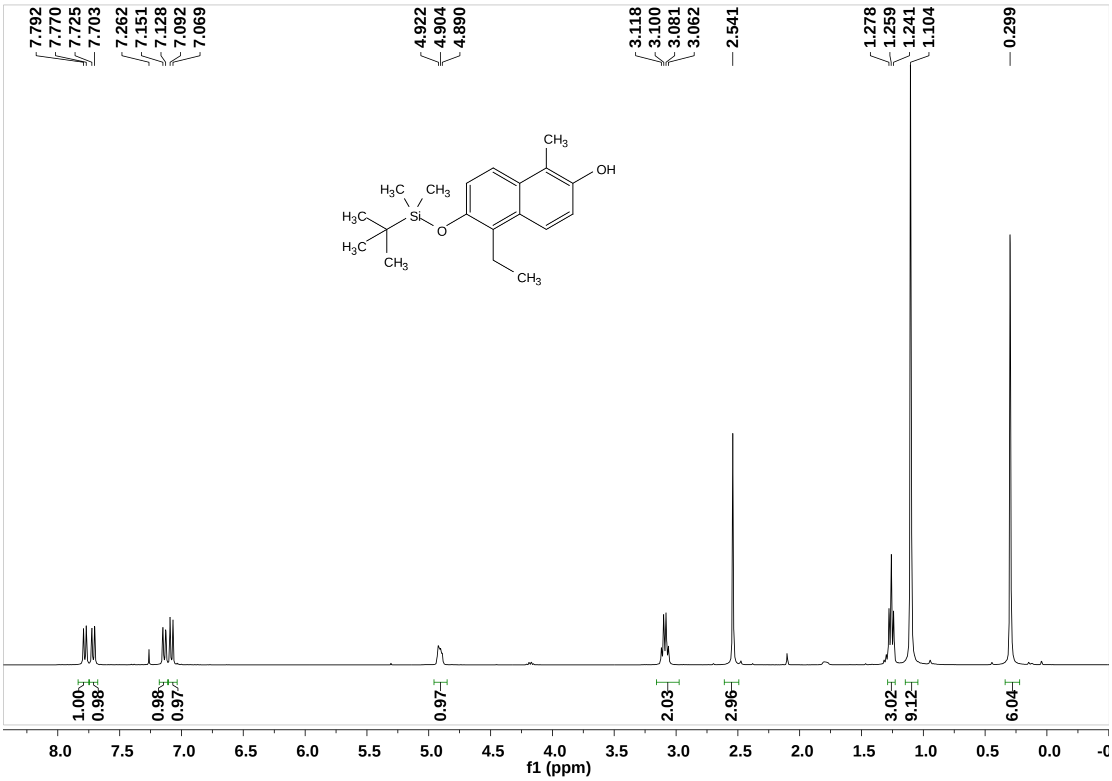
7.792
7.770
7.725
7.703
7.262
7.151
7.128
7.092
7.069
4.922
4.904
4.890
3.118
3.100
3.081
3.062
2.541
1.278
1.259
1.241
1.104
0.299
CH3
OH
H3C
CH3
Si
O
H3C
H3C
CH3
CH3
1.00
0.98
0.98
0.97
0.97
2.03
2.96
3.02
9.12
6.04
8.0 7.5 7.0 6.5 6.0 5.5 5.0 4.5 4.0 3.5 3.0 2.5 2.0 1.5 1.0 0.5 0.0
f1 (ppm)

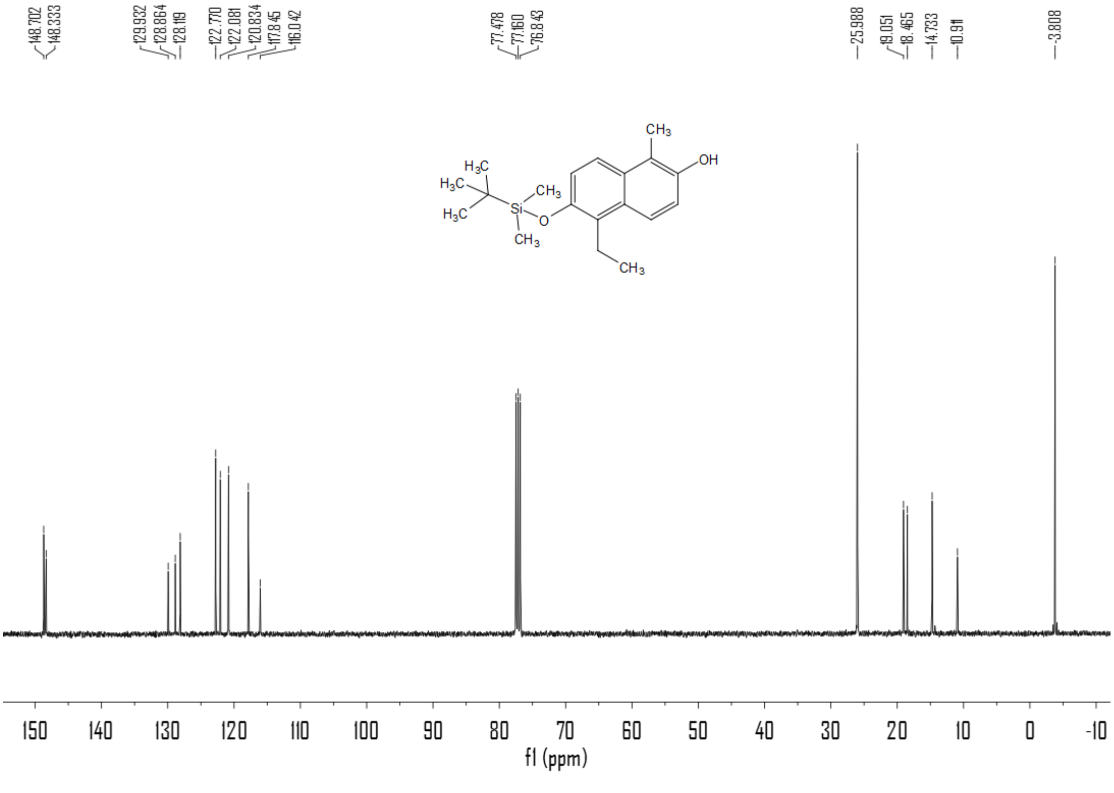
148.702
148.333
129.932
128.864
128.119
122.770
122.081
120.834
117.845
116.042
77.478
77.160
76.843
25.988
19.051
18.465
14.733
10.911
-3.808
CH3
OH
H3C
H3C
H3C
CH3
Si
O
CH3
CH3
150 140 130 120 110 100 90 80 70 60 50 40 30 20 10 0 -10
f1 (ppm)

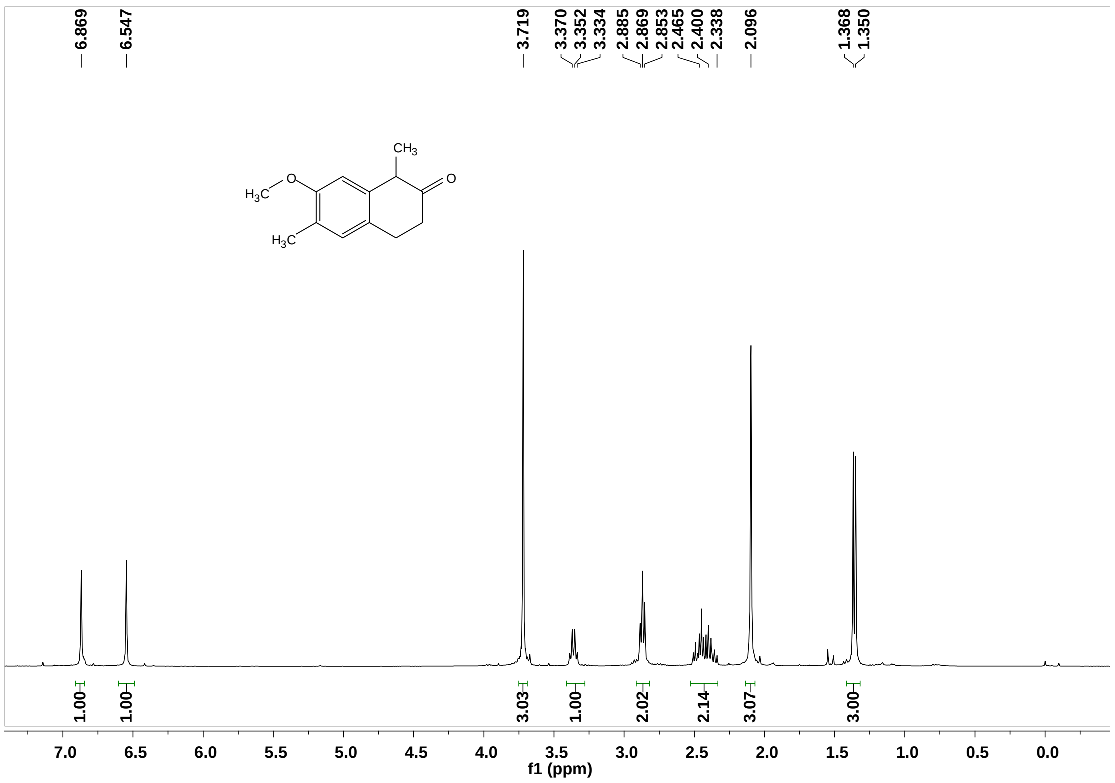

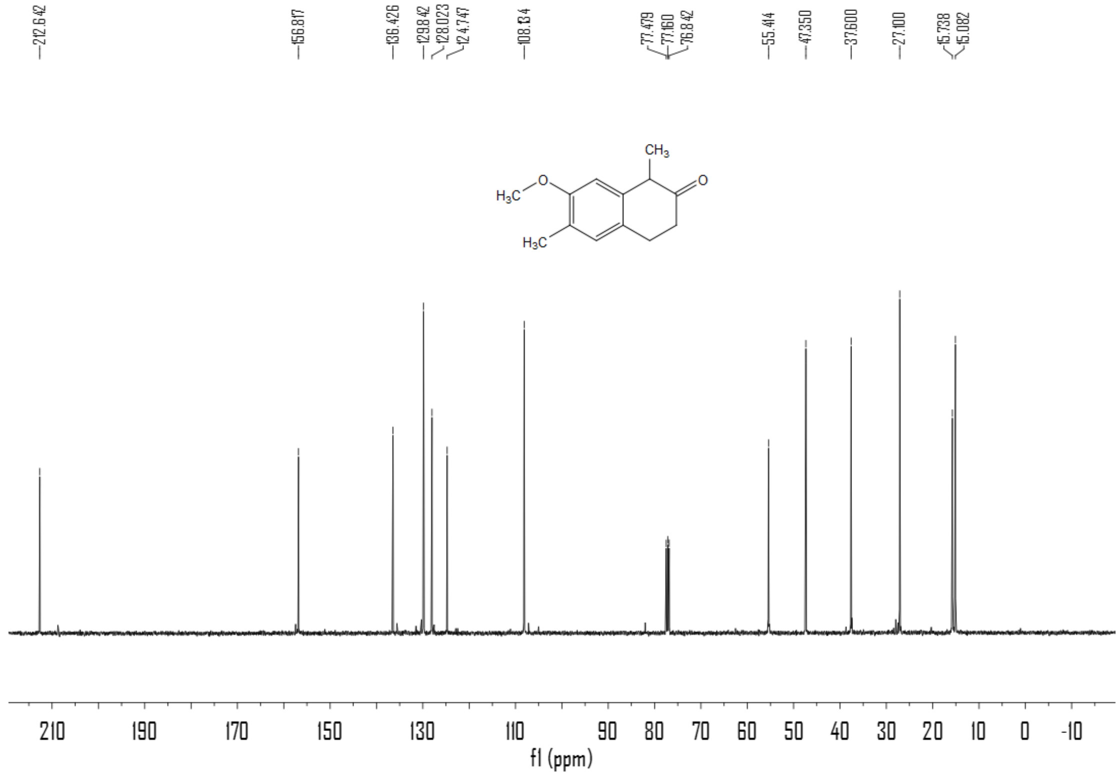

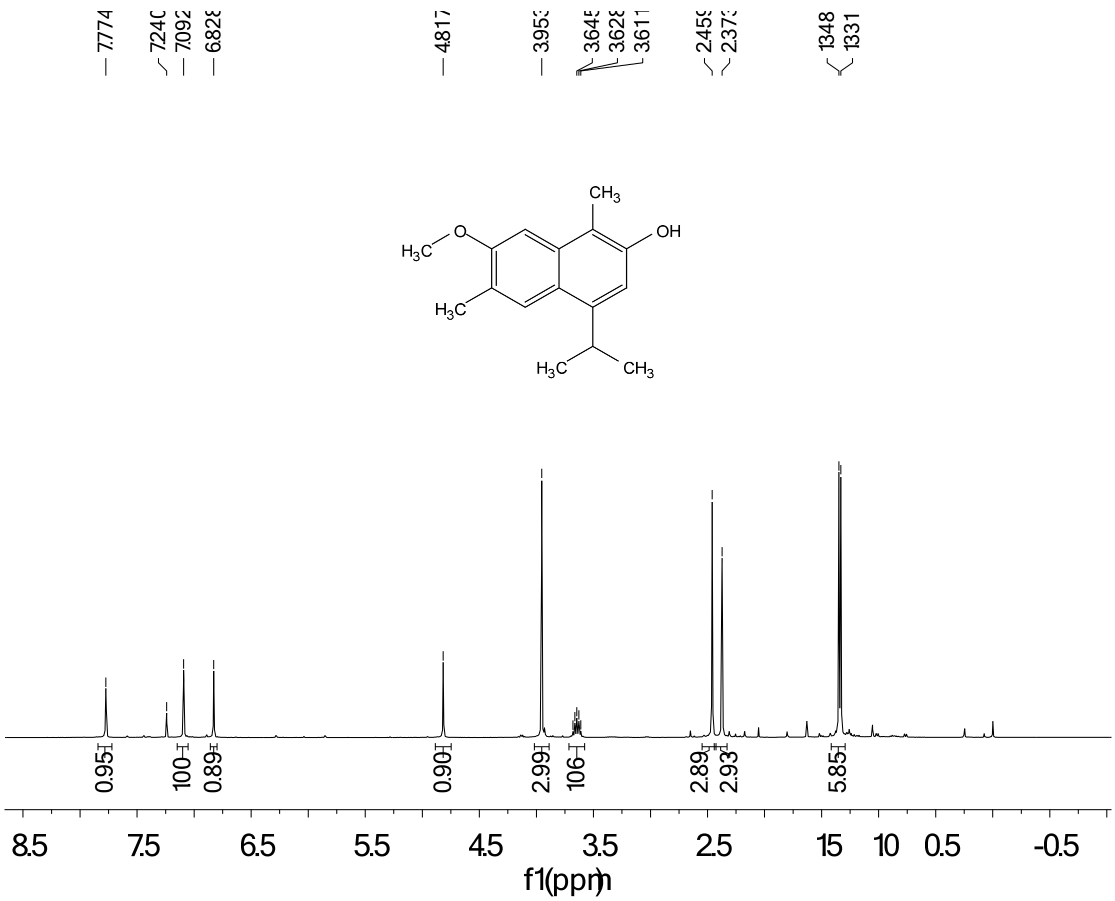

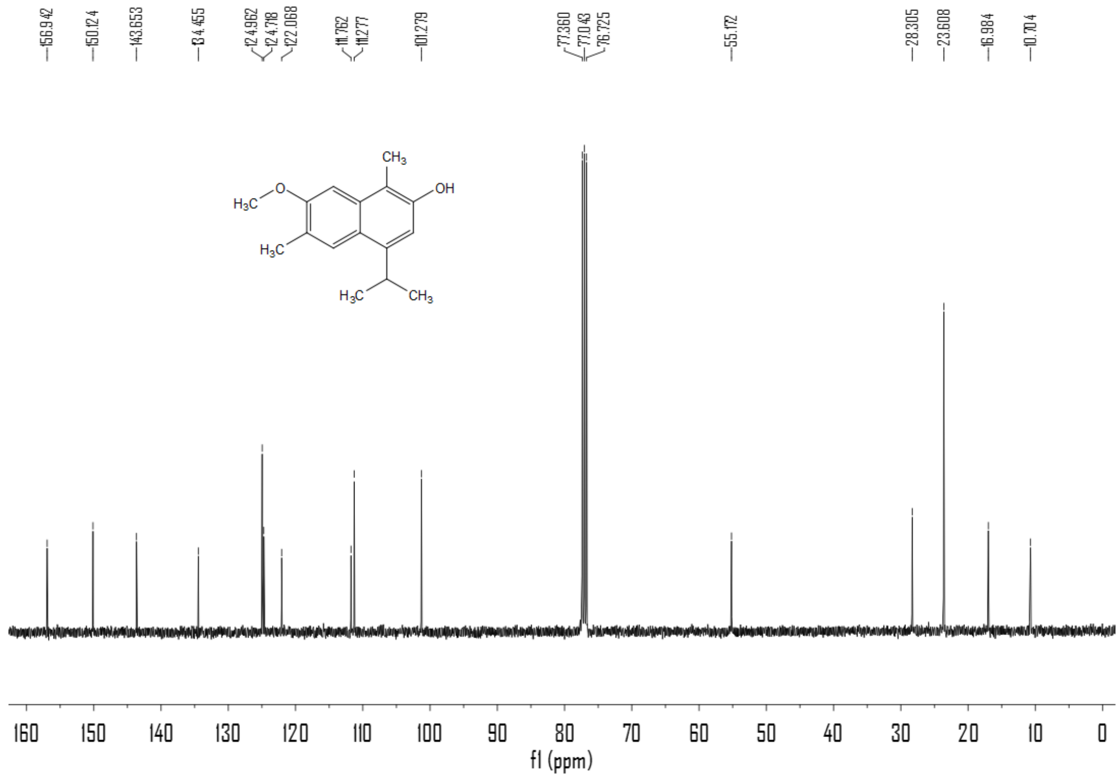

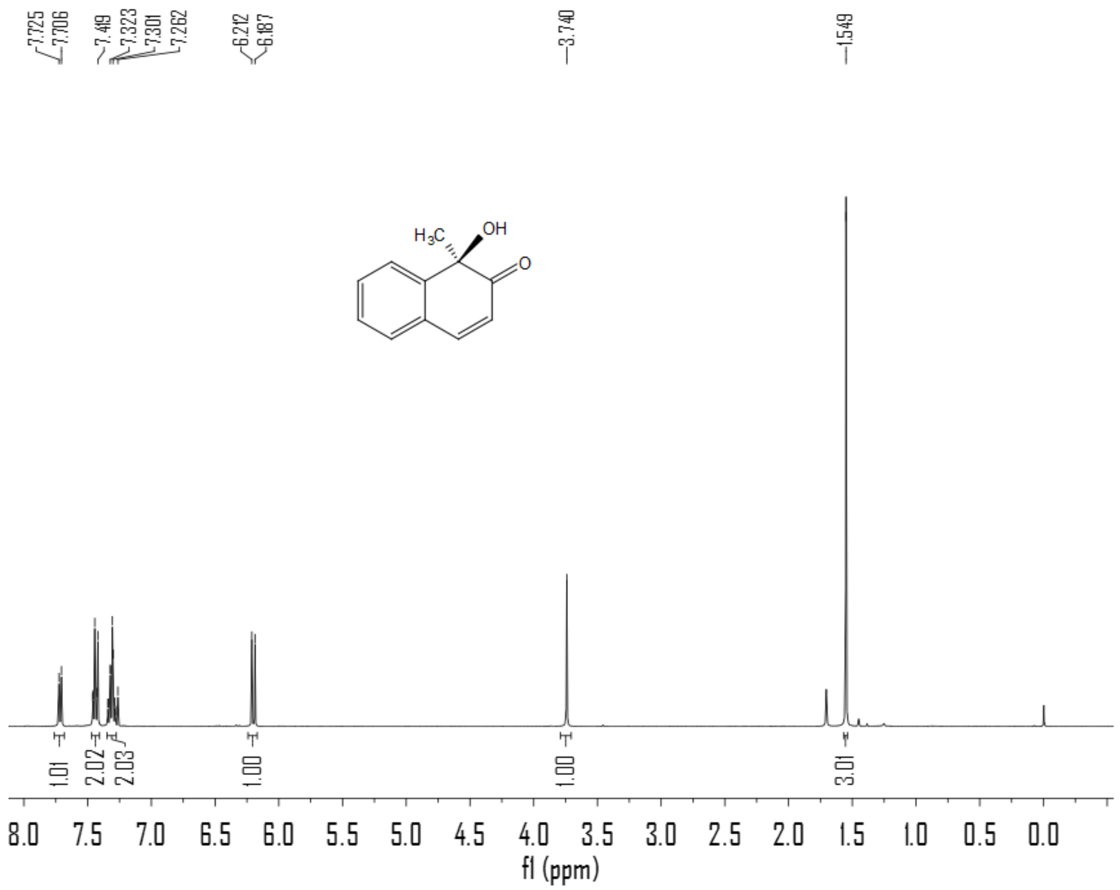

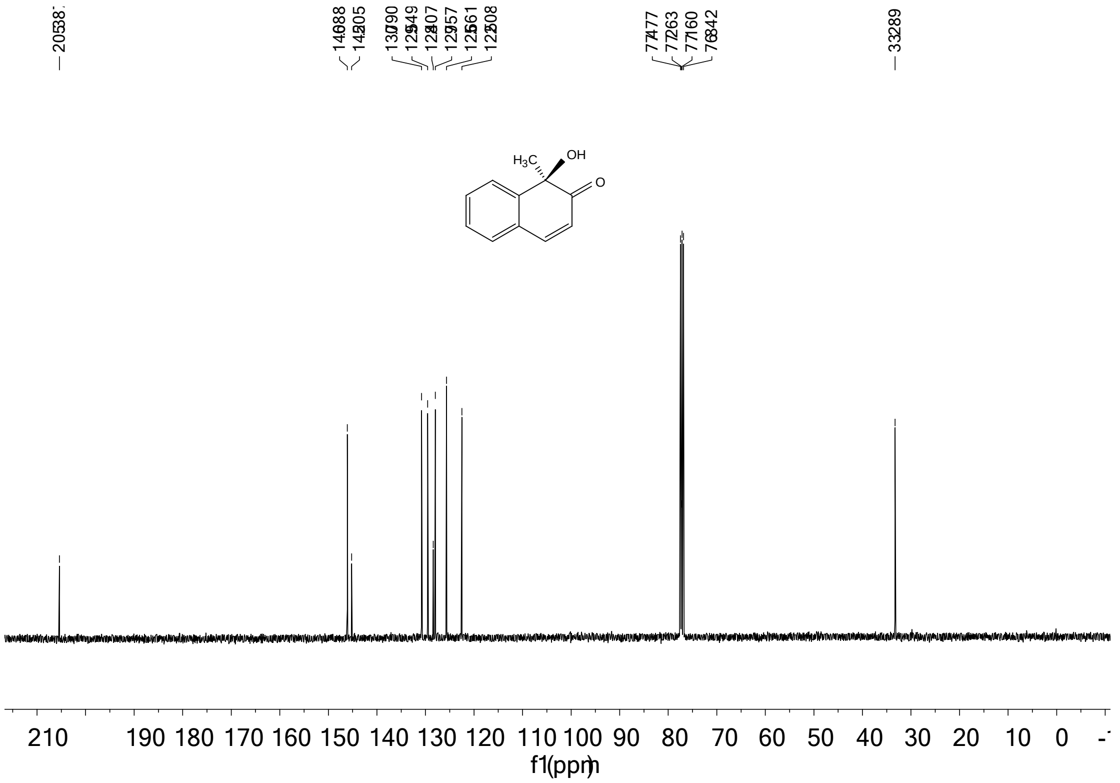

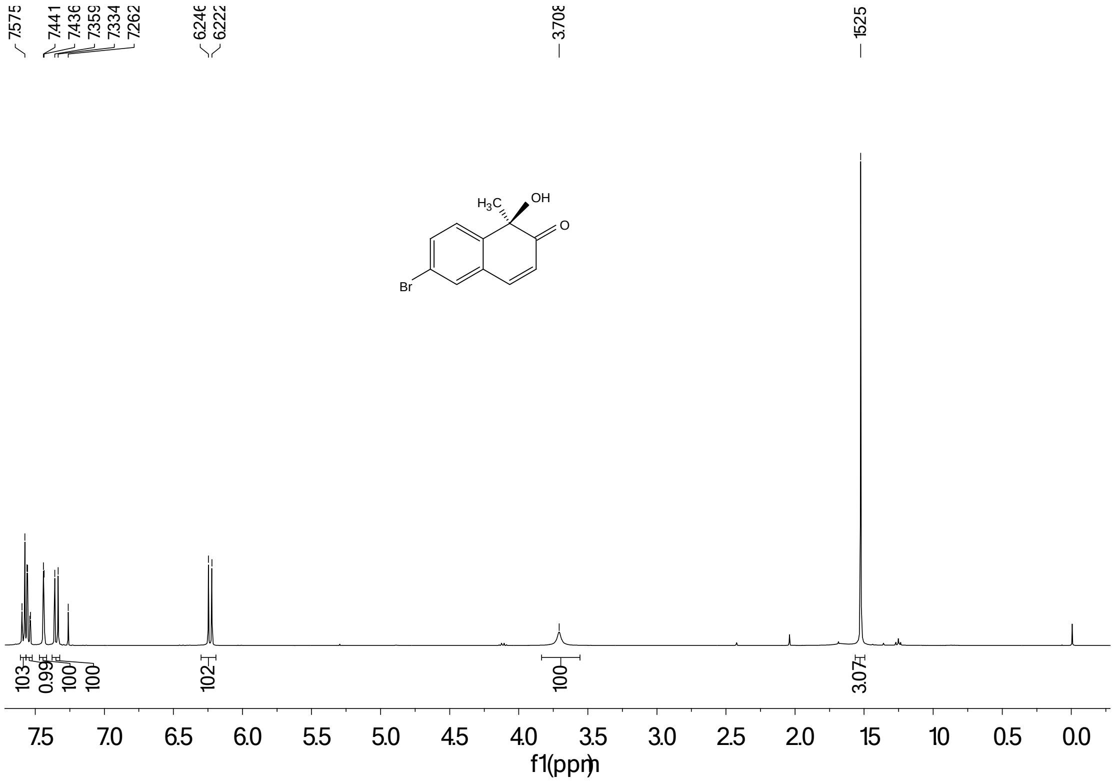

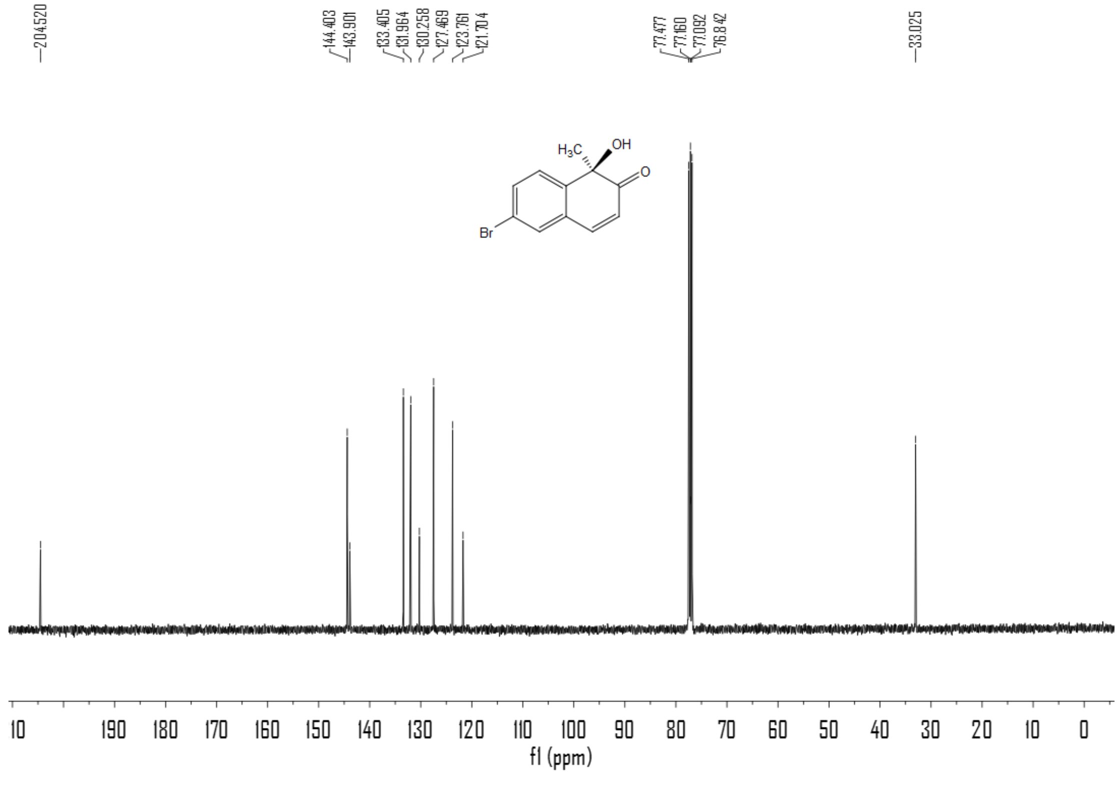

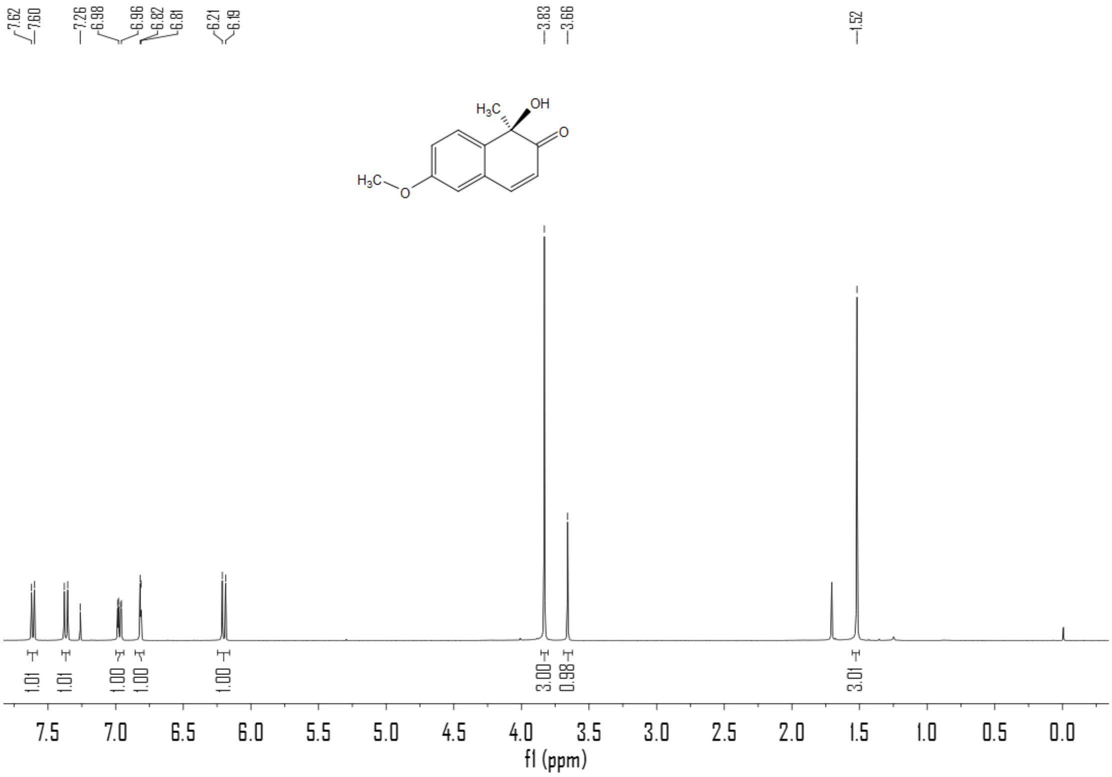

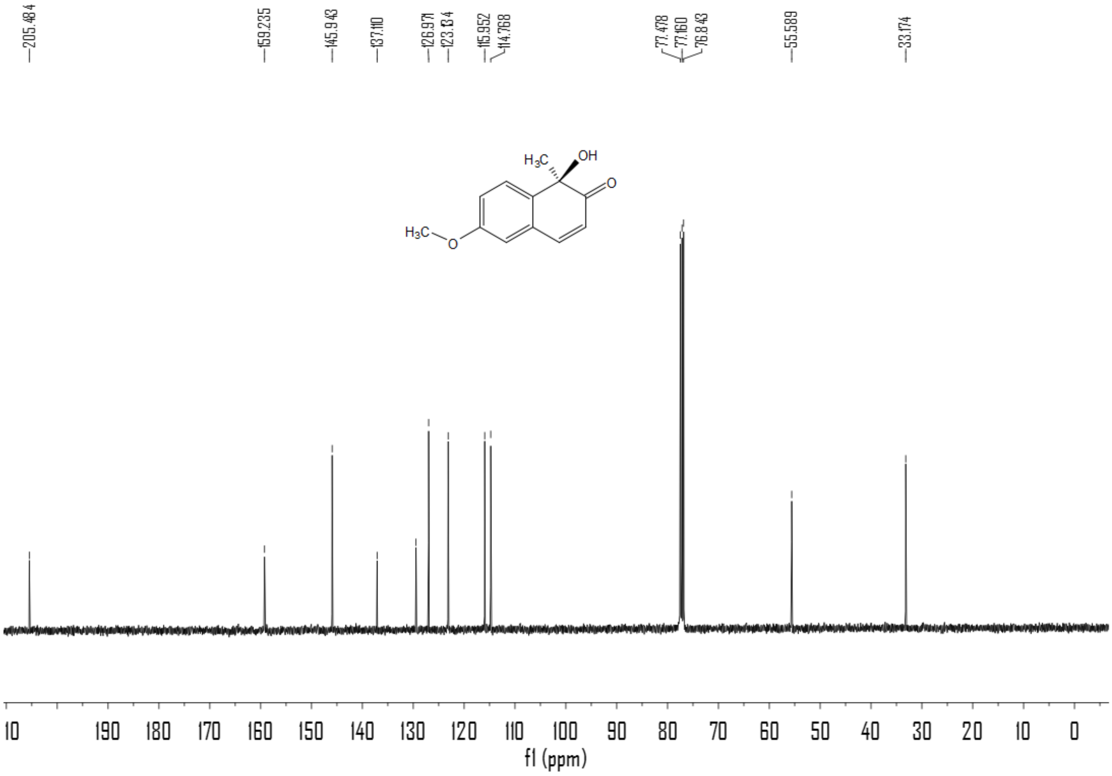

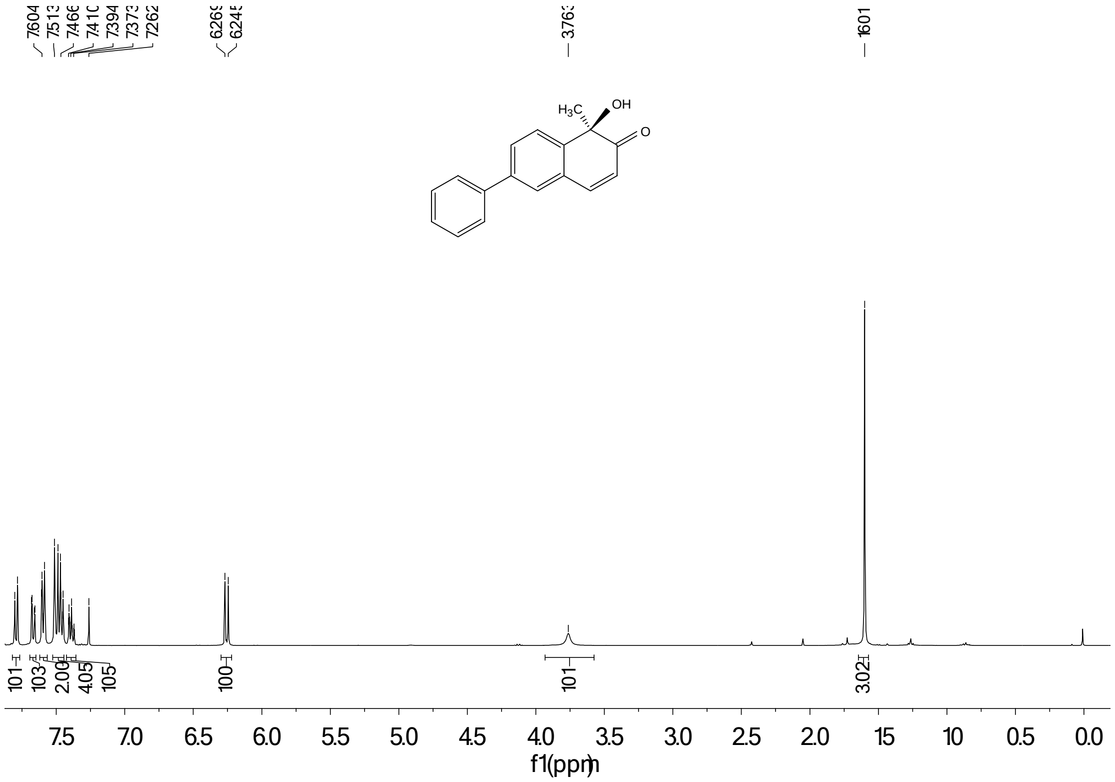

13.

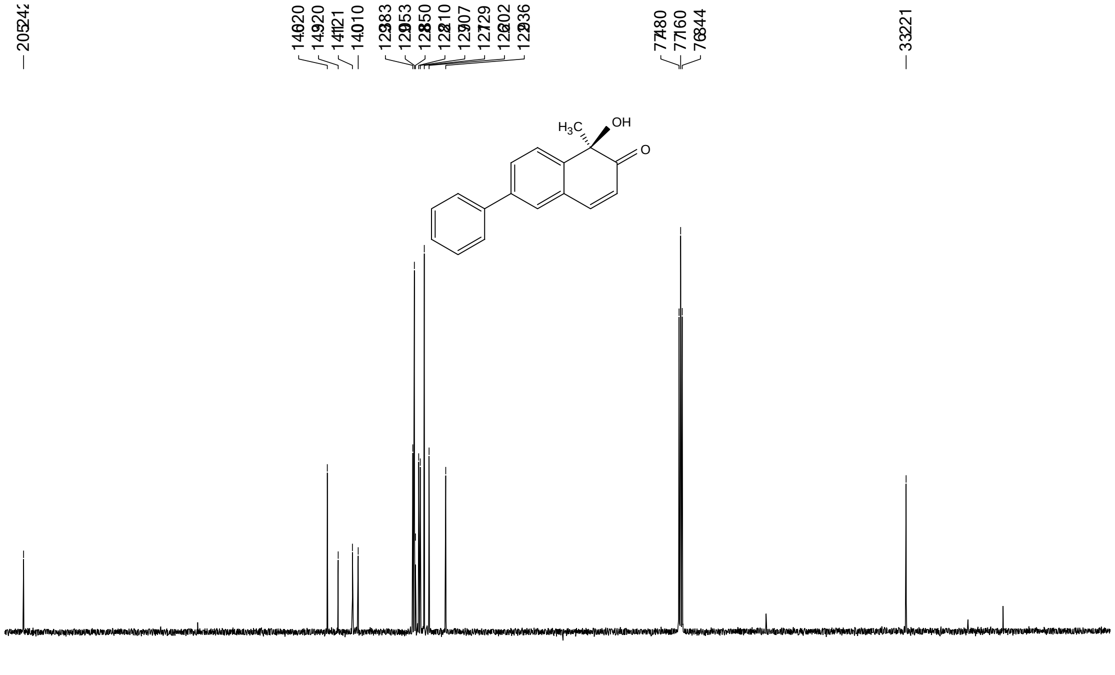

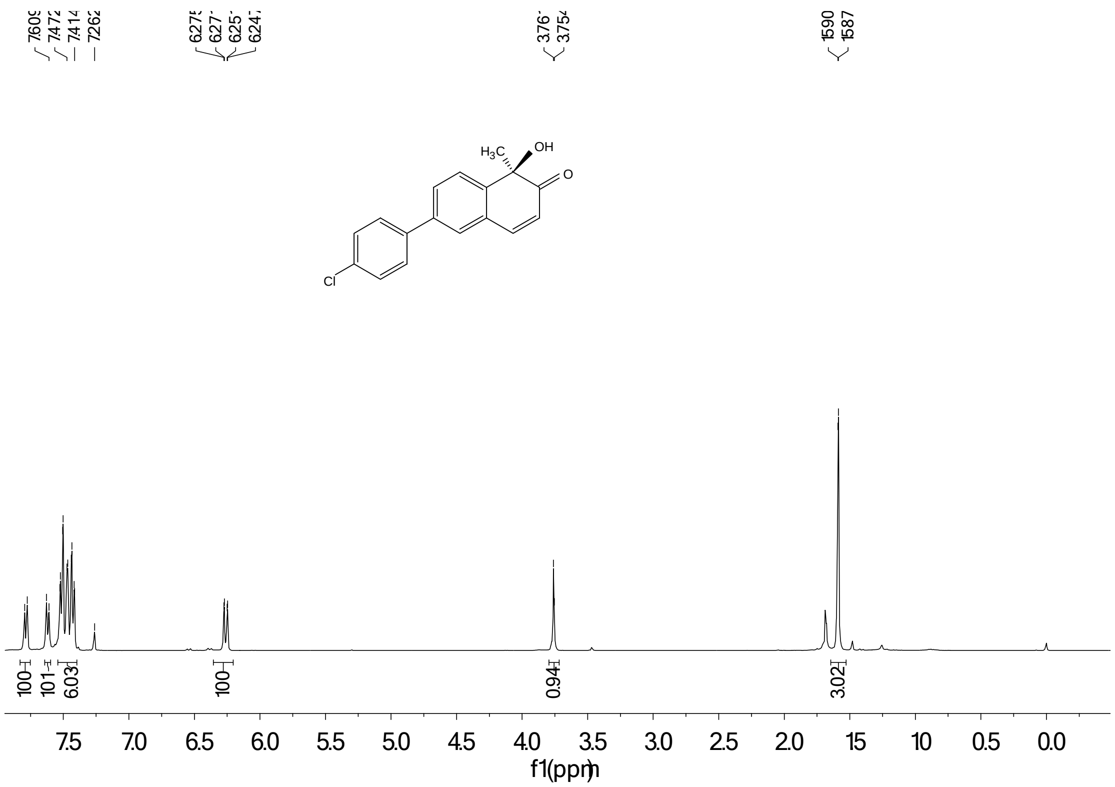

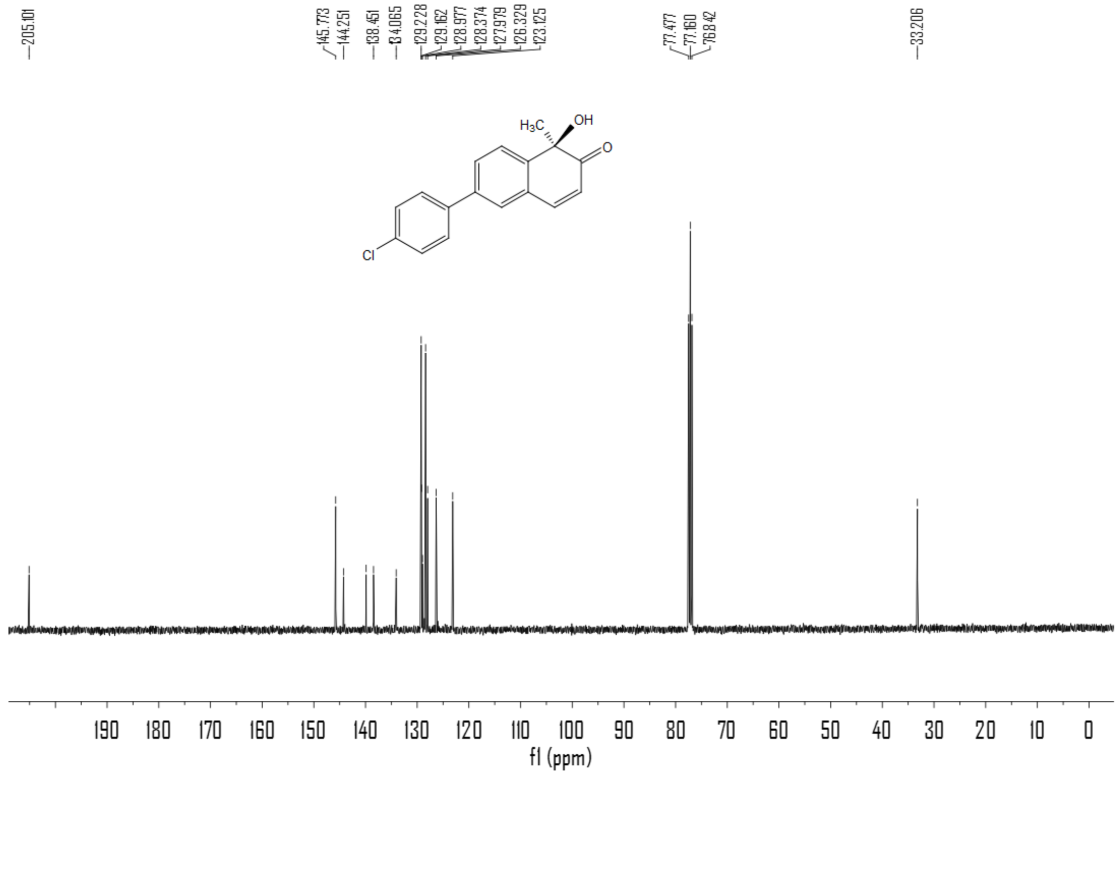

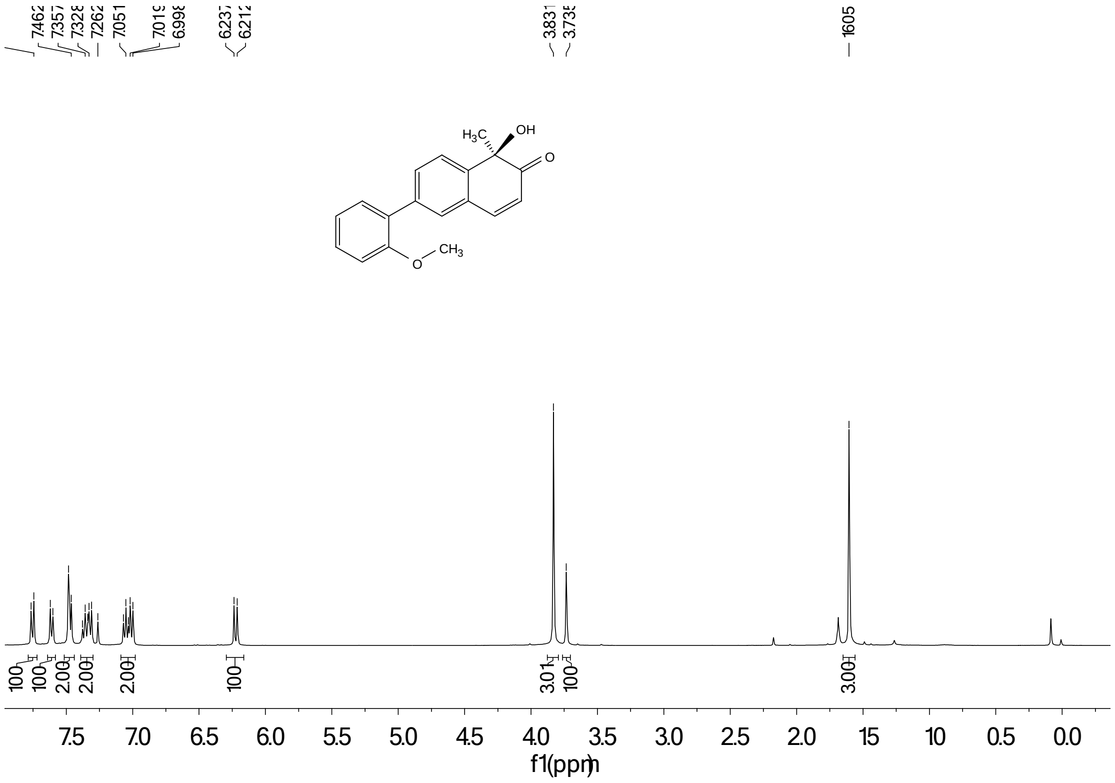

f1 (ppm)

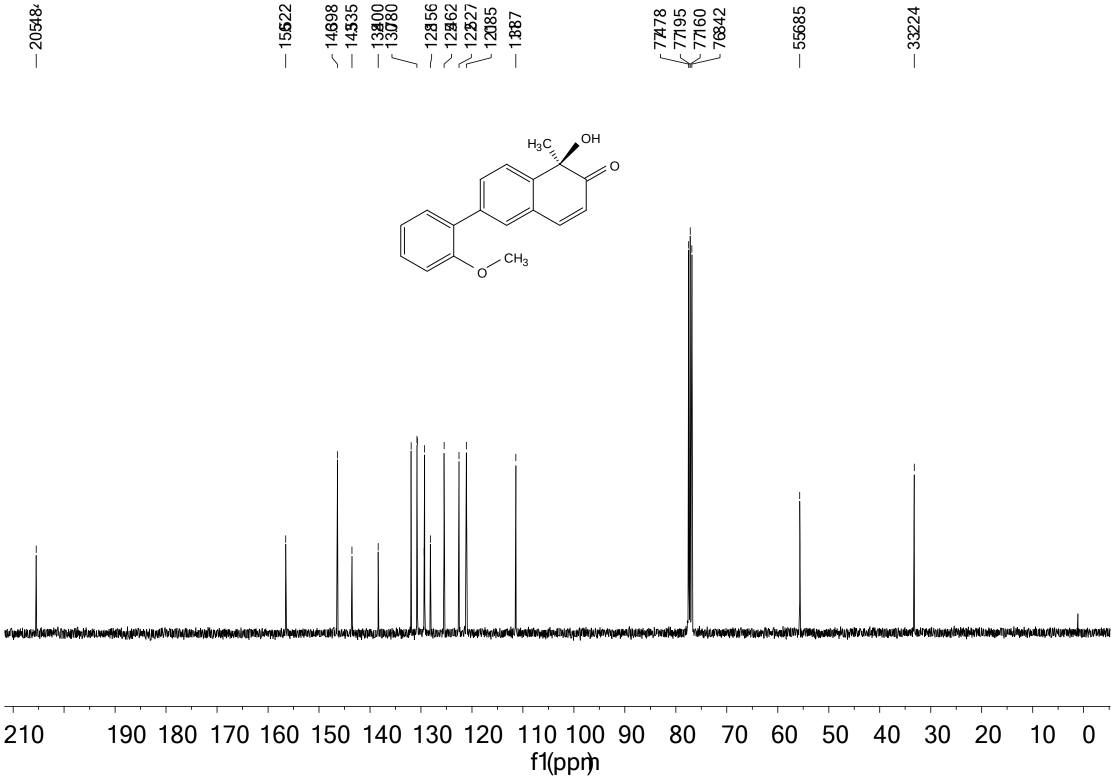

f1 (ppm)

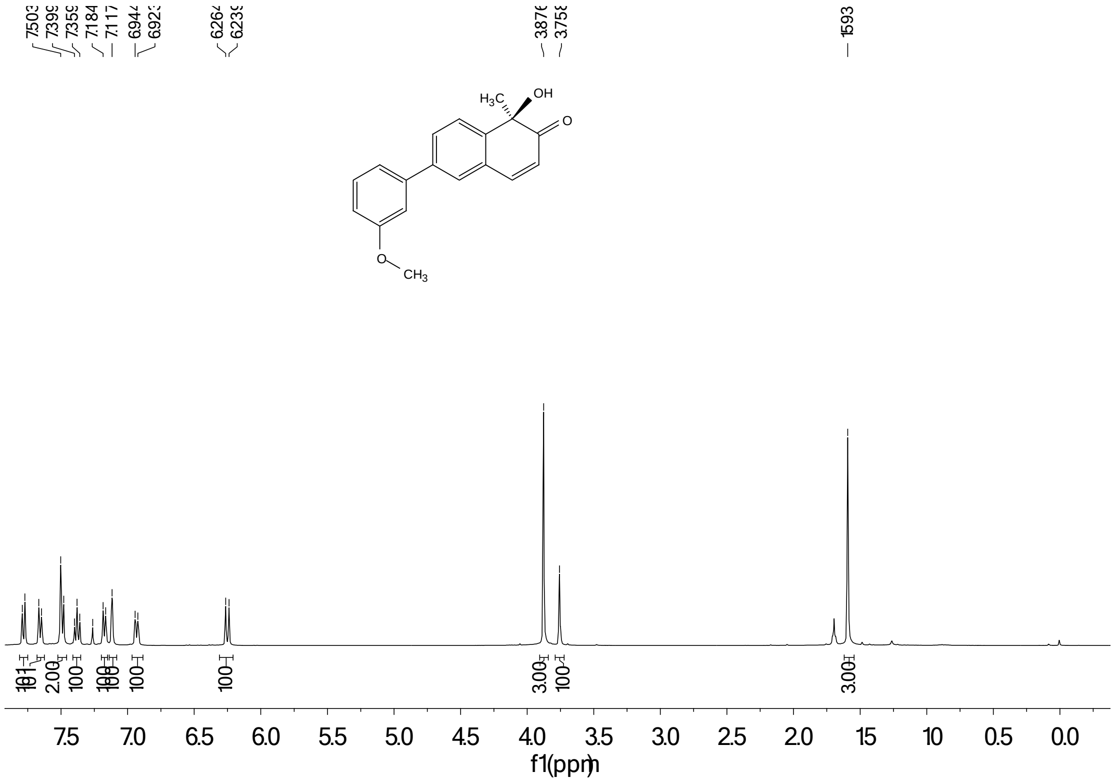
H3C
OH
O
CH3
f1 (ppm)

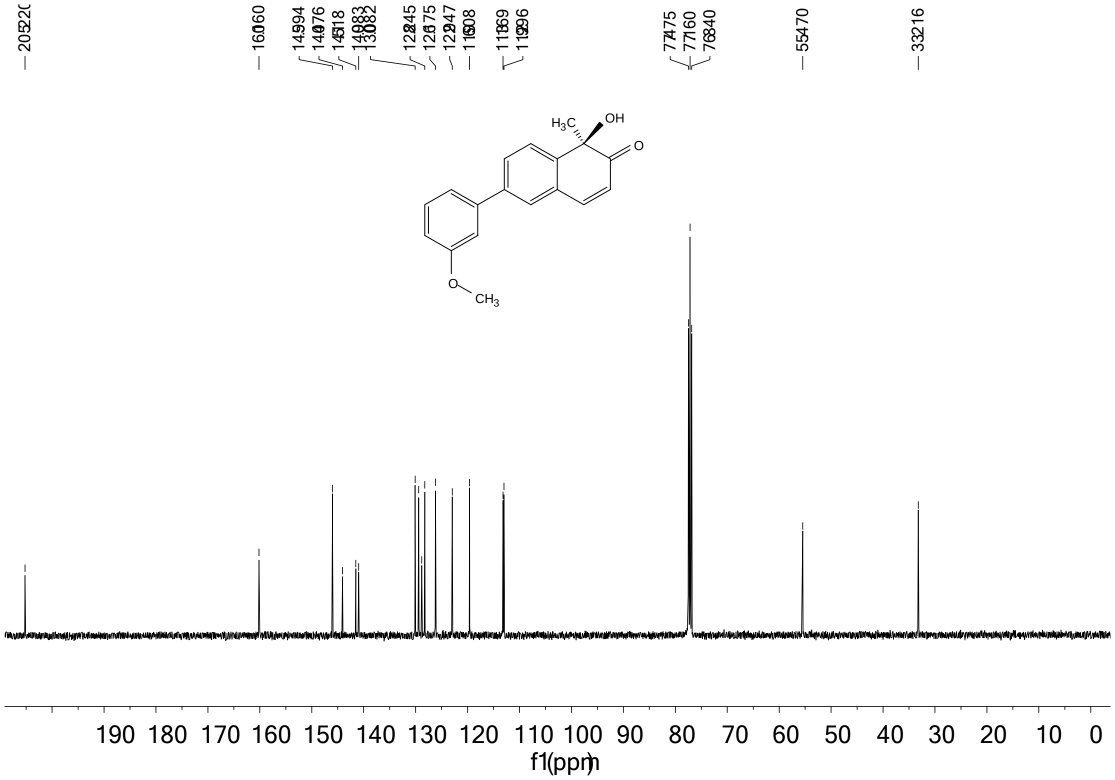
H3C
OH
O
CH3
f1 (ppm)

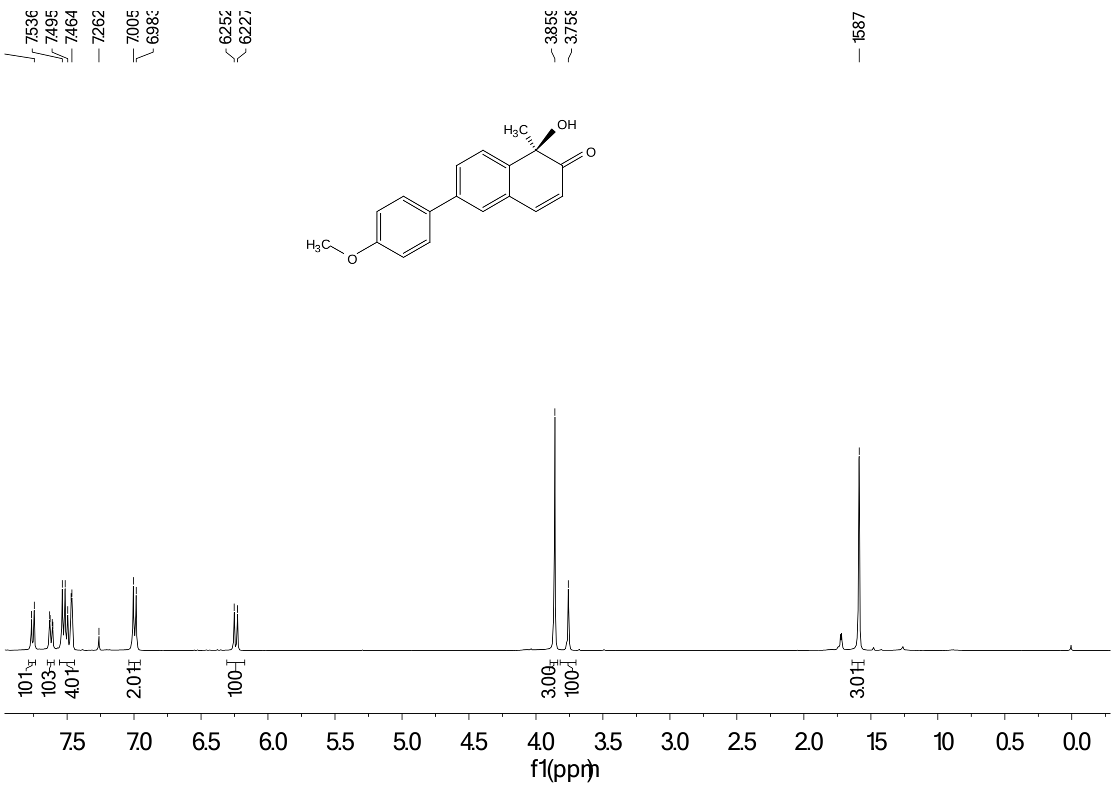

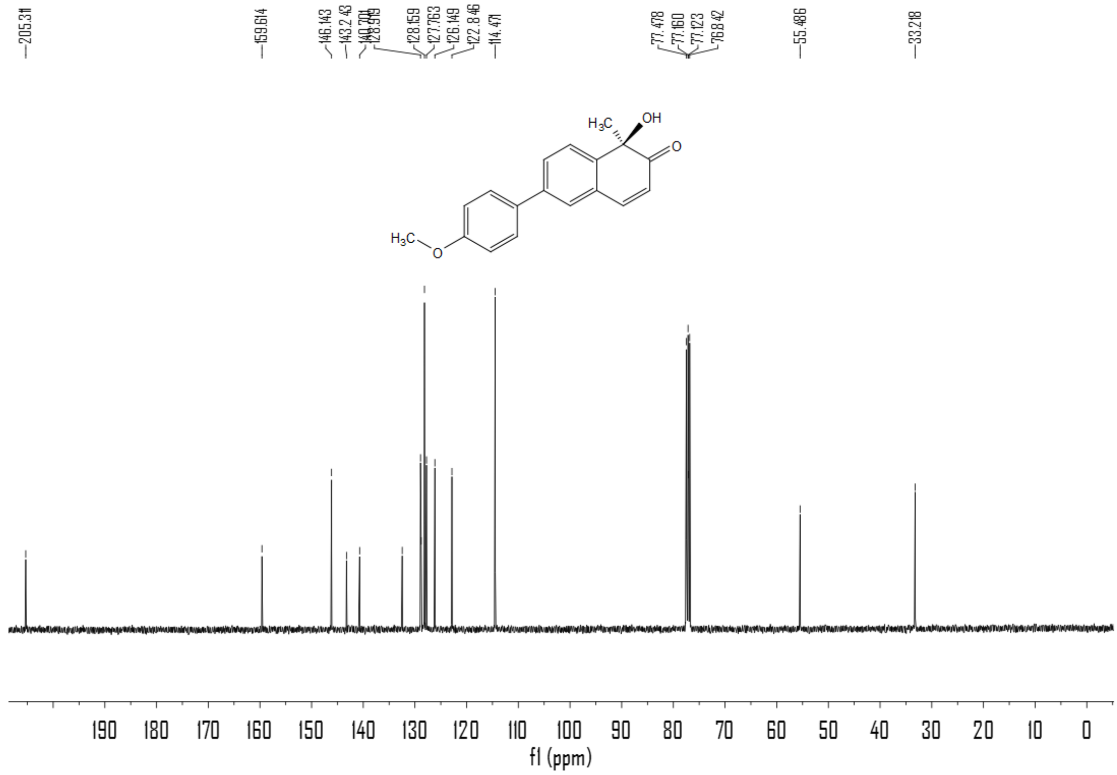

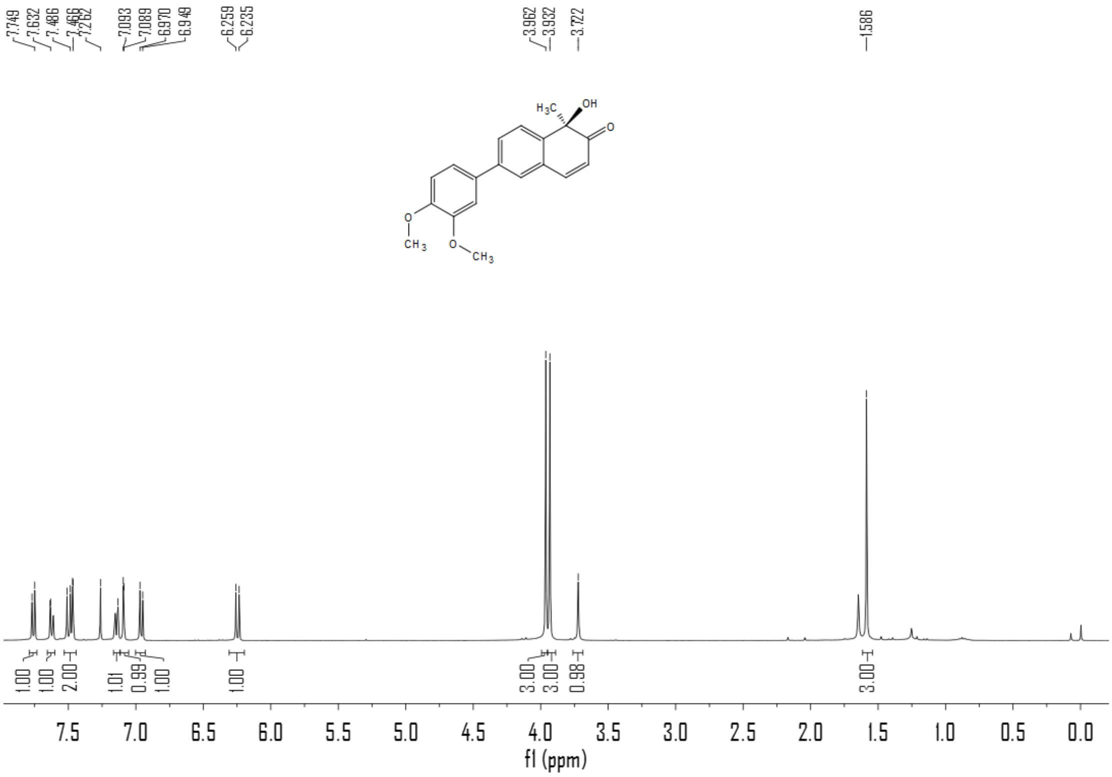

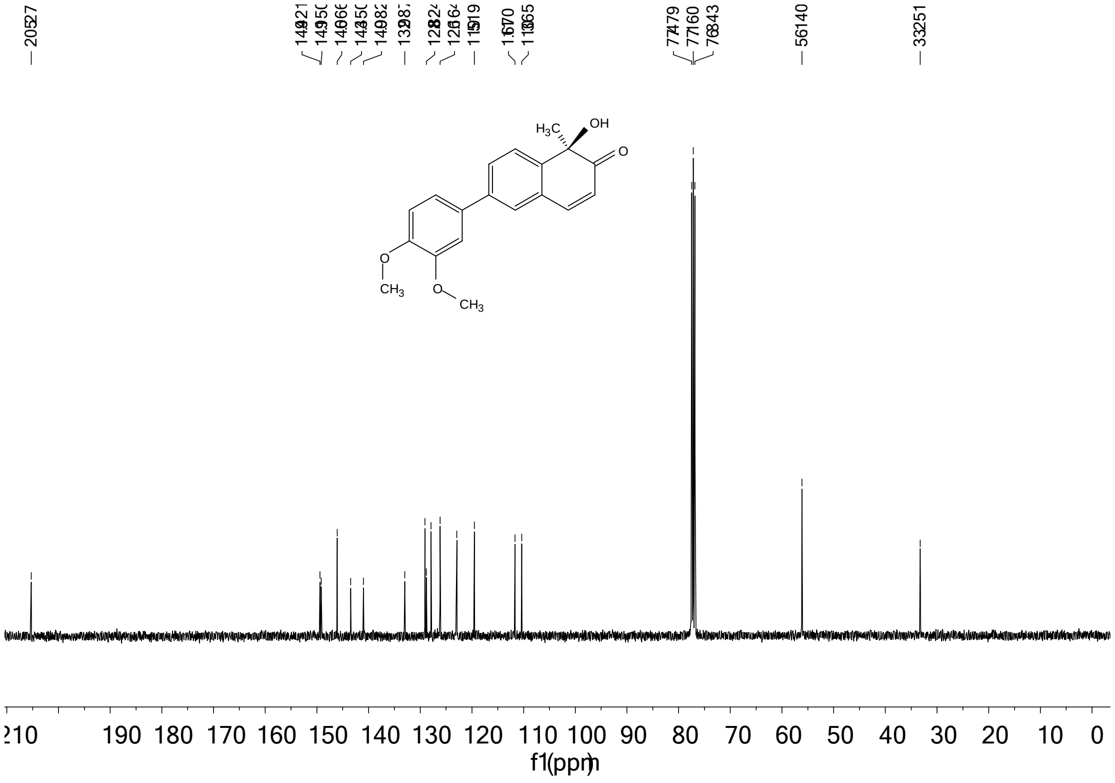

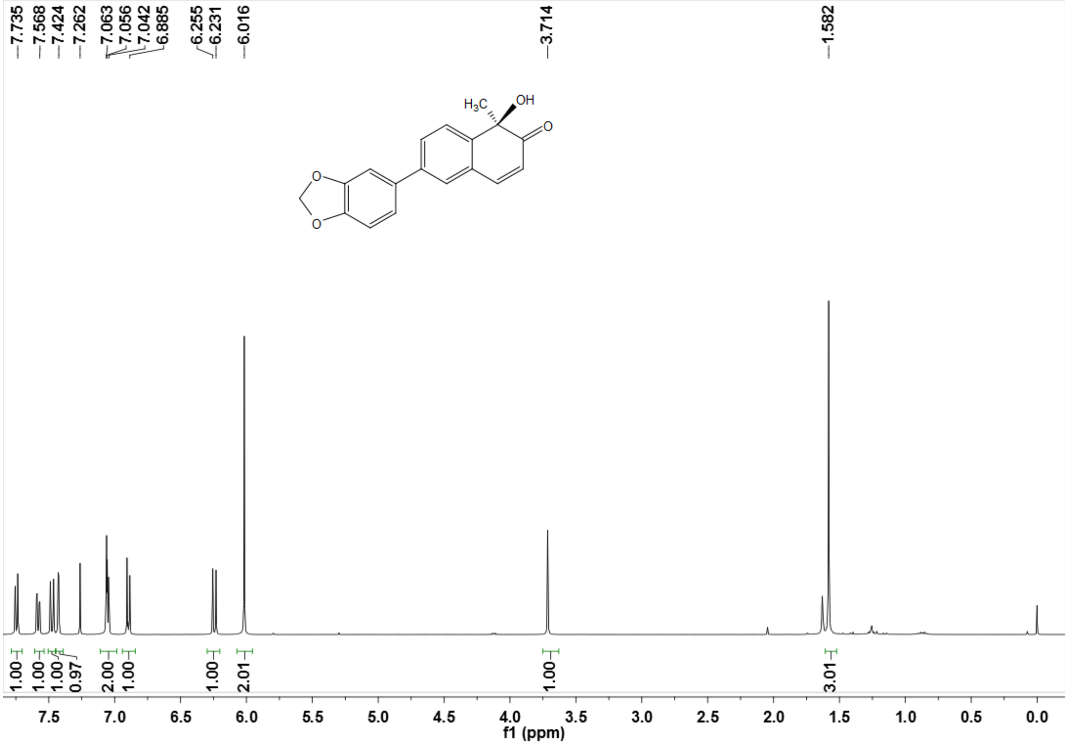
7.735
7.568
7.424
7.262
7.063
7.056
7.042
6.885
6.255
6.231
6.016
3.714
1.582
H_3C
OH
O
O
O
1.00
1.00
1.00
0.97
2.00
1.00
1.00
2.01
1.00
3.01
f1 (ppm)

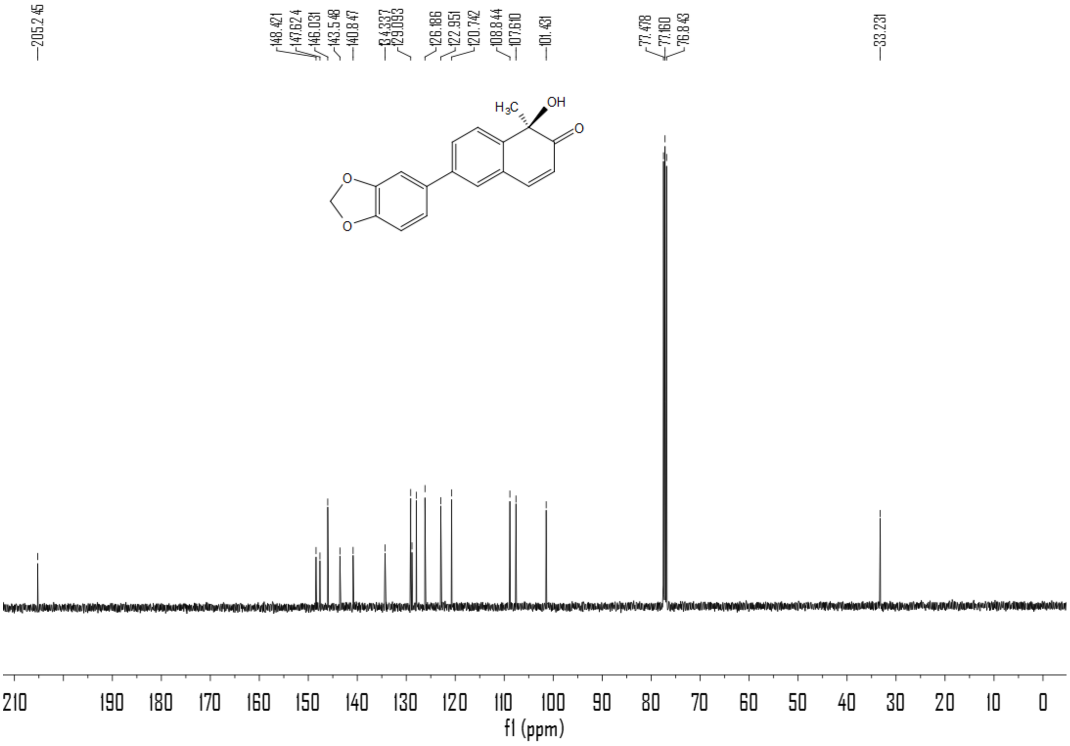
205.245
148.421
147.624
146.031
143.548
140.847
134.337
129.093
126.186
122.951
120.742
108.844
107.610
101.431
77.478
77.160
76.843
33.231
H_3C
OH
O
O
O
f1 (ppm)

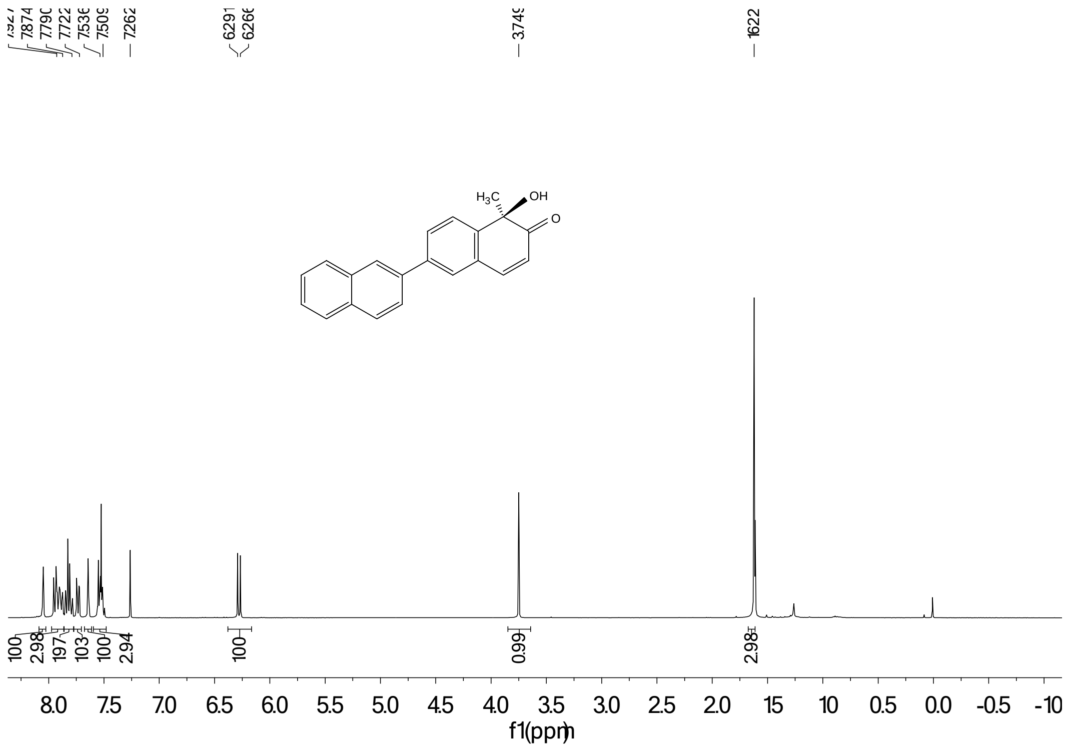
7874
7790
7722
7536
7509
7262
6291
6266
3749
1622
H3C
OH
O
100
2.98
197
103
100
2.94
100
0.99
2.98
8.0 7.5 7.0 6.5 6.0 5.5 5.0 4.5 4.0 3.5 3.0 2.5 2.0 15 10 0.5 0.0 -0.5 -10
f1(ppm)

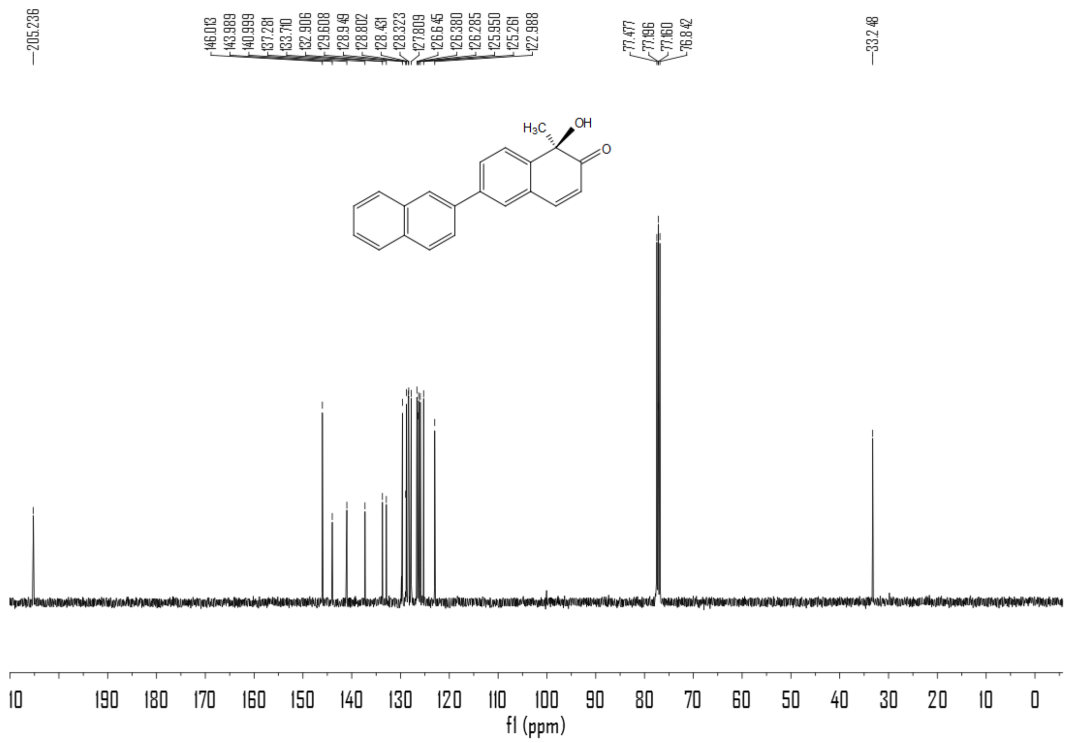
205.236
146.013
143.989
140.999
137.281
133.710
132.906
129.608
128.949
128.802
128.431
128.323
127.809
126.645
126.380
126.285
125.950
125.261
122.988
77.477
77.196
77.160
76.842
33.248
H3C
OH
O
190 180 170 160 150 140 130 120 110 100 90 80 70 60 50 40 30 20 10 0
f1 (ppm)

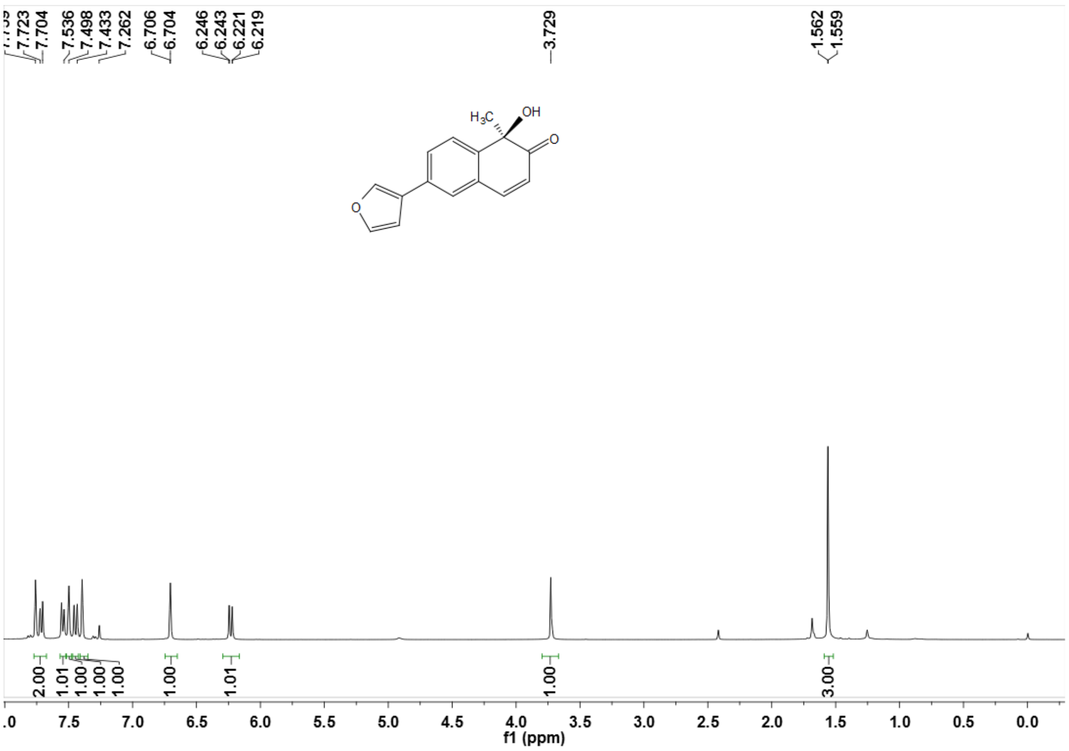

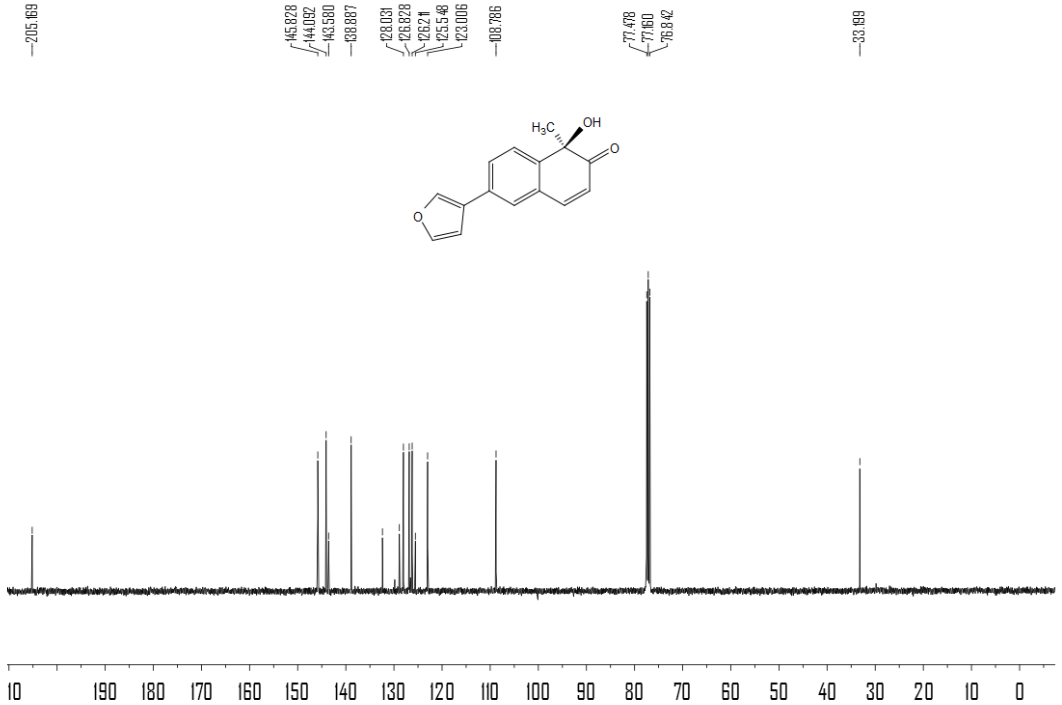

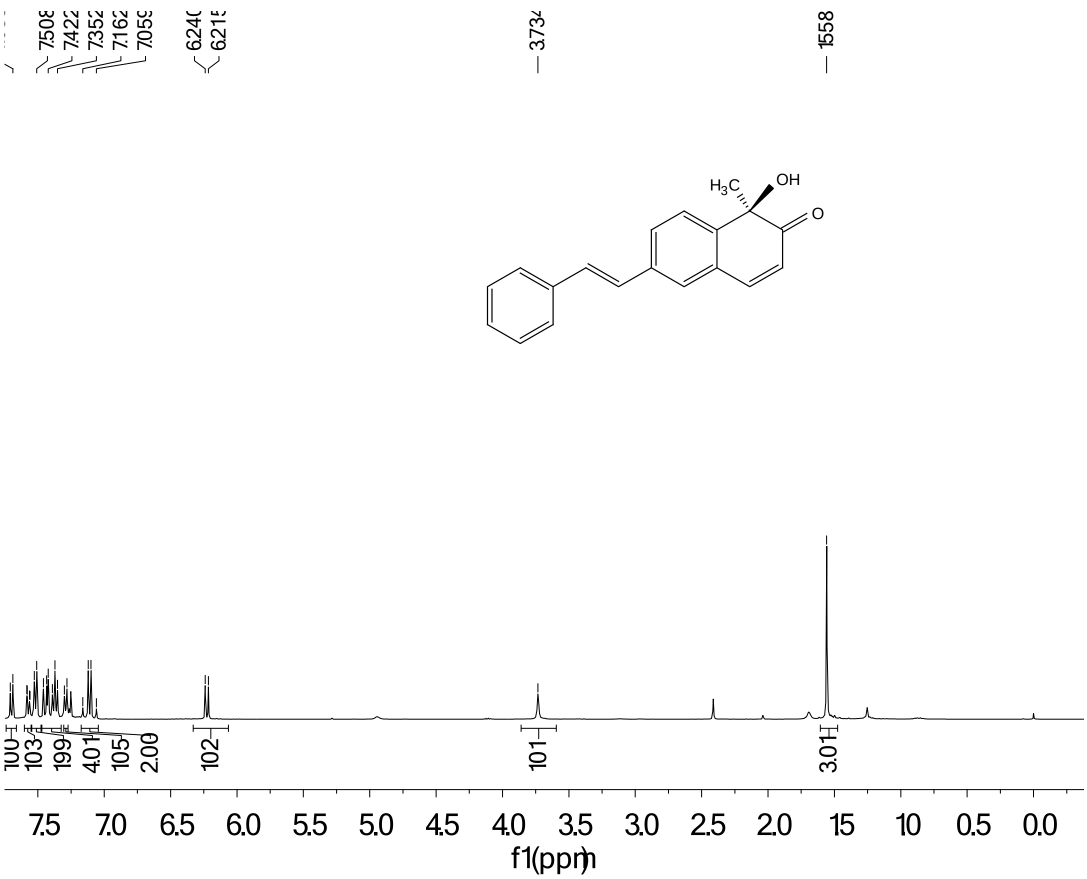

3.734
1.558
H3C
OH
O
3.01
f1 (ppm)

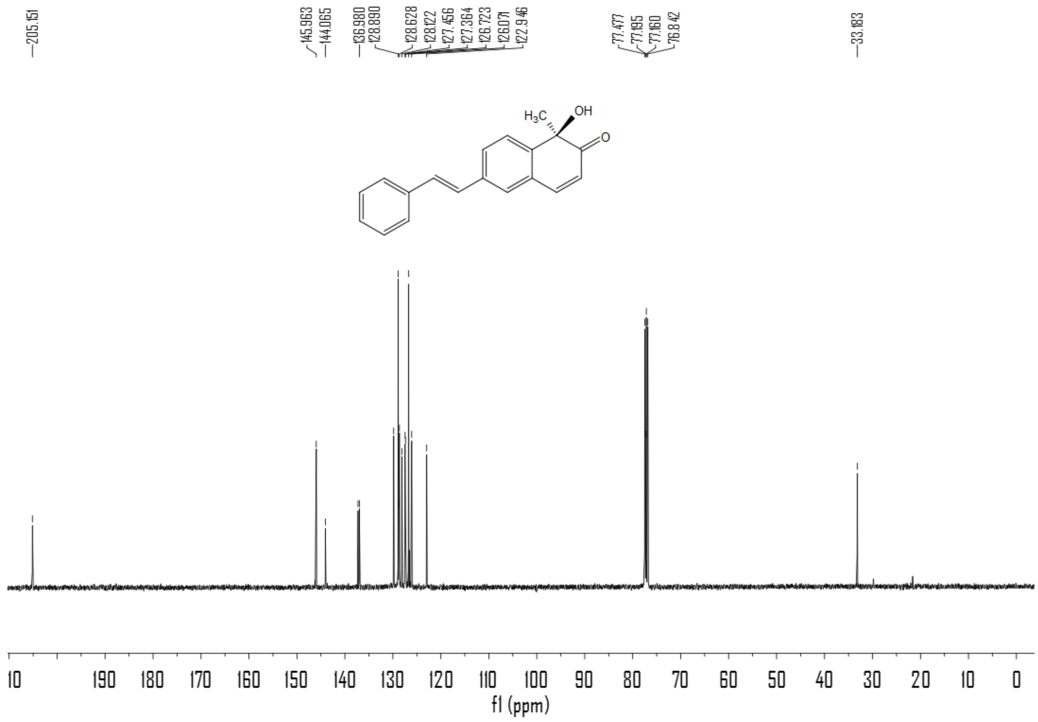

205.151
145.963
144.065
136.980
128.890
128.628
128.122
127.456
127.364
126.723
126.071
122.946
77.477
77.195
77.160
76.842
33.183
H3C
OH
O
f1 (ppm)

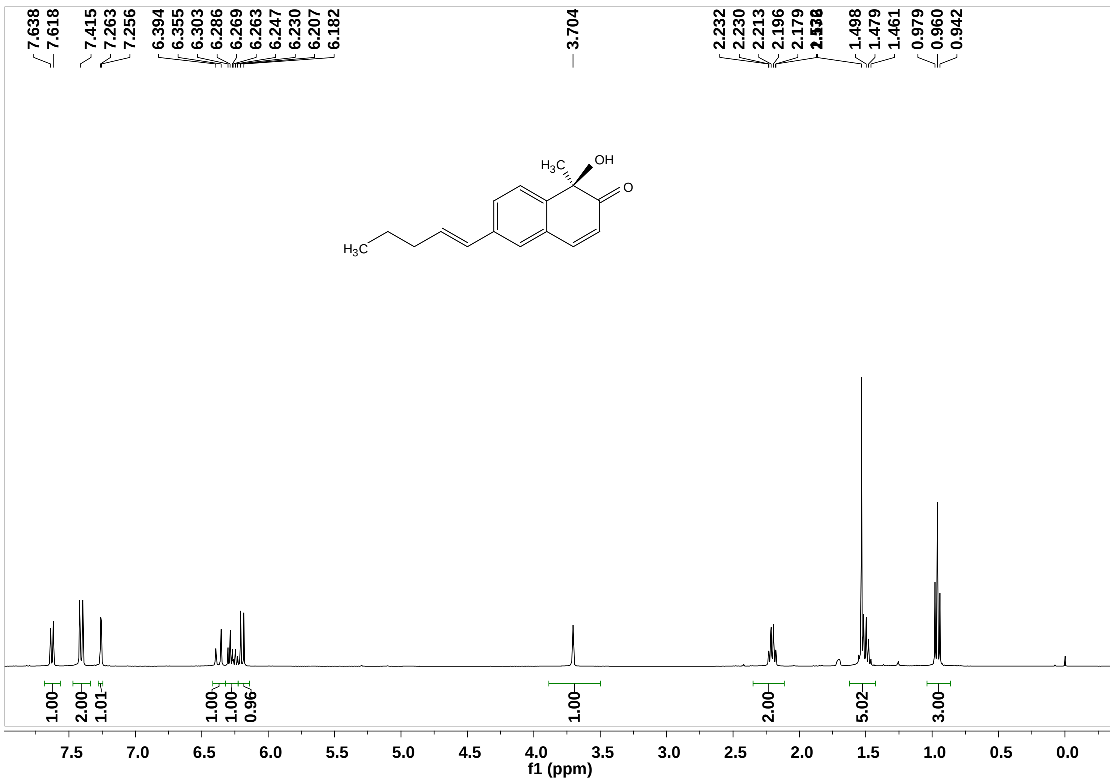

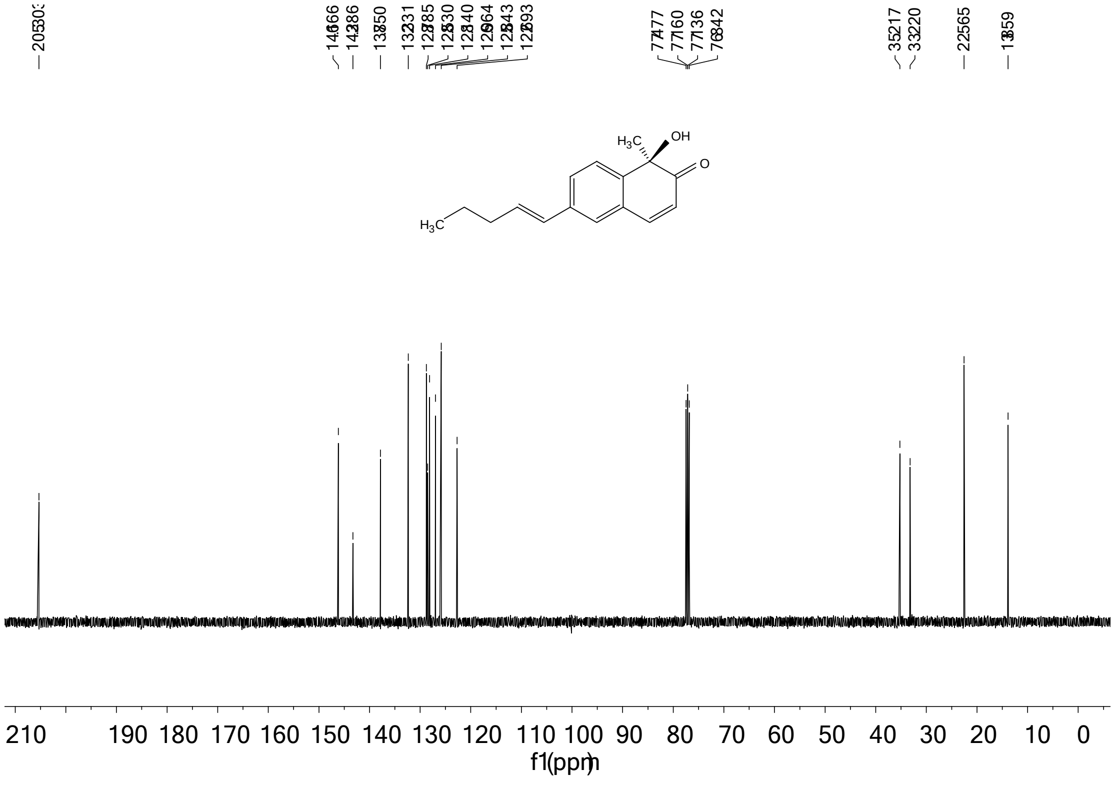

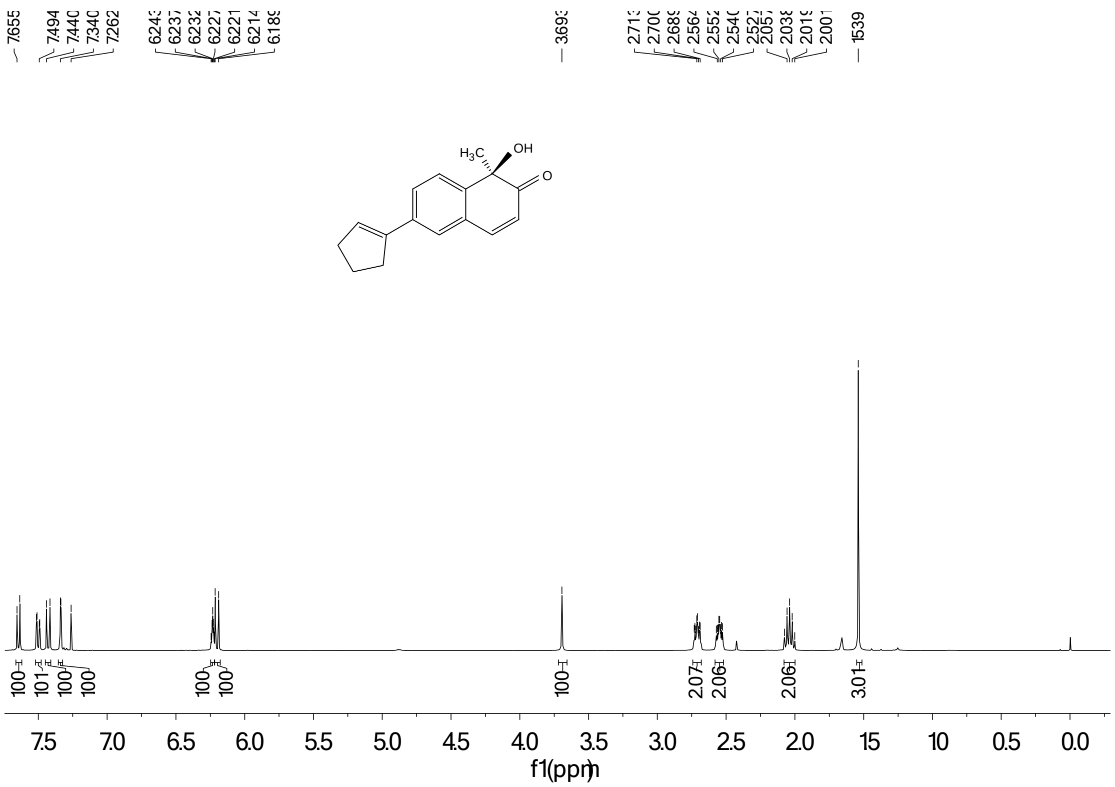

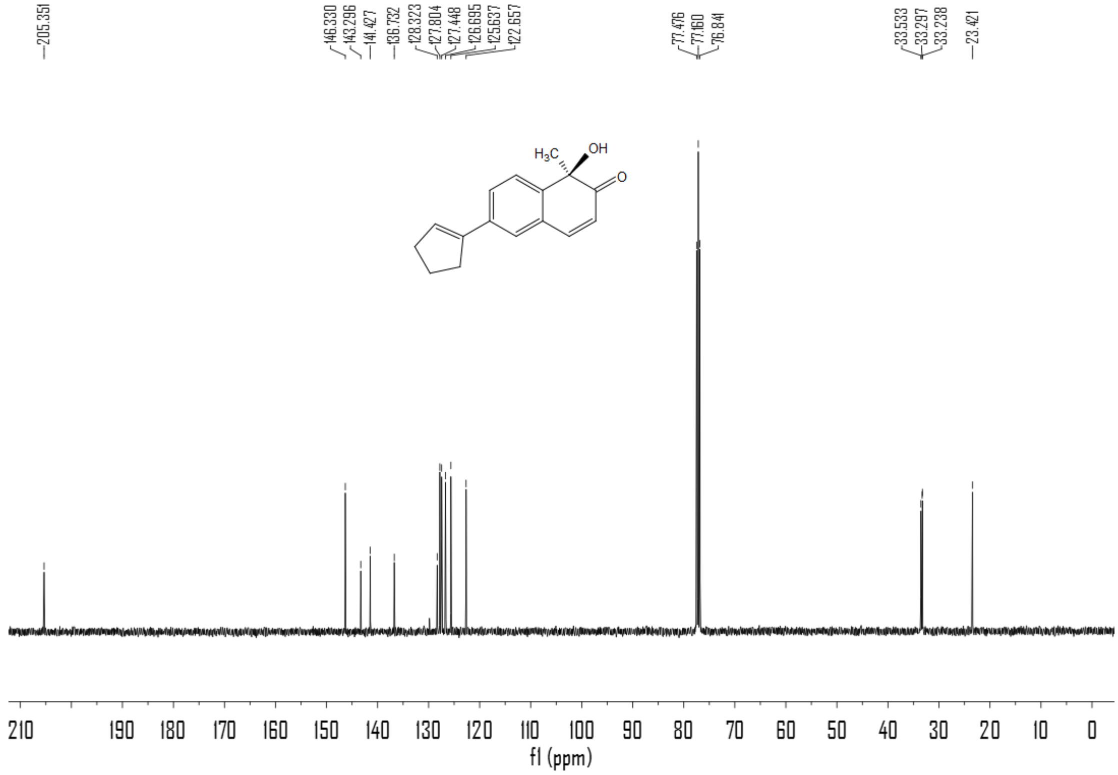

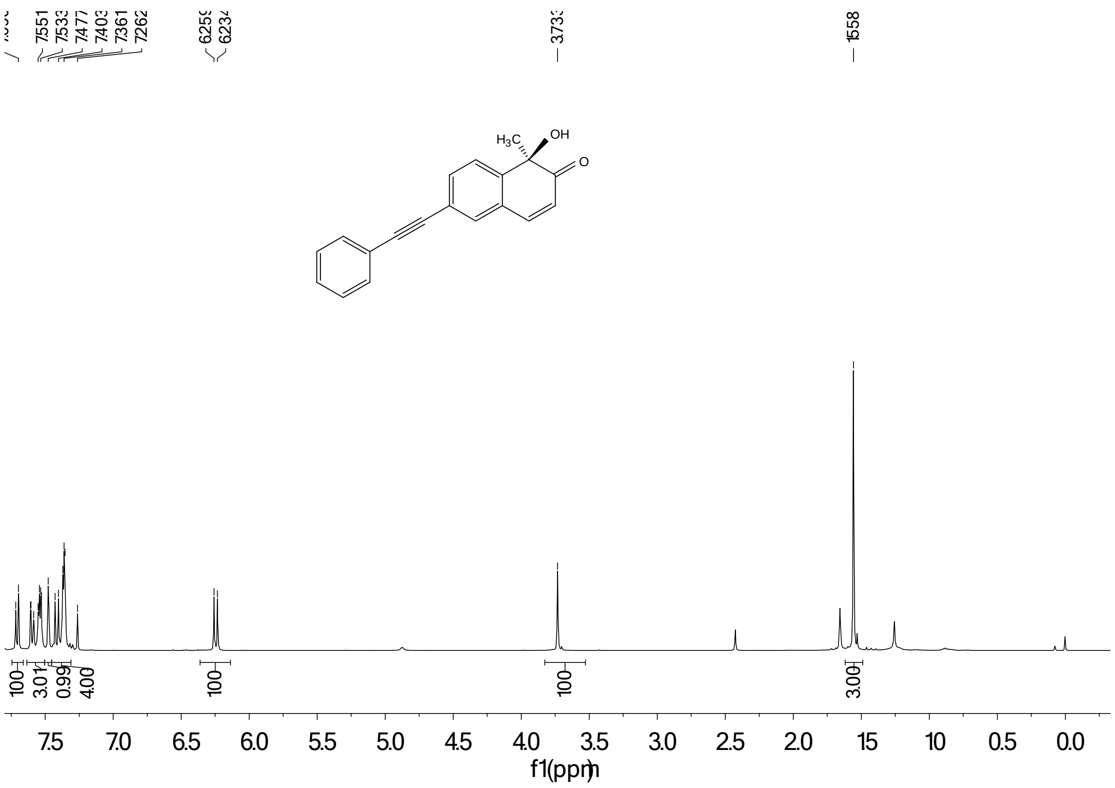

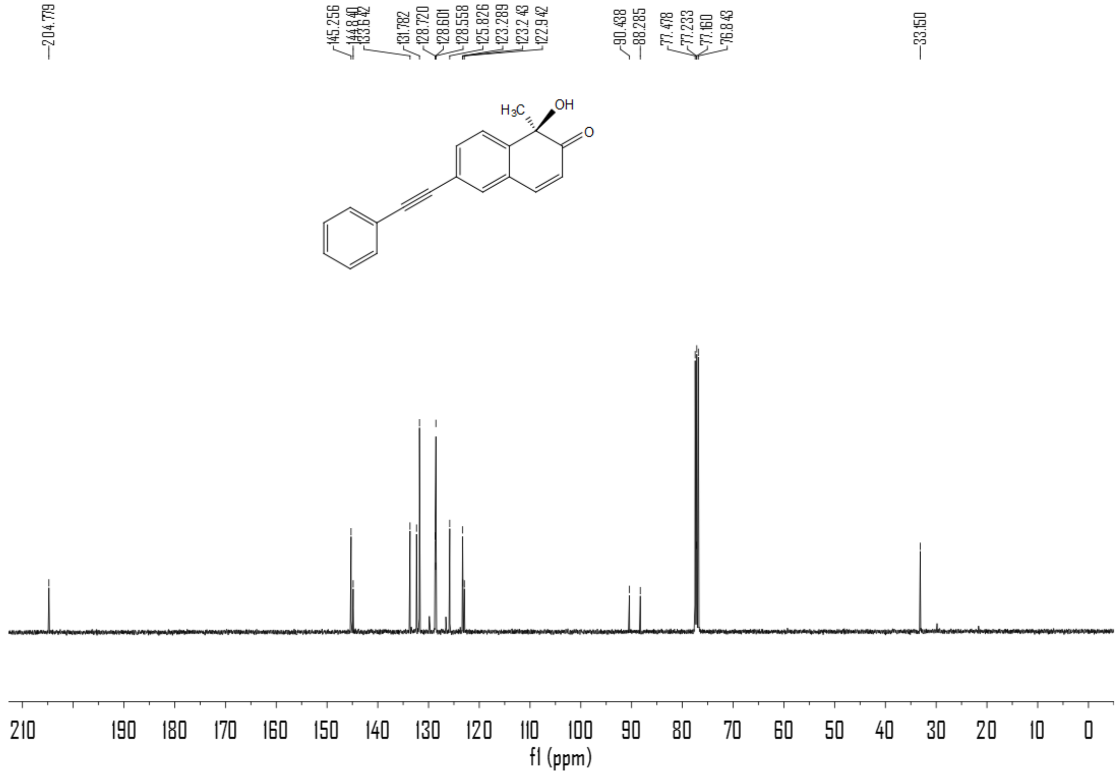

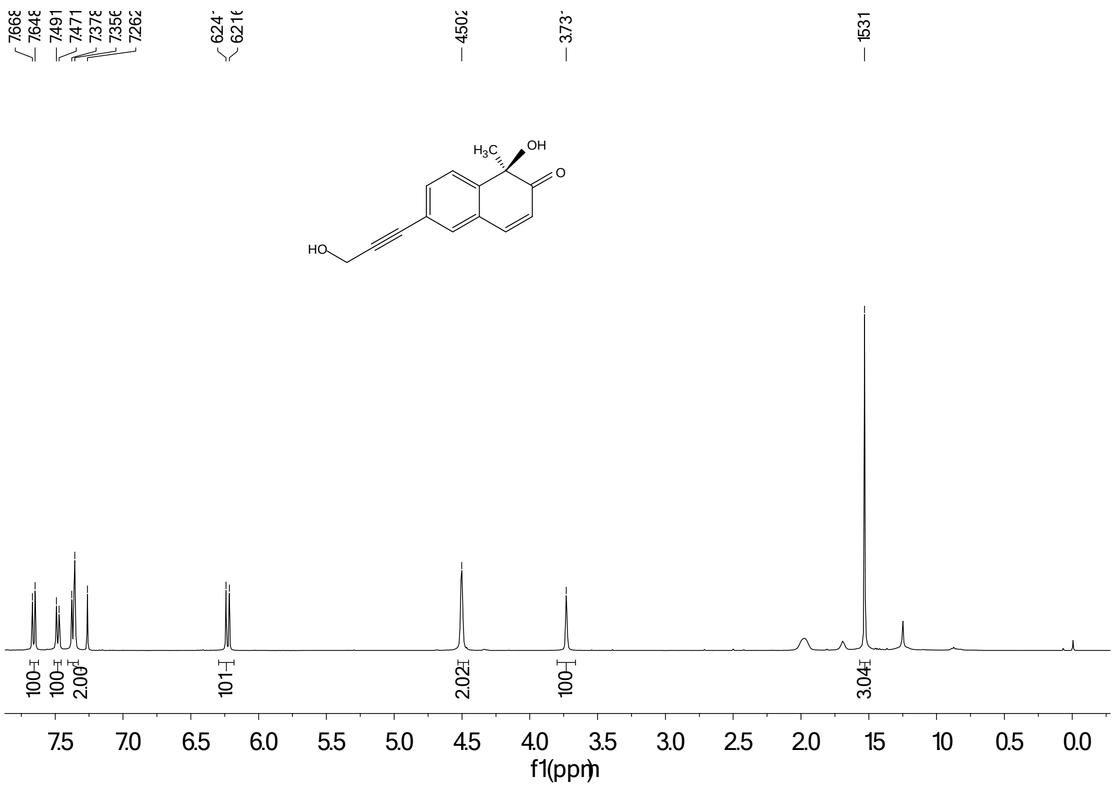
H3C
OH
O
HO
f1 (ppm)

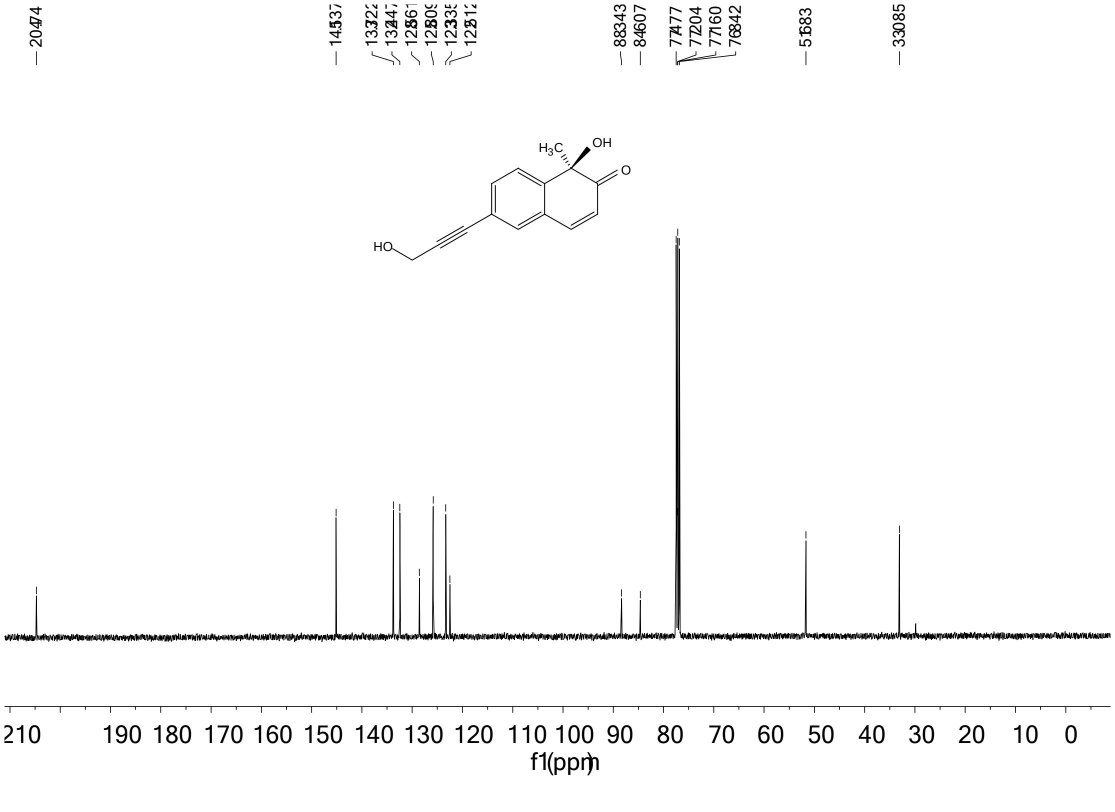
H3C
OH
O
HO
f1 (ppm)

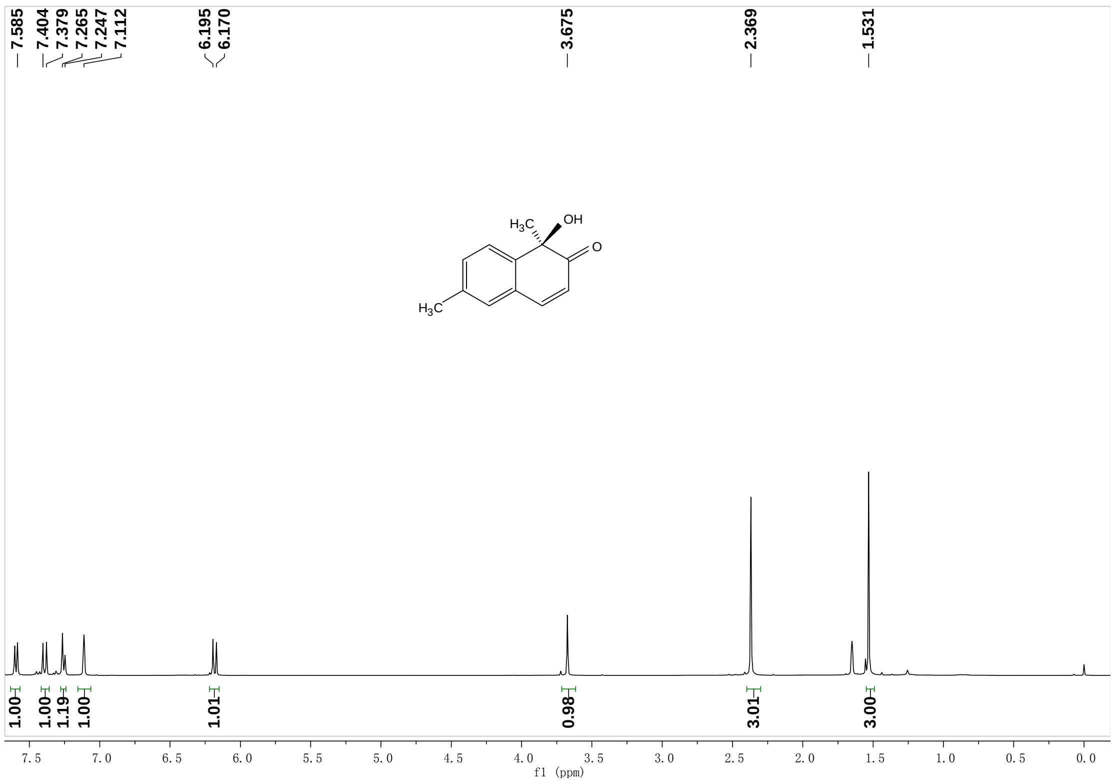

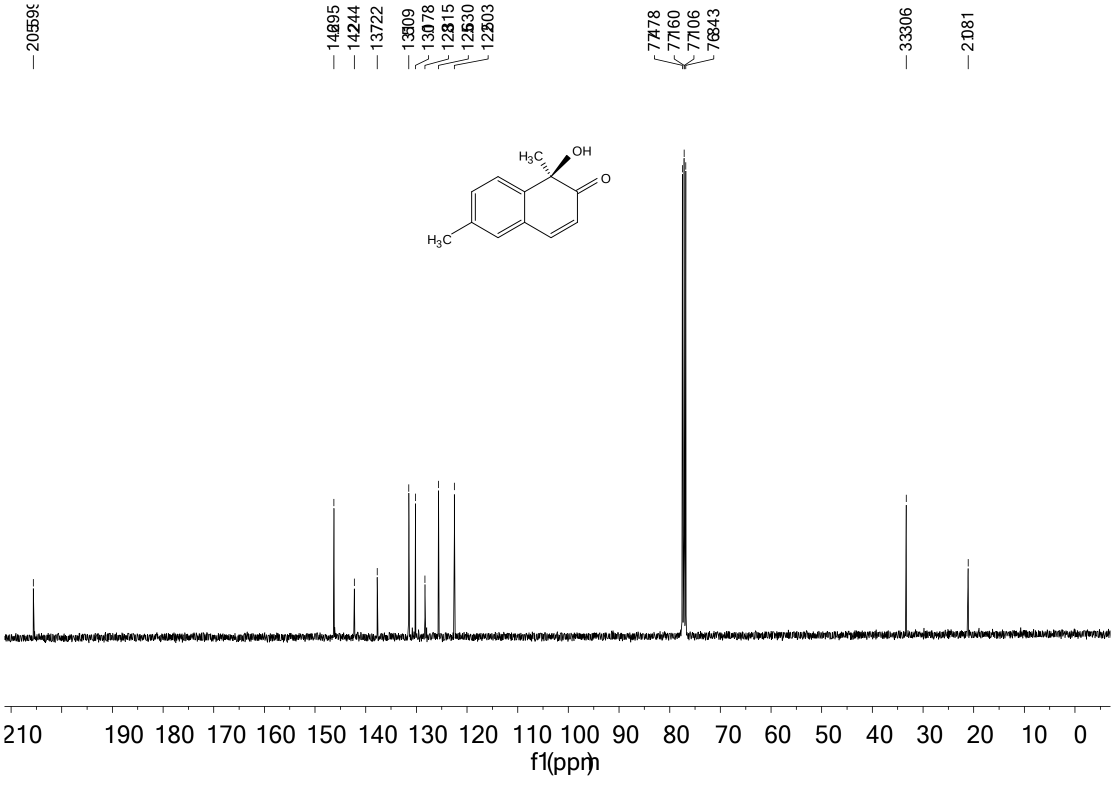

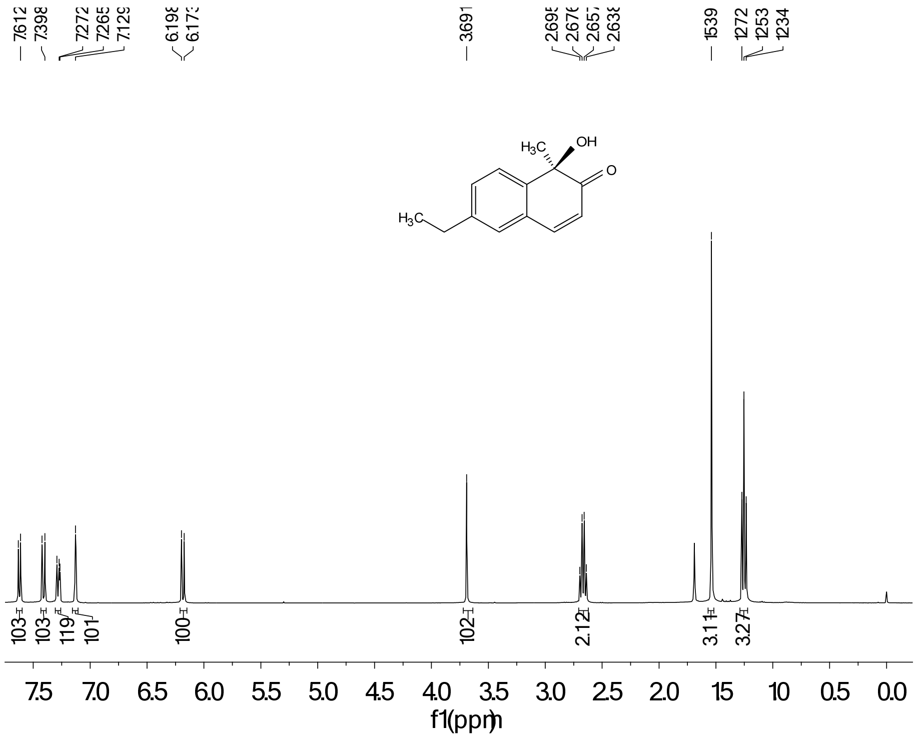
7.612
7.398
7.272
7.265
7.129
6.198
6.173
3.691
2.695
2.676
2.657
2.638
1.539
1.272
1.253
1.234
H3C
OH
O
H3C
1.03
1.03
1.19
1.01
1.00
1.02
2.12
3.11
3.27
f1 (ppm)

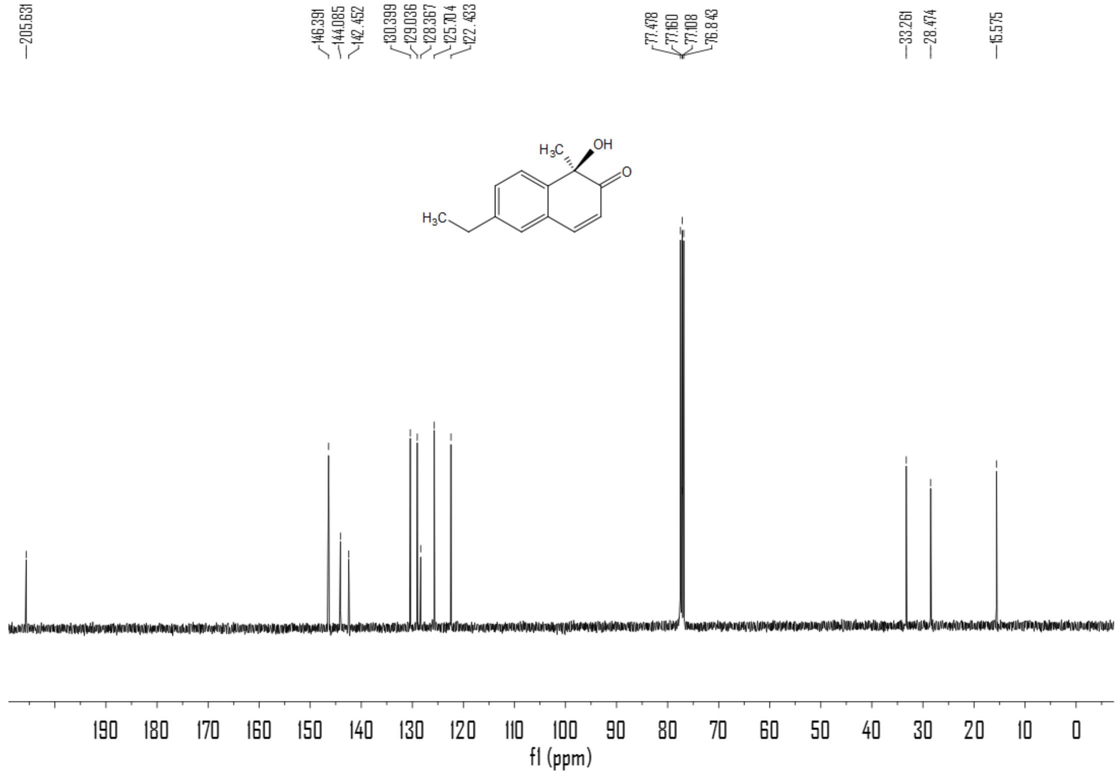
205.631
146.391
144.085
142.452
130.399
129.036
128.367
125.704
122.433
77.478
77.160
77.108
76.843
33.261
28.474
15.575
H3C
OH
O
H3C
f1 (ppm)

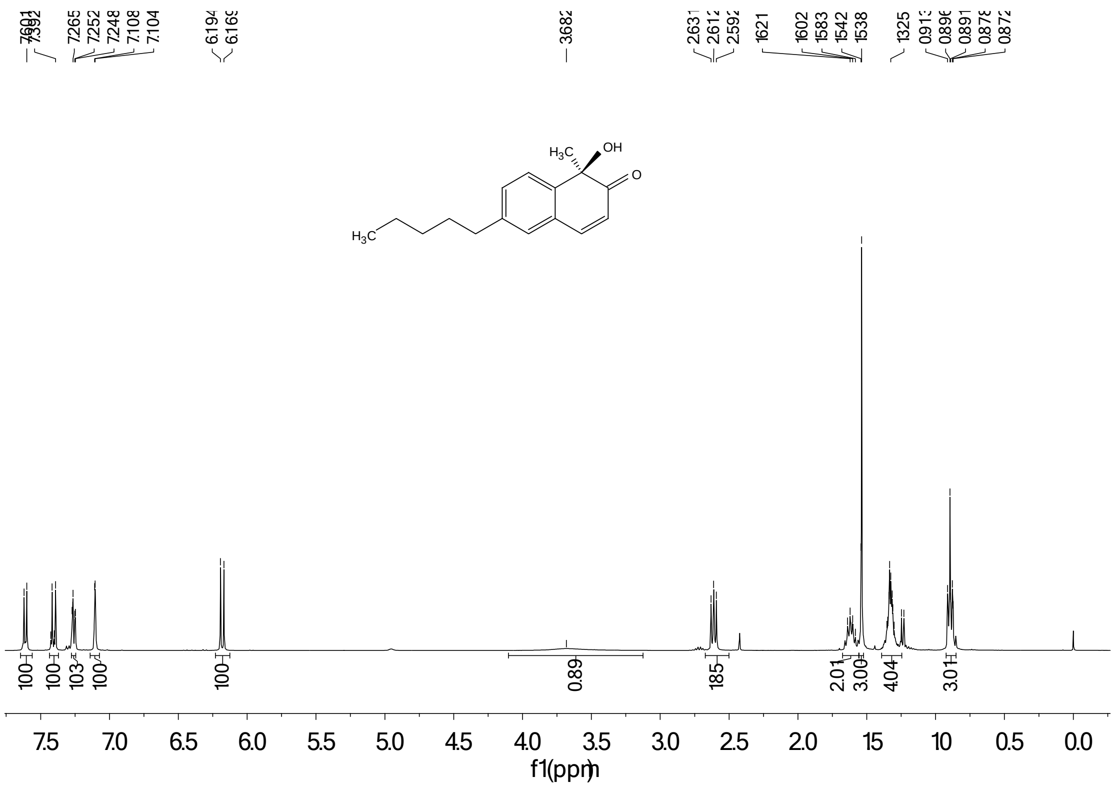

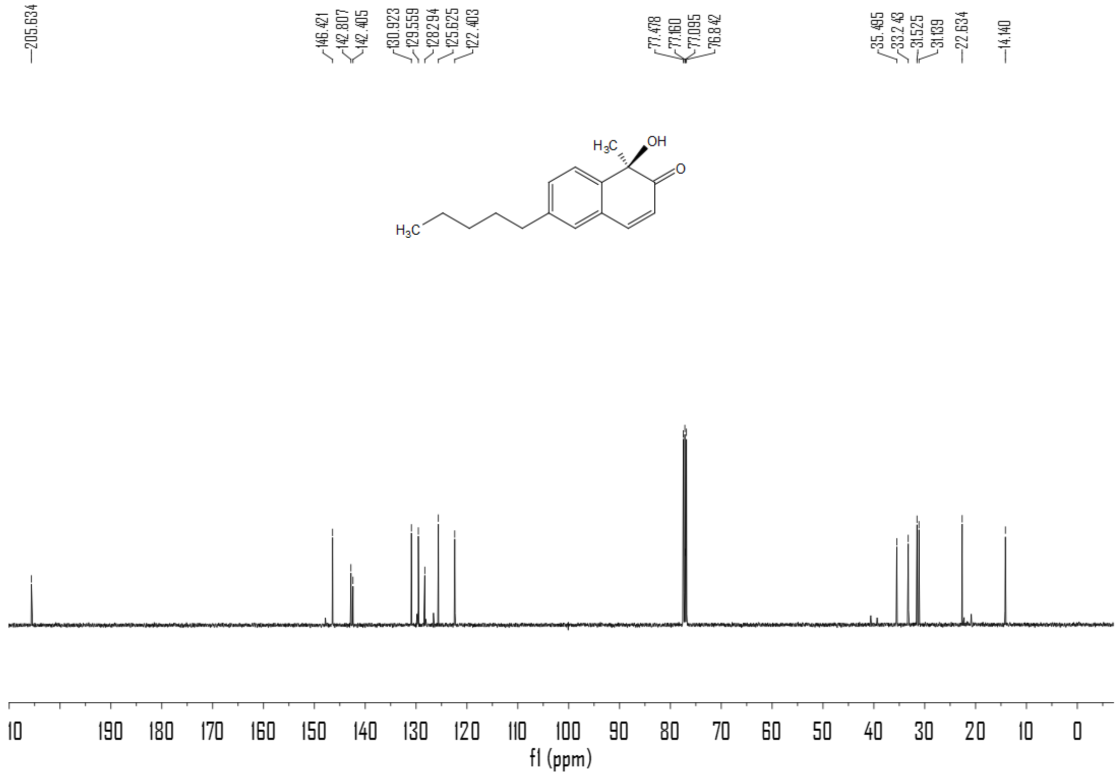

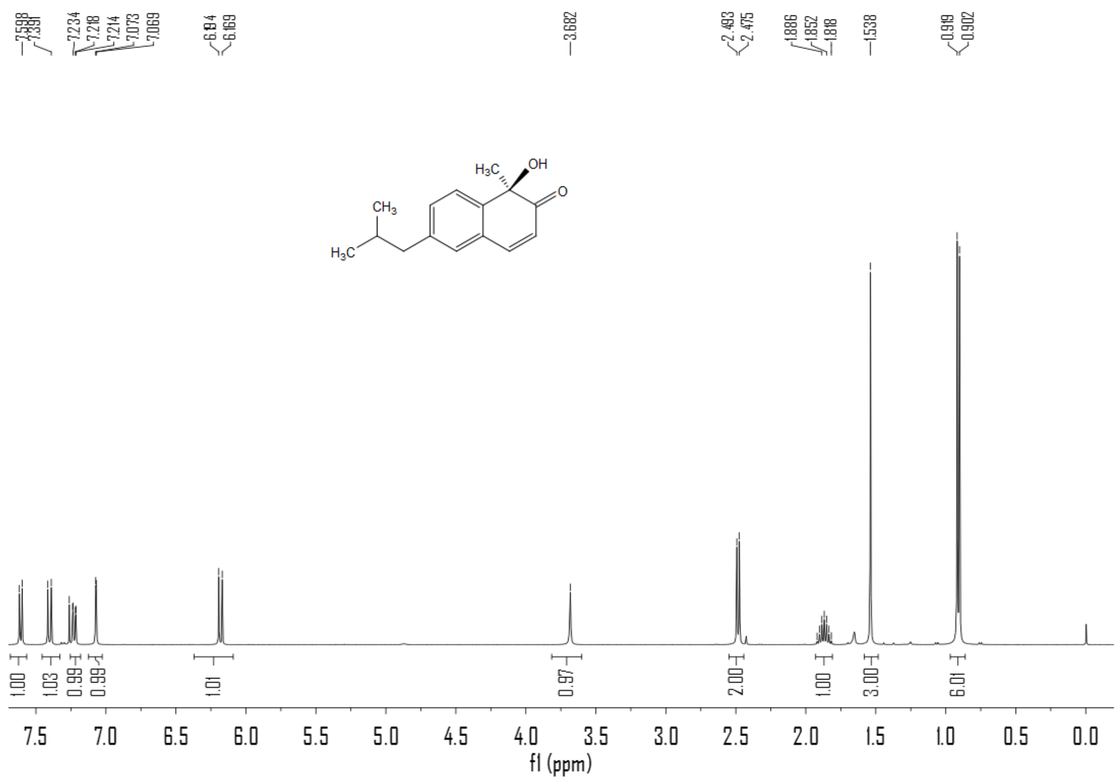

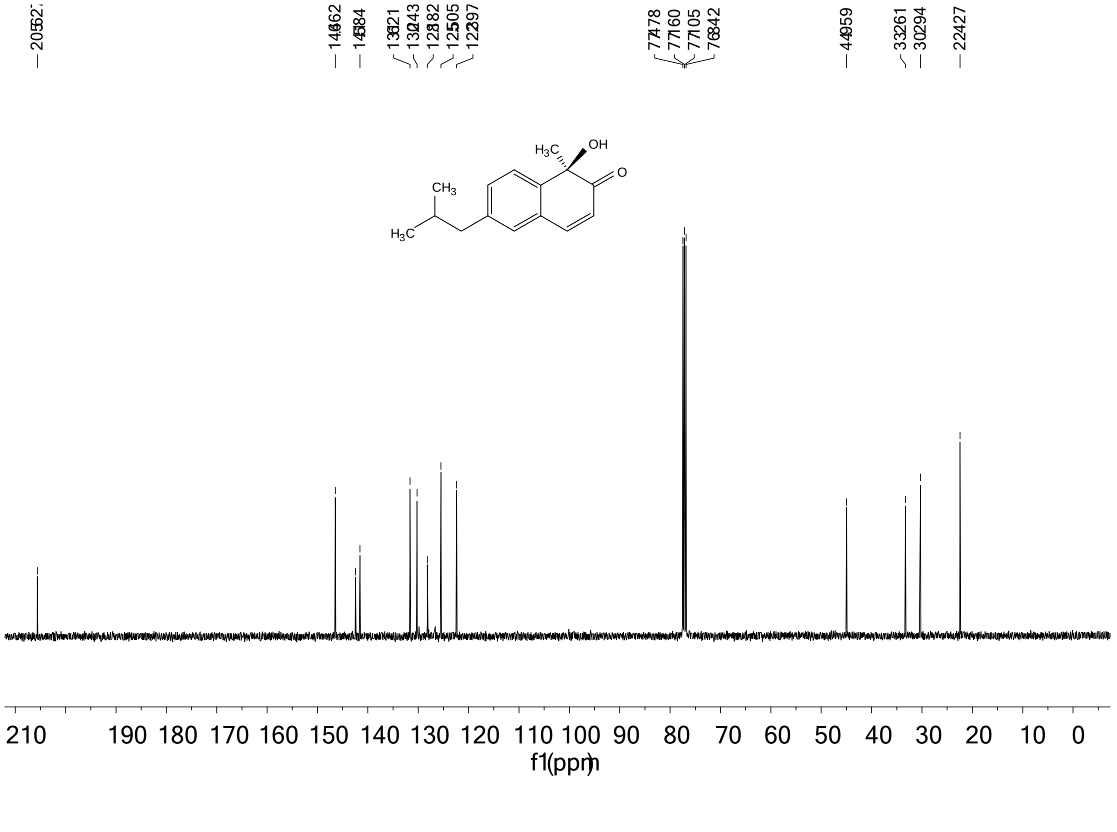

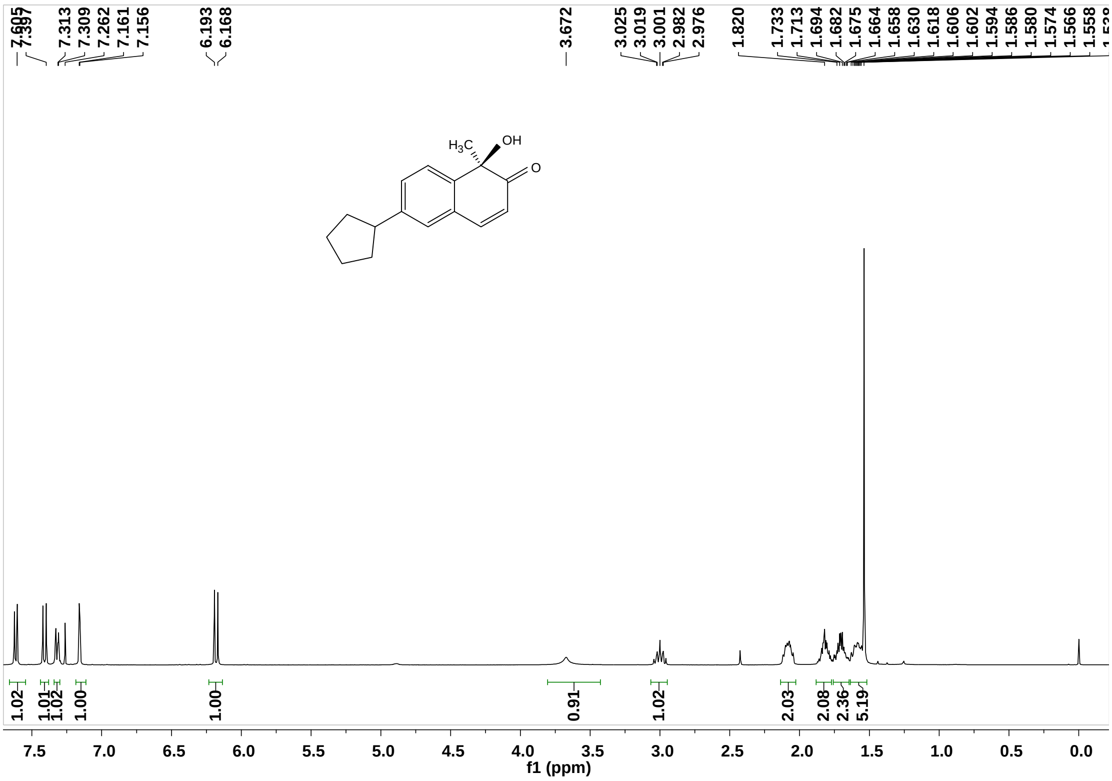
7.605
7.597
7.313
7.309
7.262
7.161
7.156
6.193
6.168
3.672
3.025
3.019
3.001
2.982
2.976
1.820
1.733
1.713
1.694
1.682
1.675
1.664
1.658
1.630
1.618
1.606
1.602
1.594
1.586
1.580
1.574
1.566
1.558
H_3C
OH
O
1.02
1.01
1.02
1.00
1.00
0.91
1.02
2.03
2.08
2.36
5.19
7.5 7.0 6.5 6.0 5.5 5.0 4.5 4.0 3.5 3.0 2.5 2.0 1.5 1.0 0.5 0.0
f1 (ppm)

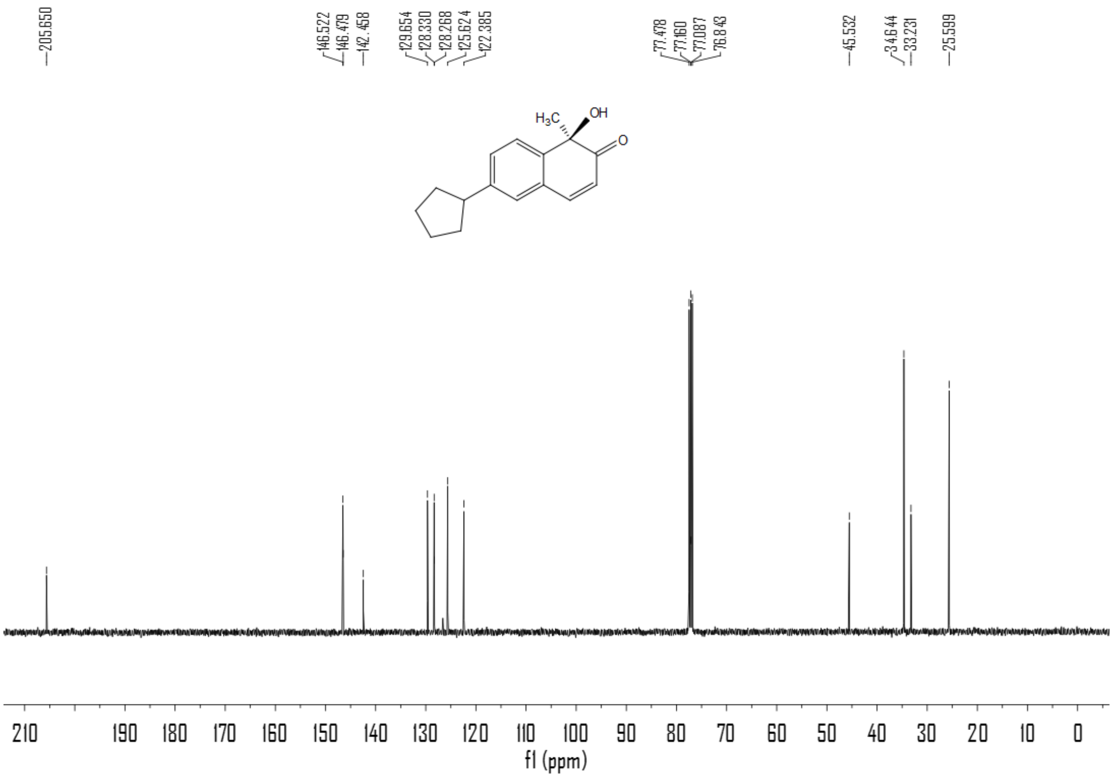
205.650
146.522
146.479
142.458
129.654
128.330
128.268
125.624
122.385
77.478
77.160
77.087
76.843
45.532
34.644
33.231
25.599
H_3C
OH
O
210 190 180 170 160 150 140 130 120 110 100 90 80 70 60 50 40 30 20 10 0
f1 (ppm)

14.

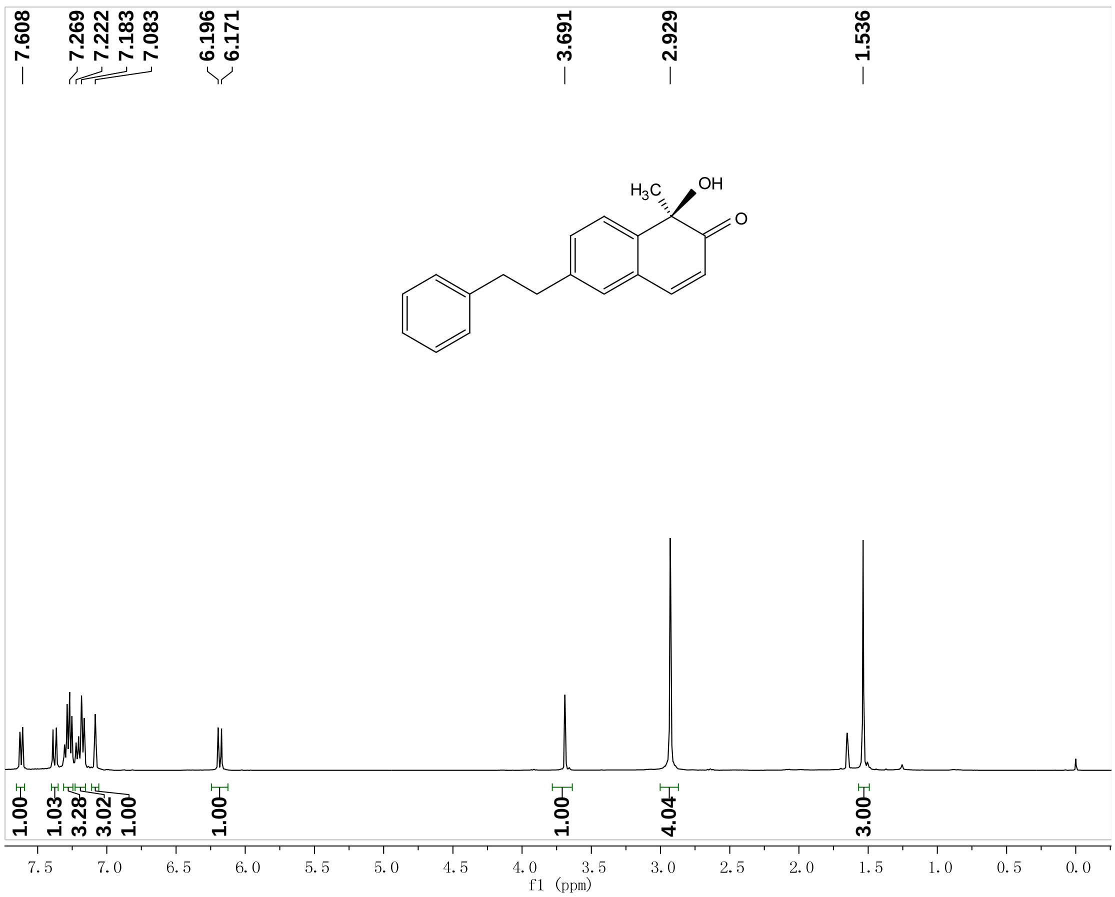
7.608
7.269
7.222
7.183
7.083
6.196
6.171
3.691
2.929
1.536
H_3C
OH
O
1.00
1.03
3.28
3.02
1.00
1.00
1.00
4.04
3.00
f1 (ppm)

15.

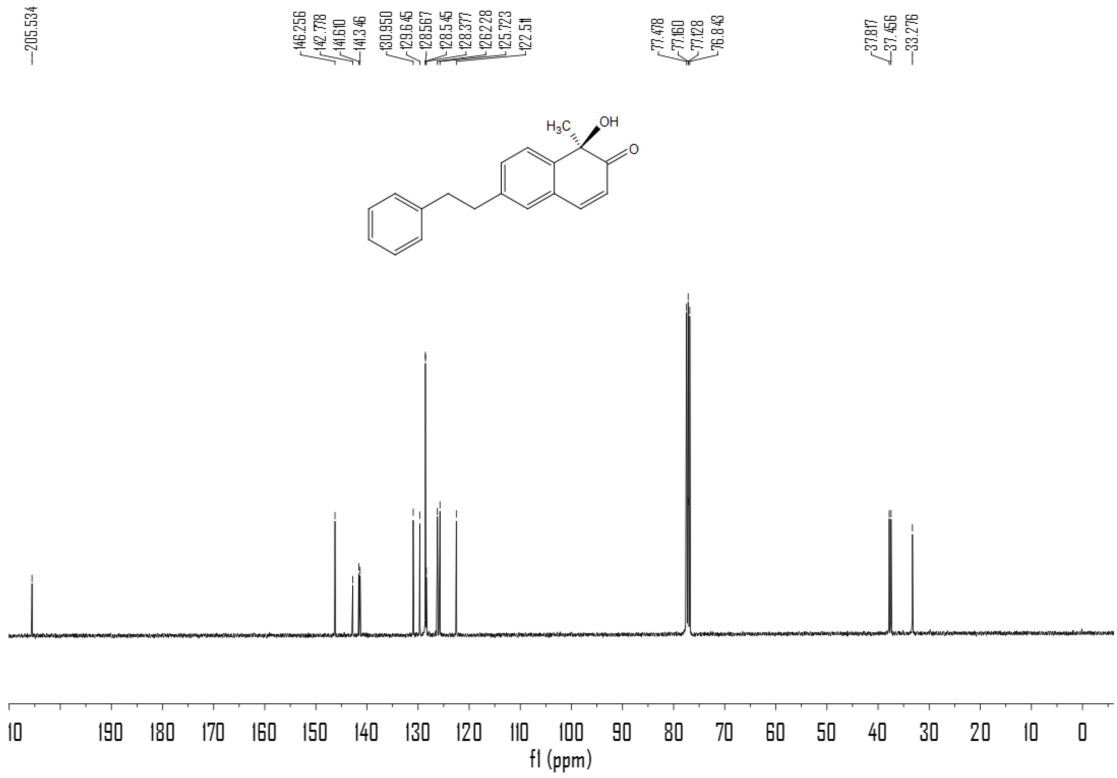
205.534
146.256
142.778
141.610
141.346
130.950
129.645
128.567
128.545
128.377
126.228
125.723
122.511
77.478
77.160
77.128
76.843
37.817
37.456
33.276
H_3C
OH
O
f1 (ppm)

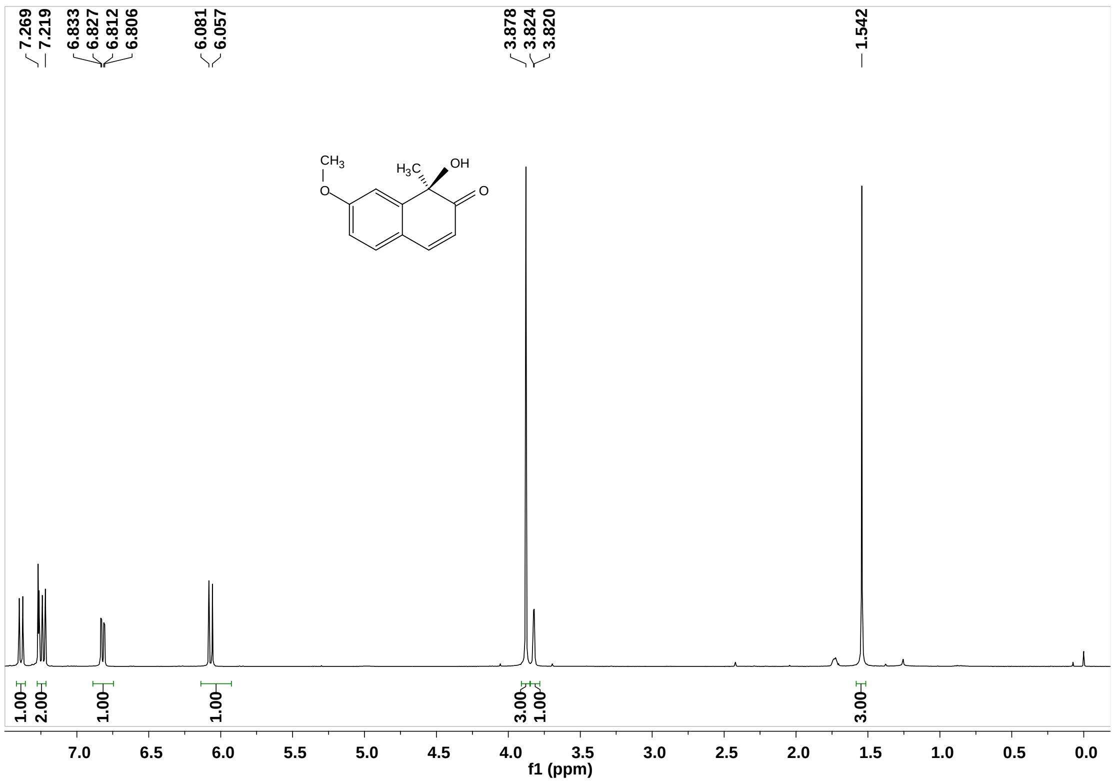

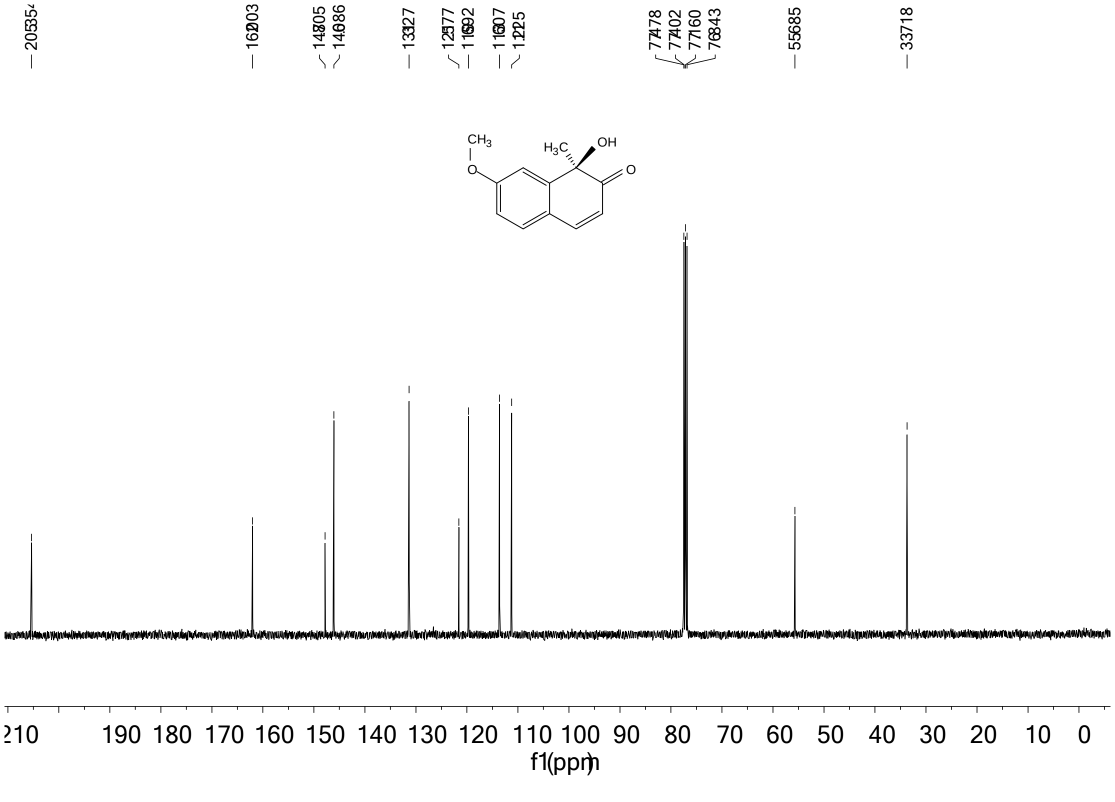

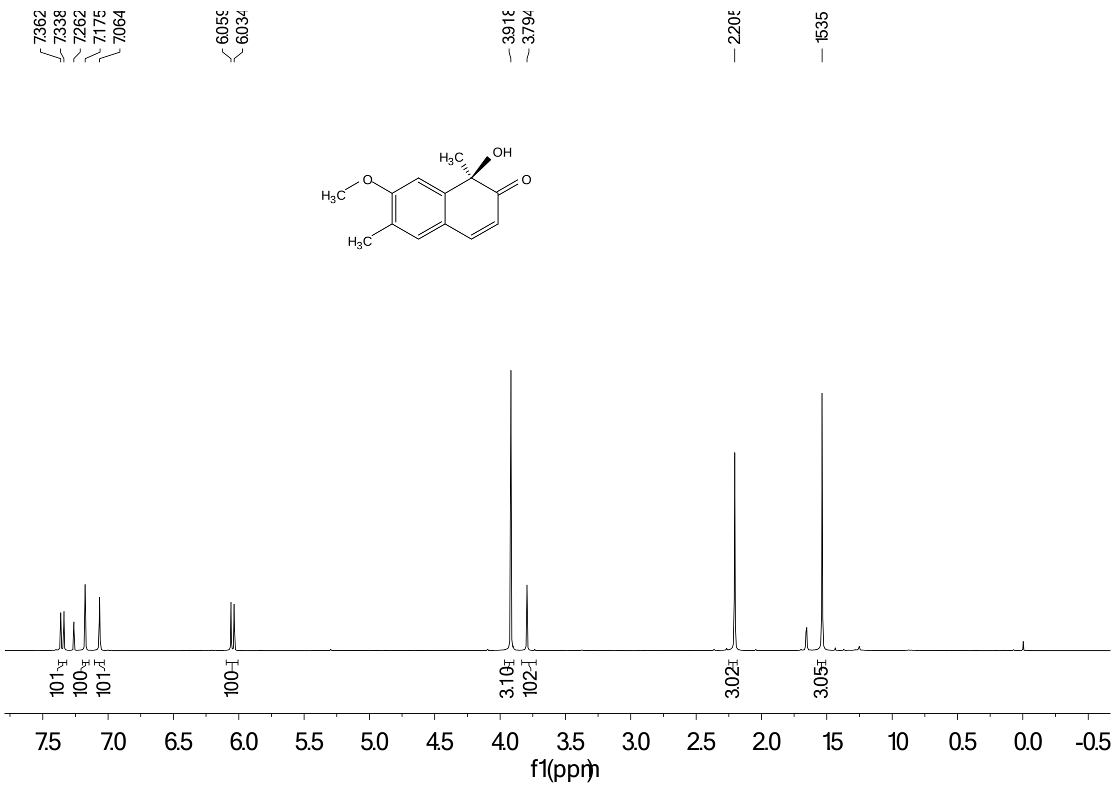

7.362
7.338
7.262
7.175
7.064
3.794
H3C
OH
O
H3C
O
H3C
7.5 7.0 6.5 6.0 5.5 5.0 4.5 4.0 3.5 3.0 2.5 2.0 1.5 1.0 0.5 0.0 -0.5
f1 (ppm)

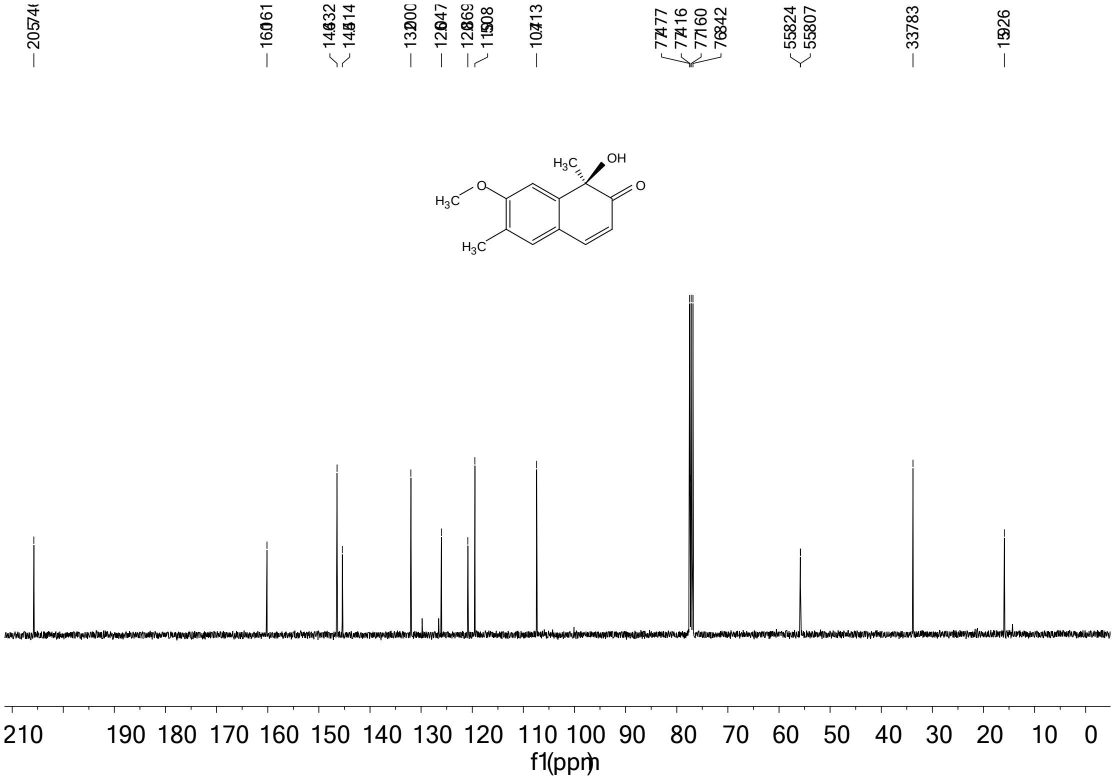

55.824
55.807
33.783
H3C
OH
O
H3C
O
H3C
210 190 180 170 160 150 140 130 120 110 100 90 80 70 60 50 40 30 20 10 0
f1 (ppm)

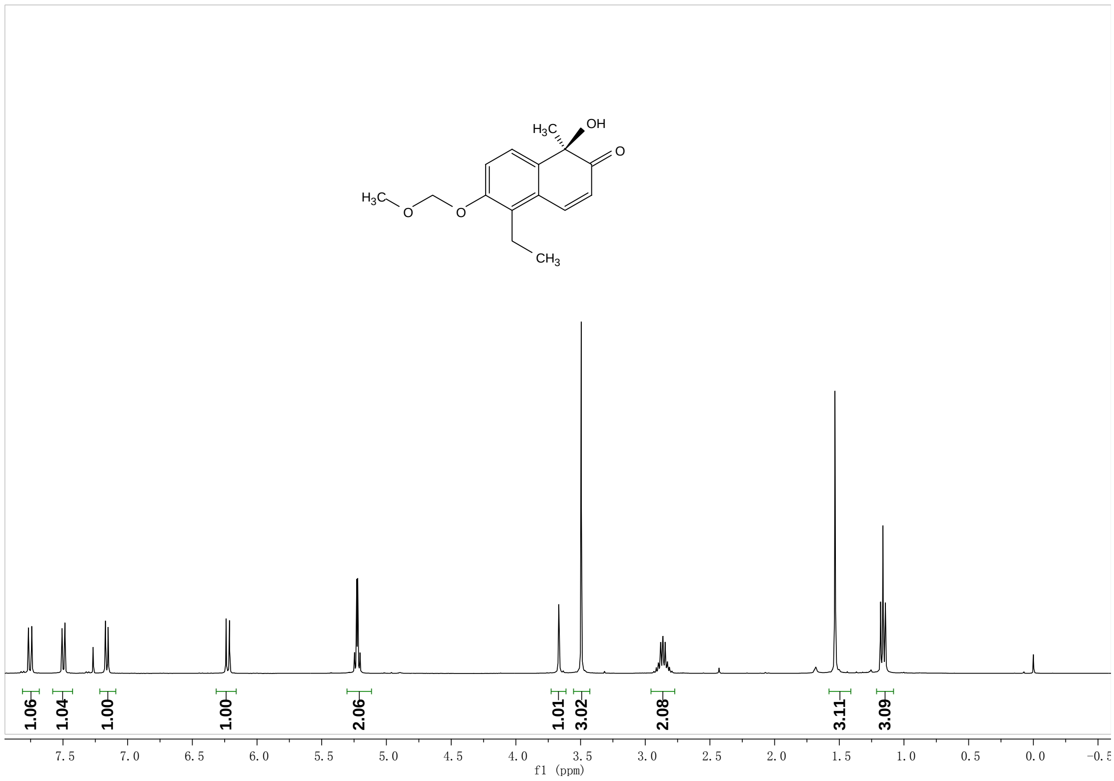

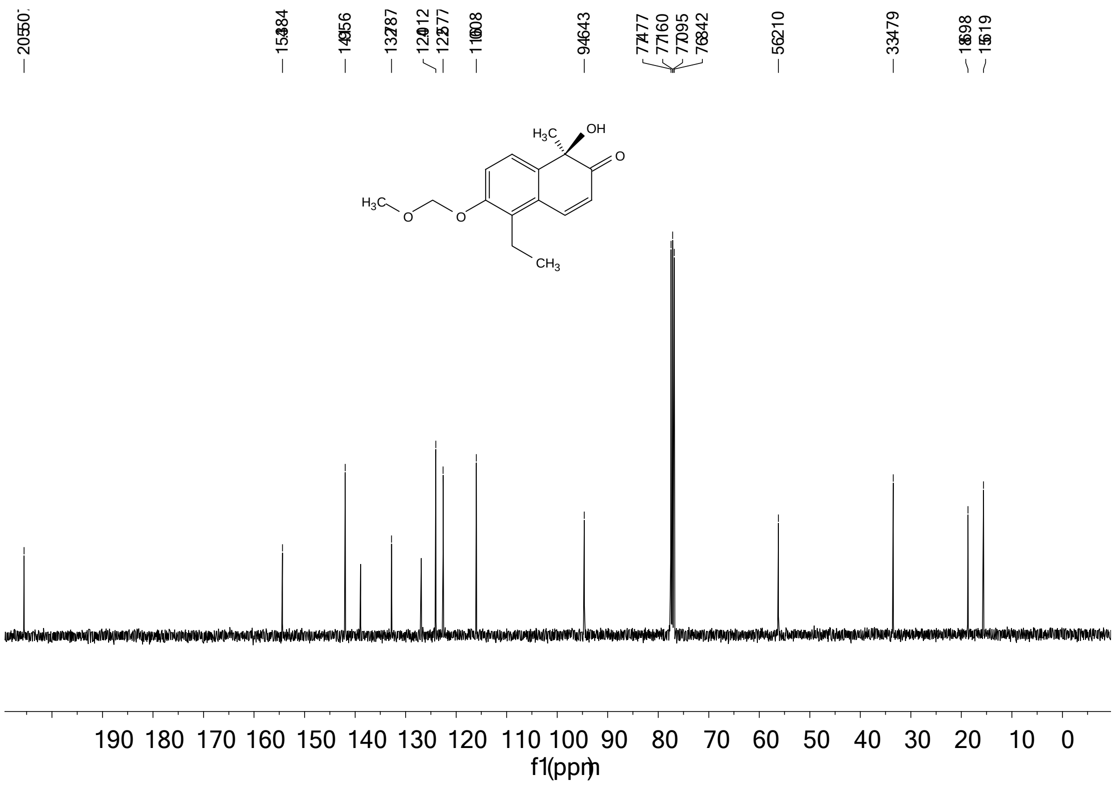

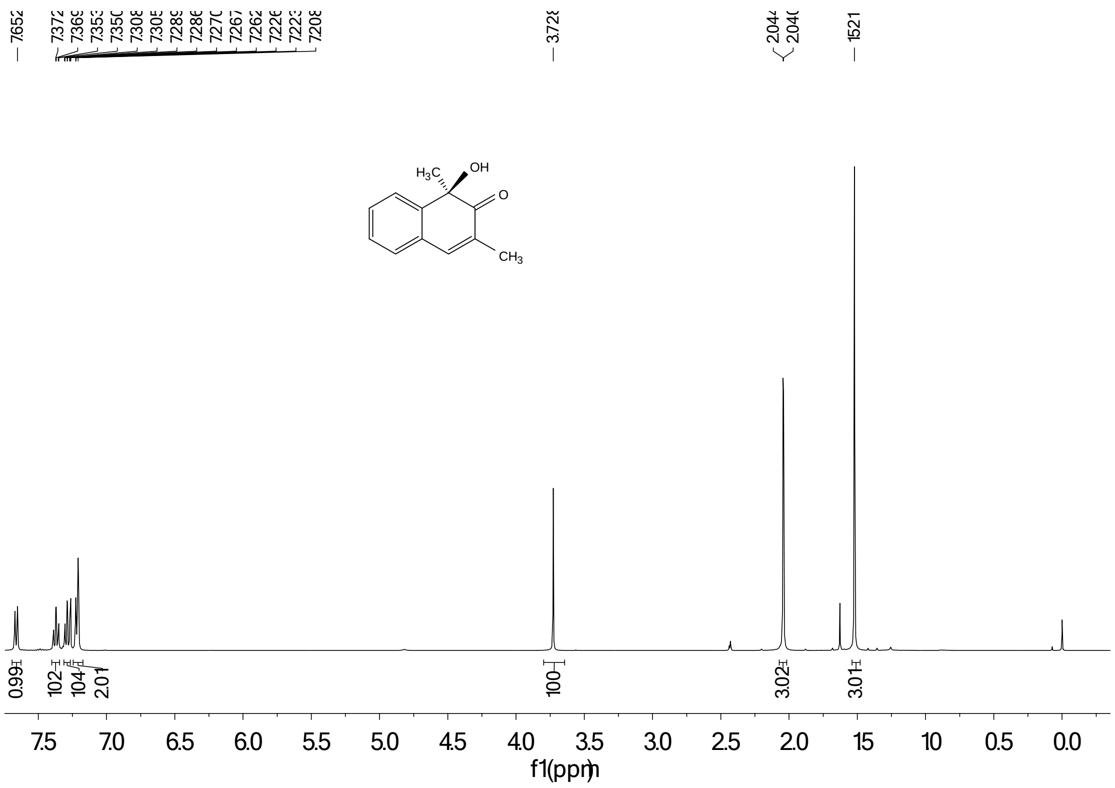

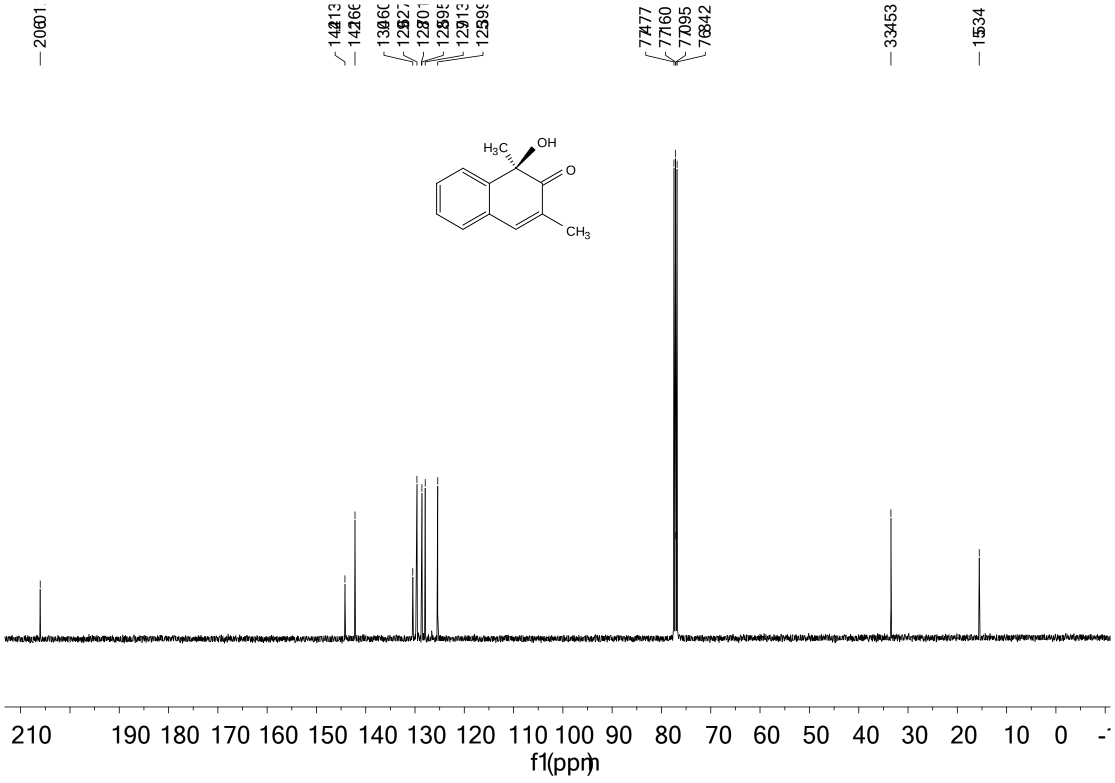

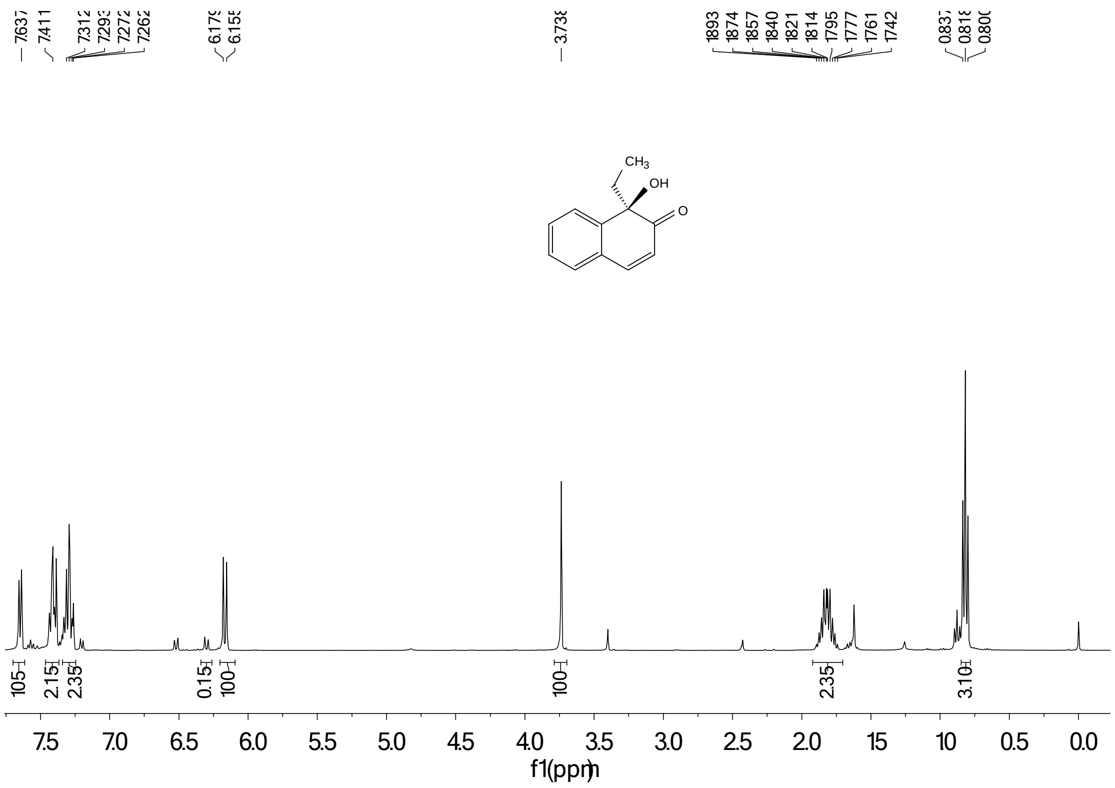

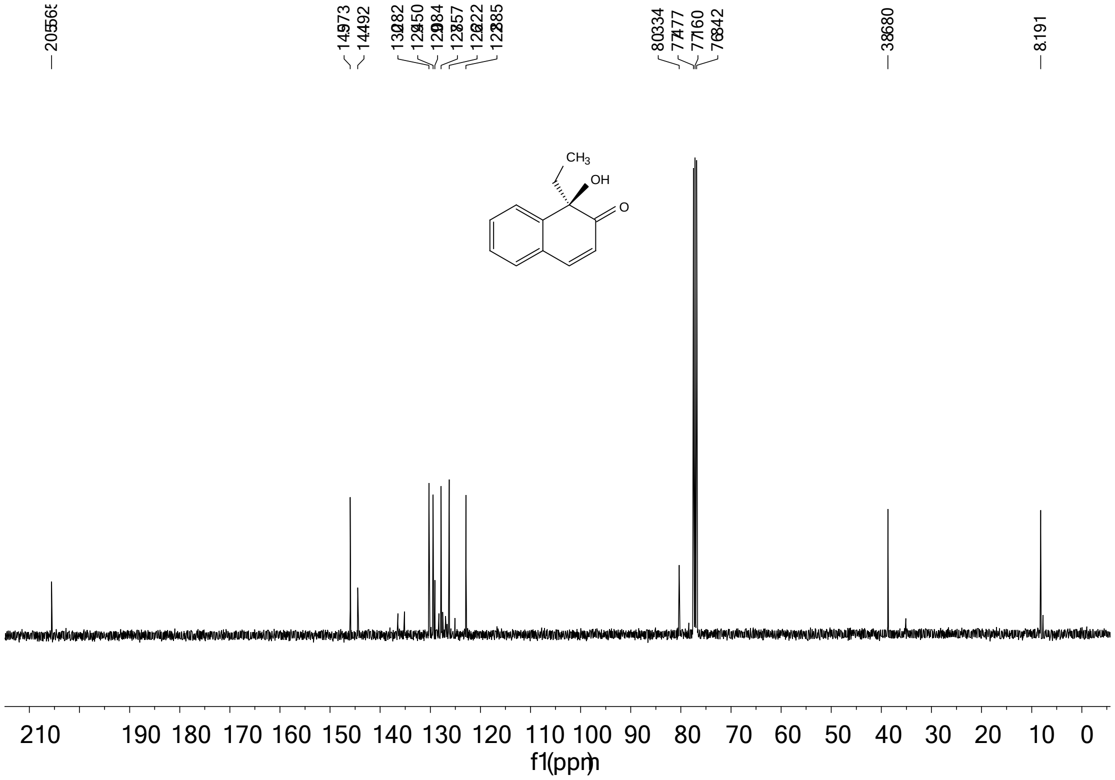

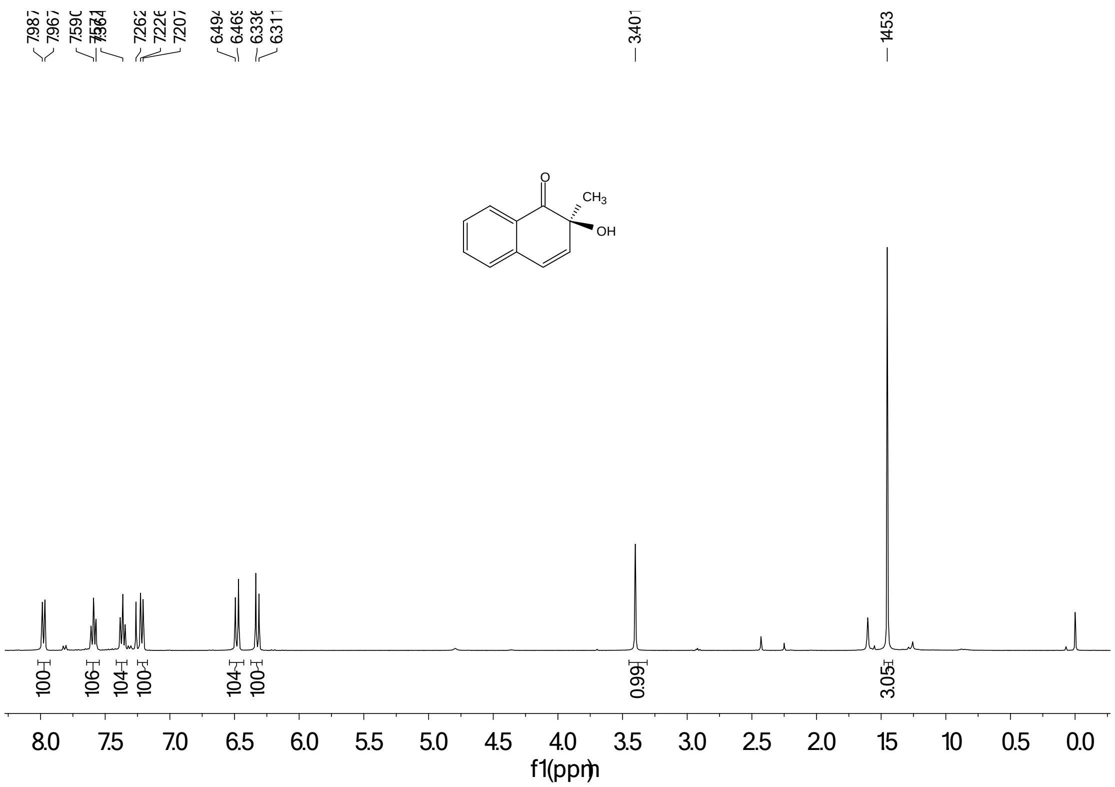

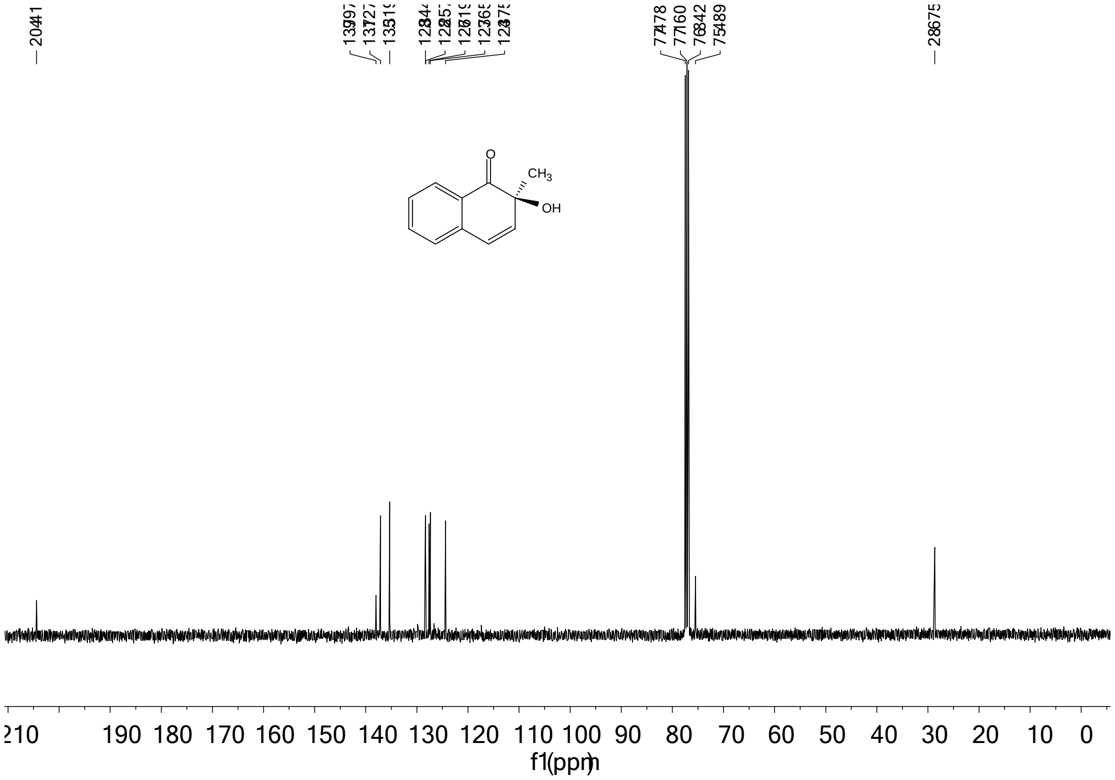

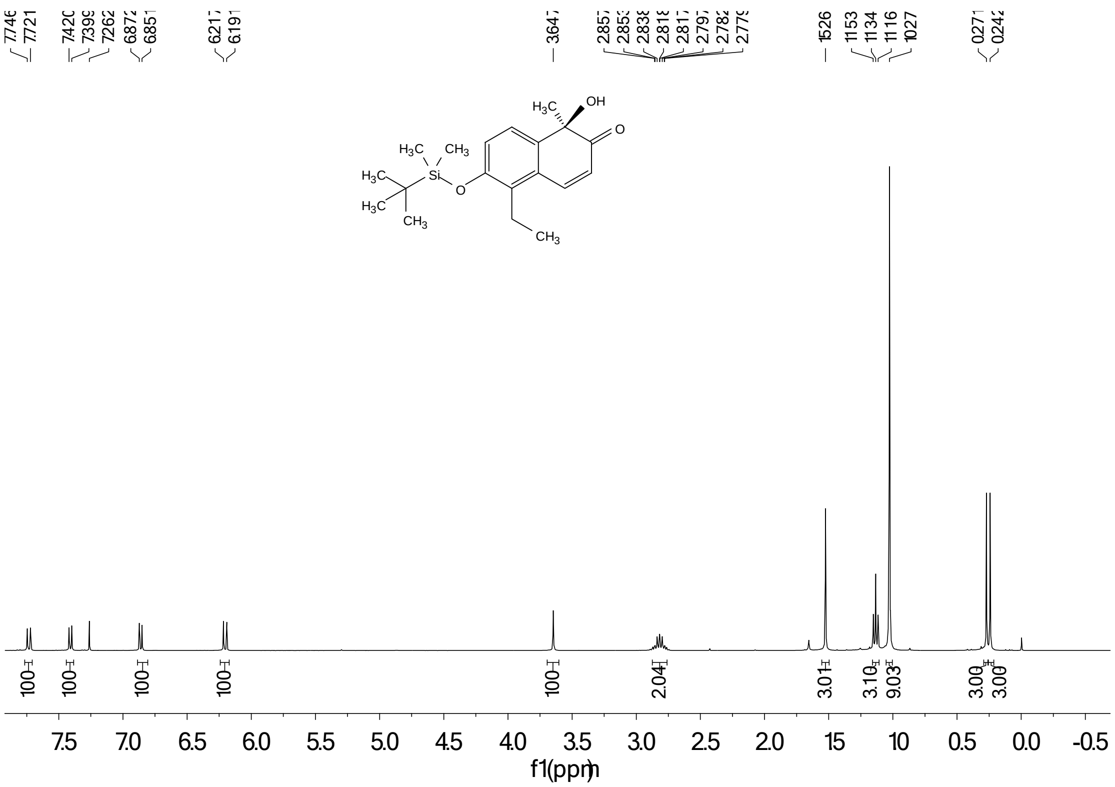
7.5 7.0 6.5 6.0 5.5 5.0 4.5 4.0 3.5 3.0 2.5 2.0 1.5 1.0 0.5 0.0 -0.5
f1 (ppm)

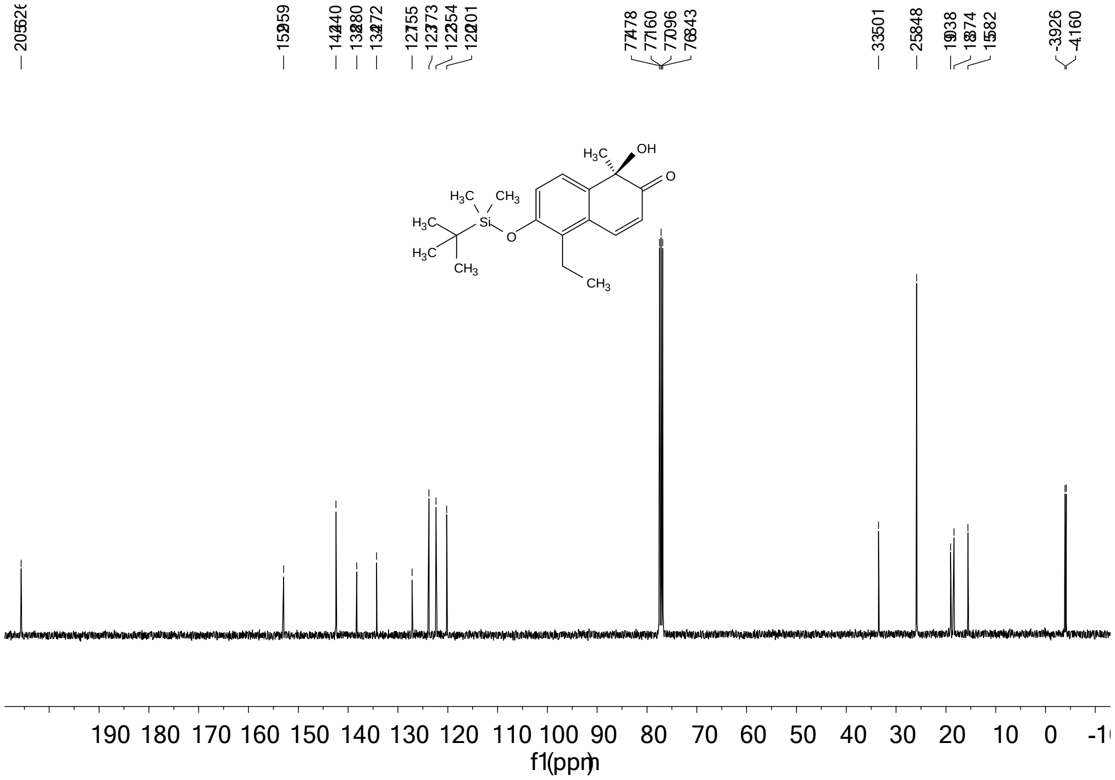
190 180 170 160 150 140 130 120 110 100 90 80 70 60 50 40 30 20 10 0
f1 (ppm)

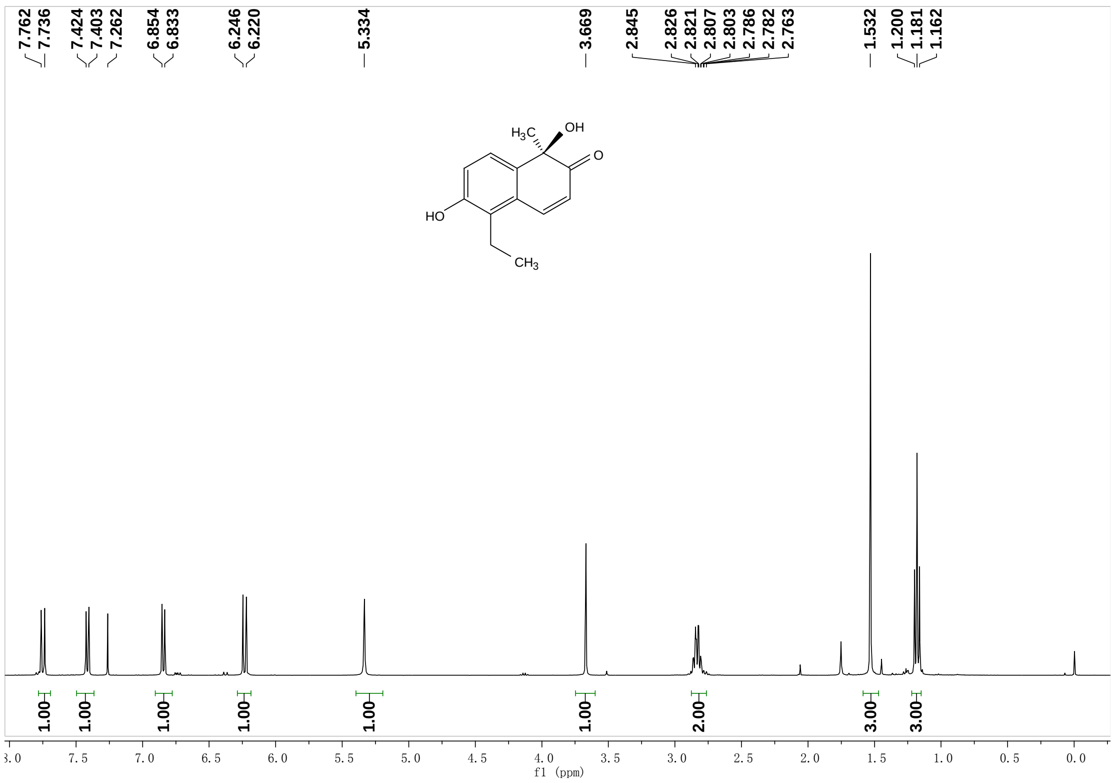

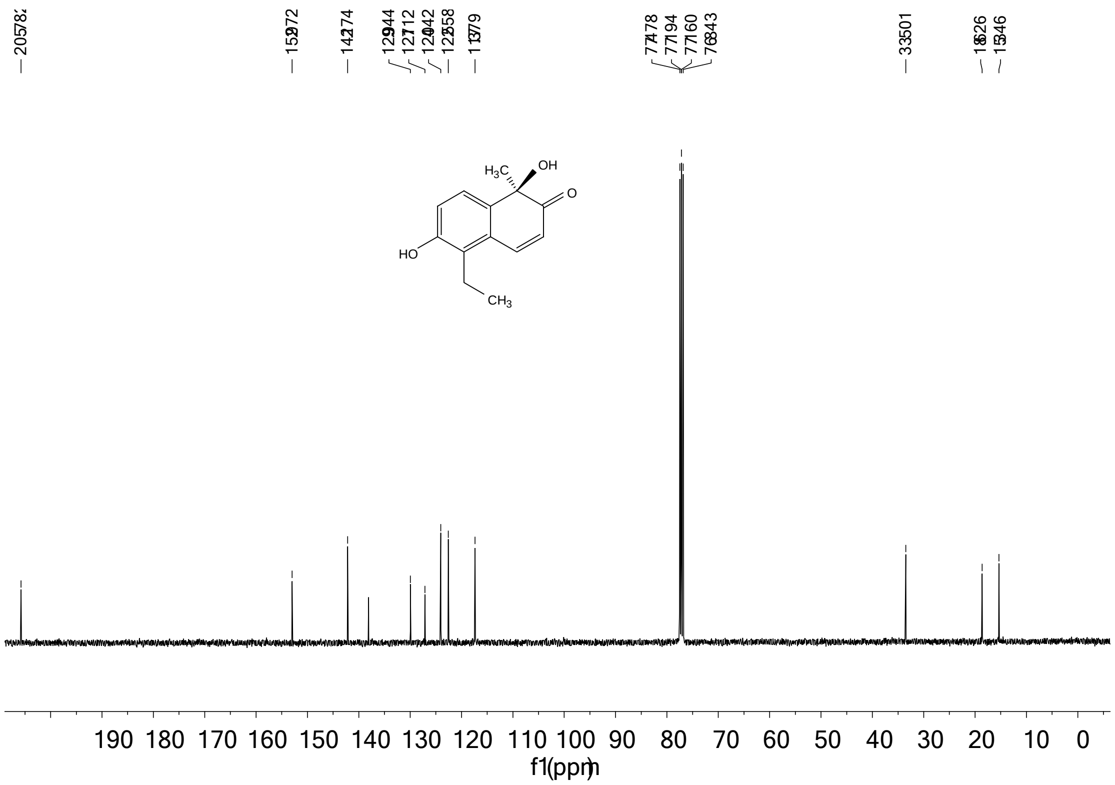

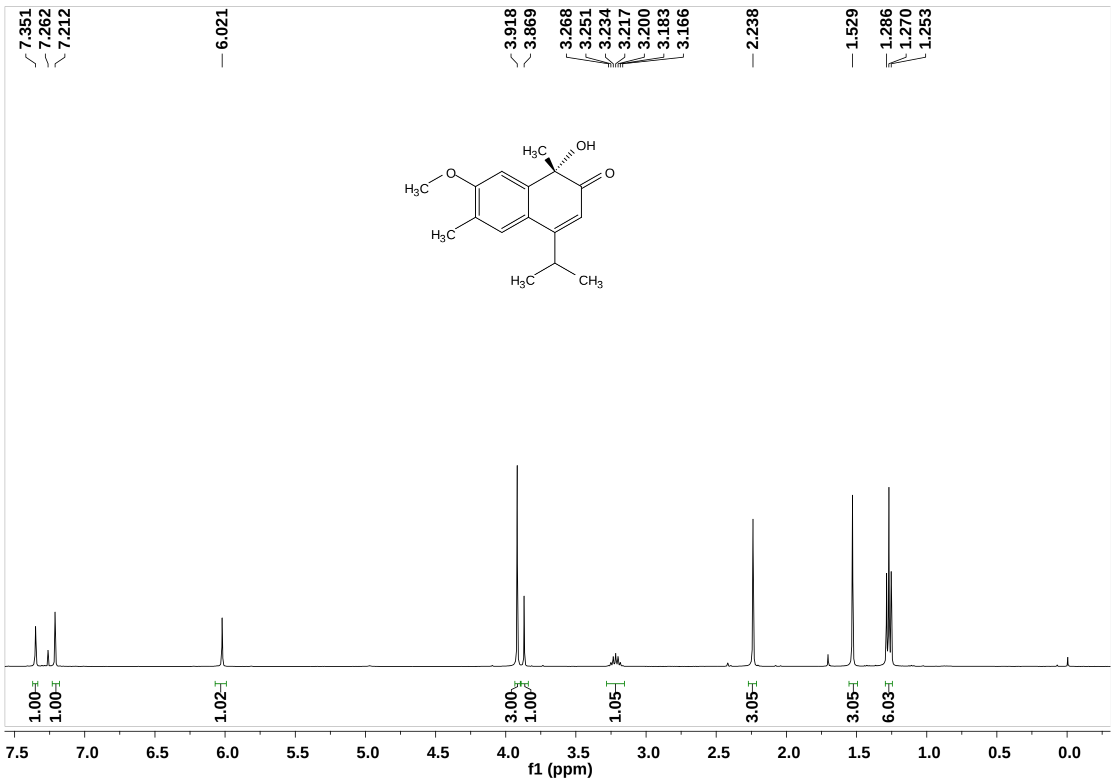

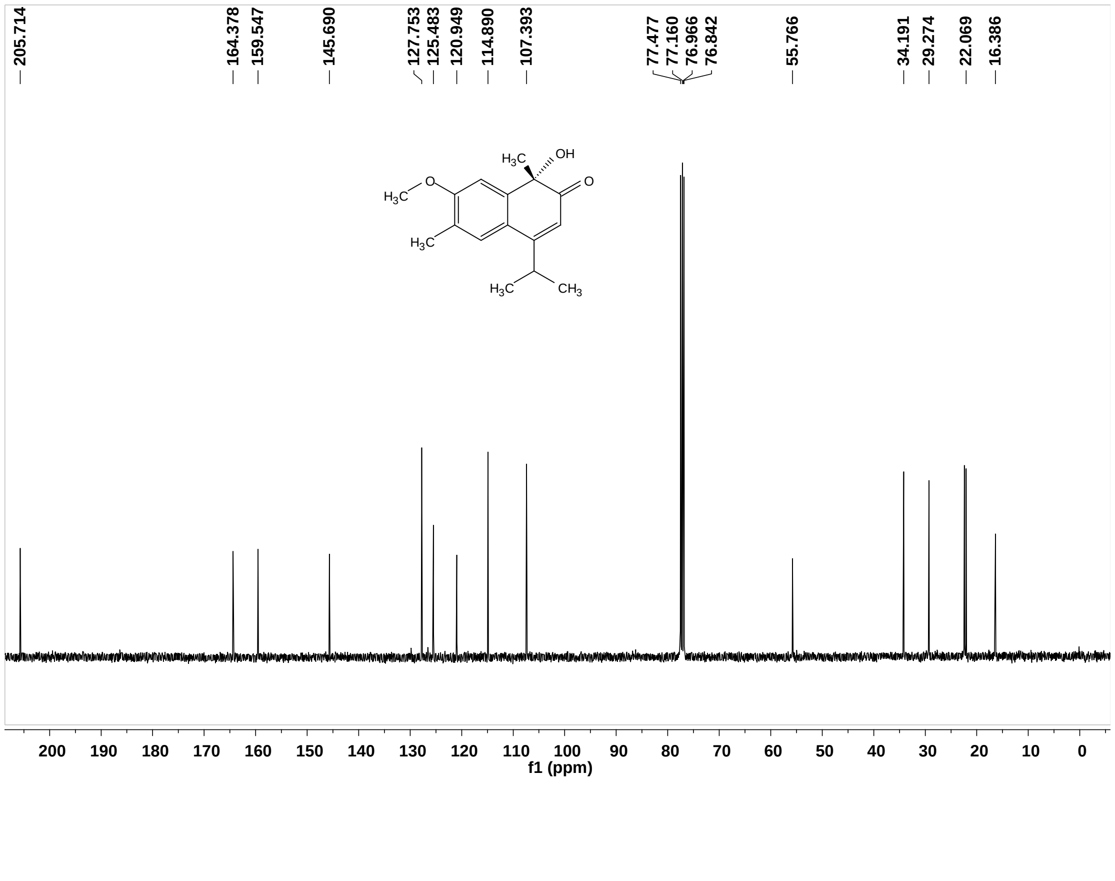

Supplement: Supplementary file 1 [file SC-008-C7SC02809A-s001.pdf]
